# Thiazoles via Formal [4 + 1] of NaSH to (*Z*)-Bromoisocyanoalkenes

**DOI:** 10.1021/acs.joc.4c02691

**Published:** 2025-03-21

**Authors:** Huan Tian, John-Paul R. Marrazzo, Tish Huynh, Fraser F. Fleming

**Affiliations:** † Department of Chemistry, Drexel University, 3401 Chestnut St., Philadelphia, Pennsylvania 19104, United States

## Abstract

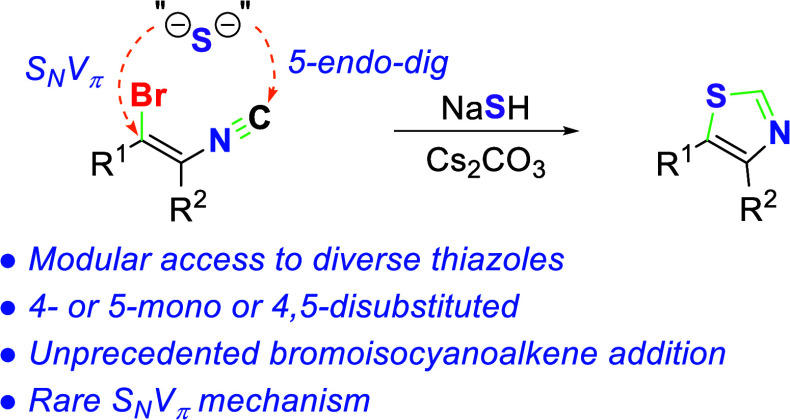

A formal [4 + 1] addition of NaSH to bromoisocyanoalkenes provides substituted thiazoles via a versatile strategy that affords an array of 4- or 5-monosubstituted or 4,5-disubstituted thiazoles. The unique assembly is achieved by a rare S_N_V_π_ displacement of NaSH on (*Z*)-bromoisocyanoalkenes. The versatile strategy addresses the dearth of conjugate additions to alkenes substituted with isocyanides as the sole electron-withdrawing group to provide an array of substituted thiazoles.

## Introduction

**Scheme 1:**
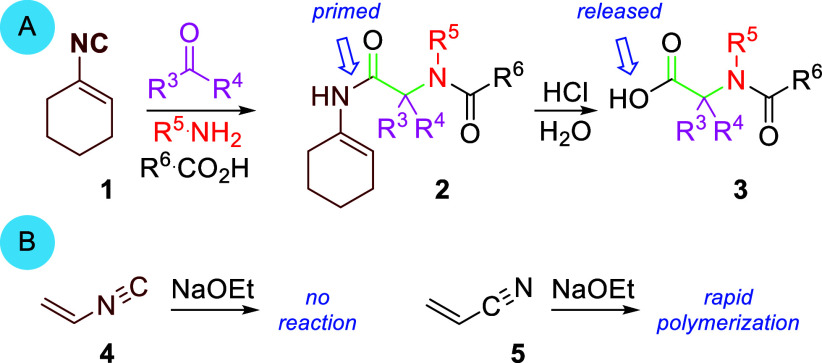
Reactivity Profile of Isocyanoalkenes

Isocyanoalkenes are a small subset of isocyanides whose reactivity is largely unexplored.^1^ The most common use of isocyanoalkenes is as “convertible isocyanides” in multicomponent reactions where the alkene, often cyclohexene (**1**),^2^ serves only to facilitate installation of the terminal isocyanide carbon with subsequent release of the alkene portion (Scheme 1A, **2** → **3**);^3^ there is a virtual absence of reactions involving additions to the C=C bond of isocyanoalkenes.^4^ The stark contrast in the reactivity of alkenes substituted with isocyanides compared to similar electron withdrawing groups is evident when comparing exposure of the parent isocyanoethene (**4**) and the isoelectronic acrylonitrile (**5**) to NaOEt (Scheme 1B): under identical conditions, isocyanoethylene (**4**) is inert whereas acrylonitrile (**5**) rapidly polymerizes.^1^

**Scheme 2:**
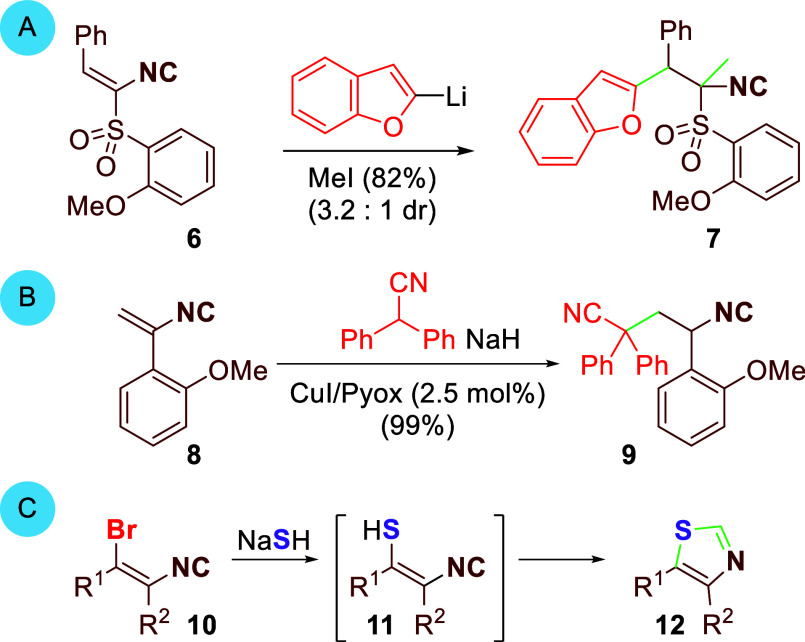
Isocyanoalkene Conjugate-Type Additions

Functionalizing the carbon–carbon double bond of isocyanoalkenes is difficult because the two π-systems are not conjugated.^1^ As a consequence, the β-carbon of the isocyanoalkene is weakly polarized, causing nucleophiles to attack the carbenoid-like terminal isocyanide rather than the olefin.^5^ Nucleophiles only add conjugately to isocyanoalkenes that are additionally substituted with powerful electron-withdrawing groups, such as the isocyano-substituted vinylsulfone **6** (Scheme 2A, **6** → **7**).^6^ A lone exception is the copper(I)-catalyzed addition of polarizable, anionic nucleophiles to α-aryl isocyanoalkene **8** (Scheme 2B, **8** → **9**).^7^ Conspicuously absent from π-additions to isocyanoalkenes are substrates with α-H or α-alkyl substituents, substituents that typically retard conjugate additions to analogous carbonyl-substituted alkenes.^8^

**Figure 1:**
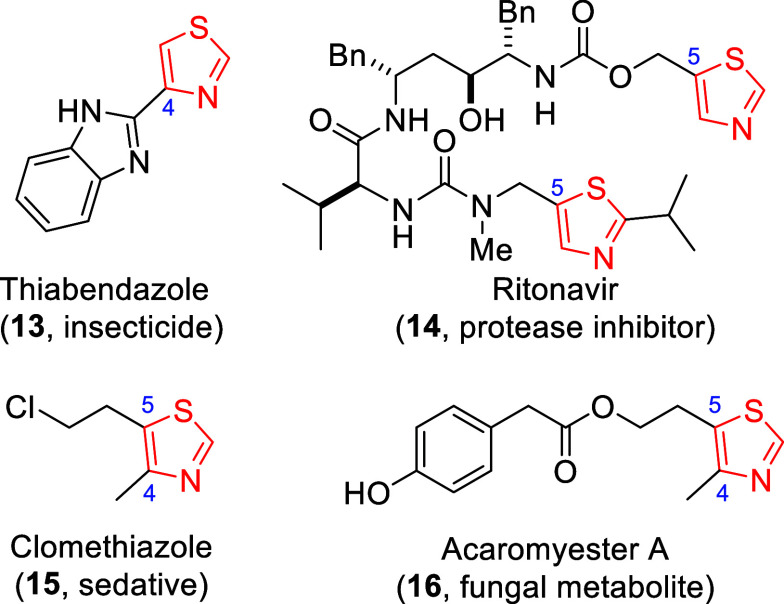
Representative bioactive substituted thiazoles.

The challenge in forging new bonds to the β-carbon of isocyanoalkenes stimulated an unorthodox conjugate addition equivalent to bromoisocyanoalkenes **10** (Scheme 2C). The strategy is predicated on an S_N_V_π_ displacement, rather than an addition–elimination, with NaSH that transiently generates isocyanoenthiols **11**, whose cyclization affordes thiazoles **12**. Described here is the S_N_V_π_ addition–cyclization of NaSH to bromoisocyanoalkenes having only α-alkyl or α-hydrogen substituents **10** (R^2^ = H or alkyl). The strategy rapidly assembles 4- or 5-monosubstituted (cf. **13**(9) and **14**,^10^ respectively) or 4,5-disubstituted thiazoles (cf. **15**(11) and **16**(12)), which are prevalent motifs in an array of agrochemicals (**13**), pharmaceuticals (**14**, **15**),^13^ and natural products (**16**,^14^Figure 1).

## Results and Discussion

**Scheme 3:**
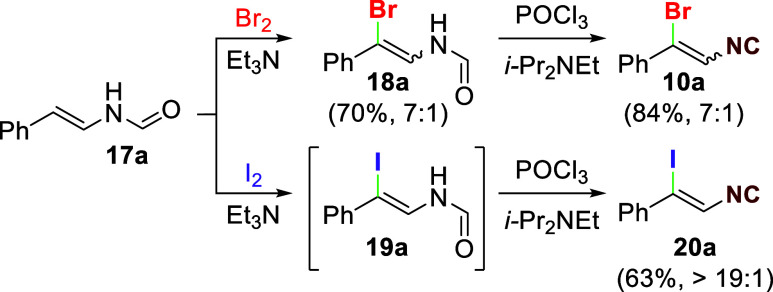
Synthesis of Haloisocyanoalkenes

The requisite β-bromoisocyanoalkenes became readily available through two-step bromination–dehydration of the corresponding vinyl formamides (Scheme 3). Initial forays employed bromination^15^ of the phenyl-substituted vinyl formamide **17a**(16) to access the bromovinylformamide **18a** (70% yield, a 7:1 ratio of geometric isomers), and subsequent dehydration relayed **18a** into the requisite bromoisocyanoalkene **10a** with the same geometric ratio (84% yield). Further optimization revealed that the bromination and dehydration could be telescoped into a more efficient one-pot procedure (**17a** → **10a**, 84% yield). An analogous iodination of **17a** produced **19a**, which was dehydrated *in situ* to afford the iodoisocyanoalkene **20a** as the only detectable diastereomer.^17^

Exploratory additions of NaSH^18^ to bromoisocyanoalkene **10a** were initially performed in methanol to fully dissolve the nucleophile (Table 1, entries 1–3). While acidic conditions were deleterious (Table 1, entry 1), switching to *i*-Pr_2_NEt provided the desired thiazole **12a** in a 1:1 mixture of methanol and water (Table 1, entry 2), in dry methanol (Table 1, entry 3), and in dry ethanol (Table 1, entry 4). Much of the remaining mass was accounted for by the formation of the thioformamide **21a** and the formamide **18a**. Presumably, the thioformamide **21a** arose from the attack of NaSH on the isocyanide followed by tautomerization, while the formamide may arise from hydrolysis of both the isocyanide **10a** and the thioformamide **21a**.^19^

**Table 1:**
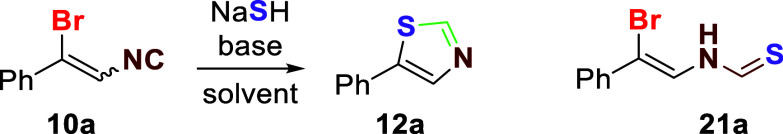
Optimizing the Conversion of **10a** to Thiazole **12a**

entry	solvent	conditions	yield (%)
1	MeOH	AcOH, rt	0
2	MeOH/H_2_O^a^	*i*-Pr_2_NEt, rt then 50 °C	46^b^
3	MeOH	*i*-Pr_2_NEt, rt then 50 °C	54
4	EtOH	*i*-Pr_2_NEt, rt then 50 °C	56^c^
5	CH_3_CN	*i*-Pr_2_NEt	28^d^
6	CH_3_CN	Cs_2_CO_3_	82^e^
7	CH_3_CN	Cs_2_CO_3_ with **20a**	58

a 1:1 ratio.

b Co-isolated an additional 13% of **18a**.

c Co-isolated an additional 11% of **18a**.

d Co-isolated 24% of thioformamide **21a**.

e Recovered 13% of (*E*)-**10a**.

The challenge in minimizing hydrolysis led to a solvent switch^20^ to acetonitrile. Although the mixture formed a suspension, sufficient NaSH dissolved to afford thiazole **12a** with *i*-Pr_2_NEt or even more efficiently when Cs_2_CO_3_ was employed as the base (Table 1, compare entry 5 with entry 6). The comparable addition of NaSH to the corresponding iodoisocyanoalkene **20a** afforded thiazole **12a**, but the yield was considerably lower (Table 1, entry 7); the reduced yield of **20a** appears to be caused by some degradation of iodoisocyanoalkene **20a** under the reaction conditions.

The telescoped bromination–dehydration and NaSH addition–cyclization protocols were applied to a series of substrates to explore the reaction scope (Table 2). Initially, modest changes were probed by varying the *para* and *ortho* electron-withdrawing and electron-donating substituents in the phenyl ring (Table 2, entries 1 and 2 and entries 3 and 4 respectively), followed by additions to disubstituted *m,p-* and *o,o*-aromatics (Table 2, entries 5–7); the *o*-substituted bromoisocyanoalkenes were generated with particularly high *Z/E* ratios (compare, for example, **10e** with **10f**, Table 2 entry 4 vs entry 5). The sequential bromination–dehydration–cyclization protocol was extended to naphthyl- and carbazole-substituted bromoisocyanoalkenes (Table 2, entries 8 and 9, respectively) and to bromoisocyanoalkenes bearing only aliphatic substituents (Table 2, entries 10–12). The protocol was equally effective with bromoisocyanoalkenes having only α-substituents (Table 2, entries 13–16) and with the 1,2-disubstituted bromoisocyanoalkene **10r** that afforded the 4,5-fused thiazole **12r** (Table 2, entry 17).

**Table 2:**
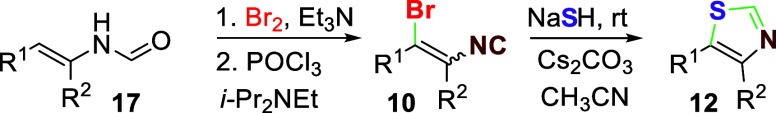

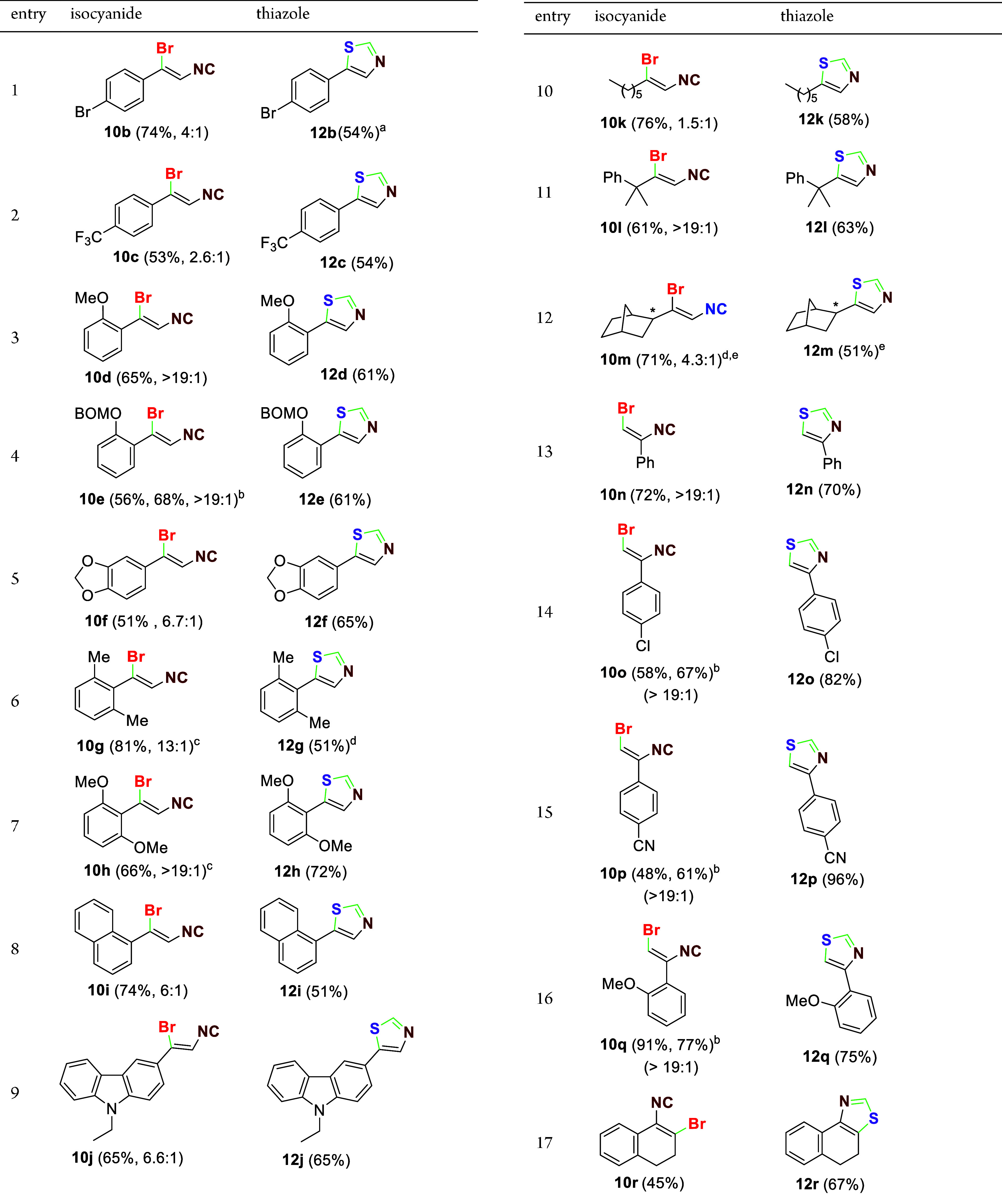
Bromoisocyanoalkene and Thiazole Synthesis

a Performed in EtOH.^21^

b The bromination and dehydration were performed in separate steps in the respective yields shown.

c 1 mmol scale.

d The yield for a 1 mmol scale reaction was 47%.

e A 1:1.1 ratio of *endo* and *exo* diastereomers.

**Scheme 4:**
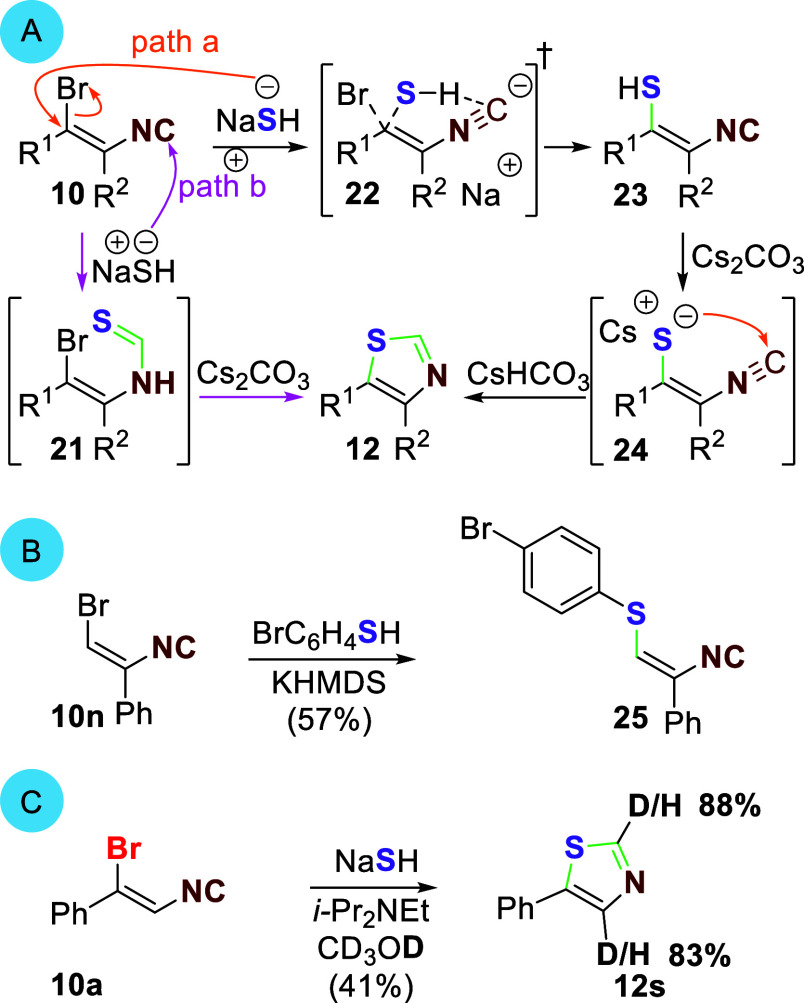
NaSH Isocyanoalkene Addition Mechanism

The addition of NaSH to the bromoisocyanoalkenes is most consistent with an S_N_V_π_ mechanism (Scheme 4A, **10 → 22 → 23**, path a).^22^ Carefully monitoring the addition of NaSH to a 7:1 *Z/E* mixture of **10a** led to the recovery of unreacted (*E*)-**10a**, in addition to thiazole **12a** (Table 1, entry 6); attempts to force the conversion of (*E*)-**10a** to thiazole **12a** led to extensive degradation. Recovery of (*E*)-**10a** is not compatible with an addition–elimination mechanism, particularly as both (*E*)- and (*Z*)-geometric isomers react smoothly with nucleophiles when isocyanoalkenes are substituted by electron-withdrawing groups.^6,23^ However, an S_N_V_π_ attack of NaSH on the π* orbital of a (*Z*)-isocyanoalkene should, based on similar displacements, be relatively facile.^24^ Potential hydrogen bonding between the hydrogen sulfide and the isocyanide may further favor transition structure **22**.^25^

The S_N_V_π_ addition of sodium hydrogen sulfide to bromoisocyanoalkenes **10** via **22** affords enethiols **23**. Deprotonation of the enethiol **23** followed by an intramolecular attack on the isocyanide (**24 → 12**) should be facile based on the analogous cyclization of isocyanoenolates.^26^ Alternatively, NaSH attack on the isocyanide might form the thioformamide **21**, whose cyclization via a base-assisted azathio-pentadienyl-type cyclization,^27^ and bromide elimination would similarly lead to **12** (Scheme 4A, path b, **10 → 21 → 12**). The inability to cyclize thioformamide **21a** (**21**, R^1^ = Ph, R^2^ = H) under the reaction conditions^28^ and the retentive displacement of bromide by bromophenylsulfenylate on **10n** (Scheme 4B) to afford **25** are more consistent with an initial attack of NaSH on the bromine-substituted carbon in the first step (**10 → 22**, path a).^29^ Further support for path a comes from an NaSH addition to **10a** in deuterated methanol, which led to deuterium incorporation at both C2 and C4 (Scheme 4C, **12s**), labeling that is consistent with deuterium incorporation via enethiolate **24** (R^1^ = Ph, R^2^ = H/D).^30^

## Conclusion

The addition of NaSH to bromoisocyanoalkenes provides a versatile synthesis of substituted thiazoles. The unique strategy employs a rare S_N_V_π_ displacement that affords intermediate enethiols whose cyclization efficiently affords 4- and 5-substituted or 4,5-disubstituted thiazoles. The [4 + 1] strategy is synthetically efficient, addresses the difficulty of performing conjugate additions to isocyanoalkenes, and is mechanistically important in addressing the challenge of manipulating the C=C bond of isocyanoalkenes.

## Data Availability

The data underlying this study are available in the published article and in the online Supporting Information.

## Supporting Information Available

Experimental procedures, compound characterization, and copies of NMR spectra (PDF)
FAIR data, including the primary NMR FID files, for compounds **17e**, **17g**, **17h**, **17j**, **17l**, **17m**, **17o**-**17r**, **18a**, **18e**, **18n**, **18o**-**18q**, **10a**–**10s**, **12a**–**12r**, **20a**, **21a**, and **25** (ZIP)
