# Supplementary material for: Thiazoles via Formal [4 + 1] of NaSH to (Z)-Bromoisocyanoalkenes
Source: J Org Chem. 2025 Mar 21;90(13):4513–7. doi: 10.1021/acs.joc.4c02691 (PMC11976862; doi:10.1021/acs.joc.4c02691)
Supplement: Supplementary file 1 — jo4c02691_si_001.pdf [file jo4c02691_si_001.pdf]

Supporting Information

Thiazoles via Formal [4+1] of NaSH to *Z*-Bromoisocyanoalkenes

Huan Tian, John-Paul R. Marrazzo, Tish Huynh, and Fraser F. Fleming*

Department of Chemistry, Drexel University, 32 South, 32nd St., Philadelphia, PA 19104

Table of Contents

		^1H and ^{13}C NMR Spectra
**General Experimental Procedures**	S10	
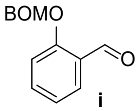 i	S10	S53
**General Procedure for Preparing Iodoalkenes**	S11	
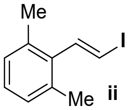 ii	S12	S54
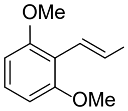 iii	S13	S55
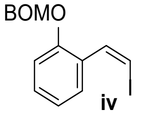 iv	S13	S56
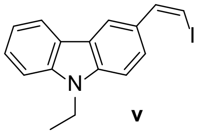 v	S14	S57
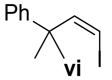 vi	S15	S58

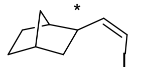
vii S15 S59

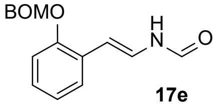
17e S16 S60

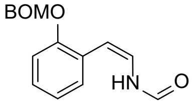
17e' S16 S61

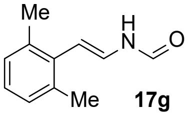
17g S17 S62

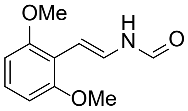
17h S18 S63

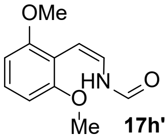
17h' S18 S64

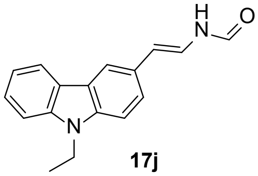
17j S19 S65

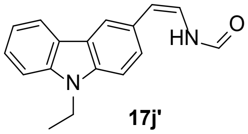
17j' S19 S66

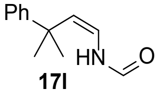
17l S21 S67

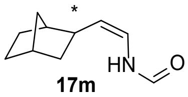
17m S21 S68

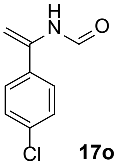
17o S22 S69

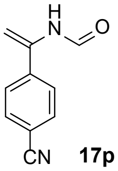
17p S23 S70

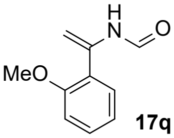
17q S24 S71

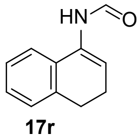
17r S24 S72

General Bromovinylformamide Synthesis S25

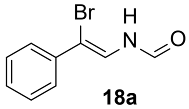
18a S26 S73

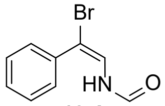
18a' S26 S74

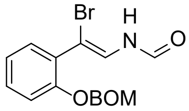
18e S27 S75

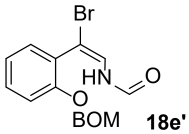
18e' S27 S76

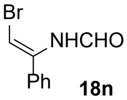
18n S28 S77

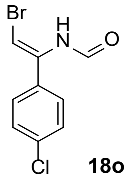 **18o**	S28	S78
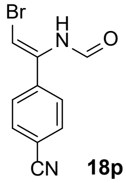 **18p**	S29	S79
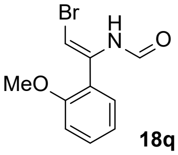 **18q**	S29	S80
**General Bromoalkeneisocyanide Synthesis A**	S30	
**General Bromoalkeneisocyanide Synthesis B**	S30	
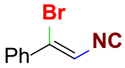 **10a** 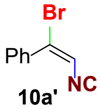 **10a'**	S30	S81
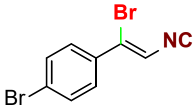 **10b**	S31	S82<br>Fig. S1. gNOESYfor **10b**<br>S83
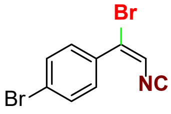 **10b'**	S31	S84
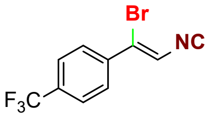 **10c**	S32	S85
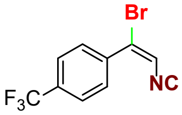 **10c'**	S32	S86

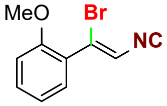 **10d**	S32	S87
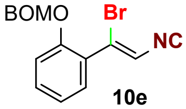 **10e**	S33	S88
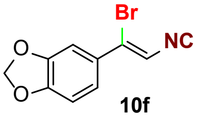 **10f**	S33	S89
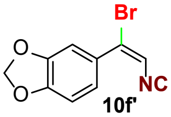 **10f'**	S33	S90
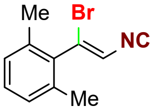 **10g** 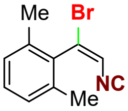 **10g'**	S34	S91
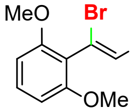 **10h**	S35	S92
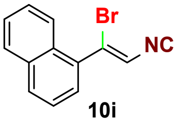 **10i**	S35	S93
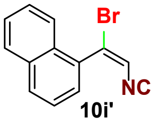 **10i'**	S35	S94
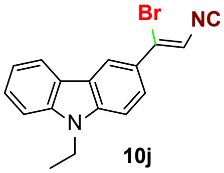 **10j**	S36	S95

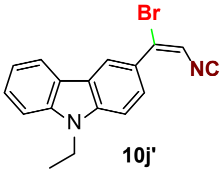 **10j'**	S36	S96
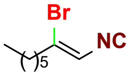 **10k**	S37	S97
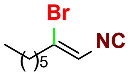 **10k** 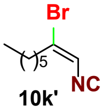 **10k'**	S37	S98
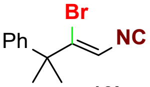 **10l**	S37	S99
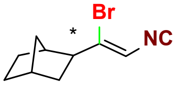 **10m**	S38	S100
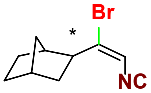 **10m'**	S38	S101
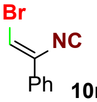 **10n**	S39	S102
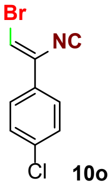 **10o**	S39	S103
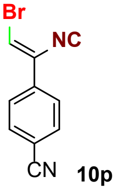 **10p**	S40	S104

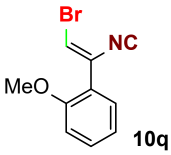
10q S41 S105

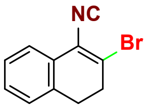
10r S41 S106

General thiazole synthesis A S42

General thiazole synthesis B S42

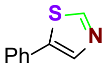
12a S42 S107

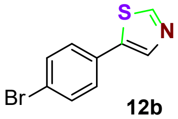
12b S42 S108

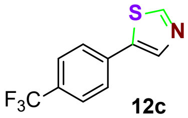
12c S43 S109

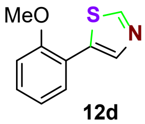
12d S43 S110

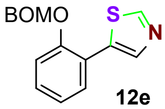
12e S44 S111

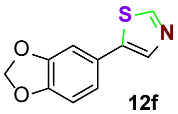
12f S44 S112

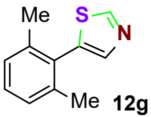	12g	S44	S113
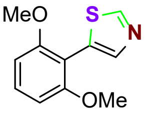	12h	S45	S114
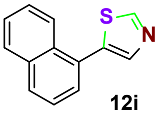	12i	S45	S115
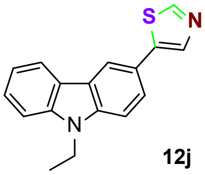	12j	S46	S116
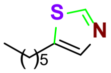	12k	S46	S117
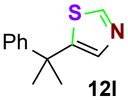	12l	S46	S118
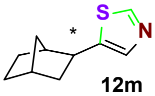	12m	S47	S119
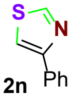	12n	S47	S120
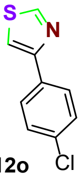	12o	S48	S121

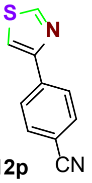 12p	S48	S122
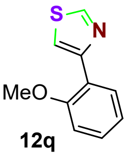 12q	S48	S123
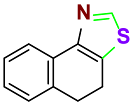 12r	S49	S124
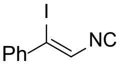 20a	S49	S125<br>Fig. S2. gNOESY for **20a** S126
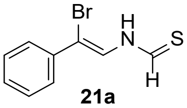 21a	S50	S127
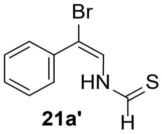 21a'	S50	S128
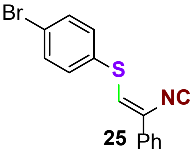 25	S51	S129<br>Fig. S3. gNOESY for **25**<br>S130
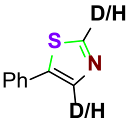 12s	S52	S131
References	S132	

General Experimental Procedures. All nonaqueous reactions were performed in oven- or

flame-dried glassware under a nitrogen atmosphere. All chemicals were purchased from commercial vendors and used as received unless otherwise specified. Anhydrous tetrahydrofuran (THF), and dioxane were distilled from benzophenone-sodium under N_2 before use. Dichloromethane and diisopropylamine were obtained from a solvent purification system (Innovative Technology Inc., model PS-MD7). Reactions were magnetically stirred and monitored by thin layer chromatography (TLC) using 250 μm precoated silica gel plates. Thermolyses were performed by immersing the reaction flask immersed in a mineral oil bath that was heated on a hotplate stirrer. ^{1}H NMR and ^{13}C NMR high resolution nuclear magnetic resonance spectra were recorded on a Varian Inova 400 (400 MHz/101 MHz) instrument. Chemical shifts are reported relative to TMS (*δ* 0.00) for ^{1}H NMR and chloroform (*δ* 77.16) for ^{13}C NMR. Structural assignments were made with additional information from gNOESY, gCOSY, gHSQC, and gHMBC experiments. IR spectra were recorded as thin films (PerkinElmer Spectrum 100 FT-IR Spectrometer). High-resolution mass spectra were obtained on a Bruker 12.0 Tesla APEX – Qe FTICR-MS with and Apollo II ion source (positive electrospray ionization) and a Thermo-Finnigan LTQ-FT 7T FT-ICR spectrometer with an atmospheric pressure chemical ionization (APCI) source with direct infusion run in positive ion mode at 5 kV. Purification of the vinyl formamides and the isocyanides was performed on silanized C-2 silica gel, prepared following the published procedure, [1] using a Reveleris X2 Flash Chromatography/Prep Purification System.

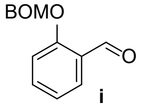

i

*2-((Benzyloxy)methoxy)benzaldehyde (**i**)*. Following a published procedure,[2] neat benzyl chloromethyl ether (961.8 mg, 6.14 mmol) was added to a CH_2Cl_2 solution (10.2 mL) of salicylaldehyde (500 mg, 4.09 mmol) and *i*-Pr_2NEt (1.6 g, 12.3 mmol). After 4 h,

saturated, aqueous NH_4Cl was added, the phases were separated, and then the aqueous phase was extracted with CH_2Cl_2 (10 mL x 3). The organic phase was combined, washed with water (10 mL x 1) and brine (10 mL x 1), dried (Na_2SO_4), and then concentrated. Purification of the crude aldehyde by MPLC (25-g cartridge silica gel, 5% EtOAc/Hexanes), furnished 429.0 mg (43%) of (2-((benzyloxy)methoxy)benzaldehyde (**i**) as clear oil: IR (ATR) 3032, 2865, 2762, 1687 cm^{-1}; ^{1}H NMR (400 MHz, $CDCl_3$) δ 10.49 (s, 1H), 7.86 (dd, *J* = 7.7, 1.7 Hz, 1H), 7.54 (ddd, *J* = 8.8, 7.7, 1.7 Hz, 1H), 7.38 – 7.28 (m, 6H), 7.11 – 7.07 (m, 1H), 5.43 (s, 2H), 4.77 (s, 2H); ^{13}C{1H} NMR (101 MHz, $CDCl_3$) δ 189.7, 159.6, 136.8, 135.9, 128.5, 128.3, 128.1, 128.0, 125.4, 121.9, 115.0, 92.5, 70.8; HRMS (+EI) m/z [M$^+$] Calcd. for $C_{15}H_{14}O_3$, 242.0943 found 242.0933.

General Procedure for Preparing Iodoalkenes

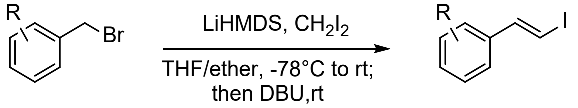

Method A: Following a previous procedure,[3] a THF solution (0.47 mL, 4.2 M) of CH_2I_2 (2 mmol, 2 equiv.) was added dropwise to a -78 °C, THF-ether solution (1M, 1:1 ratio) of LiHMDS (2 mmol, 2 equiv.) in the dark. After 20 min, a THF solution (0.76 mL, 1.3 M) of the bromide (1 mmol, 1 equiv.) was added dropwise and then the reaction mixture was allowed to slowly warm to rt. After 16 h, neat DBU (2 mmol, 2 equiv.) was added dropwise. After 1 h, the mixture was filtered through a plug of silica (1 x 1 cm) capped with a layer of Celite (1 x 1 cm) and then the filtrate was evaporated under reduced pressure. The resulting residue was purified by flash chromatography to provide the pure vinyl iodide.

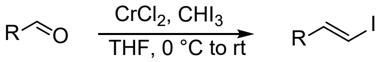

Method B: Following a previously published procedure,[4] a THF (4 mL, 0.25 M) solution of the aldehyde (1 mmol, 1 equiv.) was added to a vigorously stirred, 0 °C, THF suspension (80 mL, 0.1 M) of $CrCl_2$ (8 mmol, 8 equiv.) and iodoform (2 mmol, 2 equiv.) in the dark. After 3 h, water was added and then the phases were separated. The aqueous phase was separated and extracted with diethyl ether, and then the combined organic extract was washed with water (10 mL x 1), brine (10 mL), and dried over with Na_2SO_4. The solvent was removed in vacuo and then the residue was purified by flash chromatography to provide the pure vinyl iodide.

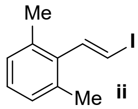
ii

(E)-2-(2-Iodovinyl)-1,3-dimethylbenzene ***(ii)***. Following the general iodoalkene synthesis method A with 2-(bromomethyl)-1,3-dimethylbenzene[5] (1.0 g, 5.0 mmol), CH_2I_2 (2.7 g, 10.1 mmol), LiHMDS (10.1 mL, 10.1 mmol) and DBU (1.53 g, 10.1 mmol) with purification by MPLC (12-g cartridge silica gel, 100% Hexanes), furnished 1.25 g (96%) of *(E)-2-(2-iodovinyl)-1,3-dimethylbenzene* ***(ii)*** as a clear oil: IR (ATR) 3059, 1593 cm^{-1}; ^{1}H NMR (400 MHz, $CDCl_3$) δ 7.45 (d, J = 15.1 Hz, 1H), 7.13 - 7.01 (m, 3H), 6.32 (d, J = 15.1 Hz), 2.28 (s, 6H); ^{13}C{1H} NMR (101 MHz, $CDCl_3$) δ 143.4, 137.6, 135.6, 127.9, 127.4, 80.2, 20.9; HRMS (+EI) *m/z* [M]$^+$ calcd. for $C_{10}H_{11}I$ 257.9906, found 257.9898.

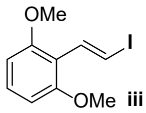
iii

(*E)-2-(2-Iodovinyl)-1,3-dimethoxybenzene.* Following the General Iodoalkene Synthesis Method B with 2,6-dimethoxybenzaldehyde (500 mg, 3.0 mmol), $CrCl_2$ (1.48 g, 12.0 mmol), and iodoform (1.54 g, 3.9 mmol) with purification by MPLC (12-g cartridge silica gel, 1% EtOAc in Hexanes), furnished 506 mg (58 %) of a 1.5:1 ratio of *trans:cis* geometric isomers of 2-(2-iodovinyl)-1,3-dimethoxybenzene (**iii**) as a white solid: mp = 68.5 – 70.3 °C; IR 2938, 2836, 1588, 1464 cm^{-1}; HRMS (+EI) *m/z* [M]$^+$ calcd. for $C_{10}H_{11}IO_2$ 289.9804, found

289.9829. For *trans*-2-(2-iodovinyl)-1,3-dimethoxybenzene: ^{1}H NMR (400 MHz, $CDCl_3$) δ 7.83 (d, J = 14.8 Hz, 1H), 7.28 – 7.24 (d, J = 14.8 Hz, 1H), 7.20 (t, J = 8.4 Hz, 1H), 6.54 (d, J = 8.4 Hz, 2H), 3.84 (s, 6H); ^{13}C{1H} NMR (101 MHz, $CDCl_3$) δ158.0, 135.3, 129.1, 114.6, 103.79, 82.4, 55.8. For *cis*-2-(2-iodovinyl)-1,3-dimethoxybenzene: ^{1}H NMR (400 MHz, $CDCl_3$) δ 7.32 – 7.27 (m, 1H), 7.26 – 7.23 (m, 1H), 6.82 (d, J = 8.1 Hz, 1H), 6.58 (d, J = 8.3 Hz, 2H), 3.84 (s, 6H); ^{13}C{1H} NMR (101 MHz, $CDCl_3$) δ 157.1, 134.2, 129.5, 115.6, 103.81, 86.8, 55.7.

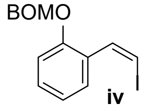

(Z)-1-((Benzyloxy)methoxy)-2-(2-iodovinyl)benzene (**iv**). Following a modification of the published procedure, [6] a THF solution (0.2 M) of 2-((benzyloxy)methoxy)benzaldehyde (**i**, 1.00 g, 4.13 mmol) was added to a vigorously stirred, -78 °C, suspension of iodomethyltriphenylphosphonium iodide (3.06 g, 5.78 mmol) and NaHMDS (5.8 mL, 5.78 mmol). The reaction was allowed to warm slowly to rt. After 16 h, saturated, aqueous $NaHCO_3$ (2 mL) was added. The mixture was diluted with hexanes (2 x 10 mL), and then filtered through a pad of Celite®. The phases were separated and then the organic phase was dried (Na_2SO_4), filtered and concentrated in vacuo. Purification by MPLC (12-g cartridge filled with silica gel, EtOAc/Hexanes 3:97) furnished 1.20 g (80 %) of (*Z*)-1-((benzyloxy)methoxy)-2-(2-iodovinyl)benzene (**iv**) as a clear oil: IR 3062, 3034, 2902, 1598 cm^{-1}; ^{1}H NMR (400 MHz, $CDCl_3$) δ 7.75 (dd, J = 7.7, 1.1 Hz, 1H), 7.41 (d, J = 8.5 Hz, 1H), 7.38 – 7.27 (m, 6H), 7.20 (dd, J = 8.3, 1.2 Hz, 1H), 7.09 – 7.03 (m, 1H), 6.61 (d, J = 8.5 Hz, 1H), 5.32 (s, 2H), 4.72 (s, 2H); ^{13}C{1H} NMR (101 MHz, $CDCl_3$) δ 154.5, 137.1, 135.1, 129.7, 129.0, 128.5, 128.0, 127.9, 126.9, 121.2, 114.5, 92.6, 81.2, 70.3; HRMS (+EI) m/z $[M]^+$ calcd. for $C_{16}H_{15}IO_2$ 366.0117, found 366.0111.

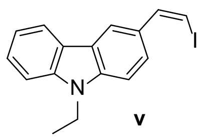

*(Z)-9-Ethyl-3-(2-iodovinyl)-9H-carbazole (**v**).* Following a modification of the published procedure,[6] a THF solution (0.2 M) of 9-ethyl-9H-carbazole-3-carbaldehyde (1.00 g, 4.47 mmol) was added to a vigorously stirred, -78 °C, suspension of iodomethyltriphenylphosphonium iodide (3.2 g, 6.27 mmol) and NaHMDS (3.2 mL, 6.27 mmol). The reaction was allowed to warm slowly to rt. After 16 h, saturated, aqueous $NaHCO_3$ (2 mL) was added. The mixture was diluted with hexanes (2 x 10 mL), and then filtered through a pad of Celite®. The phases were separated and then the organic phase was dried (Na_2SO_4), filtered and concentrated in vacuo. Purification by MPLC (12-g cartridge filled with silica gel, EtOAc/Hexanes 3:97) furnished 941 mg (60 %) of *(Z)-9-ethyl-3-(2-iodovinyl)-9H-carbazole* (***vii***) as a yellow oil consisting of an inseparable 6.8:1 mixture of *cis*- and *trans*-iodoalkenes: IR 3051, 2973, 1626, 1596 cm^{-1}; HRMS (+EI) *m/z* $[M]^+$ calcd. for $C_{16}H_{15}IN$ 348.0249, found 348.0251. For the *cis*-alkene: ^1H NMR (400 MHz, $CDCl_3$) δ 8.48 – 8.46 (m, 1H), 8.17 – 8.10 (m, 1H), 7.81 (dd, *J* = 8.5, 1.7 Hz, 1H), 7.53 – 7.44 (m, 2H), 7.44 – 7.38 (m, 2H), 7.31 – 7.22 (m, 1H), 6.48 (d, *J* = 8.5 Hz, 1H), 4.38 (q, *J* = 7.2 Hz, 2H), 1.45 (t, *J* = 7.2 Hz, 3H); $^{13}C\{1H\}$ NMR (101 MHz, $CDCl_3$) δ 145.6, 140.5, 139.87, 127.3, 126.4, 125.9, 122.94, 122.61, 120.7, 120.54, 119.1, 108.63,108.0, 76.1, 37.7, 13.9. For the minor *trans*-alkene: ^1H NMR (400 MHz, $CDCl_3$) δ 8.34 (s,1H), 8.28 – 8.26 (m, 1H), 8.10 – 8.07 (m, 1H), 8.03 – 8.01 (m, 1H), 7.65 - 7.55 (m, 2H), 7.35 (t, *J* = 8.8 Hz, 2H), 6.76 (d, *J* = 14.8 Hz, 1H), 4.41 – 4.32 (m, 2H), 1.46 – 1.39 (m, 3H); $^{13}C\{1H\}$ NMR (101 MHz, $CDCl_3$) δ 140.33, 139.85, 139.0, 129.1, 126.0, 123.7, 123.0, 122.63, 120.49, 119.2, 118.4, 108.7, 108.57, 72.6, 37.7, 13.9.

*(Z)-(4-iodo-2-methylbut-3-en-2-yl)benzene (**vi**).* Following a modification of the published procedure,[6] a THF solution (0.2 M) of 2-methyl-2-phenylpropanal[7] (0.1 g,

0.67 mmol) was added to a vigorously stirred, -78 °C, suspension of iodomethyltriphenylphosphonium iodide (0.5 g, 0.94 mmol) and NaHMDS (0.17 g, 0.94 mmol). The reaction was allowed to warm slowly to rt overnight and then saturated, aqueous $NaHCO_3$ (2 mL) was added. The mixture was diluted with hexanes (2 x 10 mL), and then filtered through a pad of Celite®. The phases were separated and then the organic phase was dried (Na_2SO_4), filtered and concentrated in vacuo. Purification by MPLC (4-g cartridge filled with silica gel, 100 % Hexanes) furnished 102 mg (56 %) of *(Z)*-(4-iodo-2-methylbut-3-en-2-yl)benzene (**v**) as a clear oil: IR 3059, 2965, 2869, 1601cm^{-1}; ^{1}H NMR (400 MHz, $CDCl_3$) δ 7.40 – 7.28 (m, 4H), 7.24 – 7.18 (m, 1H), 6.85 (d, J = 8.3 Hz, 1H), 6.32 (d, J = 8.3 Hz, 1H), 1.54 (s, 6H); ^{13}C{1H} NMR (101 MHz, $CDCl_3$) δ 148.9, 147.6, 128.2, 126.9, 125.8, 78.6, 42.3, 29.8; HRMS (+APPI) *m/z* [M]$^+$ calcd. for $C_{11}H_{13}I$ 272.0056, found 272.0068.

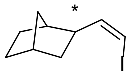

vii

*((1S,4R)-2-((Z)-2-iodovinyl)bicyclo[2.2.1]heptane (**vii**)*. Following a modification of the published procedure,[6] a THF solution (0.2 M) of (1*R*,2*R*,4*S*)-bicyclo[2.2.1]heptane-2-carbaldehyde [8] (0.5 g, 4.03 mmol) was added to a vigorously stirred, -78 °C, suspension of iodomethyltriphenylphosphonium iodide (3.0 g, 5.64 mmol) and NaHMDS (1.03 g, 5.64 mmol). The reaction was allowed to warm slowly to rt overnight and then saturated, aqueous $NaHCO_3$ (2 mL) was added. The mixture was diluted with hexanes (2 x 10 mL), and then filtered through a pad of Celite®. The phases were separated and then the organic phase was dried (Na_2SO_4), filtered and concentrated in vacuo. Purification by MPLC (12-g silica gel cartridge, 100 % Hexanes) furnished 521 mg (52 %) of *vii* as a clear oil consisting of 1.1:1 ratio of *endo* and *exo*-diastereomers: IR 2948, 2867, 1602 cm^{-1}; HRMS (+EI) *m/z* [M]$^+$ calcd. for $C_9H_{13}I$ 248.0056, found 248.0071. For the major diastereomer: ^{1}H NMR (400 MHz, $CDCl_3$) δ 6.25 – 6.20 (m, 1H), 6.11 –

6.06 (m, 1H), 2.76 – 2.63 (m, 1H), 2.09 – 2.06 (m, 1H), 1.99 – 1.89 (m, 1H), 1.61 – 1.10 (m, 7H), 0.89 (ddd, *J* = 12.4, 5.0, 2.4 Hz, 1H); $^{13}C\{1H\}$ NMR (101 MHz, $CDCl_3$) δ 145.2, 81.5, 45.3, 40.9, 39.9, 37.3, 36.58, 30.0, 23.8. For the minor diastereomer: ^{1}H NMR (400 MHz, $CDCl_3$) δ 6.17 (dd, *J* = 7.3, 0.5 Hz, 1H), 6.04 (d, *J* = 7.3 Hz, 1H), 2.37 – 2.24 (m, 2H), 1.66 (ddd, *J* = 12.4, 8.6, 2.5 Hz, 1H), 1.60 – 1.11 (m, 8H); $^{13}C\{1H\}$ NMR (101 MHz, $CDCl_3$) δ 147.1, 79.5, 47.1, 42.2, 37.8, 36.62, 36.5, 29.4, 28.8.

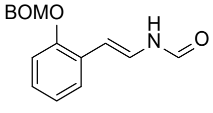

17e

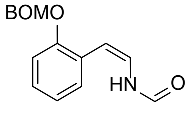

17e'

*(E)-N-(2-((benzyloxy)methoxy)styryl)formamide (**17e** and **17e'**)*. Following the published cross-coupling procedure[9] with pure (*Z*)1-((benzyloxy)methoxy)-2-(2-iodovinyl)benzene (**iv**, 180 mg, 0.492 mmol), formamide (0.39 mL, 9.83 mmol), CuI (9.4 mg, 49 µmol), Cs_2CO_3 (250.2 mg, 0.74 mmol) and *N*,*N'*-dimethylethane-1,2-diamine (16 µL, 99 µmol) and purification by MPLC (12 g cartridge filled with C-2 silica gel[1] eluting with EtOAc/Hexanes 25:75 to 40:60) furnished 114.1 mg (82%) of vinyl formamide **17e** as a 1: 1.5 mixture of *E:Z* geometric isomers. For *E*- *N*-(2-((benzyloxy)methoxy)styryl)formamide (**17e**) obtained as a white solid: mp 120 °C - 120.5 °C; IR (ATR) 3284, 2885, 1670, 1647 cm^{-1}; HRMS (+ESI) m/z [M^+] Calcd. for $C_{17}H_{17}NO_3$, 283.1208 found 283.1212. The ^{1}H NMR spectrum (400 MHz, $CDCl_3$) shows two rotamers in a 1:2.3 ratio. For the major rotamer: ^{1}H NMR (400 MHz, $CDCl_3$) δ 8.19 (s, 1H), 7.62 (dd, *J* = 14.7, 11.1 Hz, 1H), 7.40 (d, *J* = 7.3 Hz, 1H), 7.38 – 7.27 (m, 6H), 7.20 – 7.15 (m, 2H), 7.02 – 6.95 (m, 1H), 6.46 (d, *J* = 14.7 Hz, 1H), 5.34 (s, 2H), 4.74 (s, 2H); $^{13}C\{1H\}$ NMR (101 MHz, $CDCl_3$) δ 158.0, 154.08, 137.2, 128.48, 128.1, 128.01, 127.90, 126.4, 125.1, 123.7, 122.13, 114.75, 109.6, 92.7, 70.26. For the minor rotamer: ^{1}H NMR (400 MHz, $CDCl_3$) δ 8.40 (d, *J* = 11.4 Hz, 1H), 7.54 (br. s, 1H), 7.38 – 7.27 (m, 6H), 7.20 – 7.14 (m, 2H), 7.16 – 7.09 (m, 1H), 7.01 – 6.95 (m, 1H), 6.30 (d, *J* = 14.3

Hz, 1H), 5.36 (s, 2H), 4.73 (s, 2H). $^{13}C\{1H\}$ NMR (101 MHz, $CDCl_3$) δ 162.5, 154.06, 137.1, 128.50, 127.96, 127.93, 126.5, 124.9, 123.7, 122.06, 121.1, 114.37, 109.1, 92.6, 70.24. *cis*-*N*-(2-((benzyloxy)methoxy)styryl)formamide (**17e'**) was isolated as a white solid: mp 52.9-53.9 °C. The ^1H NMR spectrum (400 MHz, $CDCl_3$) shows two rotamers in a 1:2.3 ratio. For the major rotamer: ^1H NMR (400 MHz, $CDCl_3$) δ 8.11 (s, 1H), 8.08 – 7.98 (m, 1H), 7.38 – 7.19 (m, 8H), 7.08 – 6.98 (m, 2H), 5.83 (d, *J* = 9.8 Hz, 1H), 5.33 (s, 2H), 4.74 (s, 2H); $^{13}C\{1H\}$ NMR (101 MHz, $CDCl_3$) δ 158.2, 153.74, 136.91, 130.1, 128.86, 128.54, 128.04, 127.86, 125.2, 122.7, 120.1, 116.3, 107.8, 93.4, 70.7. For the minor rotamer: ^1H NMR (400 MHz, $CDCl_3$) δ 8.36 (d, *J* = 11.2 Hz, 1H), 8.03 – 7.89 (m, 1H), 7.38 – 7.18 (m, 8H), 7.08 – 7.01 (m, 1H), 6.51 (dd, *J* = 11.6, 9.6 Hz, 1H), 5.72 (d, *J* = 9.6 Hz, 1H), 5.34 (s, 2H), 4.75 (s, 2H); $^{13}C\{1H\}$ NMR (101 MHz, $CDCl_3$) δ 162.6, 153.70, 136.94 129.9, 128.88, 128.5, 127.99, 127.96, 124.5, 122.5, 120.1, 115.9, 106.4, 93.0, 70.5.

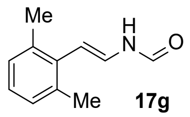

17g

(E)-N-(2,6-Dimethylstyryl)formamide. Following the published coupling procedure[9] with (*E*)-2-(2-iodovinyl)-1,3-dimethylbenzene (**ii**, 1.00 g, 3.87 mmol), formamide (3.09 mL, 77 mmol), CuI (73.8 mg, 0.387 mmol), Cs_2CO_3 (1.89 g, 5.81 mmol) and *N,N'*-dimethylethane-1,2-diamine (122 μL, 0.77 mmol) with purification by MPLC (12 g cartridge filled with C-2 silica gel[1] eluting with EtOAc/Hexanes 25:75 to 40:60), furnished 283 mg (42%) of vinyl formamide **17g** as a white solid: mp = 89-89.6 °C; IR (ATR) 3255, 3040, 2878, 1646 cm^{-1}; HRMS (+EI) *m/z* $[M]^+$ calcd. for $C_{11}H_{13}NO$ 175.0997, found 175.0999. The ^1H NMR spectrum (400 MHz, $CDCl_3$) showed two formamide rotamers in a 2.4:1 ratio. For the major rotamer: ^1H NMR (400 MHz, $CDCl_3$) δ 8.21 (s, 1H), 7.51 (br s, 1H), 7.11 – 6.99 (m, 4H), 6.18 (d, *J* = 14.9 Hz, 1H), 2.32 (s, 6H); $^{13}C\{1H\}$ NMR (101 MHz, $CDCl_3$) δ 158.9, 136.3, 134.11, 128.02, 126.7, 124.4,

112.1, 21.3. For the minor rotamer: ^{1}H NMR (400 MHz, $CDCl_3$) δ 8.36 (d, J = 11.3 Hz, 1H), 7.99 (br s, 1H), 7.11 – 7.00 (m, 3H), 6.50 (dd, J = 14.5, 10.9 Hz, 1H), 6.07 (d, J = 14.5 Hz, 1H), 2.32 (s, 6H); ^{13}C{1H} NMR (101 MHz, $CDCl_3$) δ 163.3, 136.4, 134.06 127.99 126.83, 126.76, 111.0, 21.2.

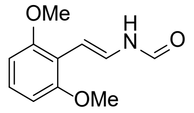
17h

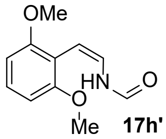
17h'

*(E)-N-(2,6-Dimethoxystyryl)formamide (**17h**)*. Following the published coupling procedure[9] with 2-(2-iodovinyl)-1,3-dimethoxybenzene (**iii**, 415 mg, 1.43 mmol trans vs cis 1.6 :1), formamide (1.14 mL, 29 mmol), CuI (27.2 mg, 0.143 mmol), Cs_2CO_3 (699.1 mg, 2.15 mmol) and *N*,*N'*-dimethylethane-1,2-diamine (45 µL, 0.286 mmol) with purification by MPLC (4 g cartridge filled with C-2 silica gel[1] eluting with EtOAc/Hexanes 25:75 to 40:60), furnished 212 mg (72%) of the vinyl formamide **17h** and **17h'** as a white solid comprised of a 15:1 mixture of *E*-:*Z*-isomers, respectively: mp = 91.2-93.5 °C; IR (ATR) 3294, 2935, 2839, 1659 cm^{-1}; HRMS (+ESI) *m/z* $[M]^+$ calcd. for $C_{11}H_{13}NO_3$ 207.0895, found 207.0899. For (*E*)-N-(2,6-dimethoxystyryl)formamide (**17h**) the ^{1}H NMR spectrum (400 MHz, $CDCl_3$) showed two rotamers in a 1:1.7 ratio. For the major rotamer: ^{1}H NMR (400 MHz, $CDCl_3$) δ 8.17 (s, 1H), 7.99 (dd, J = 14.7, 11.3 Hz, 1H), 7.24 (br. s, 1H), 7.12 (t, J = 8.3 Hz, 1H), 6.57 – 6.54 (m, 2H), 6.51 (d, J = 14.7 Hz, 1H), 3.87 (s, 6H); ^{13}C{1H} NMR (101 MHz, $CDCl_3$) δ 157.79, 157.77, 127.4, 123.8, 112.6, 105.3, 103.7, 55.7. For the minor rotamer: ^{1}H NMR (400 MHz, $CDCl_3$) δ 8.40 (d, J = 11.4 Hz, 1H), 7.60 (dd, J = 14.3, 11.4 Hz, 1H), 7.32 (br. s, 1H), 7.12 (t, J = 8.3 Hz, 1H), 6.59 – 6.55 (m, 2H), 6.40 (d, J = 14.3 Hz, 1H); ^{13}C{1H} NMR (101 MHz, $CDCl_3$) δ 162.5, 157.6, 127.2, 126.1, 112.4, 104.4, 103.9, 55.7. For (*Z*)-N-(2,6-dimethoxystyryl)formamide (**17h'**) obtained as a clear oil, the ^{1}H NMR spectrum (400 MHz, $CDCl_3$) showed two rotamers in a 1:1.4 ratio. For the major rotamer: ^{1}H NMR (400

MHz, $CDCl_3$) δ 8.17 (s, 1H), 7.89 (br s, 1H), 7.28 – 7.23 (m, 1H), 7.01 (t, *J* = 10.4 Hz, 1H), 6.63 (d, *J* = 8.1 Hz, 2H), 5.82 (d, *J* = 10.4 Hz, 1H), 3.87 (s, 6H); $^{13}C\{1H\}$ NMR (101 MHz, $CDCl_3$) δ 157.9, 157.2, 128.96, 120.4, 113.0, 104.8, 103.1, 56.3. For the minor rotamer: ^1H NMR (400 MHz, $CDCl_3$) δ 8.33 (d, *J* = 11.4 Hz, 1H), 7.73 (br s, 1H), 7.29 – 7.20 (m, 1H), 6.61 (d, *J* = 7.8 Hz, 2H), 6.53 (dd, *J* = 11.3, 9.7 Hz, 1H), 5.70 (d, *J* = 9.7 Hz, 1H), 3.87 (s, 6H), $^{13}C\{1H\}$ NMR (101 MHz, $CDCl_3$) δ 162.5, 157.1, 128.96, 122.9, 112.1, 104.3, 101.9, 56.0.

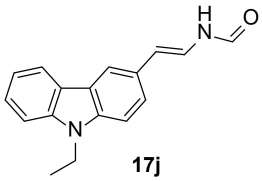
17j

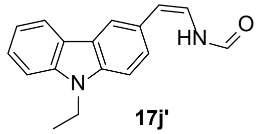
17j'

(E)-N-(2-(9-Ethyl-9H-carbazol-3-yl)vinyl)formamide ***(17j)***. Following the published coupling procedure[9] with (*E*)-9-ethyl-3-(2-iodovinyl)-9H-carbazole (**vii**, 421 mg, 1.21 mmol *trans* : *cis*, 1: 8), formamide (0.77 mL, 19.40 mmol), CuI (23.1 mg, 0.12 mmol), Cs_2CO_3 (592.6 mg, 1.82 mmol) and *N*,*N'*-dimethylethane-1,2-diamine (38 μL, 0.24 mmol) and purification by MPLC (4 g cartridge with C-2 silica gel[1] eluting with EtOAc/Hexanes 25:75 to 40:60), furnished 208.3 mg (65%) of the vinyl formamide **17j** and **17j'** as a yellow solid comprised of 10 :1 mixture of *E*-:*Z*-isomers, respectively: m.p. = 136.8-137.5 °C; IR (ATR) 3284, 2974, 1648, 1597 cm^{-1}; HRMS (+ESI) *m/z* $[M+H]^+$ calcd. for $C_{17}H_{17}N_2O$ 265.1314, found 265.1367. For (*E*)-N-(2-(9-ethyl-9H-carbazol-3-yl)vinyl)formamide (**17j**) the ^1H NMR spectrum (400 MHz, $CDCl_3$) showed two rotamers in a 3.3:1 ratio. For the major rotamer: ^1H NMR (400 MHz, $CDCl_3$) δ 8.19 (s, 1H), 8.06 (d, *J* = 7.9 Hz, 1H), 8.02 (d, *J* = 1.7 Hz, 1H), 7.58 (dd, *J* = 14.5, 10.9 Hz, 1H), 7.51 – 7.43 (m, 2H), 7.43 – 7.34 (m, 2H), 7.32 (d, *J* = 8.4 Hz, 1H), 7.25 – 7.19 (m, 1H), 6.36 (d, *J* = 14.5 Hz, 1H), 4.33 (q, *J* = 7.4 Hz, 2H), 1.41 (t, *J* = 7.4 Hz, 3H); $^{13}C\{1H\}$ NMR (101 MHz, $CDCl_3$) δ 158.0, 140.25, 139.3, 126.3, 125.8, 123.6, 123.25, 122.72, 120.46, 119.0, 118.2, 117.8, 115.6, 108.7, 108.6, 37.6, 13.8. For the

minor rotamer: ^{1}H NMR (400 MHz, $CDCl_3$) δ 8.46 (d, *J* = 11.4 Hz, 1H), 8.11 – 8.06 (m, 1H), 7.99 (d, *J* = 1.7 Hz, 1H), 7.68 (s, 1H), 7.50 – 7.44 (m, 2H), 7.43 – 7.34 (m, 1H), 7.32 (d, *J* = 8.4 Hz, 1H), 7.25 – 7.18 (m, 1H), 7.05 (dd, *J* = 14.2, 10.8 Hz, 1H), 6.30 (d, *J* = 14.2 Hz, 1H), 4.40 – 4.30 (m, 2H), 1.47 – 1.38 (m, 3H); ^{13}C{1H} NMR (101 MHz, $CDCl_3$) δ 162.4, 140.28, 139.2, 126.2, 125.9, 123.4, 123.3, 122.7, 120.4, 119.0, 118.2, 117.5, 114.7, 108.8, 108.64, 37.6, 13.8. For (*Z*)-*N*-(2-(9-ethyl-9H-carbazol-3-yl)vinyl)formamide (**17j'**) the ^{1}H NMR spectrum (400 MHz, $CDCl_3$) showed two rotamers in a 2.45:1 ratio. For the major rotamer: ^{1}H NMR (400 MHz, $CDCl_3$) δ 8.19 (s, 1H), 8.09 (d, *J* = 7.7 Hz, 1H), 8.00 (s, 1H), 7.85 (br s, 1H), 7.50 – 7.46 (m, 1H), 7.45 – 7.38 (m, 2H), 7.36 (dd, *J* = 8.4, 1.7 Hz, 1H), 7.28 – 7.22 (m, 1H), 7.03 (dd, *J* = 11.3, 9.4 Hz, 1H), 6.06 (d, *J* = 9.4 Hz, 1H), 4.37 (q, *J* = 7.2 Hz, 2H), 1.44 (t, *J* = 7.2 Hz, 3H); ^{13}C{1H} NMR (101 MHz, $CDCl_3$) δ 158.4, 140.29, 138.9, 126.15, 125.9, 125.6, 123.40, 122.5, 120.5, 119.8, 119.15, 118.7, 112.9, 109.0, 108.72, 37.6, 13.8. For the minor rotamer: ^{1}H NMR (400 MHz, $CDCl_3$) δ 8.42 (d, *J* = 11.3 Hz, 1H), 8.10 (d, *J* = 7.7 Hz, 1H), 7.96 (s, 1H), 7.85 (br s, 1H), 7.52 – 7.48 (m, 1H), 7.45 – 7.38 (m, 2H), 7.36 – 7.32 (m, 1H), 7.28 – 7.22 (m, 1H), 6.50 (dd, *J* = 11.7, 9.3 Hz, 1H), 5.93 (d, *J* = 9.3 Hz, 1H), 4.37 (q, *J* = 7.2 Hz, 2H), 1.44 (t, *J* = 7.2 Hz, 3H); ^{13}C{1H} NMR (101 MHz, $CDCl_3$) δ 162.8, 140.30, 138.9, 126.12, 125.9, 125.2, 123.44, 120.9, 120.6, 119.6, 119.12, 118.7, 111.5, 109.0, 108.67, 37.6, 13.8.

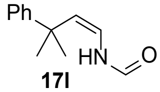

17l

*(Z)-N-(3-Methyl-3-phenylbut-1-en-1-yl)formamide (**17l**).* Following the published cross-coupling procedure[9] with pure (*Z*)-(4-iodo-2-methylbut-3-en-2-yl)benzene (50 mg, 0.18 mmol), formamide (0.12 mL, 2.94 mmol), CuI (3.5 mg, 18 µmol), Cs_2CO_3 (89.8 mg, 0.27 mmol) and *N*,*N'*-dimethylethane-1,2-diamine (5.8 µL, 36.8 µmol) and purification by MPLC (12 g cartridge filled with C-2 silica gel[1] eluting with EtOAc/Hexanes 25:75 to 40:60) furnished

28.5 mg (82%) of vinyl formamide as a clear oil: IR (ATR) 3381, 2966, 2871, 1655 cm^{-1}; HRMS (+ESI) m/z $[M+H]^+$ Calcd. for $C_{12}H_{15}NO$, 190.1232, found 190.1243. The ^1H NMR spectrum (400 MHz, $CDCl_3$) shows two rotamers in a 1:2.6 ratio. For the major rotamer: ^1H NMR (400 MHz, $CDCl_3$) δ 7.66 (s, 1H), 7.52 – 7.32 (m, 5H), 6.67 – 6.56 (m, 1H), 6.15 (br. s, 1H), 5.06 (d, *J* = 9.5 Hz,1H), 1.45 (s, 6H); $^{13}C\{1H\}$ NMR (101 MHz, $CDCl_3$) δ 157.4, 147.8, 129.0, 126.7, 126.3, 122.3, 117.7, 39.2, 30.5. For the minor rotamer: ^1H NMR (400 MHz, $CDCl_3$) δ 7.99 (d, *J* = 10.8 Hz, 1H), 7.43 – 7.33 (m, 2H), 7.32 – 7.25 (m, 3H), 6.25 – 6.03 (m, 2H), 4.94 (d, *J* = 8.8 Hz, 1H), 1.46 (s, 6H); $^{13}C\{1H\}$ NMR (101 MHz, $CDCl_3$) δ 162.0, 147.3, 129.1, 126.9, 126.1, 120.6, 120.4, 39.1, 30.6.

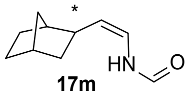
17m

*N-((Z)-2-((1S,4R)-Bicyclo[2.2.1]heptan-2-yl)vinyl)formamide (**17m**)* Following the published cross-coupling procedure[9] with (1*S*,4*R*)-2-((*Z*)-2-iodovinyl)bicyclo[2.2.1]heptane (54 mg, 0.22 mmol), formamide (0.14 mL, 3.48 mmol), CuI (4.2 mg, 22 µmol), Cs_2CO_3 (106.4 mg, 0.33 mmol) and *N,N'*-dimethylethane-1,2-diamine (6.9 µL, 44 µmol) and purification by MPLC (4 g cartridge with C-2 silica gel[1] eluting with EtOAc/Hexanes 25:75 to 40:60) furnished 18.1 mg (50%) of **17m** as a clear oil consisting of a 1.1:1 ratio of *endo*- and *exo*-diastereomers: IR (ATR) 3283, 2947, 2868, 1655 cm^{-1}; HRMS (+ESI) m/z $[M+H]^+$ Calcd. for $C_{10}H_{16}NO$, 166.1232, found 166.1234. For the major isomer, the ^1H NMR spectrum (400 MHz, $CDCl_3$) showed two rotamers in a 1.62:1 ratio. For the major rotamer ^1H NMR (400 MHz, $CDCl_3$) δ 8.15 (s, 1H), 7.18 (br s, 1H), 6.74 (ddd, *J* = 11.0, 9.0, 1.5 Hz, 1H), 4.89 (t, *J* = 9.0 Hz, 1H), 2.62 – 2.50 (m, 1H), 2.25 – 2.17 (m, 1H), 2.04 – 1.98 (m, 1H), 1.96 – 1.86 (m, 2H), 1.57 – 1.47 (m, 1H), 1.41 – 1.28 (m, 2H), 1.26 – 1.12 (m, 2H), 0.90 – 0.83 (m, 1H); $^{13}C\{1H\}$ NMR (101 MHz, $CDCl_3$) δ 158.4, 120.2, 118.8, 41.53, 40.04, 37.8, 36.7, 36.30, 29.94, 23.2. For the minor rotamer:

^{1}H NMR (400 MHz, $CDCl_3$) δ 8.26 (d, *J* = 11.4 Hz, 1H), 7.18 (br s, 1H), 6.26 (ddd, *J* = 10.8, 8.8, 1.5 Hz, 1H), 4.81 – 4.71 (m, 1H), 2.62 – 2.50 (m, 1H), 2.25 – 2.17 (m, 1H), 2.04 – 1.98 (m, 1H), 1.96 – 1.86 (m, 2H), 1.57 – 1.47 (m, 2H), 1.41 – 1.28 (m, 1H), 1.26 – 1.12 (m, 2H), 0.86 – 0.80 (m, 1H); ^{13}C{1H} NMR (101 MHz, $CDCl_3$) δ 163.1, 121.1, 116.6, 41.48, 39.98, 37.8, 36.5, 36.34, 29.99, 23.1. The ^{1}H NMR spectrum (400 MHz, $CDCl_3$) of the minor diastereomer showed two rotamers in a 1:1.57 ratio. For the major rotamer: ^{1}H NMR (400 MHz, $CDCl_3$) δ 8.15 (s,1H), 7.34 (br s, 1H), 6.61 (ddd, *J* = 11.1, 8.9, 1.3 Hz, 1H), 4.76 (t, *J* = 8.9 Hz, 1H), 2.33 – 2.08 (m, 2H), 1.67 – 1.45 (m, 3H), 1.44 – 1.29 (m, 3H), 1.29 – 1.09 (m, 3H); ^{13}C{1H} NMR (101 MHz, $CDCl_3$) δ 158.43, 119.1, 117.1, 42.8, 39.18, 38.6, 37.17, 35.9, 29.6, 28.82; For the minor rotamer ^{1}H NMR (400 MHz, $CDCl_3$) δ 8.25 (d, *J* = 11.4 Hz, 1H), 7.34 (br s, 1H), 6.12 (ddd, *J* = 10.9, 8.8, 1.4 Hz, 1H), 4.63 (t, *J* = 8.8 Hz, 1H), 2.33 – 2.08 (m, 2H), 1.67 – 1.45 (m, 3H), 1.44 – 1.29 (m, 3H), 1.29 – 1.09 (m, 3H); ^{13}C{1H} NMR (101 MHz, $CDCl_3$) δ 163.0, 119.0, 115.9, 42.9, 39.20, 38.4, 37.15, 36.0, 29.4, 28.76.

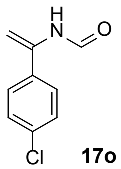

17o

*N-(1-(4-Chlorophenyl)vinyl)formamide (**17o**):* Prepared following the published procedure [10] with 4-chlorobenzonitrile (1.00 g, 7.30 mmol), CuCN (13.0 mg, 0.145 mmol), and MeLi.LiBr (5.3 mL, 8.7 mmol). After 40 min at 0 °C, isopropyl formate (3.64 mL, 36.4 mmol) was added, then the mixture was heated to 50 °C for 160 h to afford, after purification (dry loaded with Celite onto a 12 g silica gel cartridge, using 0-40% EtOAc/hexanes gradient eluent), 602 mg (46%) of vinylformamide **17o** as an orange solid: mp = 112.6-113.5 °C; IR (ATR): 3195, 1691, 1623 cm^{-1}; HRMS (EI+) m/z $[M+H]^+$ calcd. for C_9H_9ClNO 182.0373, found 182.0374. The ^{1}H NMR spectrum (400 MHz, $CDCl_3$) shows two rotamers in a 3.9:1.0 ratio. For the major rotamer: ^{1}H NMR (400 MHz, $CDCl_3$) δ 8.43 (d, *J* = 10.9 Hz, 1H), 8.20 (br d, *J* =

10.9 Hz, 1H), 7.39 (ABq, Δν = 24.0 Hz, J = 8.8 Hz, 4H), 4.97-4.94 (m, 2H); ^{13}C NMR (101 MHz, $CDCl_3$) δ 163.5, 140.5, 135.5, 134.4, 129.02, 127.8, 100.7. For the minor rotamer: ^{1}H NMR (400 MHz, $CDCl_3$) δ 8.40 (s, 1H), 7.45-7.32 (m, 4H), 7.18 (br s, 1H), 5.89 (s, 1H), 5.15 (s, 1H); ^{13}C NMR (101 MHz, $CDCl_3$) δ 159.9, 138.5, 136.1, 134.8, 128.91, 127.3, 104.5.

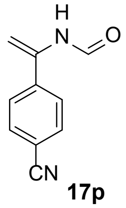
17p

*N-(1-(4-cyanophenyl)vinyl)formamide (**17p**):* Prepared following the published procedure [10] with 4-cyanobenzonitrile (1.00 g, 7.80 mmol), CuCN (14.0 mg, 0.156 mmol), and MeLi.LiBr (6.50 mL, 7.80 mmol). After 40 min at 0 °C, isopropyl formate (3.91 mL, 39.0 mmol) was added, then the mixture was heated to 50 °C for 72 h to afford, after purification (dry loaded with Celite onto a 12 g silica gel cartridge, using 0-100% CH_2Cl_2/hexanes gradient eluent), 175 mg (15%) of vinylformamide **17p** as a yellow solid: mp = 135.7-137.0 °C; IR (ATR): 3207, 3095, 2981, 2229, 1695, 1626 cm^{-1}; HRMS (EI+) m/z [M+H]$^{+}$ calcd. for $C_{10}H_9N_2O$ 173.0715, found 173.0710. The ^{1}H NMR spectrum (400 MHz, $CDCl_3$) shows two rotamers in a 2.5:1.0 ratio. For the major rotamer: ^{1}H NMR (400 MHz, $CDCl_3$) δ 8.46 (d, J = 10.9 Hz, 1H), 7.92 (br s, 1H), 7.69 (d, J = 8.4 Hz, 2H), 7.61 (d, J = 8.4 Hz, 2H), 5.13 (s, 1H), 5.11 (s, 1H); ^{13}C NMR (101 MHz, $CDCl_3$) δ 163.1, 140.3, 139.9, 132.7, 127.0, 118.2, 113.2, 103.3. For the minor rotamer: ^{1}H NMR (400 MHz, $CDCl_3$) δ 8.43 (s, 1H), 7.66 (d, J = 8.3 Hz, 2H), 7.54 (d, J = 8.3 Hz, 2H), 7.05 (br s, 1H), 5.94 (s, 1H), 5.29 (s, 1H); ^{13}C NMR (101 MHz, $CDCl_3$) δ 159.6, 141.7, 138.1, 132.6, 126.6, 118.4, 112.5, 107.1.

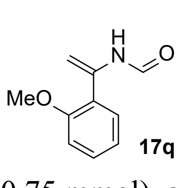
17q

*N-(1-(2-Methoxyphenyl)vinyl)formamide (**17q**):* Prepared following the published procedure [10] with 2-methoxybenzonitrile (500 mg, 3.76 mmol), CuCN (7.0 mg, 0.75 mmol), and MeLi.LiBr (3.39 mL, 4.51 mmol). After 40 min at 0 °C, isopropyl formate (1.88

mL, 18.8 mmol) was added, then the mixture was heated to 50 °C for 48 h to afford, after purification (dry loaded with Celite onto a 12 g silica gel cartridge, using 0-40% EtOAc/hexanes gradient eluent), 510 mg (77%) of vinylformamide **18q** as a yellow solid: mp = 87.7-89.0 °C; IR (ATR): 3240, 2981, 1681, 1636 cm^{-1}; HRMS (EI+) m/z $[M+H]^+$ calcd. for $C_{10}H_{12}NO_2$ 178.0868, found 178.0850. The ^1H NMR spectrum (400 MHz, $CDCl_3$) shows two rotamers in a 2.6:1.0 ratio. For the major rotamer: ^1H NMR (400 MHz, $CDCl_3$) δ 8.25 (d, J = 11.3 Hz, 1H), 7.94 (br s, 1H), 7.39-7.34 (m, 1H), 7.32 (d, J = 7.6 Hz, 1H), 7.00-6.96 (m, 1H), 6.91 (d, J = 7.6 Hz, 1H), 4.92 (s, 1H), 4.66 (s, 1H), 3.85 (s, 3H); ^{13}C NMR (101 MHz, $CDCl_3$) δ 162.9, 156.4, 140.5, 130.7, 130.41, 124.5, 121.0, 110.9, 100.4, 55.5. For the minor rotamer: ^1H NMR (400 MHz, $CDCl_3$) δ 8.33 (s, 1H), 7.56 (br s, 1H), 7.39-7.34 (m, 1H), 7.32 (d, J = 7.6 Hz, 1H), 7.00-6.96 (t, J = 7.6 Hz, 1H), 6.91 (d, J = 7.6 Hz, 1H), 6.11 (s, 1H), 5.05 (s, 1H) 3.87 (s, 3H); ^{13}C NMR (101 MHz, $CDCl_3$) δ 159.3, 156.0, 138.6, 130.43, 130.1, 126.6, 121.3, 111.1, 105.1, 55.7.

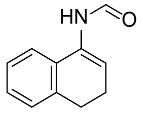

17r

N-(3,4-Dihydronaphthalen-1-yl)formamide ***(17r)****.*[11] Solid Cs_2CO_3 (143 mg, 0.44 mmol) was flame dried under vacuum, allowed to cool to rt, and then solid CuI (5.9 mg, 0.03 mmol), and neat formamide (0.19 mL, 4.69 mmol) were added sequentially. The reaction flask was placed under vacuum and backfilled with nitrogen three times. Dioxane (0.1 M), *N,N'*-dimethylethane-1,2-diamine (11 μL, 0.2 mmol,), and 4-iodo-1,2-dihydronaphthalene[12] (75 mg, 0.29 mmol,) were added and then the mixture was heated to reflux. After 4 h, the reaction mixture was allowed to cool to rt and then transferred to a separatory funnel. The precipitate remaining in the reaction flask was sequentially rinsed with EtOAc (30 mL) and saturated, aqueous NH_4Cl solution (6 mL) and the phases were transferred to the separatory funnel. The phases were separated, the organic phase was dried with Na_2SO_4, concentrated, and then the

crude product was purified by flash chromatography (hexanes: ethyl acetate, 60:40) to afford 39.5 mg (78%) of **17r** as a clear, colorless oil: IR (ATR) 3258, 2935, 2831, 1681 cm^{-1}; HRMS (+ESI) m/z [M+H] Calcd. for $C_{11}H_{11}NO$ 173.0841; found 173.0842. The ^{1}H NMR spectrum (400 MHz, $CDCl_3$) showed the presence of two rotamers in a 1:3.3 ratio. For the major rotamer: δ 8.38 (d, *J* = 11.1 Hz, 1H), 7.54 (br d, *J* = 11.1 Hz, 1H), 7.31 – 7.12 (m, 4H), 5.87 (t, *J* = 4.8 Hz, 1H), 2.86 – 2.77 (m, 2H), 2.44 – 2.33 (m, 2H). $^{13}C\{1H\}$ NMR (101 MHz, $CDCl_3$) δ 163.5, 136.6, 132.2, 131.2, 128.4, 127.9, 126.8, 121.5, 118.0, 27.5, 22.1. For the minor rotamer: δ 8.45 (d, *J* = 1.8 Hz, 1H), 7.30 – 7.12 (m, 4H), 6.95 (br s, 1H), 6.59 (t, *J* = 4.9 Hz, 1H), 2.79 – 2.73 (m, 2H), 2.43 – 2.33 (m, 2H); $^{13}C\{1H\}$ NMR (101 MHz, $CDCl_3$) δ 159.9, 136.9, 130.9, 130.3, 128.0, 127.8, 126.5, 120.3, 119.6, 27.6, 22.1.

General Bromovinylformamide Synthesis: Neat triethylamine (3 mmol, 3 equiv.) and neat bromine (1.05 mmol,1.05 equiv) was added sequentially to a 0 °C, CH_2Cl_2 solution (0.1M) of the vinylformamide (1 mmol, 1 equiv). The cooling bath was removed and then the reaction was monitored by TLC until complete. The reaction mixture was then poured into a 0 °C solution of saturated, aqueous sodium carbonate, the phases were separated, and then the aqueous phase was extracted with CH_2Cl_2 (3 x 15 mL). The combined organic extract was washed sequentially with water and brine, and then dried (Na_2SO_4), filtered, and concentrated. The crude product was purified by flash chromatography on C-2 silica[1] to afford pure bromovinylformamide.

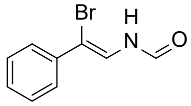

18a

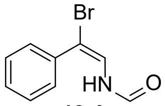

18a'

(Z)-N-(2-Bromo-2-phenylvinyl)formamide ***(18a).*** Following the general bromoalkeneisocyanide synthesis method with *(E)-N*-styrylformamide[9] (229 mg, 1.56 mmol), bromine (87 µL 1.63 mmol), triethylamine (0.9 mL,

6.22 mmol), and purification on silica using 20% EtOAc/hexanes as the eluant, furnished 241.5 mg (70%) of a clear oil consisting of a 7: 1 *Z*/*E* mixture of **18a'/18a** as a clear oil: IR (ATR) 3287, 3065, 1677, 1643 cm^{-1}; HRMS (+APCI) m/z [M+H$^+$] Calcd. for C_9H_9BrNO 225.9868, found 225.9862. For the major isomer **18a**, the ^{1}H NMR spectrum (400 MHz, $CDCl_3$) showed two rotamers in a 5.3:1 ratio. For the major rotamer: ^{1}H NMR (400 MHz, $CDCl_3$) δ 8.32 (s, 1H), 7.68 (br s, 1H), 7.58 – 7.50 (m, 2H), 7.40 – 7.27 (m, 4H); ^{13}C NMR (101 MHz, $CDCl_3$) δ 157.9, 136.49, 128.6, 128.5, 127.0, 119.5, 109.4. For the minor rotamer: ^{1}H NMR (400 MHz, $CDCl_3$) δ 8.54 (d, *J* = 11.4 Hz, 1H), 7.68 (s, 1H), 7.50 – 7.46 (m, 2H), 7.39 – 7.27 (m, 3H), 7.14 (d, *J* = 11.4 Hz, 1H); ^{13}C NMR (101 MHz, $CDCl_3$) δ 161.5, 136.51, 128.7, 128.5, 126.9, 121.9, 106.5. For the minor isomer **18a'**, the ^{1}H NMR spectrum (400 MHz, $CDCl_3$) shows two rotamers in a 1:2.8 ratio. For the major rotamer: ^{1}H NMR (400 MHz, $CDCl_3$) δ 8.03 (s, 1H), 7.49 (d, *J* = 11.3 Hz, 1H), 7.47 – 7.33 (m, 5H), 7.21 (br s, 1H); ^{13}C NMR (101 MHz, $CDCl_3$) δ 157.0, 136.2, 129.30, 129.27, 128.7, 121.4, 106.1. For the minor isomer, ^{1}H NMR (400 MHz, $CDCl_3$) δ 8.32 (d, *J* = 11.3 Hz, 1H), 7.47 – 7.31 (m, 5H), 7.21 (br s, 1H), 6.98 (d, *J* = 11.3 Hz, 1H); ^{13}C NMR (101 MHz, $CDCl_3$) δ 161.1, 135.6, 129.4, 129.31, 128.6, 123.5,103.1.

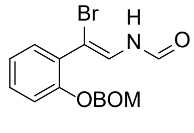

18e

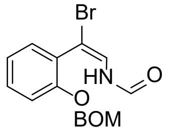

18e'

(Z)- and (E)-N-(2-(2-((Benzyloxy)methoxy)phenyl)-2-bromovinyl)formamide (***18e** and **18e'***). Following the general bromoalkeneisocyanide synthesis with *(E)-N*-(2-((benzyloxy)methoxy)styryl)formamide (**17e**, 50 mg, 0.18 mmol), bromine (14 µL 0.27 mmol), triethylamine (74 µL, 0.53 mmol), and purification on silica using 20% EtOAc/hexanes as the eluant, furnished 36 mg (56%) of **18e** as a clear oil consisting of 2.5: 1 mixture of *Z*/*E* isomers **18e/18e'**, respectively: IR (ATR) 3300, 3065, 2907,1657 cm^{-1}; HRMS (+EI) m/z [M$^+$] Calcd. for

$C_{17}H_{17}BrNO_3$ 362.0392, found 362.0387. For the major isomer **18e**, the ^{1}H NMR spectrum (400 MHz, $CDCl_3$) showed two rotamers in a 3.7:1 ratio. For the major rotamer: ^{1}H NMR (400 MHz, $CDCl_3$) δ 8.31 (s, 1H), 7.69 (br s, 1H), 7.59 (d, J = 11.1 Hz, 1H), 7.38 (dd, J = 7.7, 1.7 Hz, 1H), 7.36 – 7.27 (m, 6H), 7.21 (d, J = 8.4 Hz, 1H), 7.05 – 6.98 (m, 1H), 5.35 (s, 2H), 4.75 (s, 2H); ^{13}C NMR (101 MHz, $CDCl_3$) δ 157.9, 154.7, 136.98, 131.4, 130.20, 128.47, 128.2, 127.9, 126.6, 122.4, 121.7, 114.7, 104.4, 92.2, 70.3. For the minor rotamer: ^{1}H NMR (400 MHz, $CDCl_3$) δ 8.44 (d, J = 11.2 Hz, 1H), 7.71 (br s, 1H), 7.42 – 7.37 (m, 1H), 7.36 – 7.27 (m, 7H), 7.21 (d, J = 8.4 Hz, 1H), 7.05 – 6.97 (m, 1H), 5.36 (s, 2H), 4.75 (s, 2H); ^{13}C NMR (101 MHz, $CDCl_3$) δ 161.6, 154.6, 137.00, 131.46, 130.18, 128.50, 128.2, 128.0, 126.5, 124.7, 121.8, 114.9, 101.4, 92.5, 70.6. For the minor isomer **18e'** the ^{1}H NMR spectrum (400 MHz, $CDCl_3$) showed two rotamers in a 2.6:1 ratio. For the major rotamer: ^{1}H NMR (400 MHz, $CDCl_3$) 7.90 (s, 1H), 7.49 (d, J = 11.0 Hz, 1H), 7.42 – 7.27 (m, 7H), 7.30 – 7.21 (m, 1H), 7.11 (d, J = 7.9 Hz, 1H), 7.04 – 6.93 (m, 1H), 5.36 (s, 2H), 4.75 (s, 2H); ^{13}C NMR (101 MHz, $CDCl_3$) δ 157.0, 153.6, 136.9, 131.23, 131.1, 128.54, 128.03, 127.82, 125.6, 122.74, 122.6, 116.0, 102.7, 92.9, 70.7. For the minor rotamer: ^{1}H NMR (400 MHz, $CDCl_3$) δ 8.30 (d, J = 10.4 Hz, 1H), 7.40 – 7.29 (m, 8H), 7.29 – 7.22 (m, 1H), 7.07 (d, J = 8.6 Hz, 1H), 7.04 – 6.97 (m, 1H), 5.35 (s, 2H), 4.75 (s, 2H); ^{13}C NMR (101 MHz, $CDCl_3$) δ 161.1, 149.7, 136.8, 131.3, 131.16, 128.49, 128.04, 128.00, 124.9, 122.8, 122.71, 115.6, 99.7, 92.5, 70.5.

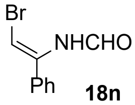

18n

(Z)-N-(2-bromo-1-phenylvinyl)formamide ***(18n)***. Following the general bromoalkeneisocyanide synthesis with *N*-(1-phenylvinyl)formamide[9] (50 mg, 0.34 mmol), bromine (18 μL 0.36 mmol), triethylamine (0.14 mL, 1.02 mmol), and purification on silica using 20% EtOAc/hexanes as the eluant, furnished 69.9 mg (91%) of **18n** as a yellow oil:

IR (ATR) 3241, 3100, 2895, 1686 cm^{-1}; HRMS (+EI) m/z [M$^+$] Calcd. for C_9H_8BrNO 224.9789, found 224.9764. The ^{1}H NMR spectrum (400 MHz, $CDCl_3$) showed two rotamers in a 12:1 ratio. For the major rotamer: ^{1}H NMR (400 MHz, $CDCl_3$) δ ^{1}H NMR (400 MHz, $CDCl_3$) δ 8.26 (d, *J* = 11.1 Hz, 1H), 7.75 (br s, 1H), 7.48 – 7.38 (m, 5H), 5.61 (s, 1H); ^{13}C NMR (101 MHz, $CDCl_3$) δ 162.5, 139.4, 133.6, 130.0, 129.2, 128.1, 89.3. For the minor rotamer: ^{1}H NMR (400 MHz, $CDCl_3$) δ 8.37 (s, 1H), 7.68 (br s, 1H), 7.45 – 7.33 (m, 5H), 6.09 (s, 1H).

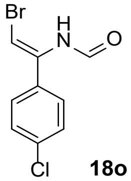

18o

(Z)-N-(2-Bromo-1-(4-chlorophenyl)vinyl)formamide ***(18o)***. Following the general bromoalkeneisocyanide synthesis with *N*-(1-(4-chlorophenyl)vinyl)formamide (**17o**, 20 mg, 0.11 mmol), bromine (6 μL, 0.12 mmol), triethylamine (46 μL, 0.33 mmol), and purification on silica using 20% EtOAc/hexanes as the eluant, furnished 16.5 mg (58%) of **18o** as a white solid: mp 92.7 – 95 °C, IR (ATR) 3252, 3097, 2893, 1689 cm^{-1}. The ^{1}H NMR spectrum (400 MHz, $CDCl_3$) shows two rotamers in a 1:6.5 ratio. For the major rotamer: ^{1}H NMR (400 MHz, $CDCl_3$) δ 8.25 (d, *J* = 11.1 Hz, 1H), 7.61 (br s, 1H), 7.41 (d, *J* = 8.6 Hz, 2H), 7.35 (d, *J* = 8.6 Hz, 2H), 5.63 (s, 1H); ^{13}C NMR (101 MHz, $CDCl_3$) δ 162.1,138.3, 136.2, 132.1, 129.5, 129.3, 90.1; For the minor rotamer: ^{1}H NMR (400 MHz, $CDCl_3$) δ 8.37 (br. s, 1H), 7.34 – 7.27 (m, 4H), 7.19 (s, 1H), 6.10 – 6.03 (s, 1H); ^{13}C NMR (101 MHz, $CDCl_3$) δ 158.4, 138.3, 136.2, 132.1, 128.8, 127.5, 97.2; HRMS (+ESI) m/z [M+H$^+$] Calcd. for $C_9H_8BrClNO$ 259.9501, found 259.9478.

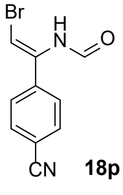

18p

(Z)-N-(2-Bromo-1-(4-cyanophenyl)vinyl)formamide ***(18p)***. Following the general bromoalkeneisocyanide synthesis with *N*-(1-(4-cyanophenyl)vinyl)formamide (**17p**, 30 mg, 0.18 mmol), bromine (18 μL 0.35 mmol), triethylamine (73 μL, 0.53 mmol),

and purification on silica with 80% CH_2Cl_2/hexanes as the eluant, furnished 21 mg (48%) of **18p** as a white solid: mp 154.6 – 156.3 °C, IR (ATR) 3251, 3074, 2227, 1668 cm^{-1}. The ^{1}H NMR spectrum (400 MHz, $CDCl_3$) shows two rotamers in a 2:1 ratio. For the major rotamer: ^{1}H NMR (400 MHz, $CDCl_3$) δ 8.22 (d, *J* = 11.1 Hz, 1H), 7.74 (d, *J* = 8.4 Hz, 1H), 7.68 – 7.58 (m, 1H), 7.55 (d, *J* = 8.4 Hz, 2H), 5.81 (s, 1H); ^{13}C NMR (101 MHz, $CDCl_3$) δ 161.7, 138.2, 137.9, 133.0, 128.5, 117.8, 113.9, 93.0. For the minor rotamer: ^{1}H NMR (400 MHz, $CDCl_3$) δ 8.31 (s,1H), 7.65 (d, *J* = 8.1 Hz, 2H), 7.46 (d, *J* = 8.1 Hz, 2H), 7.30 (br s, 1H), 6.17 (s, 1H); ^{13}C NMR (101 MHz, $CDCl_3$) δ 158.5, 139.1, 138.0, 132.3, 126.9, 118.4, 112.6, 99.4; HRMS (+ESI) m/z [M+H$^+$] Calcd. for $C_{10}H_{11}BrClNO_2$ 250.9820, found 250.9848

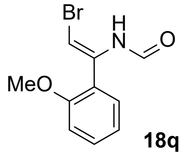

18q

*(Z)-N-(2-Bromo-1-(2-methoxyphenyl)vinyl)formamide (**18q**)*. Following the general bromoalkeneisocyanide synthesis with *N*-(1-(2-methoxyphenyl)vinyl)formamide (**17q**, 138 mg, 0.78 mmol), bromine (60 µL 1.17 mmol), triethylamine (0.32 mL, 2.34 mmol), and purification on silica using 60% CH_2Cl_2/hexanes as the eluant, furnished 142.6 mg (72%) of **18q** as a white solid: mp 115.2 – 116.9 °C; IR (ATR) 3209, 3101, 2901, 1686 cm^{-1}; ^{1}H NMR (400 MHz, $CDCl_3$) δ 8.02 (d, *J* = 11.2 Hz, 1H), 7.72 (br. d, *J* = 10.9 Hz, 1H), 7.43 (ddd, *J* = 8.4, 7.5, 1.8 Hz, 1H), 7.26 (dd, *J* = 7.5, 1.8 Hz, 1H), 7.03 – 6.96 (m, 1H), 6.92 (d, *J* = 8.4 Hz, 1H), 5.42 (s, 1H), 3.83 (s, 3H); ^{13}C NMR (101 MHz, $CDCl_3$) δ 162.4, 156.5, 137.1, 131.8, 131.3, 121.3, 121.1, 110.8, 88.1, 55.6; HRMS (+ESI) m/z [M+H$^+$] Calcd. for $C_{10}H_{11}BrClNO_2$ 255.9973, found 255.9986.

General Bromoalkeneisocyanide Synthesis A: Neat bromine (1.05 mmol, 1.05 equiv.) was added to a -10 °C, CH_2Cl_2 solution (0.1M) of the vinylformamide (1 mmol, 1 equiv). After 1 h,

triethylamine (3 mmol, 3 equiv) was added and then the mixture was allowed to warm to rt. After 24 h, the reaction was cooled to 0 °C, neat *i*-PrNEt$_2$ (9 mmol, 9 equiv) was then added followed by the dropwise addition of neat phosphoryl chloride (3 mmol, 3.0 equiv). Upon completion, as monitored by TLC, the reaction mixture was poured into 0 °C, saturated, aqueous sodium carbonate, the phases were separated, and then the aqueous phase was extracted with CH_2Cl_2 (3 x 15 mL). The combined organic extract was washed sequentially with water, then brine, and then dried (Na_2SO_4), filtered, and concentrated. The crude product was purified by flash chromatography on C-2 silica[1] to afford pure bromoisocyanoalkene.

General Bromoisocyanoalkene Synthesis B: Dry *i*-Pr$_2$NEt (9 mmol, 9 equiv.) and $POCl_3$ (3 mmol, 3 equiv.) were sequentially added to a -20 °C, CH_2Cl_2 solution (10 mL, 0.1 M) of the bromovinylformamide (1 mmol, 1 equiv.). After the bromovinylformamide was consumed, the reaction mixture was poured into a 0 °C, saturated, aqueous solution of Na_2CO_3, the phases were separated, and the aqueous phase was extracted with CH_2Cl_2 (10 mL x 3). The combined organic phase was washed with water and then with brine, dried (Na_2SO_4), and then concentrated. The crude isocyanide was then purified on a C2-silica[1] gel column to afford the bromoisocyanoalkene.

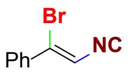

10a

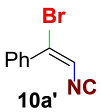

10a'

*Z- and E-Bromo-2-isocyanovinyl benzene (**10a** and **10a'**)*. Following the general bromoisocyanoalkene synthesis A with (*E*)-*N*-styrylformamide[9] (100 mg, 0.679 mmol), bromine (36 µL 0.71 mmol), triethylamine (280 µL, 2.04 mmol), $POCl_3$ (190 µL, 2.04 mmol), *i*-Pr$_2$NEt (1.07 mL, 6.12 mmol) and purification on C-2 silica[1] (1 cm x 2 cm column) using hexanes as the eluant, furnished 119 mg (84%) of (*Z*)-(1-bromo-2-isocyanovinyl)benzene (**10a**) as a yellow oil comprised of a 7:1 ratio of *E- and Z-* geometric

isomers, **10a** and **10a'**, respectively: IR (ATR) 3061, 2928, 2123, 2081 cm^{-1}; HRMS (+EI) m/z [M^+] Calcd. for C_9H_6BrN 206.9684, found 206.9693. For (*Z*)-(1-bromo-2-isocyanovinyl)benzene (**10a**): ^{1}H NMR (400 MHz, $CDCl_3$) δ 7.55 – 7.51 (m, 2H), 7.46 – 7.37 (m, 3H), 6.56 (s, 1H); ^{13}C NMR (101 MHz, $CDCl_3$) δ 170.1, 135.0, 132.1, 130.8, 128.8, 127.7, 112.2 (t, *J* = 14.5 Hz); For *(E)-(1-Bromo-2-isocyanovinyl)benzene (**10a'**):* ^{1}H NMR (400 MHz, $CDCl_3$) δ 7.64 – 7.61 (m, 2H), 7.46 – 7.36 (m, 3H), 6.41 (s, 1H); ^{13}C NMR (101 MHz, $CDCl_3$) δ 168.2, 134.2, 132.3, 130.6, 128.9, 128.6, 111.7, (t, *J* = 11.7 Hz). **From 18a:** Following the general bromoisocyanoalkene synthesis procedure B with *(Z)-N*-(2-bromo-2-phenylvinyl)formamide **(18a)** (270 mg, 1.2 mmol), $POCl_3$ (0.22 mL, 2.39 mmol.), and *i*-Pr_2NEt (1.87 mL, 10.75 mmol) for 3 h at 0 °C and purification on a short column using C-2 silica gel[1] (1 cm x 1 cm, eluting with 100% Hexane) furnished 209 mg (84% yield) of **10a/10a'** as a yellow oil exhibiting spectra data identical to that reported above.

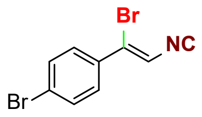

10b

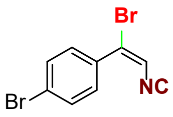

10b'

*Z- and E-1-Bromo-4-(1-bromo-2-isocyanovinyl)benzene (**10b** and **10b'**)*. Following the general bromoisocyanoalkene synthesis A with (*E*)-*N*-(4-bromostyryl)formamide (100 mg, 0.442 mmol), bromine (24 μL 0.464 mmol), triethylamine (0.18 mL, 1.33 mmol), $POCl_3$ (0.12 mL, 1.33 mmol) and *i*-Pr_2NEt (0.69 mL, 3.98 mmol). Purification on C-2 silica[1] (1 cm x 2 cm column) with 100% hexanes as the eluant, furnished 93.9 mg (74%) of **10b** as a black oil consisting of a 4:1 ratio of *Z:E*-diastereomers. Repeated purification by column chromatography on C-2 silica gel afforded pure samples of each diastereomer. For *(Z)-1-bromo-4-(1-bromo-2-isocyanovinyl)benzene*: IR (ATR) 3068, 2924, 2115, 1585 cm^{-1}; HRMS (+EI) m/z [M^+] Calcd. for $C_9H_5Br_2N$, 284.8789 found 284.8788; ^{1}H NMR (400 MHz, $CDCl_3$) δ 7.62 – 7.55 (m, 2H), 7.53 – 7.46 (m, 2H), 6.41 (s, 1H); ^{13}C{1H} NMR (101 MHz, $CDCl_3$) δ 169.1, 133.1, 131.9, 130.8, 130.4, 125.1, 112.2. For (*E*)-1-

bromo-4-(1-bromo-2-isocyanovinyl)benzene : δ 7.55 – 7.51 (m, 2H), 7.41 – 7.37 (m, 2H), 6.56 (s, 1H); ^{13}C{1H} NMR (101 MHz, $CDCl_3$) δ 170.9, 133.9, 132.1, 130.8, 129.1, 125.3, 112.6.

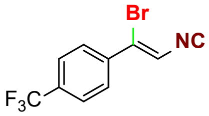

10c

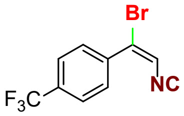

10c'

Z- and E-1-(1-Bromo-2-isocyanovinyl)-4-(trifluoromethyl)benzene ***(10c** and **10c')***. Following the general bromoisocyanoalkene synthesis A with (*E*)-N-(4-(trifluoromethyl)styryl)formamide[9] (71 mg, 0.33 mmol), bromine (18 μL 0.35 mmol), triethylamine (1.34 mL, 0.99 mmol), $POCl_3$ (90 μL, 0.99 mmol), and *i*-Pr_2NEt (0.52 mL, 2.9 mmol) and purification on C-2 silica[1] (1 cm x 2 cm column) with 100% hexanes as the eluant, furnished 48 mg (53%) of *Z- and E-1-(1-bromo-2-isocyanovinyl)-4-(trifluoromethyl)benzene* ***(10c** and **10c')*** as a yellow oil consisting of a 2.6:1 ratio of *Z:E*-diastereomers: IR 3060, 2926, 2128, 2089 cm^{-1}; HRMS (ESI) m/z [M+H$^+$] Calcd for$C_{10}H_5F_3N$ 274.9557, found 274.9565. Repeated purification by column chromatography on C-2 silica gel afforded pure samples of each diastereomer. For *(Z)-1-(1-bromo-2-isocyanovinyl)-4-(trifluoromethyl)benzene* (**10c**): ^{1}H NMR (400 MHz, $CDCl_3$) δ 7.71 – 7.64 (m, 4H), 6.63 (s, 1H); ^{13}C{1H} NMR (101 MHz, $CDCl_3$) δ 171.6, 138.4, δ 132.51 (q, J = 33.0 Hz), 130.3, 128.1, 125.88 (q, J = 3.8 Hz), 123.46 (q, J = 272.5 Hz), 113.8 (br s). For *(E)-1-(1-bromo-2-isocyanovinyl)-4-(trifluoromethyl)benzene* (**10c'**): ^{1}H NMR (400 MHz, $CDCl_3$) δ 7.76 – 7.69 (m, 4H), 6.48 (s, 1H); ^{13}C{1H} NMR (101 MHz, $CDCl_3$) δ 167.0, 135.1 (q, J = 1.5 Hz), 129.8 (q, J = 32.8 Hz), 127.6, 126.8, 123.1 (q, J = 3.8 Hz), 120.9 (q, J = 272.5 Hz) 110.6 (br s).

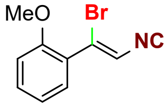

10d

(Z)-1-(1-Bromo-2-isocyanovinyl)-2-methoxybenzene ***(10d)***. Following the general bromoisocyanoalkene synthesis A with *(E)-N-(2-methoxystyryl)formamide*[9] (80

mg, 0.45 mmol), bromine (24 µL 0.47 mmol), triethylamine (0.18 mL, 1.4 mmol), $POCl_3$ (124 µL, 1.4 mmol) and *i*-Pr_2NEt (0.71 mL, 4.1 mmol) with purification on C-2 silica[1] (1 cm x 2 cm column) with 100% hexanes as the eluant, furnished 70 mg (65%) of *(Z)-1-(1-Bromo-2-isocyanovinyl)-2-methoxybenzene* ***(10d)*** as a black oil ; IR (ATR) 3064, 2942, 2122, 2069 cm^{-1}; ^1H NMR (400 MHz, $CDCl_3$) δ 7.47 (dd, *J* = 7.7, 1.7 Hz, 1H), 7.39 – 7.34 (m, 1H), 7.02 – 6.97 (m, 1H), 6.93 (d, *J* = 8.3 Hz, 1H), 6.70 (s, 1H), 3.88 (s, 3H); $^{13}C\{1H\}$ NMR (101 MHz, $CDCl_3$) δ 169.9, 156.5, 131.7, 131.6, 127.1, 123.8, 120.7, 115.7 (t, *J* = 13.8 Hz), 111.3, 55.8. HRMS (ESI) m/z [M+H$^+$] Calcd for$C_{10}H_9BrNO$ 237.9868, found 237.9872.

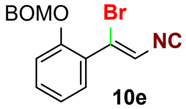

10e

(Z)-1-((Benzyloxy)methoxy)-2-(1-bromo-2-isocyanovinyl)benzene ***(10e)***. Following the general bromoisocyanoalkene synthesis B with (*Z*)-*N*-(2-(2-((benzyloxy)methoxy)phenyl)-2-bromovinyl)formamide (**17e**, 32 mg, 0.09 mmol), $POCl_3$ (25 µL, 0.265 mmol.), and *i*-Pr_2NEt (0.14 mL, 0.795 mmol) for 3 h at 0 °C and purification on a short C-2 silica gel column[1] (1 cm x 1 cm, eluting with 100% Hexane) furnished 20.6 mg (68% yield) of *(Z)-1-((Benzyloxy)methoxy)-2-(1-bromo-2-isocyanovinyl)benzene* ***(10e)*** as a yellow oil; IR 3062, 2917, 2122, 1595 cm^{-1}; ^1H NMR (400 MHz, $CDCl_3$) δ 7.41 (dd, *J* = 7.7, 1.7 Hz, 1H), 7.39 – 7.29 (m, 6H), 7.24 (dd, *J* = 8.4, 1.1 Hz, 1H), 7.08 – 7.02 (m, 1H), 6.50 (s, 1H), 5.36 (s, 2H), 4.74 (s, 2H); $^{13}C\{1H\}$ NMR (101 MHz, $CDCl_3$) 169.9, 154.3, 136.8, 131.6, 131.3, 128.5, 128.1, 128.0, 127.1, 124.9, 121.8, 115.6, 114.9, 92.5, 70.8; HRMS (ESI) m/z [M+H$^+$] Calcd for $C_{17}H_{15}BrNO_2$ 344.0286, found 344.0294.

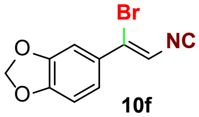

10f

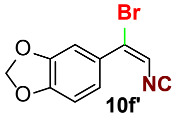

10f'

Z- and E-5-(1-Bromo-2-isocyanovinyl)benzo[d][1,3]dioxole ***(10f** and **10f')***. Following the general bromoisocyanoalkene

synthesis A with (Z)-N-(2-(benzo[d][1,3]dioxol-5-yl)-2-bromovinyl)formamide[9] (60.1 mg, 0.33 mmol), bromine (11 μL 0.35 mmol), triethylamine (81 μL, 0.58 mmol), $POCl_3$ (54 μL, 0.58 mmol) and *i*-Pr_2NEt (0.3 mL, 1.74 mmol) and purification on C-2 silica[1] (1 cm x 2 cm column) with 100% hexanes as the eluant, furnished a 6.7:1 ratio of *Z:E*-diastereomers 40.1 mg (51%) of **10f** and **10f'** as a white solid that polymerized upon heating: IR 2908, 2110, 1608, 1502 cm^{-1}; HRMS (+ESI) m/z $[M+H]^+$ Calcd. for $C_{10}H_7BrNO_2$, 251.9660, found 251.9671. For (Z)-5-(1-bromo-2-isocyanovinyl)benzo[d][1,3]dioxole (**10f**): ^{1}H NMR (400 MHz, $CDCl_3$) δ 7.05 (dd, J = 8.2, 2.0 Hz, 1H), 6.99 (d, J = 2.0 Hz, 1H), 6.80 (d, J = 8.2 Hz, 1H), 6.44 (s, 1H), 6.03 (s, 2H); ^{13}C{1H} NMR (101 MHz, $CDCl_3$) δ169.7, 149.8, 148.1, 131.6, 129.0, 122.4, 111.1(m), 108.3, 107.9, 101.9. For (E)-5-(1-bromo-2-isocyanovinyl)benzo[d][1,3]dioxole (**10f'**): ^{1}H NMR (400 MHz, $CDCl_3$) δ 7.19 (dd, J = 8.2, 1.9 Hz, 1H), 7.10 (d, J = 1.9 Hz, 1H), 6.85 (d, J = 8.2 Hz, 1H), 6.33 (s, 1H), 6.04 (s, 2H); ^{13}C{1H} NMR (101 MHz, CDCl3) δ 168.1, 149.4, 147.7, 131.9, 127.9, 124.0, 110.9 (br s), 109.3, 108.2, 101.8.

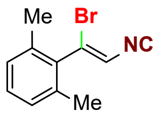

10g

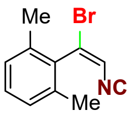

10g'

*Z- and E-2-(1-bromo-2-isocyanovinyl)-1,3-dimethylbenzene (**10g** and **10g'**)*. Following the general bromoisocyanoalkene synthesis A with (*E*)-*N*-(2,6-dimethylstyryl)formamide (**17g**, 200 mg, 1.1 mmol), bromine (62 μL, 1.2 mmol), triethylamine (0.47 mL, 3.4 mmol), $POCl_3$ (0.31 mL, 3.4 mmol) and *i*-Pr_2NEt (1.79 mL, 10.3 mmol) and purification on C-2 silica[1] (1 cm x 2 cm column) with 100% hexanes as the eluant, furnished 218 mg (81%) of **10g** as a clear oil consisting of a 13:1 ratio of *Z:E*-diastereomers): IR 3055, 2923, 2863, 2122 cm^{-1}; HRMS (+APPI) m/z $[M]^+$ Calcd. for $C_{11}H_{10}BrN$, 234.9991, found 235.0031. For (Z)-2-(1-bromo-2-isocyanovinyl)-1,3-dimethylbenzene (**10g**): ^{1}H NMR (400 MHz, $CDCl_3$) δ 7.22-7.17 (m, 1H), 7.09-7.05 (m, 1H), 6.20

(s, 1H), 2.29 (s, 6H); $^{13}C\{1H\}$ NMR (101 MHz, $CDCl_3$) δ 169.7, 136.7, 134.7, 129.8, 129.4, 127.8, 115.5 (t, J = 11.6 Hz), 19.7. For (*E*)-2-(1-bromo-2-isocyanovinyl)-1,3-dimethylbenzene (**10g'**): ^1H NMR (400 MHz, $CDCl_3$) δ 7.20 – 7.17 (m, 1H), 7.12 – 7.08 (m, 2H), 6.46 (s, 1H), 2.31 (s, 6H); $^{13}C\{1H\}$ NMR (101 MHz, $CDCl_3$) δ 167.2, 135.6, 133.7, 132.3, 130.0, 128.0, 115.1, 19.3.

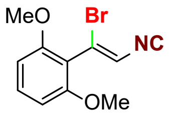

10h

(Z)-2-(1-Bromo-2-isocyanovinyl)-1,3-dimethoxybenzene ***(10h)***. Following the general bromoisocyanoalkene synthesis A with (*Z*)-*N*-(2-bromo-2-(2,6-dimethoxyphenyl)vinyl)formamide (**17h**, 124 mg, 0.66 mmol), bromine (110.4 mg 0.63 mmol), triethylamine (181.7 mg, 1.8 mmol), $POCl_3$ (275.3 mg, 1.8 mmol) and *i*-Pr_2NEt (696.1 mg, 5.4 mmol) and purification on C-2 silica[1] (1 cm x 2 cm column) with 100% hexanes as the eluant, furnished 106 mg (66%) of *(Z)-2-(1-Bromo-2-isocyanovinyl)-1,3-dimethoxybenzene* ***(10h)*** as a white solid: m.p. 121.5 – 123.9 °C; IR 3059, 2936, 2839, 2121 cm^{-1}; ^1H NMR (400 MHz, $CDCl_3$) δ 7.31 (t, J = 8.5 Hz, 1H), 6.55 (d, J = 8.5 Hz, 1H), 6.19 (s, 1H), 3.83 (s, 6H); $^{13}C\{1H\}$ NMR (101 MHz, $CDCl_3$) δ 169.0, 158.0, 131.8, 122.7, 116.6 (t, J = 14.2 Hz) 113.4, 103.8, 56.0; HRMS (+EI) m/z $[M]^+$ Calcd. for $C_{11}H_{10}BrNO_2$, 266.9895, found 266.9916.

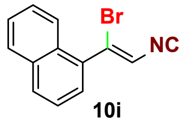

10i

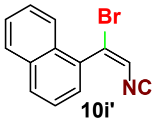

10i'

Z- and E-1-(1-Bromo-2-isocyanovinyl)naphthalene ***(10i and 10i')***. Following the general bromoisocyanoalkene synthesis A with (*E*)-*N*-(2-(naphthalen-1-yl)vinyl)formamide[9] (768 mg, 3.89 mmol), bromine (0.21 mL 4.1 mmol), triethylamine (1.6 mL, 12 mmol), $POCl_3$ (1.1 mL, 12 mmol) and *i*-Pr_2NEt (6.1 mL, 35 mmol) and purification on C-2 silica[1] (1 cm x 2 cm column) with 100% hexanes as the eluant, furnished 750 mg (74%) as a yellow oil consisting of a 6:1 ratio of *Z:E*-diastereomers of ***10i/10i'***: IR 3056, 2935, 2861, 2212 cm^{-1}; HRMS (+APCI) m/z $[M+H^+]$ Calcd for $C_{13}H_9BrN$, 257.9918,

found 257.9912. Repeated purification by column chromatography on C-2 silica gel afforded pure samples of each diastereomer. For *(Z)-1-(1-bromo-2-isocyanovinyl)naphthalene (**10i**)*: ^{1}H NMR (400 MHz, $CDCl_3$) δ 8.02 (d, *J* = 8.3 Hz, 1H), 7.96 – 7.86 (m, 2H), 7.65 – 7.51 (m, 2H), 7.49 – 7.44 (m, 2H), 6.45 (s, 1H); ^{13}C{1H} NMR (101 MHz, $CDCl_3$) δ 170.0, 133.6, 133.2, 130.9, 130.5, 129.2, 128.6, 127.3, 127.2, 126.7, 125.0, 124.8, 115.9 (br t, *J* = 11.3 Hz). For *(E)-1-(1-bromo-2-isocyanovinyl)naphthalene (**10i'**)*: ^{1}H NMR (400 MHz, $CDCl_3$) δ 8.00 – 7.88 (m, 3H), 7.64 – 7.51 (m, 4H), 6.63 (s, 1H); ^{13}C{1H} NMR (101 MHz, $CDCl_3$) δ 167.8, 133.7, 132.2, 130.94, 130.87, 129.3, 128.7, 127.2, 127.0, 126.7, 125.2, 124.5, 115.5 (br s).

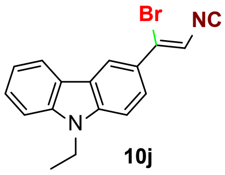

10j

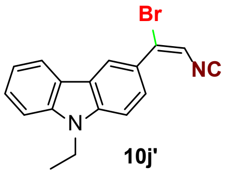

10j'

*Z- and E-3-(1-bromo-2-isocyanovinyl)-9-ethyl-9H-carbazole (**10j** and **10j'**).* Following the general bromoisocyanoalkene synthesis A with *(E)-N-(2-(9-Ethyl-9H-carbazol-3-yl)vinyl)formamide* (**17j**, 68 mg, 0.26 mmol), bromine (43.2 mg 0.27 mmol), triethylamine (78.1 mL, 0.77 mmol), $POCl_3$ (118.4 mg, 0.77 mmol) and *i*-Pr_2NEt (299.3 mg, 2.32 mmol) and purification on C-2 silica[1] (1 cm x 2 cm column) with 100% hexanes as the eluant, furnished 54 mg (65%) of a clear oil consisting of a 6.6:1 ratio of *Z*:*E*-diastereomers **10j**/**10j'**: IR 3058, 2978, 2932, 2122 cm^{-1}; HRMS (+ESI) m/z [M+H^{+}] Calcd for $C_{17}H_{15}BrN_2O$, 343.0446, found 343.0435. For *Z-3-(1-bromo-2-isocyanovinyl)-9-ethyl-9H-carbazole* (**10j**): ^{1}H NMR (400 MHz, $CDCl_3$) δ 8.28 (d, *J* = 2.0 Hz, 1H), 8.12 (dt, *J* = 7.8, 1.0 Hz, 1H), 7.63 (dd, *J* = 8.7, 2.0 Hz, 1H), 7.53 (ddd, *J* = 8.3, 7.1, 1.2 Hz, 1H), 7.44 (dt, *J* = 8.3, 1.0 Hz, 1H), 7.38 (d, *J* = 8.7 Hz, 1H), 7.29 (ddd, *J* = 8.0, 7.1, 1.0 Hz, 1H), 6.60 (s, 1H), 4.38 (q, *J* = 7.3 Hz, 2H), 1.45 (t, *J* = 7.3 Hz, 3H); ^{13}C{1H} NMR (101 MHz, $CDCl_3$) δ 168.8, 141.1, 140.6, 133.4, 126.6, 125.6, 125.2, 123.1, 122.5, 120.7, 120.5, 119.8, 110.2 (br s), 109.0, 108.6, 37.8, 13.8. For *E-3-(1-bromo-2-isocyanovinyl)-9-*

ethyl-9H-carbazole (**10j'**): ^{1}H NMR (400 MHz, $CDCl_3$) δ 8.40 (d, J = 1.9 Hz, 1H), 8.13 (d, J = 7.8 Hz, 1H), 7.79 (dd, J = 8.6, 1.9 Hz, 1H), 7.56 – 7.48 (m, 1H), 7.43 (t, J = 1.8 Hz, 2H), 7.33 – 7.27 (m, 1H), 6.42 (s, 1H), 4.40 (q, J = 7.2 Hz, 2H), 1.46 (t, J = 7.2 Hz, 3H); ^{13}C{1H} NMR (101 MHz, $CDCl_3$) δ 167.0, 140.8, 140.5, 133.9, 126.6, 126.5, 124.6, 122.74, 122.66, 122.0, 120.7, 119.7, 109.9 (m), 108.9, 108.2, 37.8, 13.9.

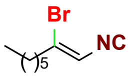

10k

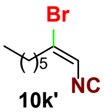

10k'

*Z- and E-2-Bromo-1-isocyanooct-1-ene (**10k** and **10k'**).* Following the general bromoisocyanoalkene synthesis A with (*E*)-*N*-(oct-1-en-1-yl)formamide (92 mg, 0.59 mmol), bromine (99.5 mg, 0.63 mmol), triethylamine (179.9 mg, 0.77 mmol), $POCl_3$ (272.6 mg, 1.78 mmol) and *i*-Pr_2NEt (689.4 mg, 5.33 mmol) and purification on C-2 silica[1] (1 cm x 2 cm column) with 100% hexanes as the eluant, furnished 98 mg (76%) of a clear oil consisting of a 1.5:1 ratio of *Z:E*-diastereomers **10k** and **10k'**: IR 2955, 2929, 2858, 2118 cm^{-1}. HRMS (+ESI) m/z [M^+] Calcd for $C_9H_{14}BrN$, 215.0304, found 215.0316. For Z-2-bromo-1-isocyanooct-1-ene (**10k**): ^{1}H NMR (400 MHz, $CDCl_3$) δ 6.14 (t, J = 1.3 Hz, 1H), 2.54 – 2.41 (m, 2H), 1.66 – 1.46 (m, 3H), 1.37 – 1.14 (m, 5H), 0.88 (br. t, J = 6.6 Hz, 3H); ^{13}C{1H} NMR (101 MHz, $CDCl_3$) δ 167.2, 135.7, 111.9 (m), 35.7, 31.3, 28.01, 27.8, 22.4, 14.00. For *E-2-bromo-1-isocyanooct-1-ene* (**10k'**): ^{1}H NMR (400 MHz, $CDCl_3$) δ 6.11 (s, 1H), 2.67 (t, J = 7.4 Hz, 2H), 1.65 – 1.50 (m, 2H), 1.43 – 1.21 (m, 5H), 0.95 – 0.81 (m, 3H); ^{13}C{1H} NMR (101 MHz, $CDCl_3$) δ 167.0, 138.5, 112.8 (m), 38.2, 31.4, 27.98, 27.2, 22.5, 14.01.

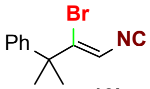

10l

(Z)-(3-Bromo-4-isocyano-2-methylbut-3-en-2-yl)benzene (10l). Following the general bromoisocyanoalkene synthesis A with (*Z*)-*N*-(3-methyl-3-phenylbut-1-en-1-yl)formamide (**17l**, 82.8 mg, 0.44 mmol), bromine (24 µL, 0.46 mmol), triethylamine (0.18 mL,

1.31 mmol), $POCl_3$ (21 μL, 1.31 mmol) and *i*-Pr_2NEt (0.69 mL, 3.94 mmol) and purification on C-2 silica[1] (1 cm x 2 cm column) with 100% hexanes as the eluant, furnished 66.9 mg (61%) of *(Z)*-(3-bromo-4-isocyano-2-methylbut-3-en-2-yl)benzene (**10l**) as a clear oil: IR 3062, 2978, 2929, 2126 cm^{-1}; ^{1}H NMR (400 MHz, $CDCl_3$) δ 7.38 – 7.32 (m, 2H), 7.30 – 7.25 (m, 3H), 6.22 (s, *J* = 0.8 Hz, 1H), 1.60 (s, *J* = 0.7 Hz, 6H); ^{13}C{1H} NMR (101 MHz, $CDCl_3$) δ 168.5, 145.7, 144.5, 128.6, 127.2, 126.0, 112.4 (t, *J* = 14.3 Hz), 46.4, 28.6; HRMS (+EI) m/z [M^+] Calcd. for $C_{12}H_{12}BrN$, 249.0153, found 249.0152.

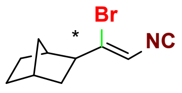

10m

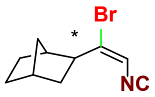

10m'

Z- and E-(1S,4R*)-2-(1-bromo-2-isocyanovinyl)bicyclo-[2.2.1]heptane (**10m** and **10m'**).* Following the general bromoisocyanoalkene synthesis A with *N-((Z)-2-((1S,4R)-Bicyclo[2.2.1]heptan-2-yl)vinyl)formamide* (**17m** as a 1.1:1 ratio of *endo*- and *exo*-diastereomers, 50.5 mg, 0.305 mmol), bromine (16 μL 0.32 mmol), triethylamine (0.13 mL, 0.92 mmol), $POCl_3$ (86 μL, 0.916 mmol) and *i*-Pr_2NEt (0.48 mL, 2.75 mmol) and purification on C-2 silica[1] (1 cm x 2 cm column) with 100% hexanes as the eluant, furnished 48.8 mg (71%) of a clear oil consisting of 4.3:1 ratio of *Z:E*-diastereomers **10m** and **10m'**: IR 2955, 2872, 2123, 1454 cm^{-1}; HRMS (+EI) m/z [M^+] Calcd. for $C_{10}H_{12}BrN$, 225.0147, found 225.0158. The ^{1}H NMR of (1*S**,4*R**)-2-((*Z*)-1-bromo-2-isocyanovinyl)-bicyclo[2.2.1]heptane was a 1:1.1 mixture of *endo*- and *exo*- diastereomers. For the major diastereomer of **10m**: ^{1}H NMR (400 MHz, $CDCl_3$) δ 6.22 (d, *J* = 2.2 Hz, 1H), 3.01 – 2.91 (m, 1H), 2.57 (t, *J* = 4.3 Hz, 1H), 2.41 – 2.32 (m, 2H), 1.82 – 1.73 (m, 1H), 1.68 – 1.08 (m, 6H); ^{13}C{1H} NMR (101 MHz, $CDCl_3$) δ 167.6, 140.7, 111.8 (m), 49.5, 41.8, 39.2, 37.6, 36.05, 29.86, 22.9, For the minor diastereomer of **10m**: ^{1}H NMR (400 MHz, $CDCl_3$) δ 6.14 (d, *J* = 1.5 Hz, 1H), 2.55 – 2.48 (m, 1H), 2.41 – 2.32 (m, 2H), 1.68 – 1.08 (m, 8H). ^{13}C{1H} NMR (101 MHz,

$CDCl_3$) δ 167.8, 138.8, 110.3 (m), 50.2, 41.4, 37.9, 36.7, 33.7, 29.93, 28.3. The ^{1}H NMR of (1*S**,4*R**)-2-((*E*)-1-bromo-2-isocyanovinyl)bicyclo[2.2.1]heptane **10m'** was a 1:1.4 of *endo*- and *exo*-diastereomers. For the major diastereomer of **10m'**: ^{1}H NMR (400 MHz, $CDCl_3$) δ 6.23 (d, *J* = 1.3 Hz, 1H), 3.35 – 3.25 (m, 1H), 2.64 (t, *J* = 4.4 Hz, 1H), 2.36 – 2.30 (m, 1H), 1.87-1.77 (m, 1H), 1.73 - 1.13 (m, 7H); $^{13}C\{1H\}$ NMR (101 MHz, $CDCl_3$) δ 167.6 (m), 142.8, 113.44 (t, *J* = 11.7 Hz), 44.4, 42.6, 41.0, 37.4, 35.2, 28.9, 23.8. For the minor diastereomer **10m'**: ^{1}H NMR (400 MHz, $CDCl_3$) δ 6.05 (s, 1H), 2.96 (dd, *J* = 8.8, 6.1 Hz, 1H), 2.35 – 2.30 (m, 1H), 2.27 (s, 1H), 1.89 – 1.77 (m, 1H), 1.74 – 1.15 (m, 7H); $^{13}C\{1H\}$ NMR (101 MHz, $CDCl_3$) δ 167.1 (m), 144.1, 111.4 (m), 44.7, 43.0, 37.7, 37.0, 35.9, 30.8, 28.0.

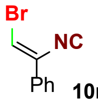
10n

*(Z)-(2-Bromo-1-isocyanovinyl)benzene (**10n**).* Following the general bromoisocyanoalkene synthesis B with (*Z*)-*N*-(2-bromo-1-phenylvinyl)formamide (**18n**, 79 mg, 0.35 mmol), $POCl_3$ (98.1 μL, 1.05 mmol), and *i*-Pr_2NEt (0.54 mL, 3.15 mmol) for 3 h at 0 °C and purification on a short C-2 silica gel1 column (1 cm x 1 cm, eluting with 100% Hexane) furnished 52.1 mg (72%) of *(Z)*-(2-bromo-1-isocyanovinyl)benzene (**10n**) as a clear oil: IR (ATR) 3088, 2918 2850, 2114 cm^{-1}; ^{1}H NMR (400 MHz, CDC13) δ 7.59 – 7.50 (m, 2H), 7.48 – 7.39 (m, 3H), 6.98 (s, 1H); $^{13}C\{1H\}$ NMR (101 MHz, $CDCl_3$) δ 170.5, 131.8 (t, *J* = 13.0 Hz) , 131.2, 130.2, 129.1, 125.2, 108.0; HRMS (+EI) m/z [M+H] Calcd. for C_9H_6BrN 206.9684, found 206.9686.

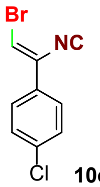
10o

*(Z)-1-(2-Bromo-1-isocyanovinyl)-4-chlorobenzene (**10o**).* Following the general bromoisocyanoalkene synthesis B with (*Z*)-*N*-(2-bromo-1-(4-chlorophenyl)vinyl)formamide (**18o**, 16.5 mg, 63 μmol), $POCl_3$ (29.1 mg, 0.19 mmol),

and *i*-Pr_2NEt (73.7 mg, 0.57 mmol) for 3 h at -20 °C and purification on a short C-2 silica gel[1] column (1 cm x 1 cm, eluting with 100% Hexane) furnished 10.4 mg (67%) of *(Z)-1-(2-Bromo-1-isocyanovinyl)-4-chlorobenzene* ***(10o)*** as a crystalline solid: mp 57.3 – 59.2 °C; IR (ATR) 3080, 2922, 2121, 1594 cm^{-1}; 1H NMR (400 MHz, $CDCl_3$) δ 7.51 – 7.44 (m, 2H), 7.43 – 7.37 (m, 2H), 6.99 (s, 1H); $^{13}C\{1H\}$ NMR (101 MHz, $CDCl_3$) δ 171.1, 136.3, 130.8, 129.7, 129.4, 126.4, 108.6; HRMS (+ESI) m/z [M+H] Calcd. for C_9H_5BrClN 240.9288, found 240.9299.

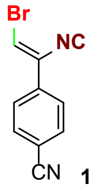
10p

(Z)-4-(2-Bromo-1-isocyanovinyl)benzonitrile ***(10p)***. Following the general bromoisocyanoalkene synthesis B with (*Z*)-*N*-(2-bromo-1-(4-cyanophenyl)vinyl)formamide (**18p**, 19.8 mg, 79 μmol), $POCl_3$ (36.3 mg, 0.24 mmol), and *i*-Pr_2NEt (91.4 mg, 0.71 mmol) for 3 h at -20 °C and purification on a short C-2 silica gel[1] column (1 cm x 1 cm, eluting with 100% Hexane) furnished 11.3 mg (61%) of *(Z)*-4-(2-bromo-1-isocyanovinyl)benzonitrile (**10p**) as a crystalline solid: mp 142.7 – 143.8 °C; IR (ATR) 3083, 2928, 2224, 2124 cm^{-1}; ^1H NMR (400 MHz, $CDCl_3$) δ 7.77 – 7.72 (m, 2H), 7.69 – 7.64 (m, 2H), 7.20 (s, 1H); $^{13}C\{1H\}$ NMR (101 MHz, $CDCl_3$) δ 172.2, 135.1, 133.0, 130.4, 125.7, 117.8, 113.8, 111.9. Attempts to obtain HRMS data on a Waters Q-Tof Premier, time-of-flight, LCMS with electrospray ionization (ESI) and a scimaX FT-ICR instrument with an atmospheric pressure photoionization (APPI) have not been successful, probably because the isocyanide is very sensitive to heat.[13] Complete identification therefore rests on both the data provided and by conversion into thiazole **12p** whose characterization includes HRMS.

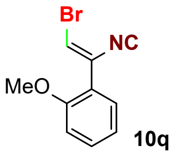

10q

(Z)-1-(2-Bromo-1-isocyanovinyl)-2-methoxybenzene ***(10q)***. Following the general bromoisocyanoalkene synthesis B with (*Z*)-*N*-(2-bromo-1-(2-methoxyphenyl)vinyl)formamide (**18q**, 43.4 mg, 169 µmol), $POCl_3$ (36.3 mg, 0.24 mmol), and *i*-Pr_2NEt (91.4 mg, 0.71 mmol) for 3 h at -20 °C and purification on a short C-2 silica gel[1] column (1 cm x 1 cm, eluting with 100% Hexane) furnished 31.2 mg (77%) of *(Z)-1-(2-Bromo-1-isocyanovinyl)-2-methoxybenzene* ***(10q)*** as clear oil: IR (ATR) 2940, 2842, 2116, 1599 cm^{-1}; ^1H NMR (400 MHz, CDCl3) δ 7.55 (dd, *J* = 7.7, 1.5 Hz, 1H), 7.42 – 7.34 (m, 1H), 7.08 – 6.98 (m, 1H), 6.96 (d, *J* = 8.3 Hz, 1H), 3.90 (s, 3H); $^{13}C\{1H\}$ NMR (101 MHz, $CDCl_3$) δ 168.4, 156.6, 131.1, 129.0, 128.4, 120.9, 119.9, 112.7, 111.5, 55.7; HRMS (+ESI) m/z [M+H] Calcd. for $C_{10}H_8BrNO$ 237.9868, found 237.9872.

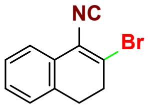

10r

3-Bromo-4-isocyano-1,2-dihydronaphthalene (10r). Following the general bromoisocyanoalkene synthesis A with *N*-(3,4-dihydronaphthalen-1-yl)formamide (**17r**, 22 mg, 0.13 mmol), bromine (21.3 mg 0.13 mmol), triethylamine (38.6 mg, 0.38 mmol), $POCl_3$ (58.4 mg, 0.38 mmol) and *i*-Pr_2NEt (147.8 mL, 1.2 mmol) and purification on C-2 silica[1] (1 cm x 2 cm column) with 100% hexanes as the eluant, furnished 13.4 mg (45%) of **10r** as a yellow oil: IR (ATR) 2923, 2836, 2115, 1618 cm^{-1}; ^1H NMR (400 MHz, $CDCl_3$) δ 7.50 – 7.44 (m, 1H), 7.36 – 7.26 (m, 2H), 7.17 – 7.12 (m, 1H), 3.02 – 2.86 (m, 4H); $^{13}C\{1H\}$ NMR (101 MHz, $CDCl_3$) δ 168.8, 133.1, 129.2, 128.0, 127.7, 127.4, 125.1 (t, *J* = 13.6 Hz), 124.8, 123.0, 33.4, 28.1; HRMS (+EI) m/z [M+H] Calcd. for $C_{11}H_8BrN$ 232.9840, found 232.9826.

General Thiazole Synthesis Procedure A: Cs_2CO_3 (1.5 mmol, 1.5 equiv.) and NaSH (1.8 mmol, 1.8 equiv.) were added to a rt, CH_3CN solution (0.1 M) of the bromoisocyanoalkene (1 mmol, 1

equiv.). After 16 h, the reaction mixture was filtered through a pad of silica, concentrated, and purified by flash chromatography (hexanes: ethyl acetate, 85:15) to afford the pure thiazole.

General Thiazole Synthesis Procedure B: Neat *i*-Pr_2NEt (1.3 mmol, 1.3 equiv.) and solid NaSH (1.3 mmol, 1.3 equiv.) were added to a rt, ethanolic solution (0.1 M) of the bromoisocyanoalkene (1 mmol, 1 equiv.). After 16 h, the reaction mixture was filtered through a pad of silica, concentrated, and purified by flash chromatography (hexanes: ethyl acetate, 85:15) to afford the pure thiazole.

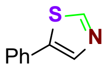

12a

*5-Phenylthiazole (**12a**)*. Following the general thiazole synthesis procedure A with 7:1 ratio of *Z*/*E*-(1-bromo-2-isocyanovinyl)benzene (**10a**, 50 mg, 0.24 mmol), NaSH (17.5 mg, 0.312 mmol), Cs_2CO_3 (117.5 mg, 0.36 mmol) and purification by MPLC (4 g silica gel cartridge, eluting with EtOAc/Hexanes 10% to 15 %) furnished 31.8 mg (82%) of thiazole **12a** as a colorless oil identical to material previously reported,[14] and 1.6 mg (13%) of recovered *E*-**10a** as a yellow oil, identical to material previously isolated. **From 20a**: Cs_2CO_3 (64.6 mg, 0.20 mmol) and NaSH (13.3 mg, 0.24 mmol.) were added to a rt, CH_3CN solution (0.1 M) of pure (Z)-(1-iodo-2-isocyanovinyl)benzene **20a**, (33.7 mg, 0.13 mmol). After 16 h, the reaction mixture was filtered through a pad of silica, concentrated, and purified by flash chromatography (hexanes: ethyl acetate, 85:15) to afford 12.4 mg (58%) of the pure thiazole as a yellow oil, identical to material previously isolated.

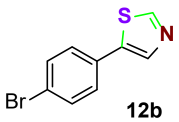

12b

*5-(4-Bromophenyl)thiazole (**12b**)*. Following general thiazole synthesis procedure B with 4:1 ratio of *Z*/*E*-1-bromo-4-(1-bromo-2-isocyanovinyl)benzene (**10b**, 39

mg, 0.14 mmol), NaSH (11.4 mg, 0.20 mmol), *i*-Pr_2NEt (22.9 mg, 0.18 mmol) and purification by MPLC (4 g silica gel cartridge, eluting with 10% to 15% EtOAc/Hexanes) furnished 17.4 mg (54%) of thiazole **12b** as a white solid: mp 63.8- 66.2 °C; IR (ATR) 3071, 2922, 1899, 1521 cm^{-1}; ^{1}H NMR (400 MHz, $CDCl_3$) δ 8.77 (s, 1H), 8.06 (s, 1H), 7.59 – 7.51 (m, 2H), 7.47 – 7.41 (m, 2H); ^{13}C{1H} NMR (101 MHz, $CDCl_3$) δ 152.4, 139.3, 138.2, 132.3, 130.0, 128.4, 122.4; HRMS (+ESI) m/z [M+H] Calcd. for C_9H_7BrNS 239.9483, found 239.9501.

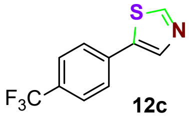
12c

5-(4-(Trifluoromethyl)phenyl)thiazole ***(12c)***. Following the general thiazole synthesis procedure B with 2.6:1 ratio of *Z*/*E*-1-(1-bromo-2-isocyanovinyl)-4-(trifluoromethyl)benzene (**10c**, 36 mg, 0.13 mmol), NaSH (9.5 mg, 0.17 mmol), *i*-Pr_2NEt (21.9 mg, 0.17mmol), and purification by MPLC (4 g silica gel cartridge, eluting with EtOAc/Hexanes 10% to 15 %) furnished 16.1 mg (54%) of **12c** as yellow solid: mp 44.6 - 47.7 °C exhibiting spectral data identical to that of previously reported material.[15]

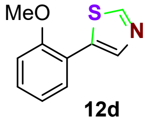
12d

5-(2-Methoxyphenyl)thiazole ***(12d)***. Following general thiazole synthesis procedure A with (*Z*)-1-(1-bromo-2-isocyanovinyl)-2-methoxybenzene (**10e**, 58 mg, 0.25 mmol), NaSH (24.6 mg, 0.44 mmol), Cs_2CO_3 (119.1 mg, 0.37 mmol) and purification by MPLC (4 g silica gel cartridge, eluting with EtOAc/Hexanes 10% to 15%) furnished 28.2 mg (61%) of thiazole **12e** as a clear oil: IR (ATR) 3071, 2938, 2836, 1598 cm^{-1}; ^{1}H NMR (400 MHz, $CDCl_3$) δ 8.78 (s, 1H), 8.27 (d, *J* = 1.1 Hz, 1H), 7.66 – 7.60 (m, 1H), 7.37 – 7.29 (m, 1H), 7.06 – 6.97 (m, 2H), 3.94 (s, 3H); ^{13}C{1H} NMR (101 MHz, $CDCl_3$) δ 155.4, 152.7, 141.0, 134.0, 129.4, 128.7, 121.0, 120.2, 111.5, 55.6; HRMS (+APCI) m/z [M+H] Calcd. for $C_{10}H_{10}NOS$ 192.0483, found 192.0477.

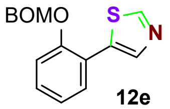
12e

*5-(2-((Benzyloxy)methoxy)phenyl)thiazole (**12e**)*. Following the general thiazole synthesis procedure A with pure (*Z*)-1-((benzyloxy)methoxy)-2-(1-bromo-2-isocyanovinyl)benzene (**10e**, 20.6 mg, 59.9 µmol), NaSH (6 mg, 0.11 µmol), Cs_2CO_3 (29.3 mg, 89 µmol) and purification by MPLC (4 g silica gel cartridge, eluting with EtOAc/Hexanes 10% to 15 %) furnished 10.9 mg (61%) of thiazole **12e** as a yellow oil: IR (ATR) 3068, 3033, 2851, 1697 cm^{-1}; ^1H NMR (400 MHz, $CDCl_3$) δ 8.81 (s, 1H), 8.27 (s, 1H), 7.67 – 7.64 (m, 1H), 7.39 – 7.27 (m, 7H), 7.13 – 7.05 (m, 1H), 5.41 (s, 2H), 4.73 (s, 2H); $^{13}C\{1H\}$ NMR (101 MHz, $CDCl_3$) δ 153.1, 153.0, 141.1, 136.8, 133.9, 129.5, 128.9, 128.5, 128.1, 128.0, 122.2, 120.8, 115.0, 92.2, 70.4; HRMS (+ESI) m/z [M+H] Calcd. for $C_{17}H_{16}NO_2S$ 298.0902, found 298.0912.

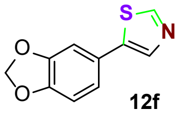
12f

*5-(Benzo[d][1,3]dioxol-5-yl)thiazole (**12f**)*. Following the general thiazole synthesis procedure A with 6.7:1 ratio of *Z*/E-5-(1-bromo-2-isocyanovinyl)benzo[d][1,3]dioxole (**10f**, 27.4 mg, 0.11 mmol), NaSH (10.9 mg, 0.19 mmol), Cs_2CO_3 (53.2 mg, 0.17 mmol), and purification by MPLC (4 g silica gel cartridge, eluting with EtOAc/Hexanes 10% to 15 %) furnished 14.6 mg (65%) of **12g** as a yellow solid: mp 95.6 - 97.2 °C, exhibiting spectral data identical to that of previously reported material.[15]

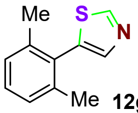
12g

*5-(2,6-Dimethylphenyl)thiazole (**12g**)*. Following the general thiazole synthesis procedure A with 14:1 ratio of *Z/E*-2-(1-bromo-2-isocyanovinyl)-1,3-dimethylbenzene (**10g**, 45 mg, 0.19 mmol), NaSH (19.3 mg, 0.34 mmol), Cs_2CO_3 (93.2 mg, 0.29 mmol) and purification by MPLC (4 g silica gel cartridge, eluting with EtOAc/Hexanes 10% to 15 %) furnished 18.7 mg (51%) of thiazole **12g** as a yellow solid: mp 62.5- 64.3 °C; IR (ATR) 3071,

2921 2853, 1607 cm^{-1}; ^{1}H NMR (400 MHz, $CDCl_3$) δ 8.91 (s, 1H), 7.65 (s, 1H), 7.25-7.19 (m, 1H), 7.12 (d, J = 7.8 Hz, 2H), 2.13 (s, 6H); ^{13}C{1H} NMR (101 MHz, $CDCl_3$) δ 153.5, 141.8, 138.5, 135.3, 129.7, 128.8, 127.5, 20.9; HRMS (+ESI) m/z [M+H] Calcd. for $C_{11}H_{12}NS$ 190.0690, found 190.0690. **At 1 mmol scale:** following general procedure A with a 14:1 ratio of *Z/E*-2-(1-bromo-2-isocyanovinyl)-1,3-dimethylbenzene (**10g**, 237 mg, 1 mmol), NaSH (101 mg, 1.8 mmol), Cs_2CO_3 (490 mg, 1.51 mmol) and purification by MPLC (4 g silica gel cartridge, eluting with EtOAc/Hexanes 10% to 15 %) furnished 90 mg (47%) of thiazole **12g** as yellow solid identical to material previously isolated.

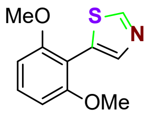
12h

*5-(2,6-Dimethoxyphenyl)thiazole (**12h**)*. Following the general thiazole synthesis procedure A with pure (*Z*)-2-(1-bromo-2-isocyanovinyl)-1,3-dimethoxybenzene (**10h**, 20.5 mg, 76.5 μmol), NaSH (7.7 mg, 0.14 mmol), Cs_2CO_3 (37.4 mg, 0.12 mmol), and purification by MPLC (4 g silica gel cartridge, eluting with EtOAc/Hexanes 10% to 15 %) furnished 12.1 mg (72%) of thiazole **12h** as white solid: m.p 49.8 – 51.2 °C; IR (ATR) 2936, 2838, 1584, 1473 cm^{-1}; ^{1}H NMR (400 MHz, $CDCl_3$) δ 8.82 (s, 1H), 8.40 (s, 1H), 7.28 (t, J = 8.4 Hz, 1H), 6.67 (d, J = 8.4 Hz, 2H), 3.88 (s, 6H); ^{13}C{1H} NMR (101 MHz, $CDCl_3$) δ 157.4, 152.5, 144.2, 129.3, 128.3, 109.2, 104.2, 55.9. HRMS (EI) m/z [M$^+$] Calcd. for $C_{11}H_{11}NO_2S$, 211.0156 found 211.0151.

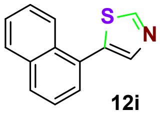
12i

*5-(Naphthalen-1-yl)thiazole (**12i**)*. Following the general thiazole synthesis procedure A with 1: 6.7 ratio E/*Z*-1-(1-bromo-2-isocyanovinyl)naphthalene (**10i**, 40.2 mg, 156 μmol), NaSH (15.2 mg, 0.280 mmol), Cs_2CO_3 (76.1 mg, 0.234 mmol) and purification by MPLC (4 g silica gel cartridge, eluting with EtOAc/Hexanes 10% to 15 %)

furnished 17 mg (51%) of thiazole **12i** as a colorless oil as identical to material previously reported.[14]

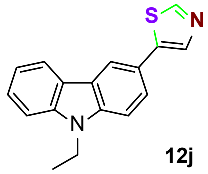

12j

*5-(9-Ethyl-9H-carbazol-3-yl)thiazole (**12j**).* Following the general thiazole synthesis procedure A with pure (*Z*)-3-(1-bromo-2-isocyanovinyl)-9-ethyl-9H-carbazole (**10j**, 19 mg, 58 µmol), NaSH (5.9 mg, 0.11 mmol), Cs_2CO_3 (28.6 mg, 88 µmol) and purification by MPLC (4 g silica gel cartridge, eluting with EtOAc/Hexanes 10% to 15%) furnished 10.5 mg (65%) of thiazole **12j** as yellow solid: mp 134.1 – 138.9 °C; IR (ATR) 3054, 2975, 2928, 1598 cm^{-1}; ^1H NMR (400 MHz, $CDCl_3$) δ 8.73 (s, 1H), 8.28 (d, *J* = 1.2 Hz, 1H), 8.14 (d, *J* = 7.9 Hz, 1H), 8.11 (s, 1H), 7.69 (dd, *J* = 8.5, 1.8 Hz, 1H), 7.51 (ddd, *J* = 8.2, 7.0, 1.2 Hz, 1H), 7.45 – 7.41 (m, 2H), 7.31 – 7.24 (m, 1H), 4.39 (q, *J* = 7.2 Hz, 2H), 1.46 (t, *J* = 7.2 Hz, 3H); $^{13}C\{1H\}$ NMR (101 MHz, $CDCl_3$) δ 151.0, 140.7, 140.4, 139.9, 137.9, 126.2, 124.9, 123.5, 122.6, 121.9, 120.6, 119.3, 119.1, 109.0, 108.8, 37.7, 13.8; HRMS (+APCI) m/z [M+H] Calcd. $C_{17}H_{15}NS$; 279.0956; found 279.0974.

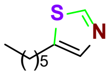

12k

*5-Hexylthiazole (**12k**).* Following the general thiazole synthesis procedure A with pure (*Z*)-2-bromo-1-isocyanooct-1-ene (**10k**, 45.4 mg, 0.21 mmol), NaSH (21.2 mg, 0.38 mmol), Cs_2CO_3 (102.7 mg, 0.32 mmol) and purification by MPLC (4 g silica gel cartridge, eluting with EtOAc/Hexanes 10% to 15 %) furnished 20.5 mg (58%) of thiazole **12k** as clear, colorless oil: IR (ATR) 2955, 2926, 2855, 1465 cm^{-1}; ^1H NMR (400 MHz, $CDCl_3$) δ 8.63 (s, 1H), 7.58 (s, 1H), 2.84 (t, *J* = 7.6 Hz, 2H), 1.66 (p, *J* = 7.2 Hz, 2H), 1.41 – 1.22 (m, 4H), 0.88 (t, *J* = 6.8 Hz, 3H); $^{13}C\{1H\}$ NMR (101 MHz, $CDCl_3$) δ 151.2, 140.2, 139.6, 31.6, 31.4, 28.6, 26.7, 22.5, 14.0;

HRMS (+APCI) m/z [M+H] Calcd. for $C_9H_{16}NS$ 170.1003, found 170.0996.

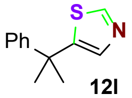
12l

5-(2-Phenylpropan-2-yl)thiazole (12l). Following the general thiazole synthesis procedure A with pure (*Z*)-(3-bromo-4-isocyano-2-methylbut-3-en-2-yl)benzene (**10l**, 35.2 mg, 0.14 mmol), NaSH (14.2 mg, 0.24 mmol), Cs_2CO_3 (69.0 mg, 0.22 mmol), and purification by MPLC (4 g silica gel cartridge, eluting with EtOAc/Hexanes 10% to 15 %) furnished 18 mg (63%) of **12l** as a yellow oil: IR 3061, 2971, 2871, 1701 cm^{-1}; ^{1}H NMR (400 MHz, $CDCl_3$) δ 8.68 (s, 1H), 7.65 (s, 1H), 7.32 – 7.27 (m, 4H), 7.24 – 7.19 (m, 1H), 1.79 (s, 6H); ^{13}C{1H} NMR (101 MHz, $CDCl_3$) δ 152.2, 150.7, 148.5, 139.2, 128.3, 126.5, 125.9, 40.1, 31.9; HRMS (EI) m/z [M+H$^+$] Calcd. for $C_{12}H_{14}NS$; 204.0847, found 204.0848.

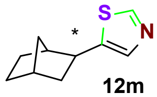
12m

5-((S,R*)-Bicyclo[2.2.1]heptan-2-yl)thiazole* (**12m**). Following the general thiazole synthesis procedure A with pure *Z-(1S*,4R*)-2-(1-bromo-2-isocyanovinyl)bicyclo-[2.2.1]heptane* (**10m**, 20.8 mg, 0.09 mmol), NaSH (9.3 mg, 0.17 mmol), Cs_2CO_3 (45.0 mg, 0.14 mmol), and purification by MPLC (4 g silica gel cartridge, eluting with EtOAc/Hexanes 10% to 15%) furnished 8.4 mg (51%) of a 1.2:1 ratio of *endo* and *exo* isomers of **12m** as a yellow oil: IR 3078, 2950, 2870, 1697 cm^{-1}; HRMS (EI) m/z [M+H$^+$] Calcd. for $C_{10}H_{14}NS$ 180.0847, found 180.0842. For the major diastereomer: ^{1}H NMR (400 MHz, $CDCl_3$) δ 8.66 (s, 1H), 7.60 (dd, J = 1.3, 0.7 Hz, 1H), 3.41 – 3.32 (m, 1H), 2.43 – 2.29 (m, 2H), 2.13 (dddd, J = 12.4, 11.5, 4.7, 3.1 Hz, 1H), 1.68 – 1.49 (m, 3H), 1.47 – 1.12 (m, 4H); ^{13}C{1H} NMR (101 MHz, $CDCl_3$) δ 151.3, 146.4, 139.8, 43.4, 40.8, 39.8, 37.3, 36.2, 30.0, 28.5. For the minor diastereomer: ^{1}H NMR (400 MHz, $CDCl_3$) δ 8.61 (d, J = 0.8 Hz, 1H), 7.57 (t, J = 0.9 Hz, 1H),

2.98 (dd, J = 9.0, 5.2 Hz, 1H), 2.43 – 2.30 (m, 2H), 1.86 (ddd, J = 12.8, 8.9, 2.4 Hz, 1H), 1.68 – 1.49 (m, 3H), 1.47 – 1.12 (m, 4H); $^{13}C\{1H\}$ NMR (101 MHz, $CDCl_3$) δ 151.0, 142.9, 138.6, 44.8, 40.4, 38.9, 37.0, 36.6, 29.8, 23.0.

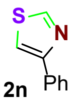

12n

*4-Phenylthiazole (**12n**).* Following the general thiazole synthesis procedure A with pure *(Z)*-(2-bromo-1-isocyanovinyl)benzene (**10n**, 24.7 mg, 0.12 mmol), NaSH (12.0 mg, 0.21 mmol), Cs_2CO_3 (58.1 mg, 0.18 mmol), and purification by MPLC (4 g silica gel cartridge, eluting with EtOAc/Hexanes 10% to 15 %) furnished 13.4 mg (70%) of thiazole **12o** as clear oil exhibiting spectral data identical to that of material previously reported.[15]

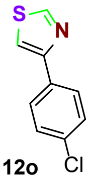

12o

*4-(4-Chlorophenyl)thiazole (**12o**).* Following the general thiazole synthesis procedure A with pure (*Z*)-1-(2-bromo-1-isocyanovinyl)-4-chlorobenzene (**10o**, 42.6 mg, 0.17 mmol), NaSH (17.8 mg, 0.32 mmol), Cs_2CO_3 (85.9 mg, 0.26 mmol) and purification by MPLC (4 g cartridge filled with silica gel,[1] eluting with EtOAc/Hexanes 10% to 15 %) furnished 28.2 mg (82 %) of **12o** exhibiting spectral properties identical to that of previously reported material.[15]

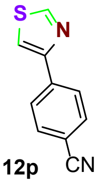

12p

*4-(Thiazol-4-yl)benzonitrile (**12p**).* Following the general thiazole synthesis procedure A with pure (*Z*)-4-(2-bromo-1-isocyanovinyl)benzonitrile (**10p**, 10 mg, 43 µmol), NaSH (4.3 mg, 77 µmol), Cs_2CO_3 (21.0 mg, 65 µmol), and purification by MPLC (4 g silica gel cartridge, eluting with EtOAc/Hexanes 10% to 15%) furnished 7.7 mg (96 %) of **12p** as a white solid: mp 113.7-115.0 °C; IR 3115, 3093, 2225, 1607 cm^{-1}; ^1H NMR (400 MHz, $CDCl_3$) δ 8.91 (d, J = 1.9 Hz, 1H), 8.09 – 8.00 (m, 2H), 7.75 – 7.71 (m, 2H), 7.76-7.69 (m, 1H); $^{13}C\{1H\}$ NMR (101

MHz, $CDCl_3$) δ 154.4, 153.5, 138.2, 132.7, 126.9, 118.8, 115.4, 111.6; HRMS (ESI) m/z [$M+H^+$] Calcd. for $C_{10}H_7N_2S$ 187.0330, found 187.0336.

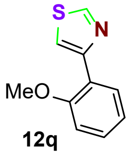
12q

4-(2-Methoxyphenyl)thiazole ***(12q)***. Following the general thiazole synthesis procedure A with pure (*Z*)-1-(2-bromo-1-isocyanovinyl)-2-methoxybenzene (**10q**, 35.2 mg, 0.15 mmol), NaSH (15.0 mg, 0.27 mmol), Cs_2CO_3 (72.3 mg, 0.22 mmol), and purification by MPLC (4 g silica gel cartridge, eluting with EtOAc/Hexanes 10% to 15%) furnished 21.2 mg (75 %) of **12q** as a clear, colorless oil: IR 3145, 3064, 2938,2835 cm^{-1}; ^1H NMR (400 MHz, $CDCl_3$) δ 8.84 (d, J = 2.0 Hz, 1H), 8.27 (dd, J = 7.3, 1.8 Hz, 1H), 8.00 (d, J = 2.0 Hz, 1H), 7.33 (ddd, J = 8.3, 7.3, 1.8 Hz, 1H), 7.12 – 7.06 (m, 1H), 7.02 (d, J = 8.3 Hz, 1H), 3.96 (s, 3H); $^{13}C\{1H\}$ NMR (101 MHz, $CDCl_3$) δ 156.7, 151.9, 150.8, 130.0, 129.0, 123.0, 120.9, 116.7, 111.1, 55.5; HRMS (ESI) m/z [$M+H^+$] Calcd. for $C_{10}H_{10}NOS$ 191.0483, found 191.0488.

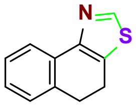
12r

4,5-Dihydronaphtho[2,1-d]thiazole ***(12r)***. Following the general thiazole synthesis procedure A with 4-bromo-3-isocyano-1,2-dihydronaphthalene (**10r**, 54.5 mg, 0.23 mmol), NaSH (23.5 mg, 0.42 mmol), Cs_2CO_3 (113.8 mg, 0.35 mmol) and purification by MPLC (4 g silica gel cartridge, eluting with EtOAc/Hexanes 10% to 15%) furnished 29.3 mg (67 %) of thiazole **12r** as clear, colorless oil: IR (ATR) 3059, 2935, 2892, 2836 cm^{-1}; ^1H NMR (400 MHz, $CDCl_3$) δ 8.68 (s, 1H), 7.99 – 7.94 (m, 1H), 7.35 – 7.29 (m, 1H), 7.25 – 7.19 (m, 2H), 3.07 (s, 4H); $^{13}C\{1H\}$ NMR (101 MHz, $CDCl_3$) δ 150.7, 150.4, 134.5, 131.4, 129.7, 127.9, 127.5, 127.2, 123.2, 29.0, 22.0; HRMS (+ESI) m/z [M+H] Calcd. for $C_{11}H_{19}NS$ 187.0456, found 187.0449.

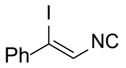

20a

(Z)-(1-Iodo-2-isocyanovinyl)benzene ***(20a).*** Solid iodine (172.5 mg, 0.68 mmol) was added to a -10 °C, CH_2Cl_2 solution (0.1M) of the (*E*)-*N*-styrylformamide[9] (50 mg, 0.34 mmol). After 1 h, triethylamine (0.14 mL, 1.02 mmol) was added and then the mixture was allowed to warm to rt. After 24 h, the reaction was cooled to 0 °C, neat *i*-$PrNEt_2$ (0.53 mL, 3.06 mmol) was then added followed by the dropwise addition of neat phosphoryl chloride (94 μL, 1.02 mmol). Upon completion, as monitored by TLC, the reaction mixture was poured into 0 °C, saturated, aqueous sodium carbonate, the phases were separated, and then the aqueous phase was extracted with CH_2Cl_2 (3 x 15 mL). The combined organic extract was washed sequentially with water, then brine, and then dried (Na_2SO_4), filtered, and concentrated. The crude product was purified by flash chromatography on C-2 silica[1] to afford pure *Z*-**20a** 45 mg (52%) as a white oil: IR (ATR) 3052, 2119, 1490, 1443 cm^{-1}; ^1H NMR (400 MHz, $CDCl_3$) δ 7.54 – 7.43 (m, 2H), 7.42 – 7.33 (m, 3H), 6.42 (s, 1H); $^{13}C\{1H\}$ NMR (101 MHz, $CDCl_3$): 169.4, 138.2, 130.4, 128.8, 128.6, 118.89 (t, J = 14.0 Hz), 111.4; HRMS (+EI) m/z $[M+H]^+$ Calcd. for C_9H_6IN 254.9529, found 254.9551.

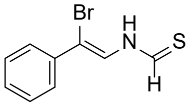

21a

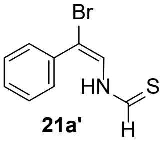

21a'

(Z)-N-(2-Bromo-2-phenylvinyl)methanethioamide ***(21a).*** Neat 2,4-bis(4-methoxyphenyl)-2,4-dithioxo-1,3,2,4-dithiadiphosphetane was added to a 7:1 *Z/E* mixture of *N*-(2-bromo-2-phenylvinyl)formamide (56 mg, 0.25 mmol) in 0.1 M THF at 50 °C. After 3 h, the reaction was allowed to cool, water was added, and then EtOAc. The phases were separated, and the aqueous phase was extracted with EtOAc (10 mL x 2). The combined organic layer was washed with H_2O (10 mL x 1), brine (10 mL x 1), dried with Na_2SO_4 and concentrated. Purification on silica gel with (10:90 EtOAc/Hexane) provided 40.6 mg (68%) of **21a** as a white solid: mp 79.0 - 80.2 °C consisting of 4.9:1 mixture of *Z:E*-**21a**: IR (ATR) 3335, 3057, 2828, 1635 cm^{-1}; HRMS

(+ESI) m/z [M+H] Calcd. for $C_9H_{10}BrNS$ 241.9639; found 241.9653. For *(Z)-N-(2-bromo-2-phenylvinyl)methanethioamide* (**21a**) the ^{1}H NMR spectrum (400 MHz, $CDCl_3$) showed the presence of two rotamers in a 5.4:1 ratio. For the major rotamer: ^{1}H NMR (400 MHz, $CDCl_3$) δ 9.44 (dd, *J* = 5.8, 0.9 Hz, 1H), 9.04 (br s, 1H), 8.32 (dd, *J* = 10.8, 0.9 Hz, 1H), 7.63 – 7.54 (m, 2H), 7.42 – 7.30 (m, 3H); ^{13}C{1H} NMR (101 MHz, CD, Cl_3) δ 185.7, 136.1, 129.2, 128.7, 127.3, 121.7, 114.8. For the minor isomer: ^{1}H NMR (400 MHz, $CDCl_3$) δ 9.56 (d, *J* = 14.0 Hz, 1H), 9.19 (br s, 1H), 7.54 – 7.49 (m, 2H), 7.45 – 7.31 (m, 4H); ^{13}C{1H} NMR (101 MHz, $CDCl_3$) δ 188.6, 135.9, 129.1, 128.8, 127.1, 125.9, 108.1. For *(E)-N-*(2-bromo-2-phenylvinyl)methanethioamide (**21a'**) the ^{1}H NMR spectrum (400 MHz, $CDCl_3$) showed the presence of two rotamers in a 4.7:1 ratio. For the major rotamer: ^{1}H NMR (400 MHz, $CDCl_3$) δ 9.16 (dd, *J* = 5.9, 0.9 Hz, 1H), 8.60 (br. s, 1H), 8.09 (dd, *J* = 10.9, 0.9 Hz, 1H), 7.51 – 7.35 (m, 5H); ^{13}C{1H} NMR (101 MHz, $CDCl_3$) δ 184.3, 135.9, 129.8, 129.4, 128.65, 127.8, 112.2. For the minor rotamer: ^{1}H NMR (400 MHz, $CDCl_3$) δ 9.33 (d, *J* = 14.1 Hz, 1H), 8.79 (br. s, 1H), 7.51 – 7.34 (m, 5H), 7.12 (d, *J* = 11.5 Hz, 1H); ^{13}C{1H} NMR (101 MHz, $CDCl_3$) δ 187.7, 135.2, 129.9, 129.5, 128.66, 123.9, 106.9.

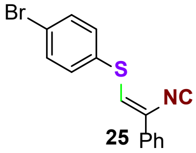
25

(Z)-(4-Bromophenyl)(2-isocyano-2-phenylvinyl)sulfane (25). A THF solution (0.7 M) of KHMDS (1.54 mL, 1.08 mmol) was added to a THF (0.1 M) solution of *β*-bromo-α-phenylisocyanoalkene **18j** (150 mg, 0.72 mmol) and 4-bromothiophenol (204.7 mg, 1.08 mmol) at 0 °C. After 2 h, water was added and then the crude reaction mixture was diluted with EtOAc. The phases were separated and then the aqueous phase was extracted with EtOAc (10 mL x 3). The combined organic phase was washed with water (10 mL x 1) and brine (10 mL), and then concentrated. The crude reaction mixture was purified on C-2 silica gel[1] (2% CH_2Cl_2/Hexanes) to provide 131 mg (57%) of **25** as a solid: mp 92.7 - 95 °C; IR

3064, 2109, 1474, 1386 cm^{-1}; ^{1}H NMR (400 MHz, $CDCl_3$) δ 7.58 – 7.48 (m, 4H), 7.44 – 7.33 (m, 5H), 6.97 (s, 1H); ^{13}C{1H} NMR (101 MHz, C_6D_6) δ 173.0, 132.3, 132.13, 132.11, 131.5, 128.7, 128.6, 127.1, 124.1, 122.9 (br), 122.2; HRMS (+EI) m/z [M$^+$] Calcd. for $C_{15}H_{10}BrNS$ 314.9717, found 314.9711.

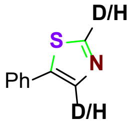

12s

5-Phenylthiazole-2,4-d2 ***(12s).*** Following the general thiazole synthesis procedure B with a 7:1 ratio of Z/*E*-bromo-2-isocyanovinyl benzene **10a** (21.2 mg, 0.10 mmol), NaSH (7.5 mg, 0.13 mmol), and *i*-Pr_2NEt (24 μL, 0.13mmol), but using MeOD as the solvent instead of EtOH, and purification by MPLC (4 g silica gel cartridge, eluting with EtOAc/Hexanes 10% to 15 %) furnished 6.8 mg (41%) of **12s** as a yellow solid: mp 35-36.8 °C; IR 3063, 2925, 1601, 1509 cm^{-1}; ^{1}H NMR (400 MHz, $CDCl_3$) δ 8.75 (s, 0.15H), 8.08 (s, 0.15H), 7.63 – 7.55 (m, 2H), 7.47 – 7.40 (m, 2H), 7.39 – 7.32 (m, 1H); ^{13}C{1H} NMR (101 MHz, $CDCl_3$) δ 153-1 (m), 139.2, 140-138 (m), 131.1, 129.1, 128.4, 127.0; HRMS (+ESI) m/z [M+H] Calcd for $C_9H_5D_2BrNS$ 164.0503, found 164.0499.

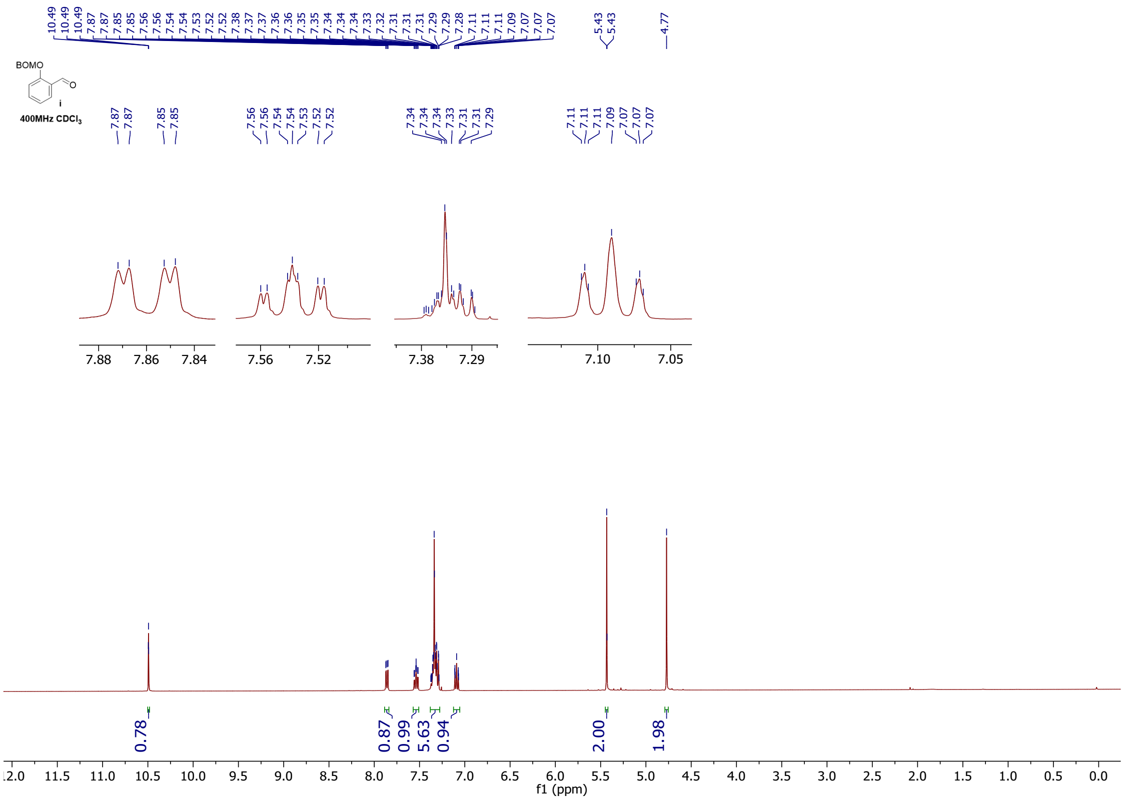

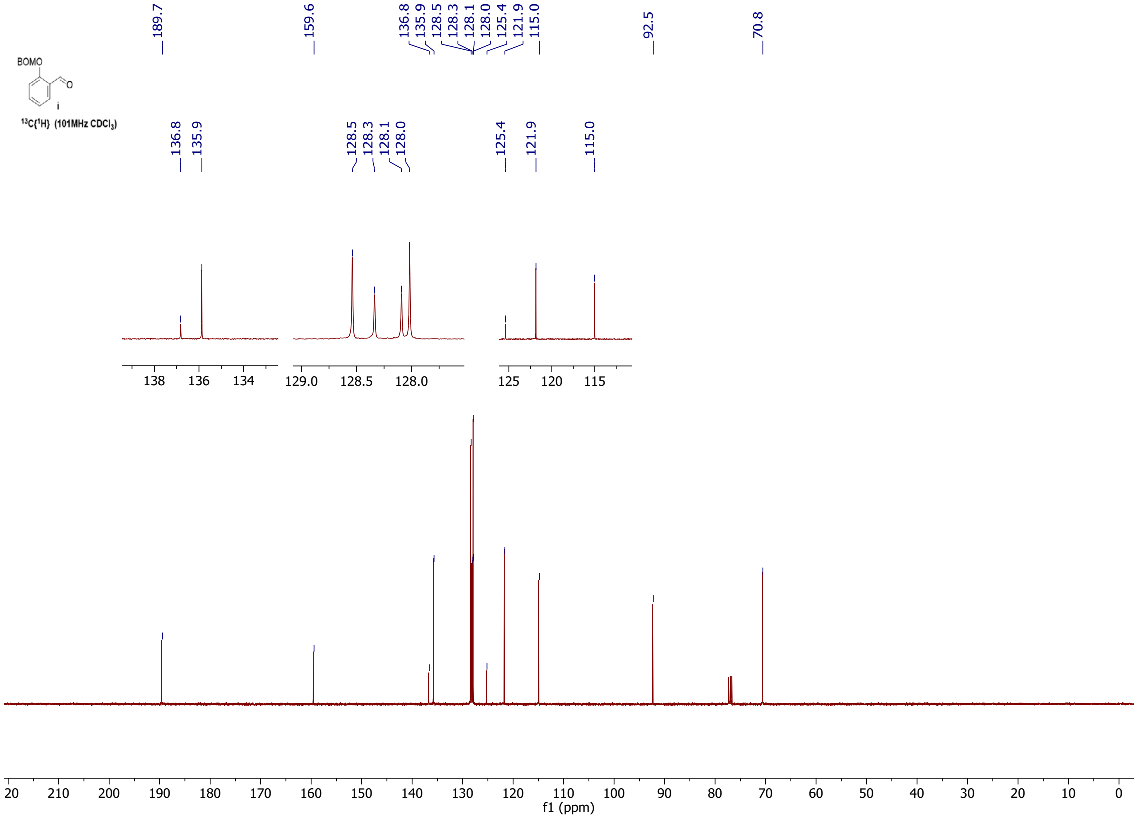

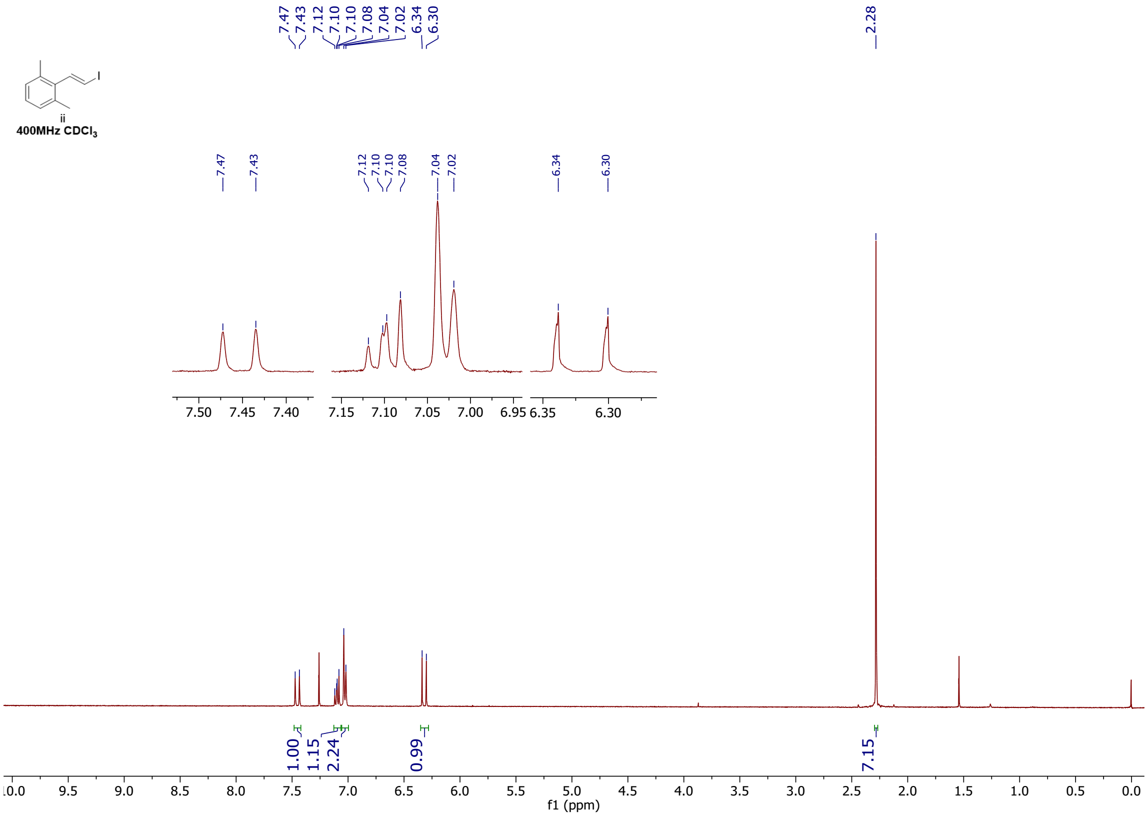
ii
400MHz $CDCl_3$
7.47 7.43 7.12 7.10 7.10 7.08 7.04 7.02 6.34 6.30
2.28
1.00 1.15 2.24 0.99 7.15
f1 (ppm)

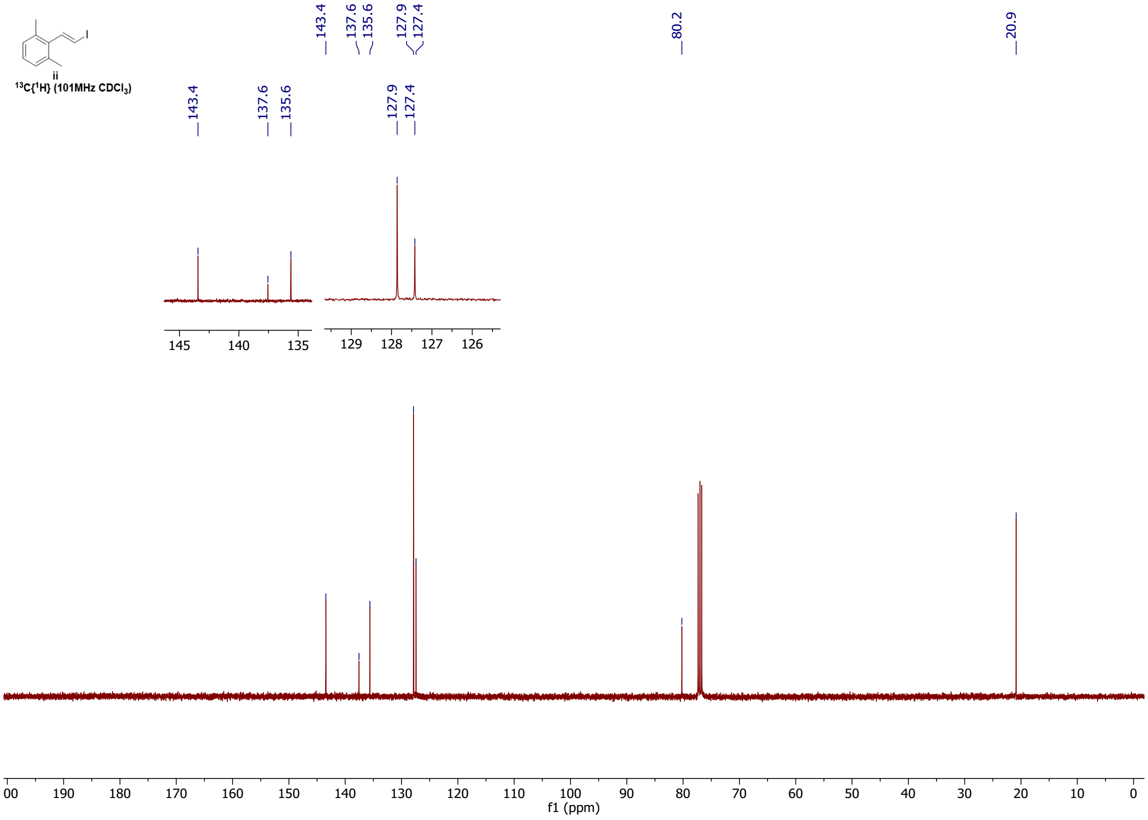
ii
$^{13}C\{^1H\}$ (101MHz $CDCl_3$)
143.4 137.6 135.6 127.9 127.4
80.2
20.9
f1 (ppm)

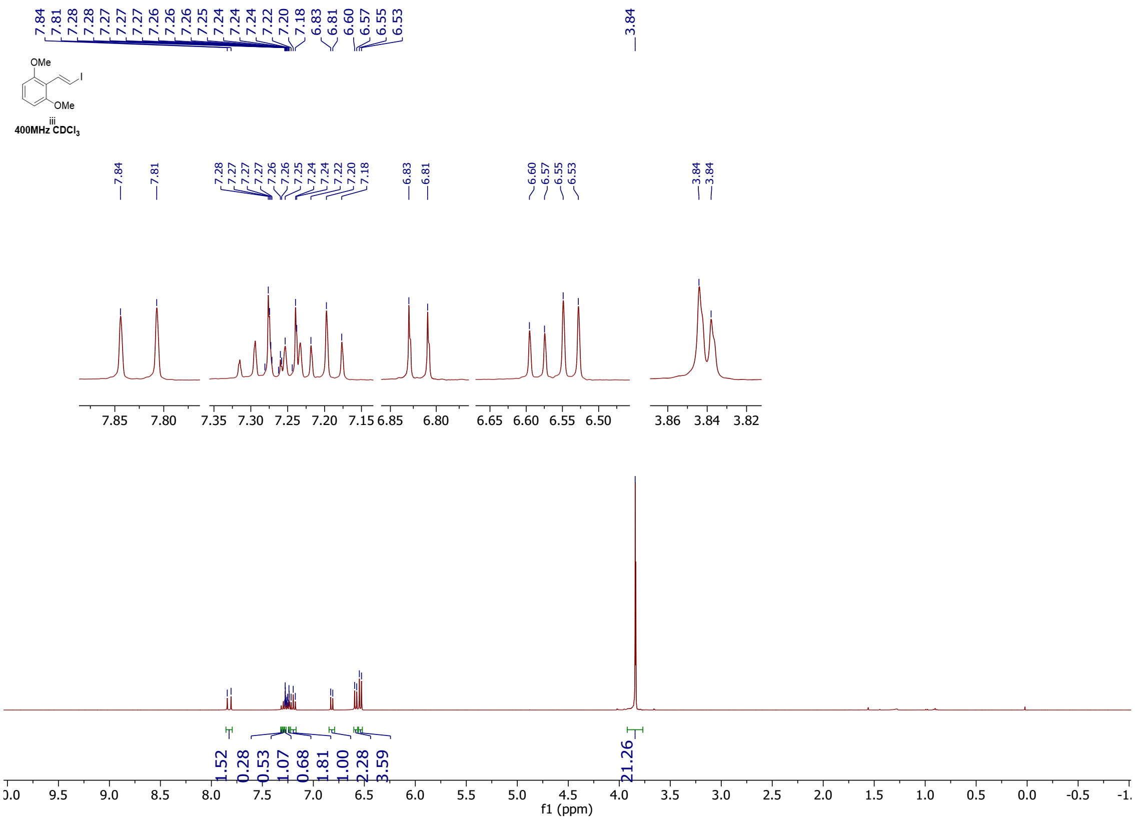
OMe
I
OMe
iii
400MHz CDCl3
f1 (ppm)

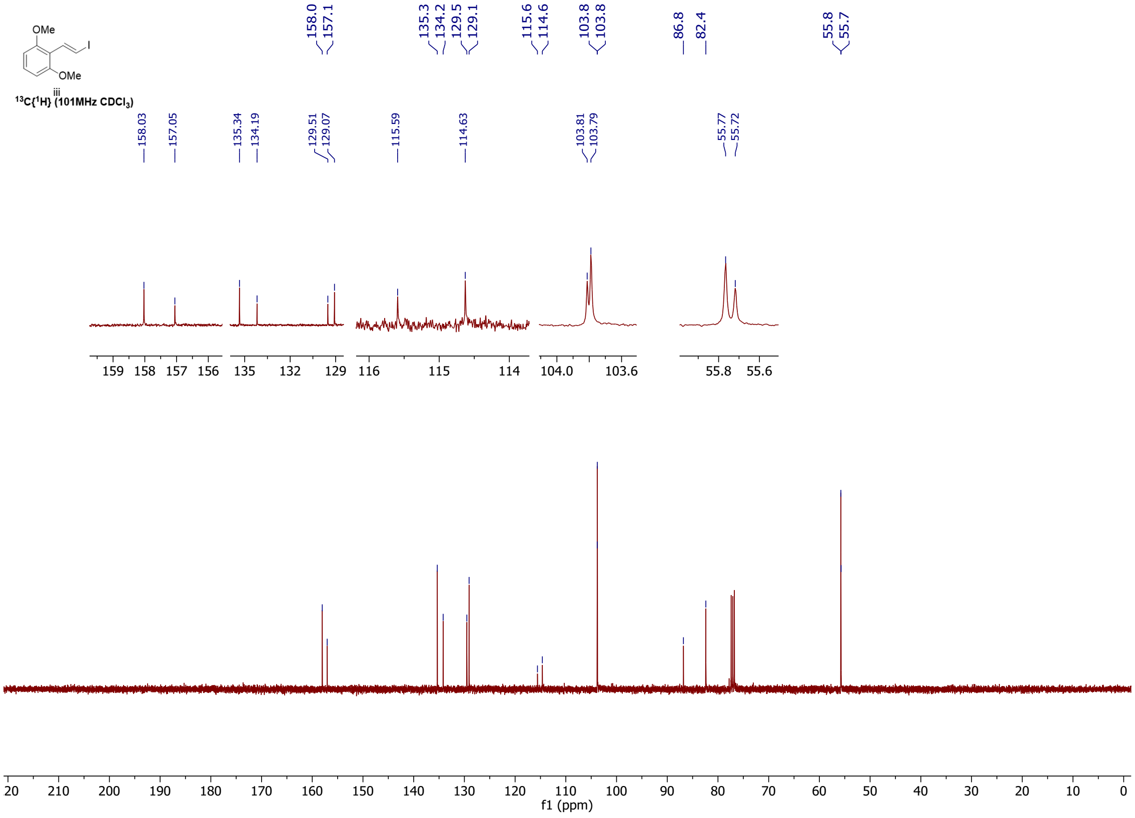
OMe
I
OMe
iii
13C{1H} (101MHz CDCl3)
f1 (ppm)

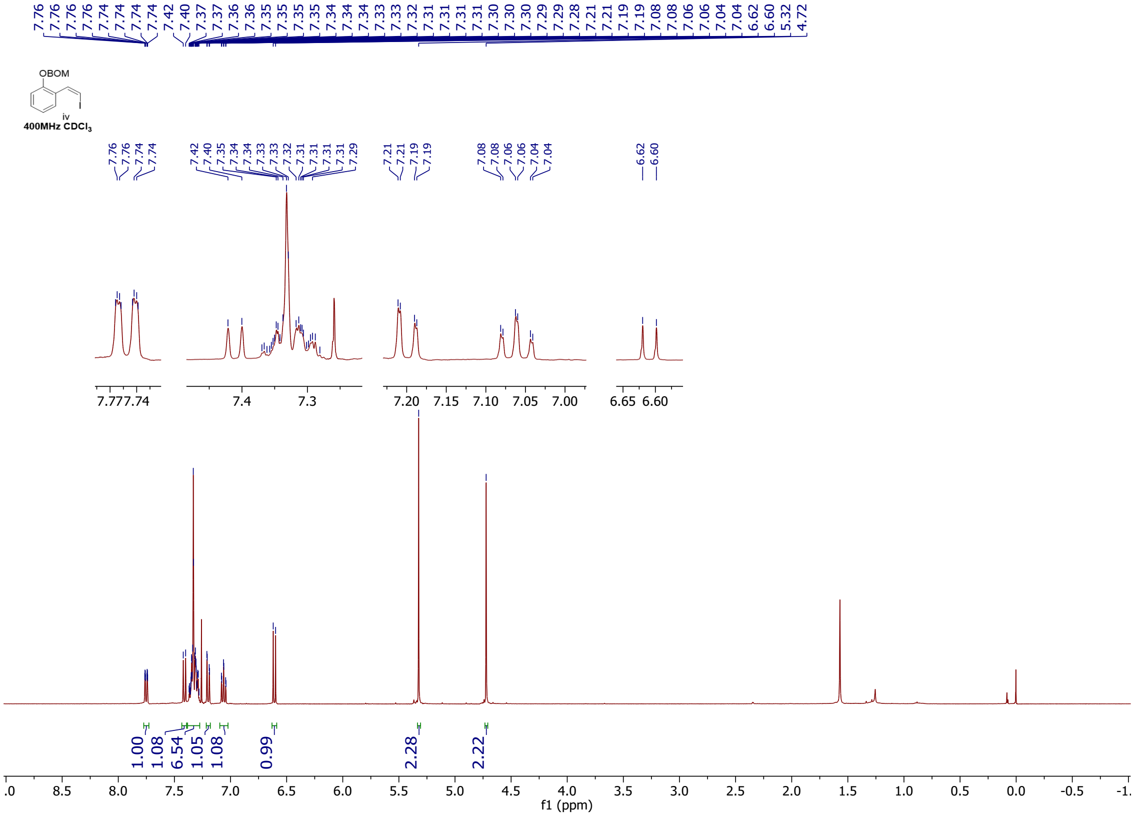
OBOM
iv
400MHz CDCl3
7.76 7.76 7.76 7.74 7.74 7.74 7.74 7.42 7.40 7.37 7.37 7.36 7.35 7.35 7.35 7.34 7.34 7.34 7.33 7.33 7.32 7.31 7.31 7.31 7.31 7.30 7.30 7.30 7.29 7.29 7.28 7.21 7.21 7.19 7.19 7.08 7.08 7.06 7.06 7.04 7.04 6.62 6.60 5.32 4.72
1.00
1.08
6.54
1.05
1.08
0.99
2.28
2.22
f1 (ppm)

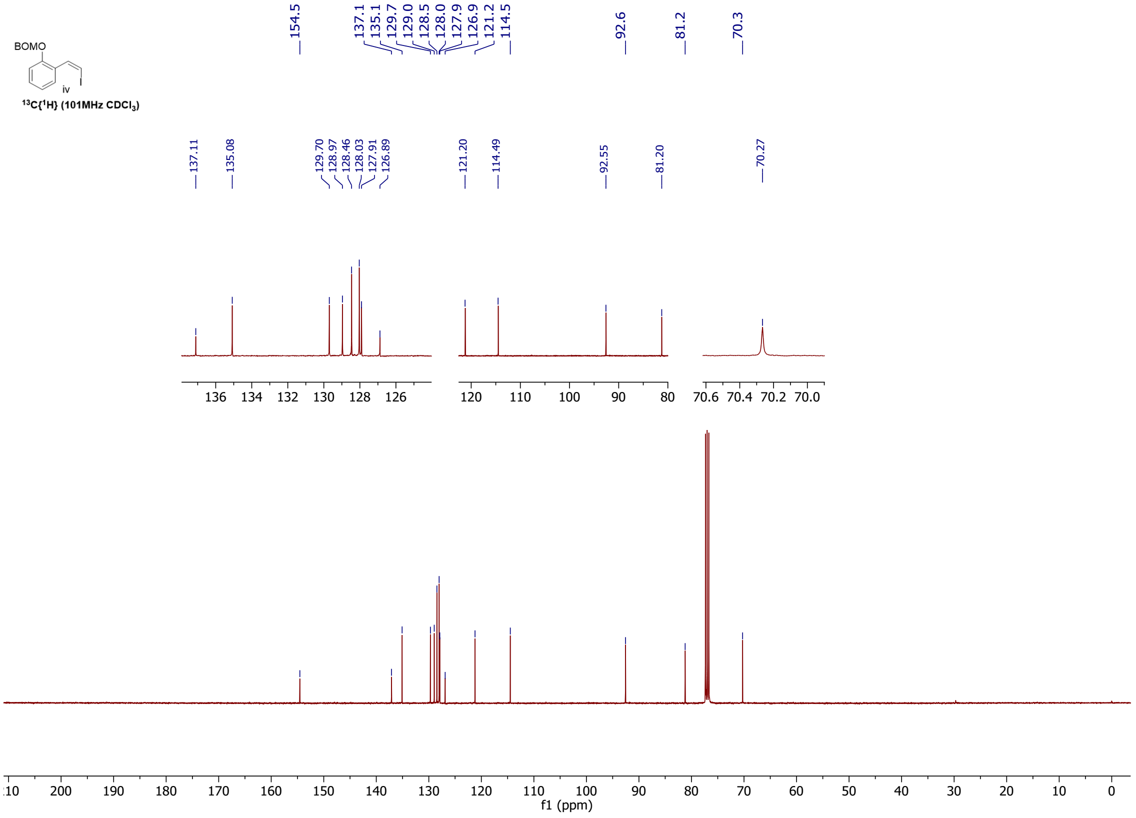
BOMO
iv
13C{1H} (101MHz CDCl3)
154.5 137.1 135.1 129.7 129.0 128.5 128.0 127.9 126.9 121.2 114.5 92.6 81.2 70.3
137.11 135.08 129.70 128.97 128.46 128.03 127.91 126.89 121.20 114.49 92.55 81.20 70.27
f1 (ppm)

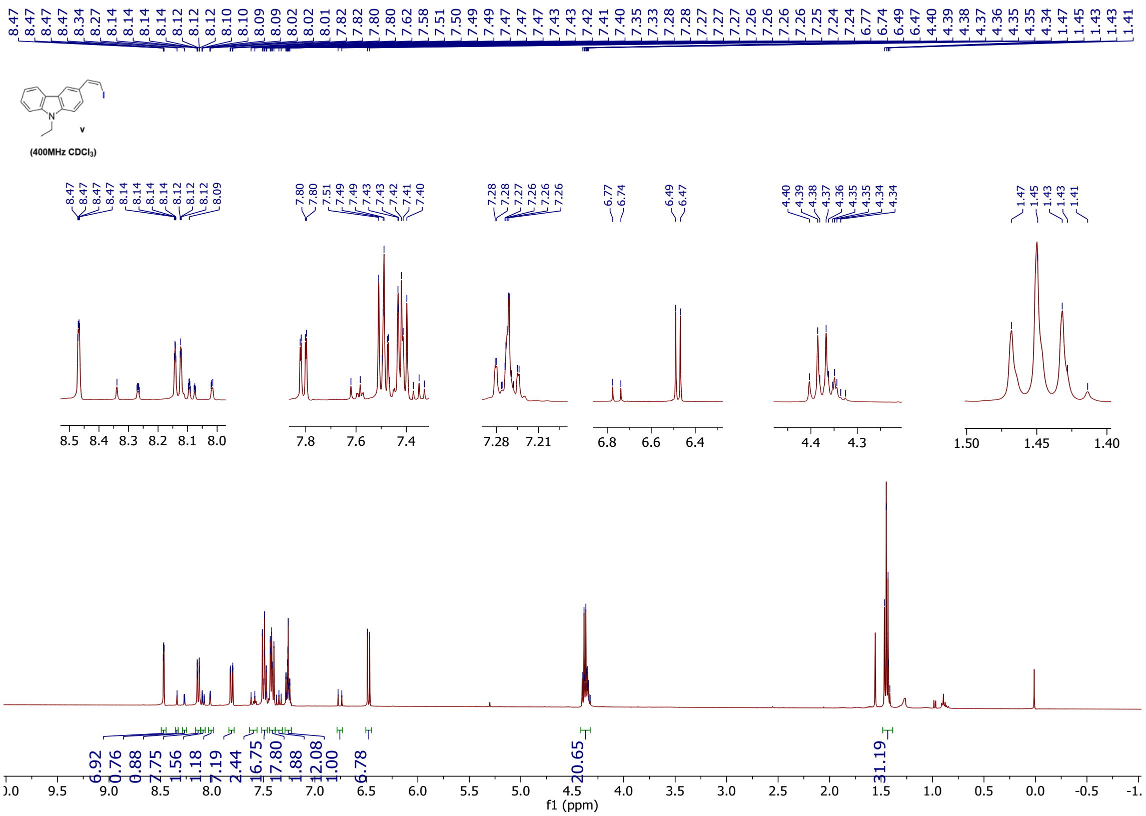
v
(400MHz CDCl3)
f1 (ppm)

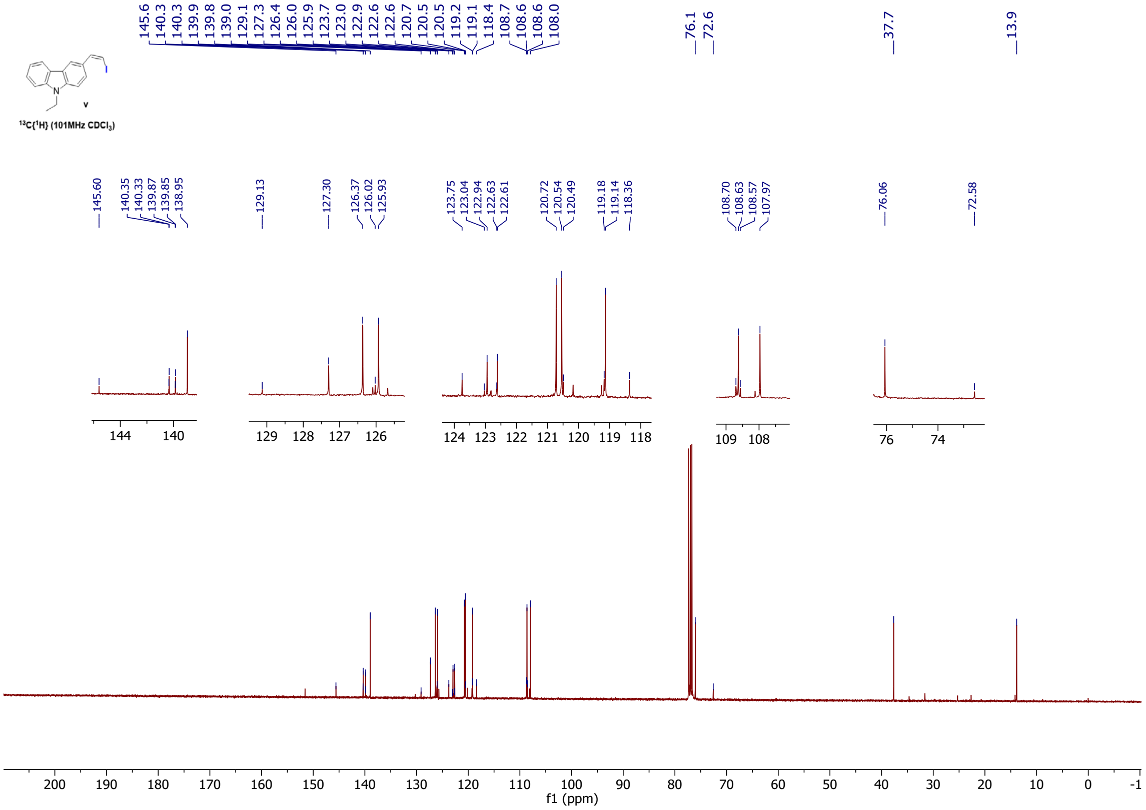
v
13C{1H} (101MHz CDCl3)
f1 (ppm)

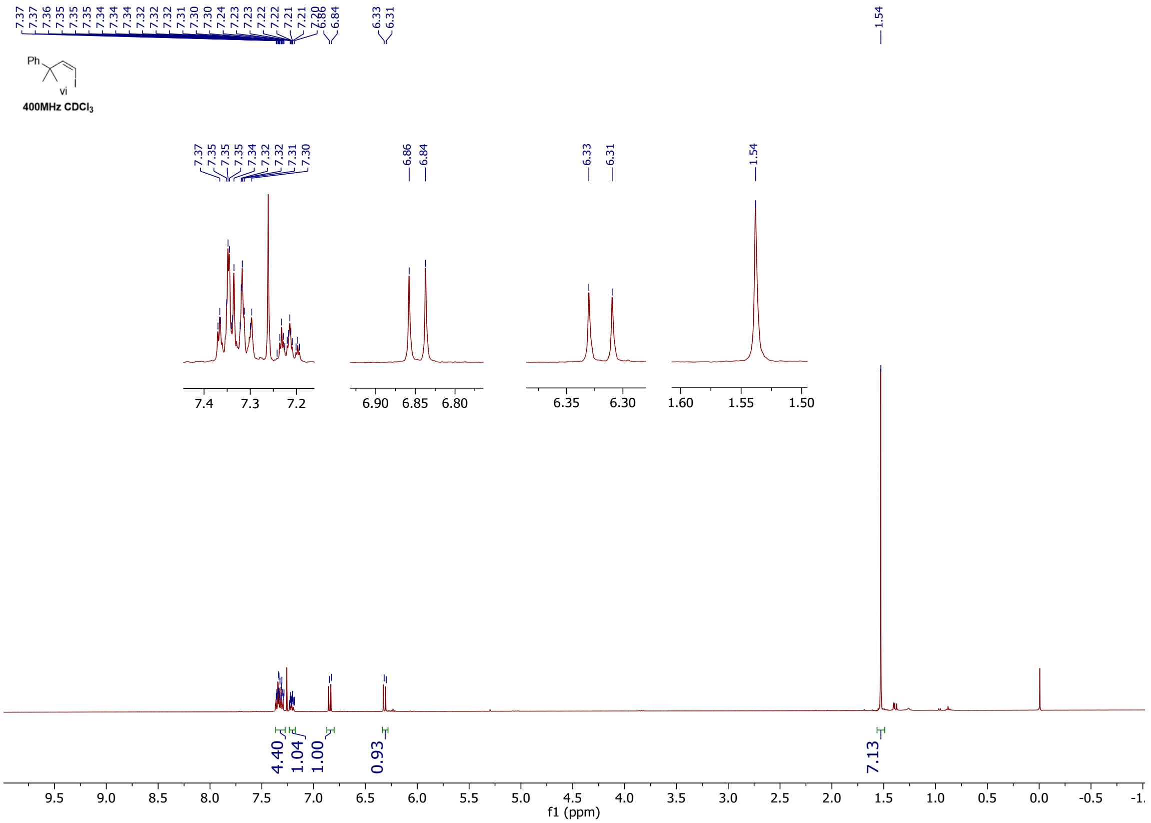
Ph
vi
400MHz CDCl3
4.40
1.04
1.00
0.93
7.13
f1 (ppm)

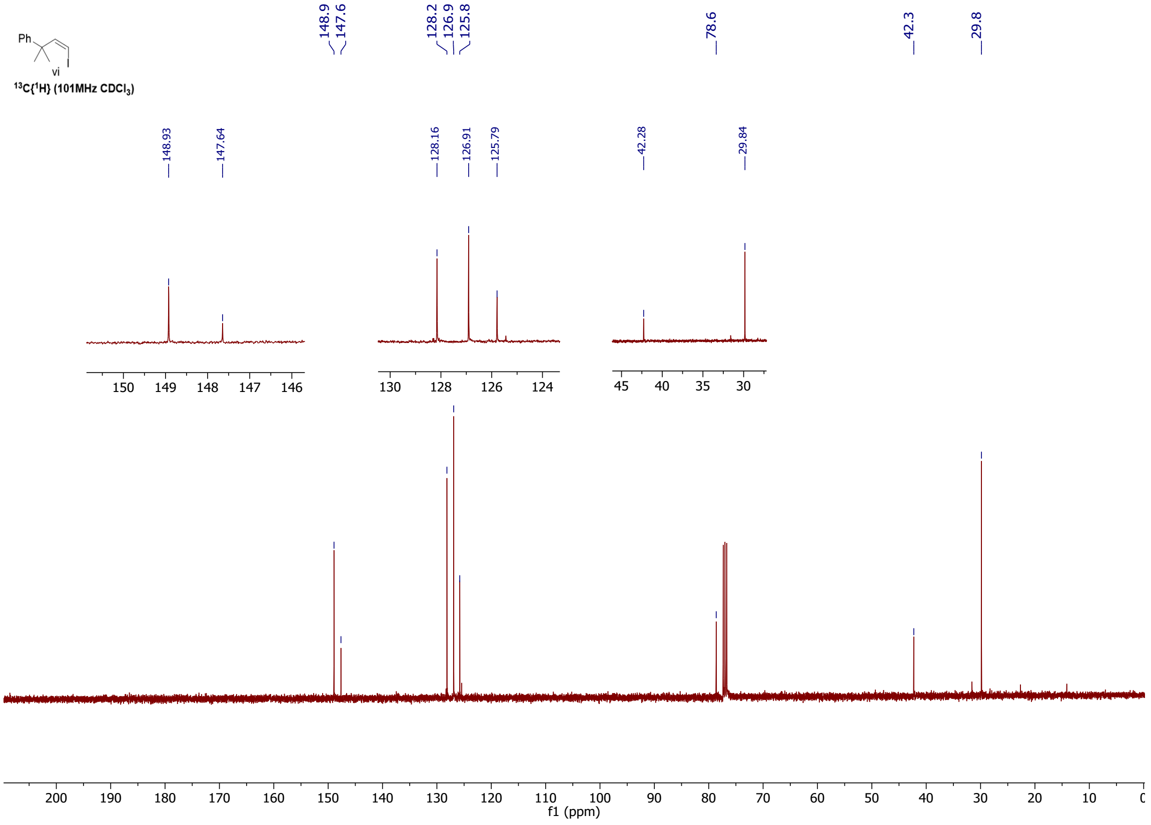
Ph
vi
13C{1H} (101MHz CDCl3)
148.9
147.6
128.2
126.9
125.8
78.6
42.3
29.8
f1 (ppm)

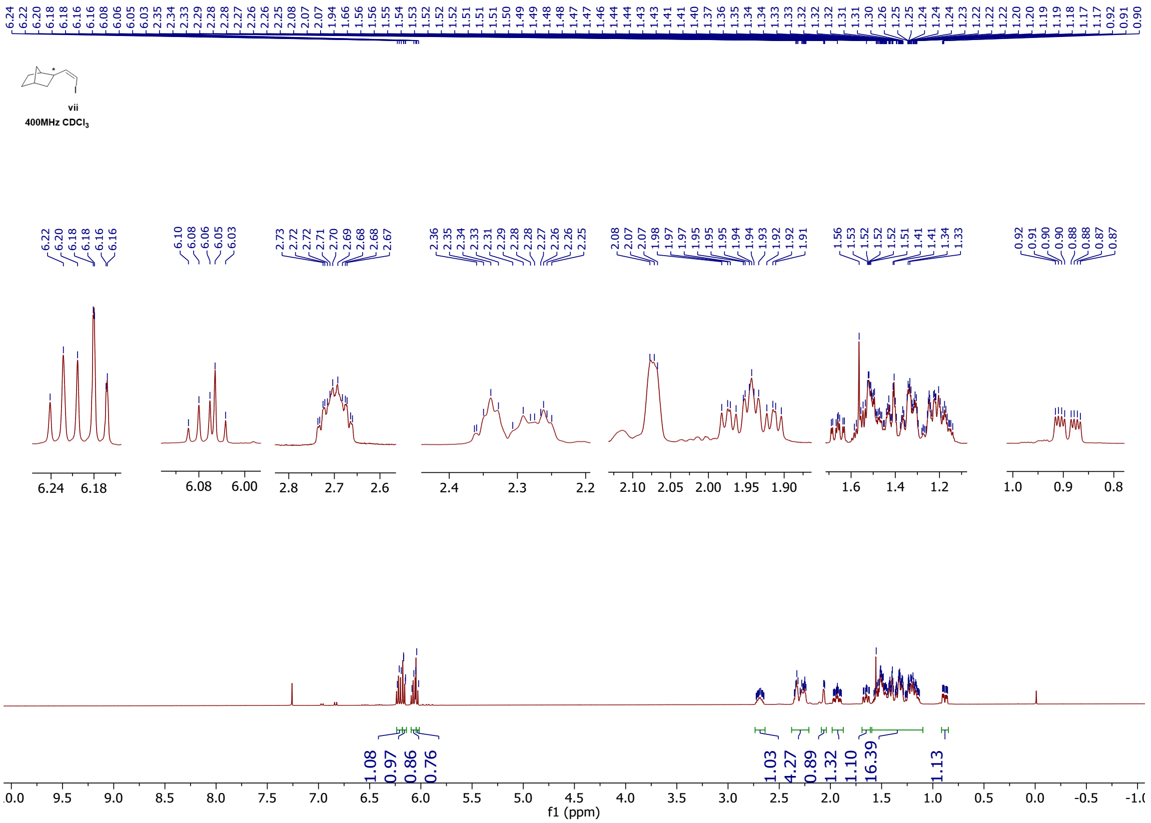

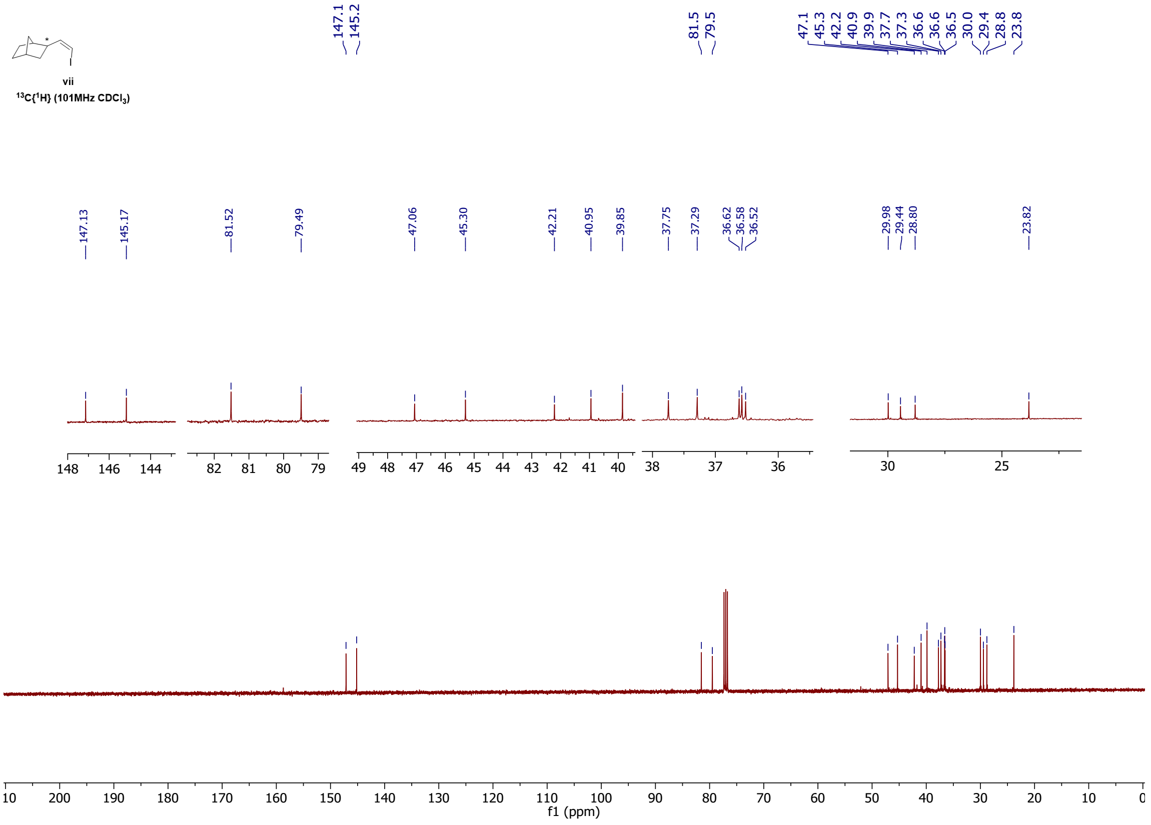

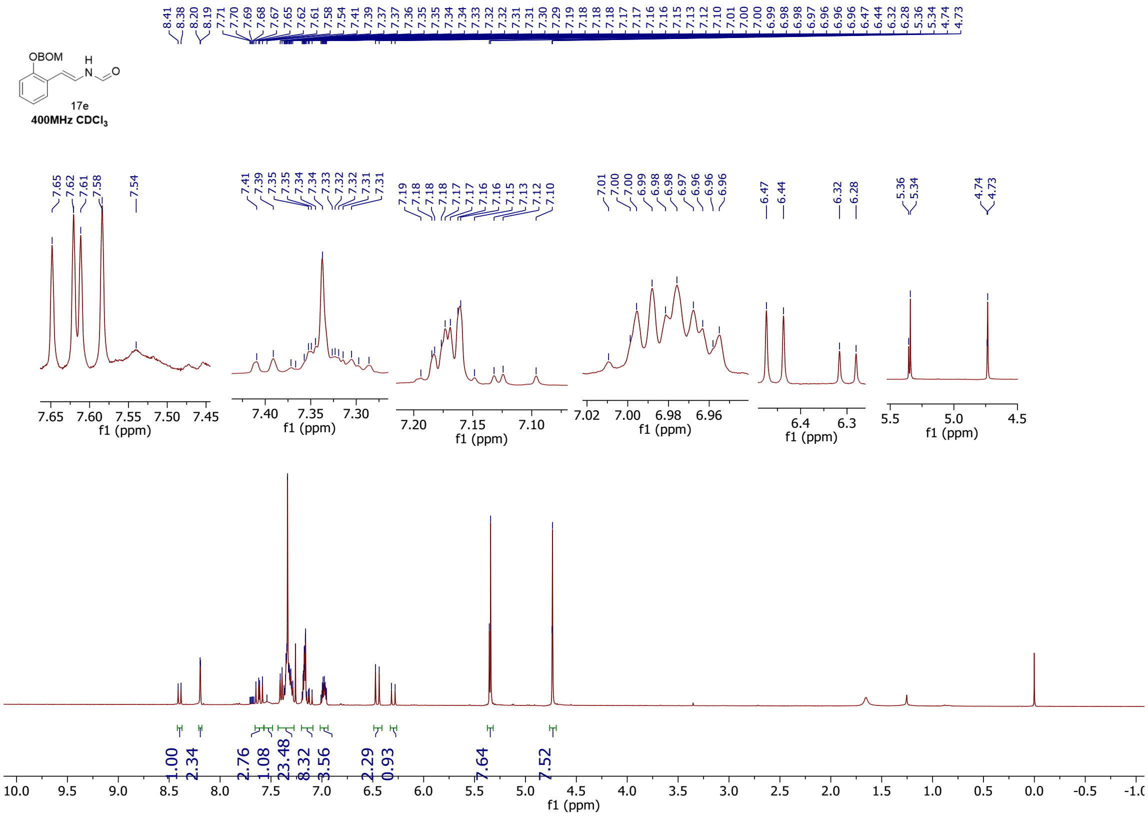
OBOM
17e
400MHz $CDCl_3$
f1 (ppm)

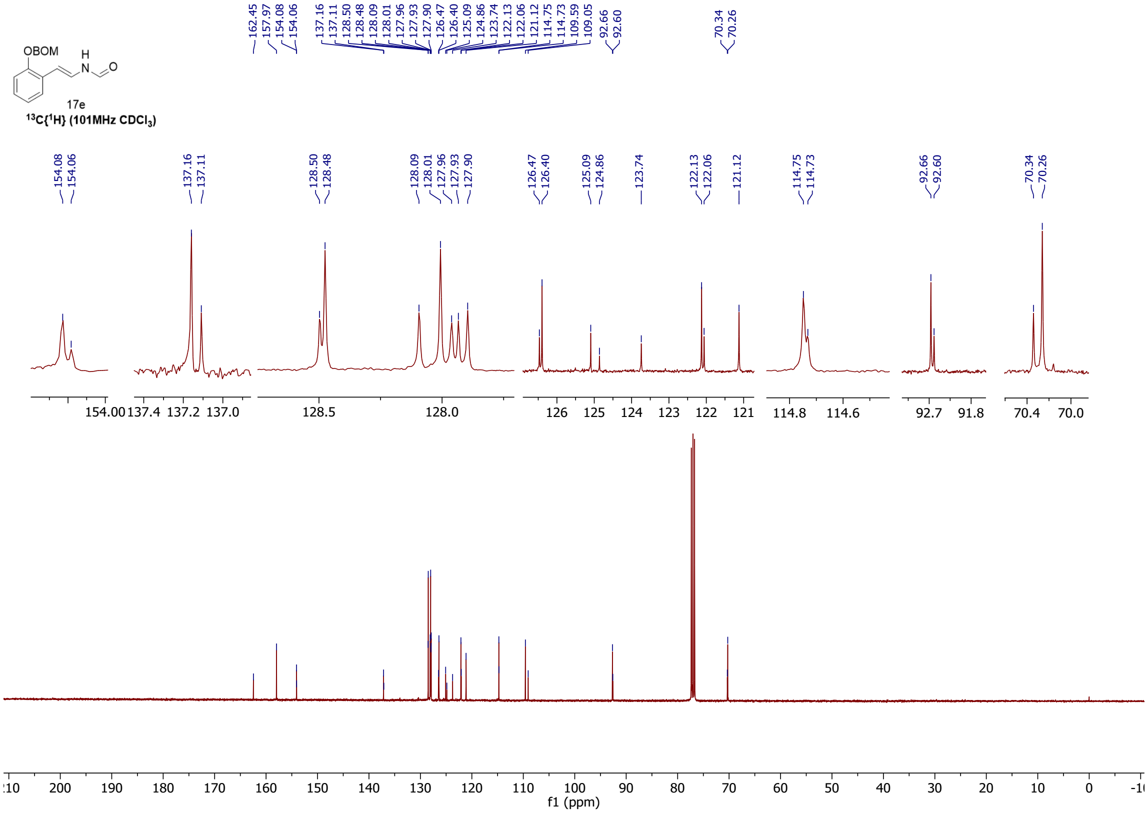
OBOM
17e
$^{13}C\{^1H\}$ (101MHz $CDCl_3$)
f1 (ppm)

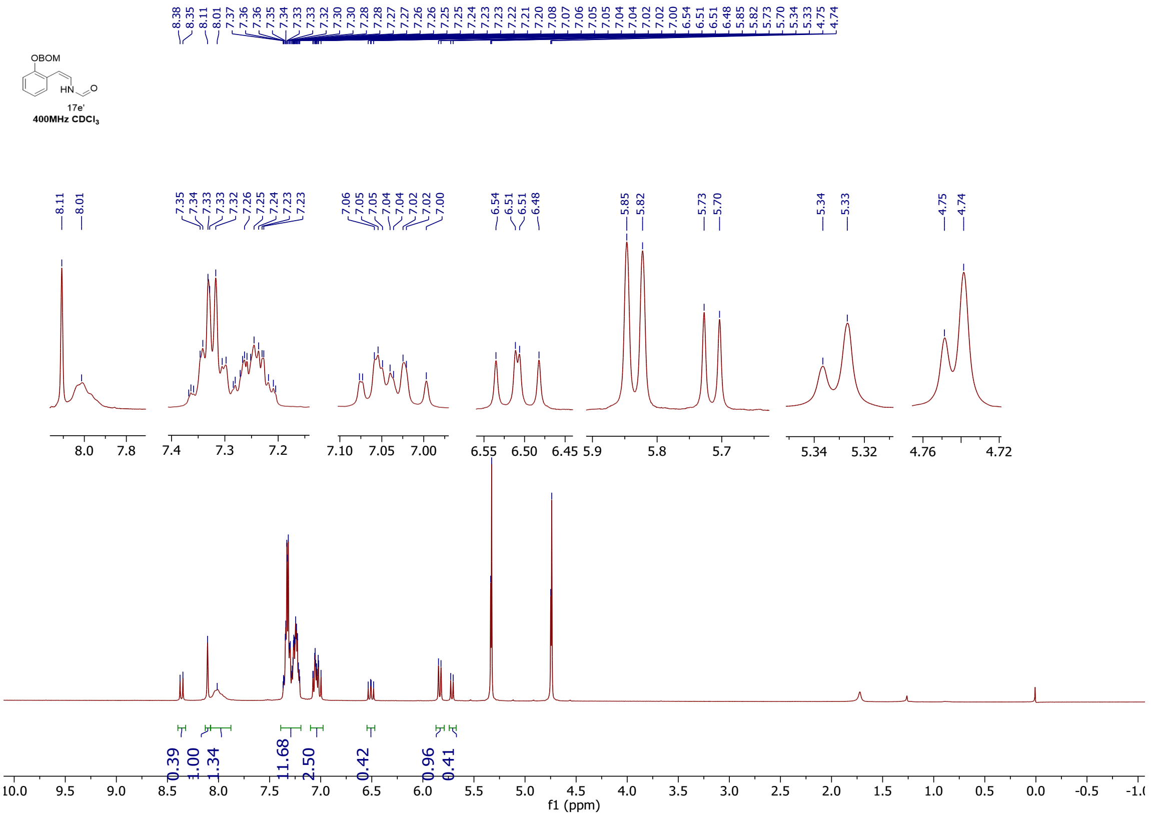
OBOM
HN
O
17e'
400MHz CDCl3
f1 (ppm)

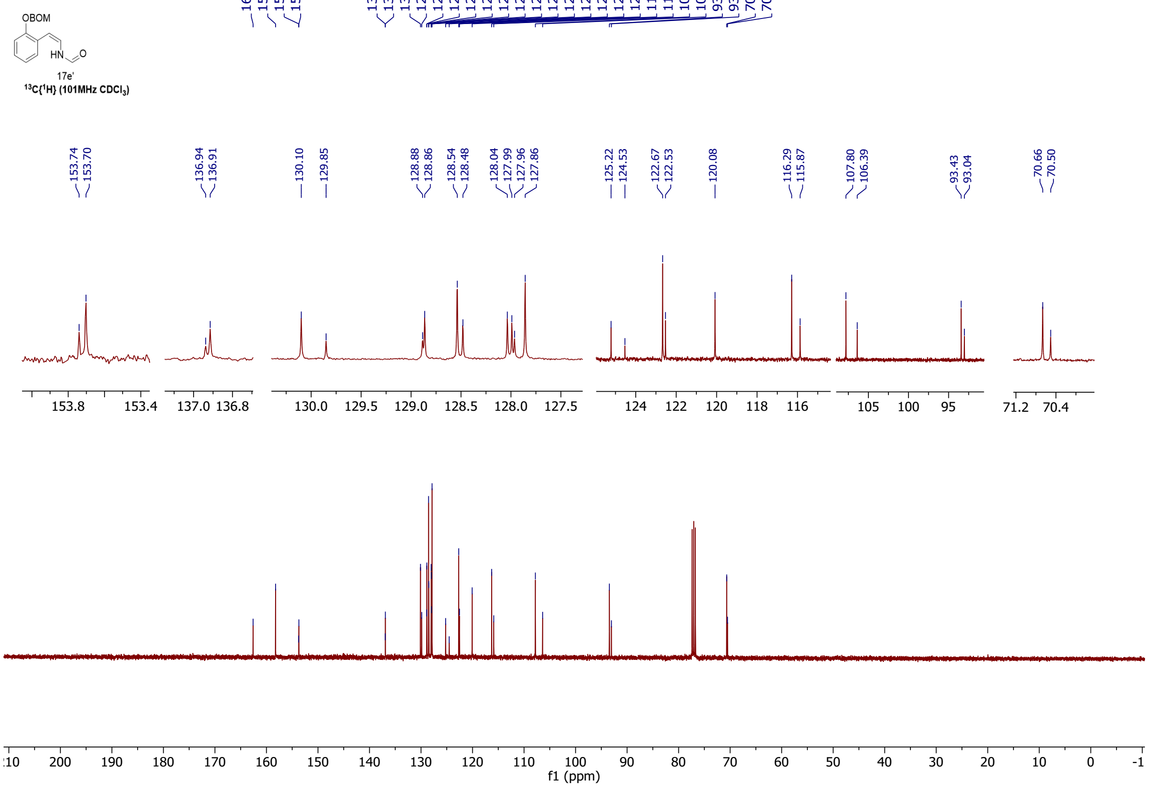
OBOM
HN
O
17e'
13C{1H} (101MHz CDCl3)
f1 (ppm)

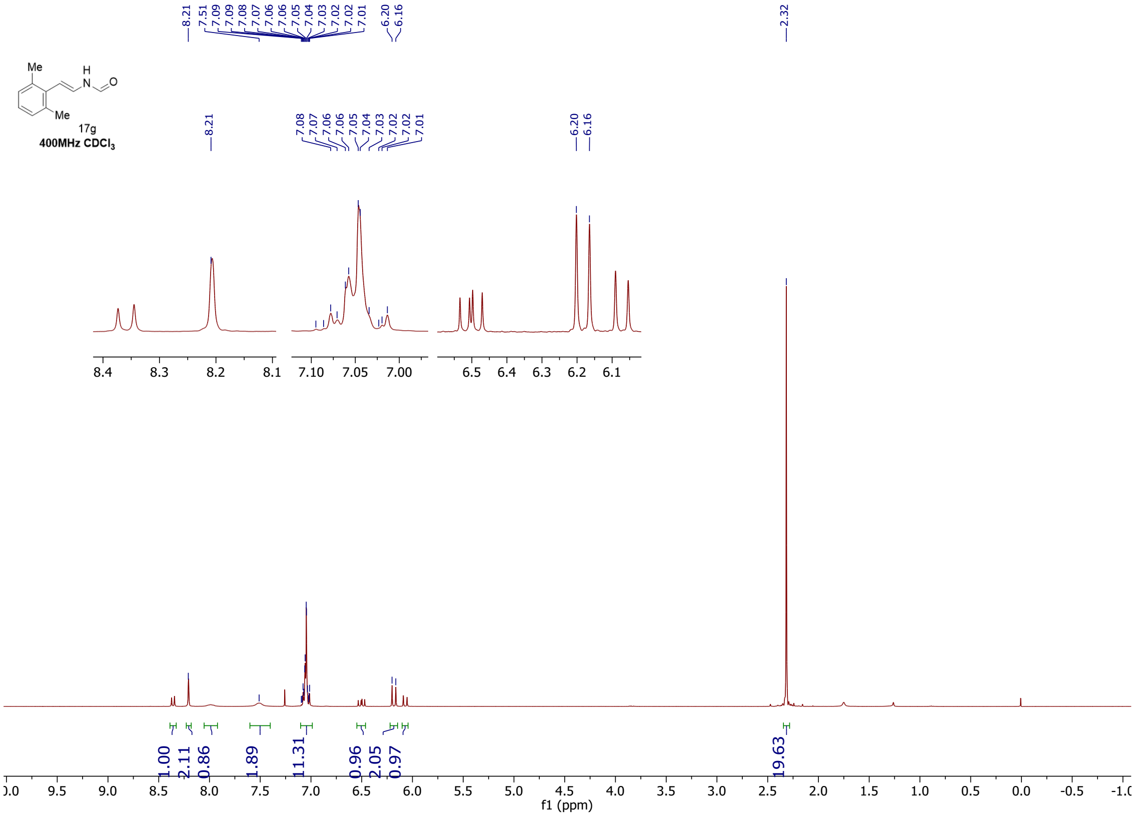
Me
H
N
O
Me
17g
400MHz CDCl3
f1 (ppm)

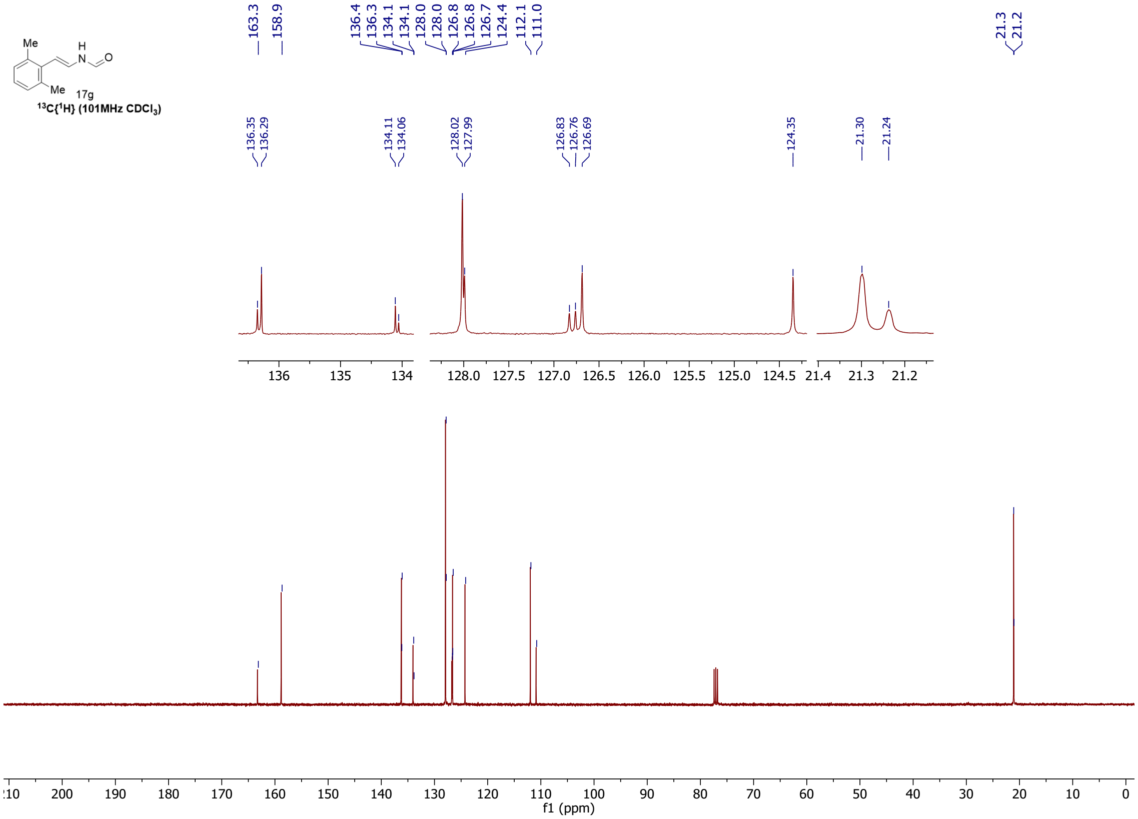
Me
H
N
O
Me
17g
13C{1H} (101MHz CDCl3)
f1 (ppm)

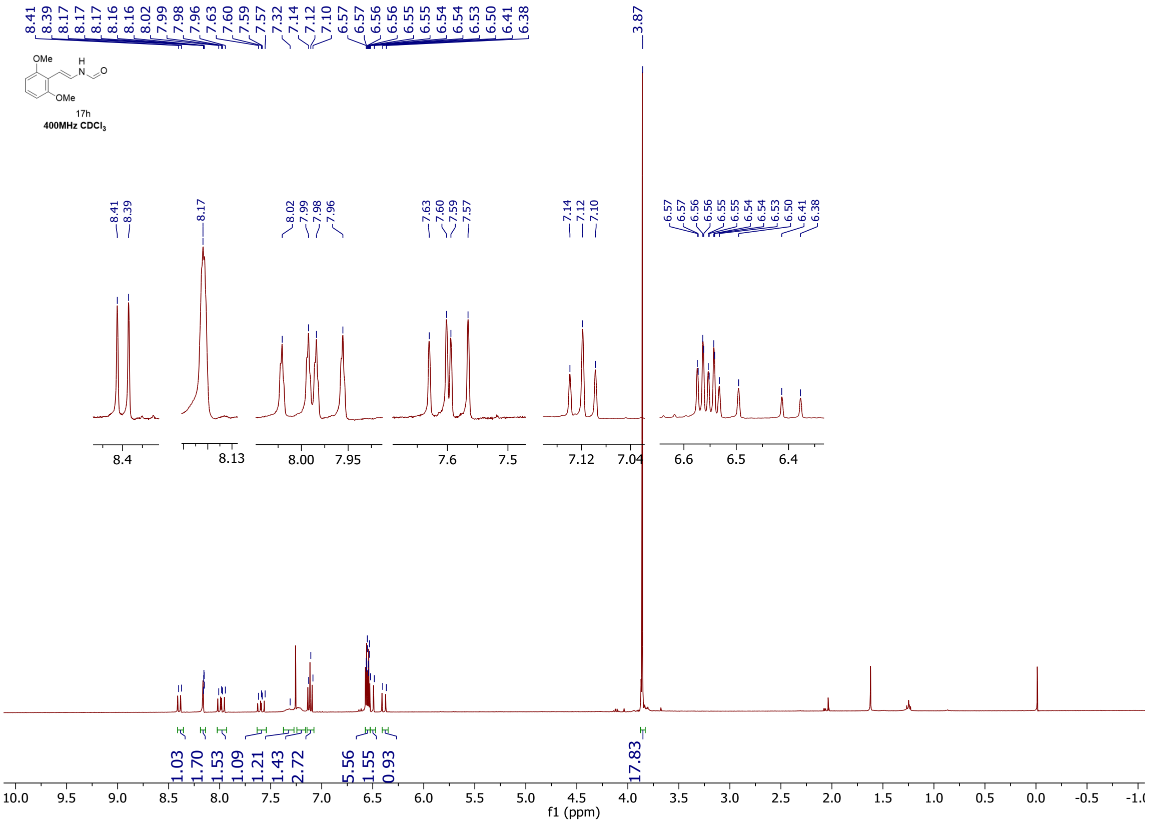
8.41
8.39
8.17
8.17
8.17
8.16
8.16
8.02
7.99
7.98
7.96
7.63
7.60
7.59
7.57
7.32
7.14
7.12
7.10
6.57
6.57
6.56
6.56
6.55
6.55
6.54
6.54
6.53
6.50
6.41
6.38
3.87
OMe
H
N
O
OMe
17h
400MHz CDCl3
1.03
1.70
1.53
1.09
1.21
1.43
2.72
5.56
1.55
0.93
17.83
f1 (ppm)

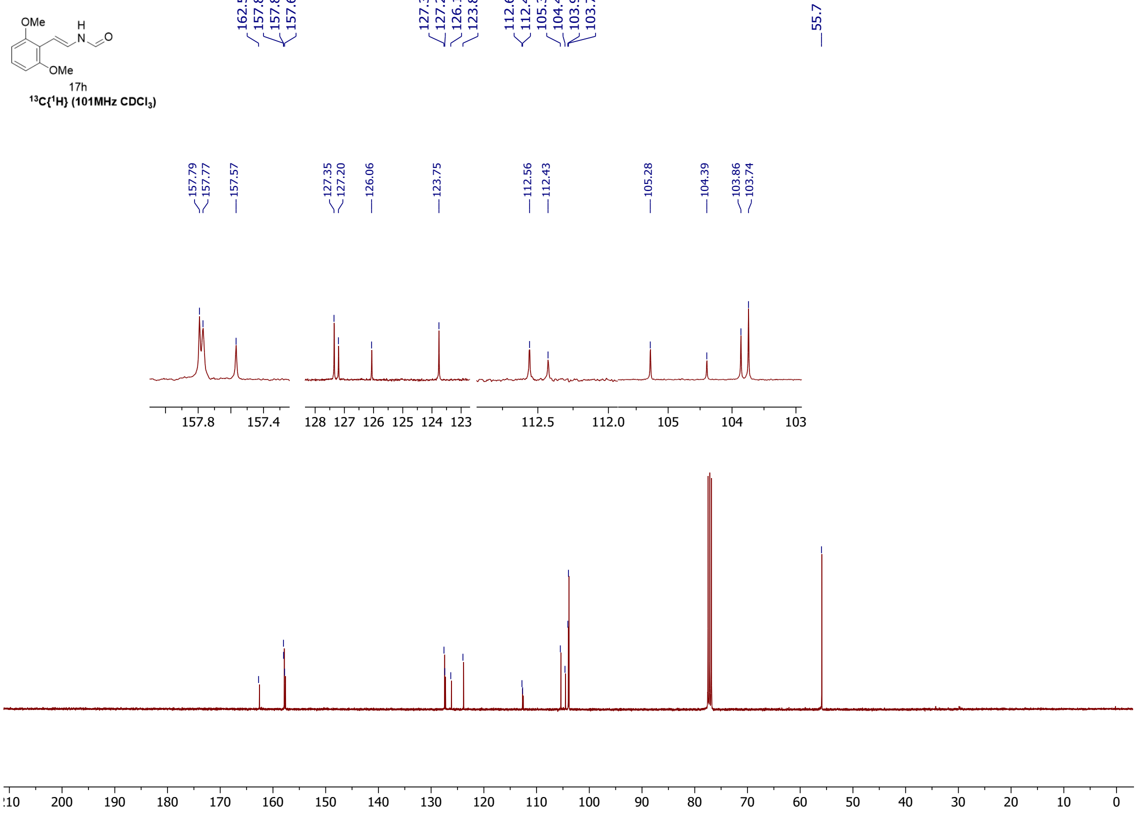
162.5
157.8
157.8
157.6
127.3
127.2
126.1
123.8
112.6
112.4
105.3
104.4
103.9
103.7
55.7
OMe
H
N
O
OMe
17h
13C{1H} (101MHz CDCl3)
157.79
157.77
157.57
127.35
127.20
126.06
123.75
112.56
112.43
105.28
104.39
103.86
103.74

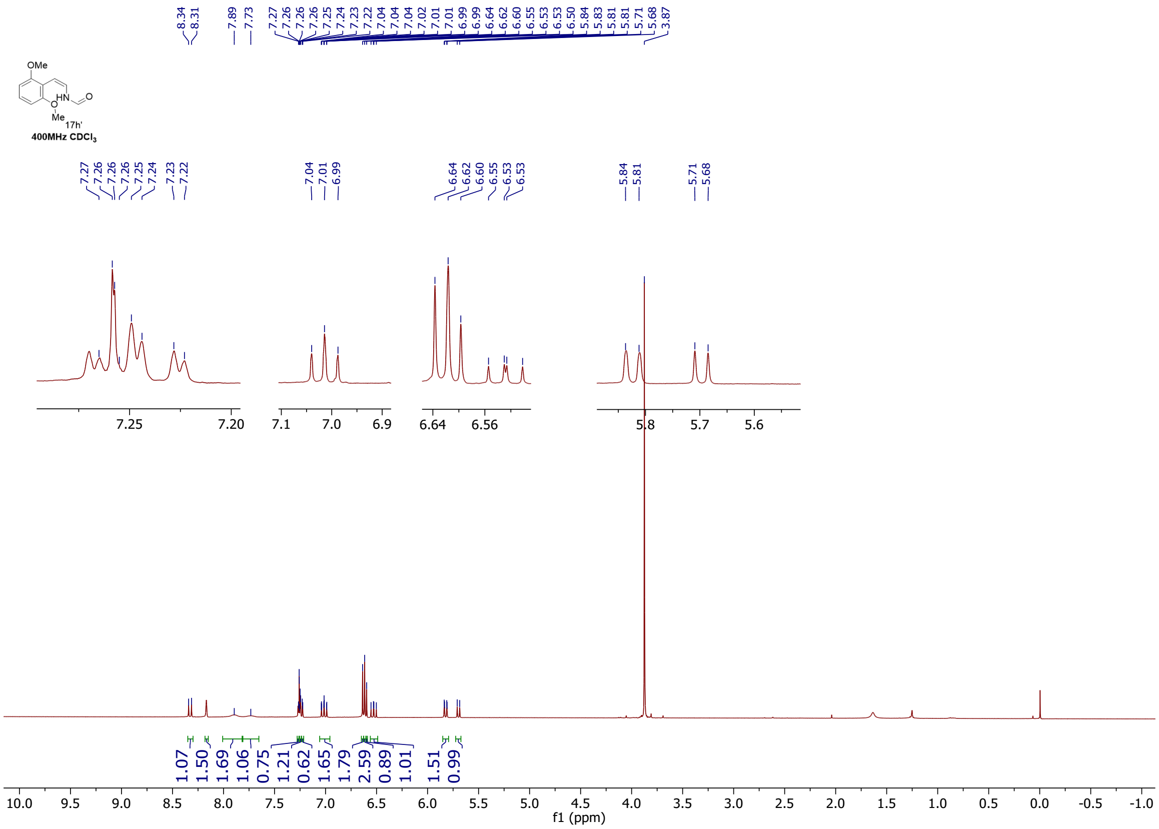

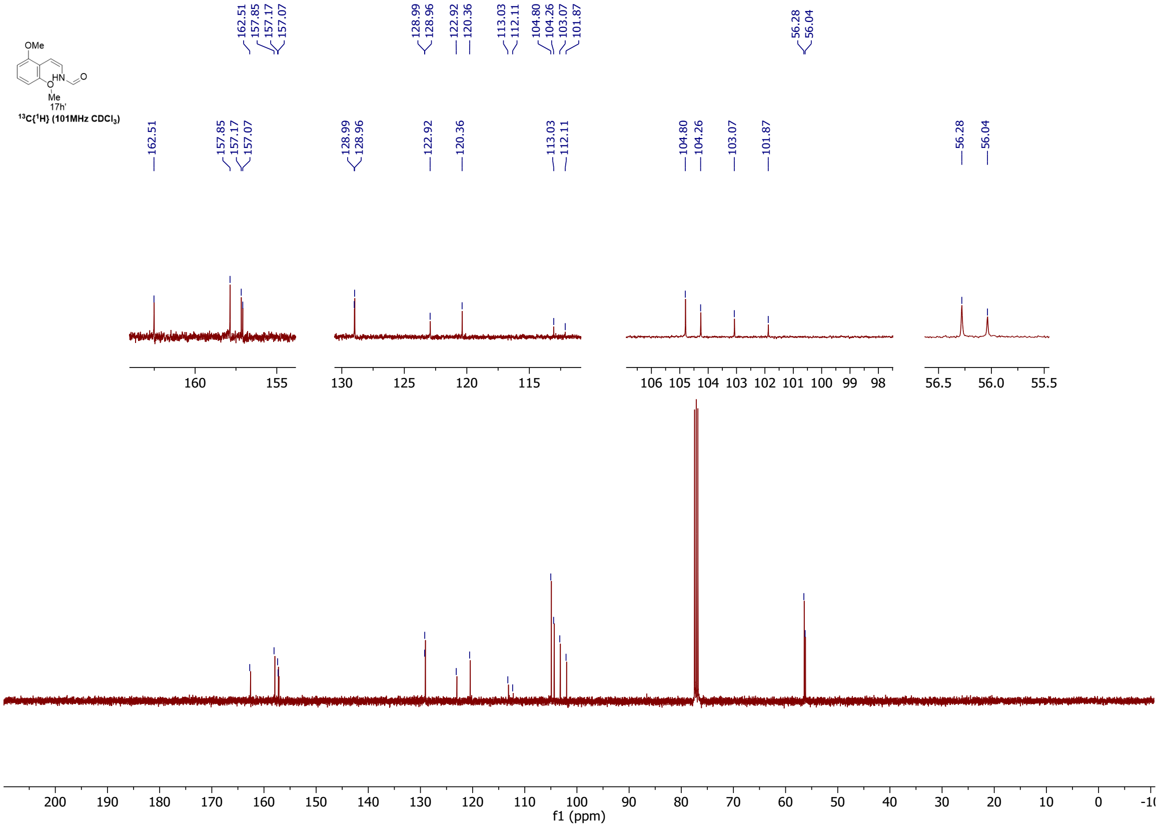

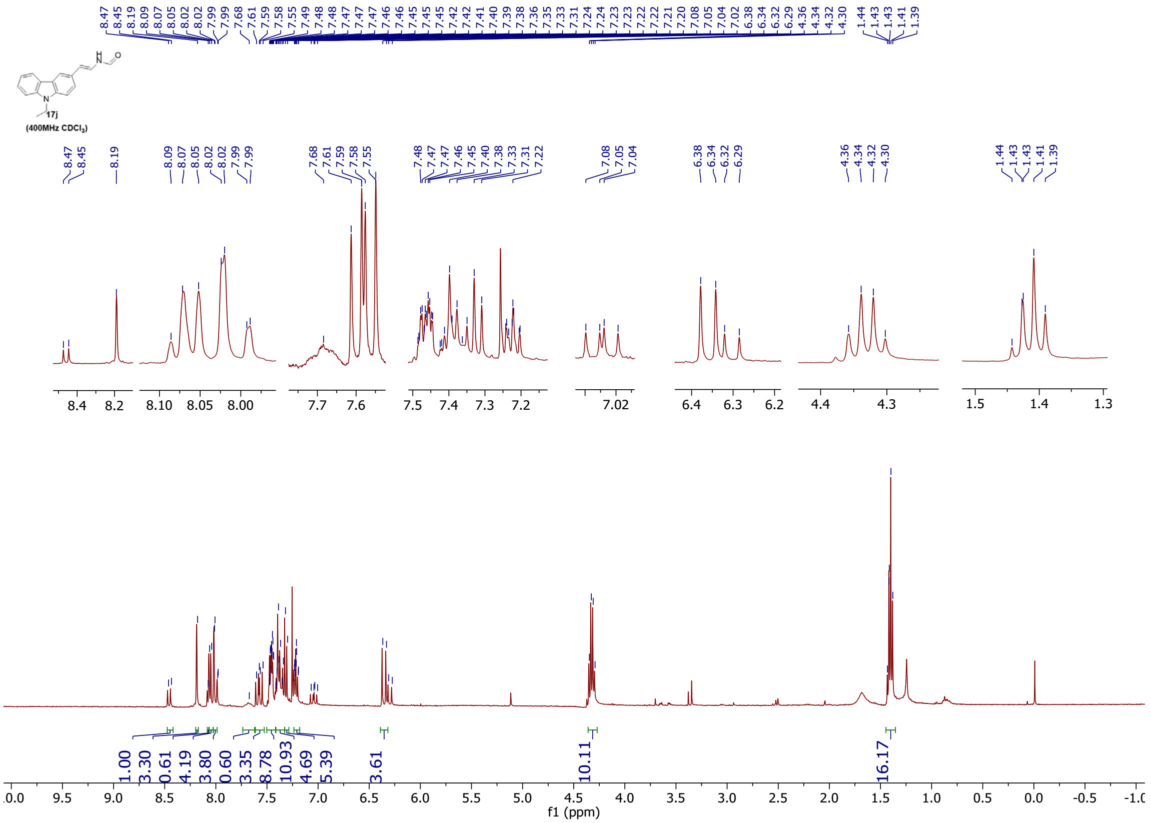

17j
(400MHz CDCl3)

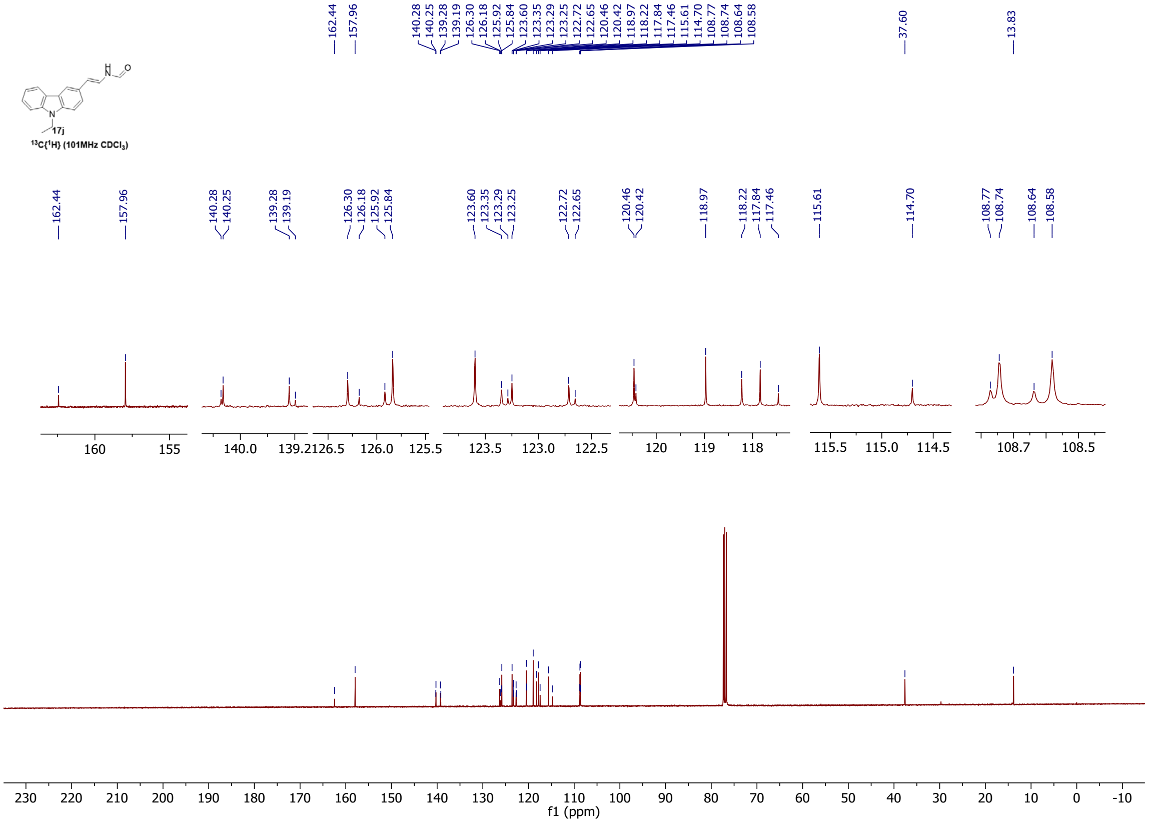

17j
13C{1H} (101MHz CDCl3)

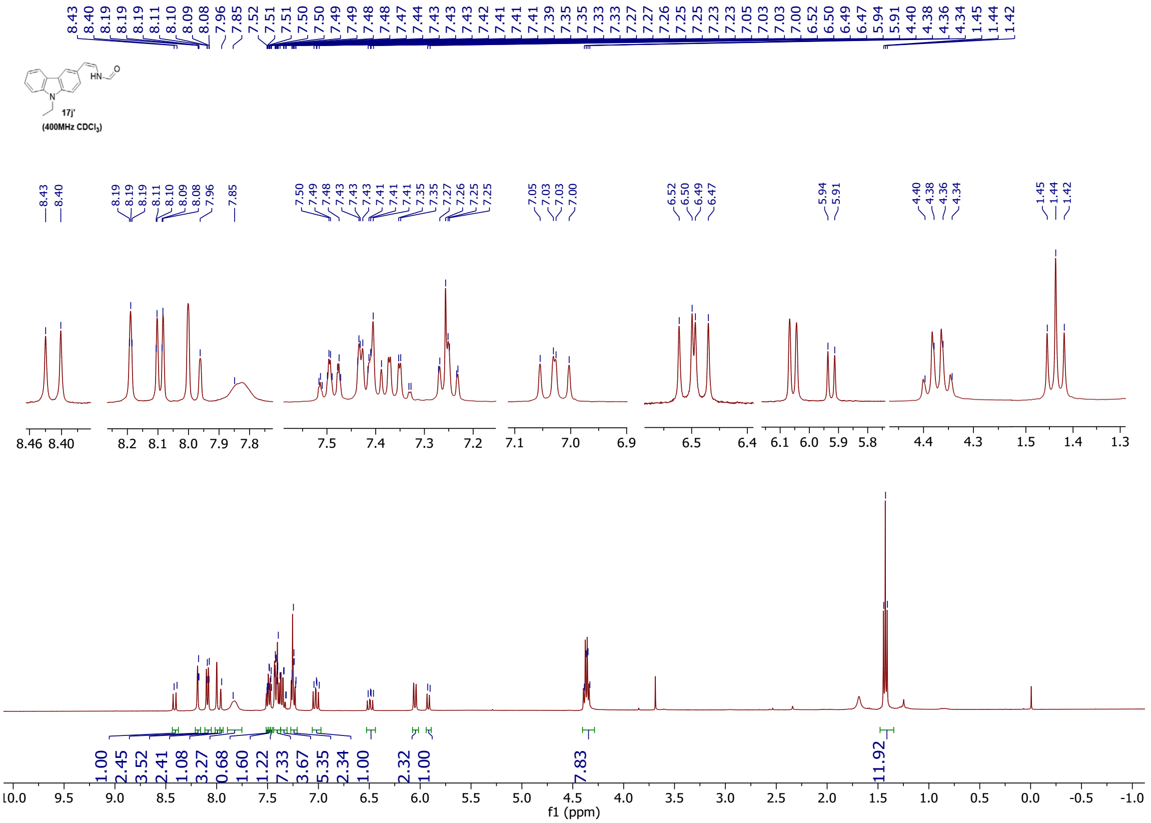
17j'
(400MHz CDCl3)
f1 (ppm)

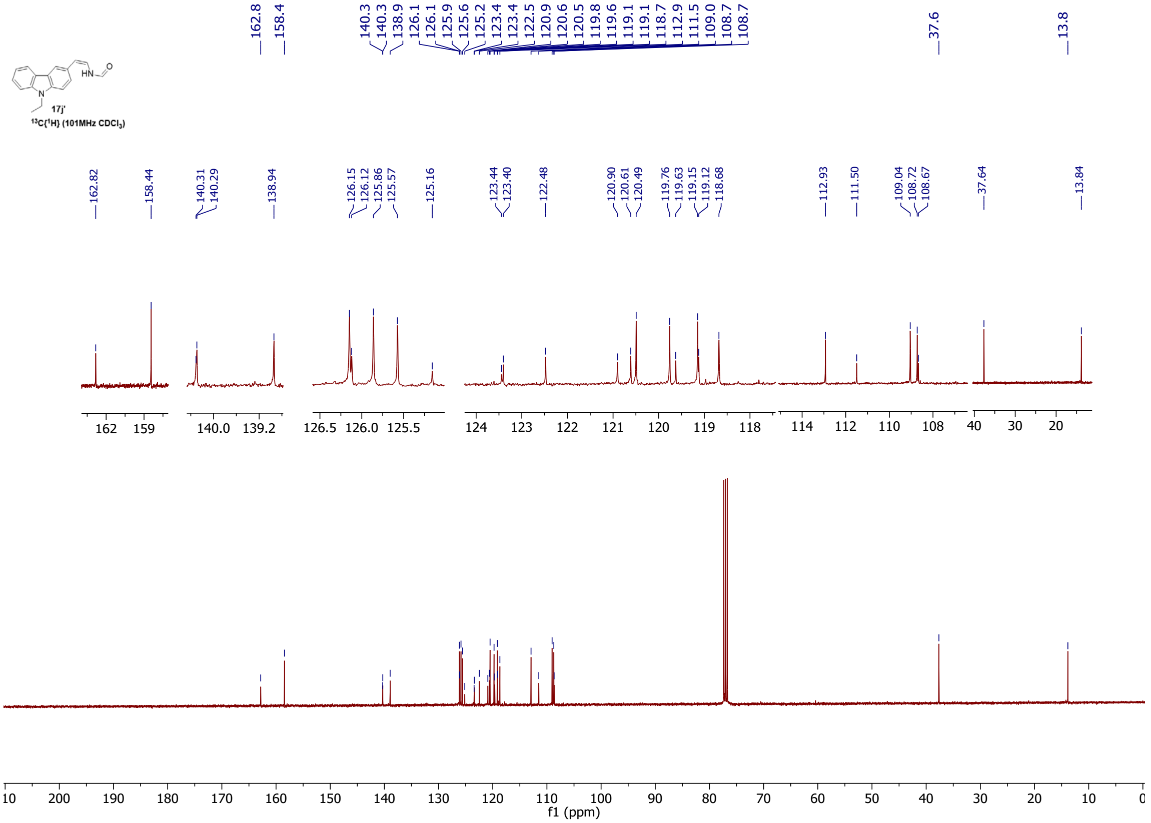
17j'
13C{1H} (101MHz CDCl3)
f1 (ppm)

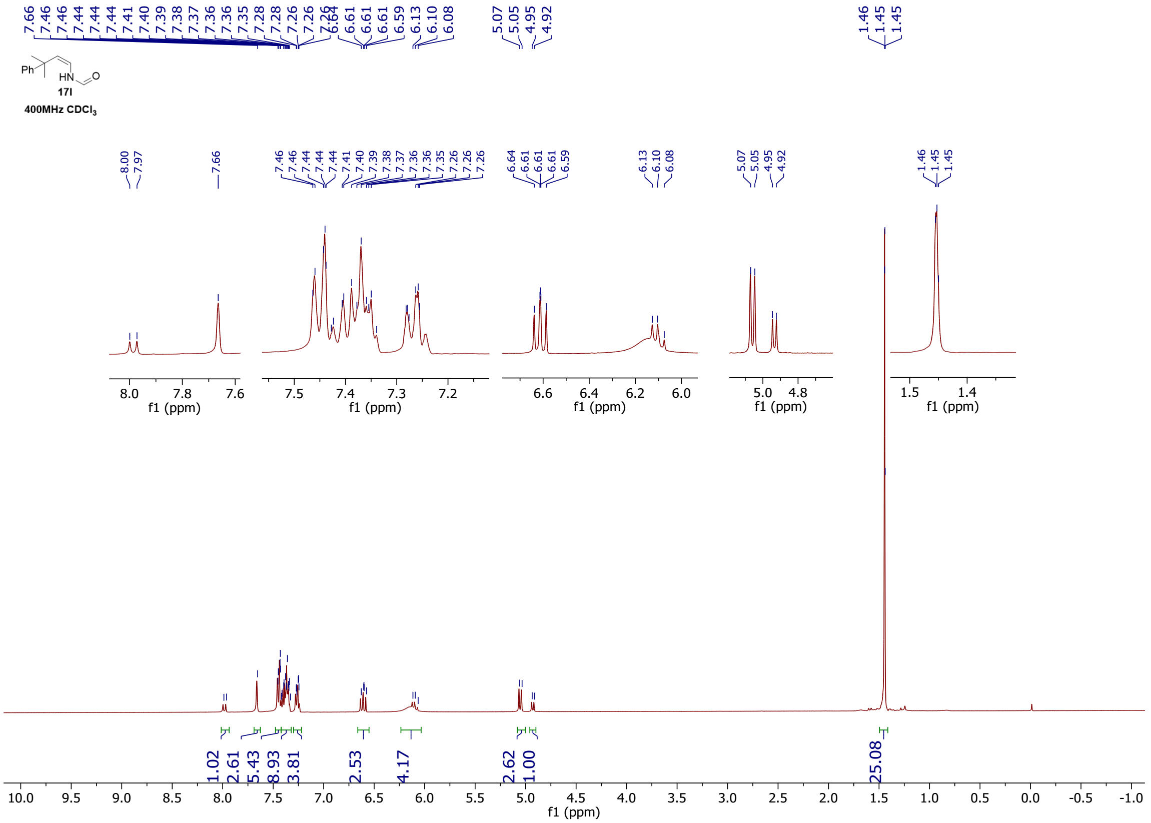

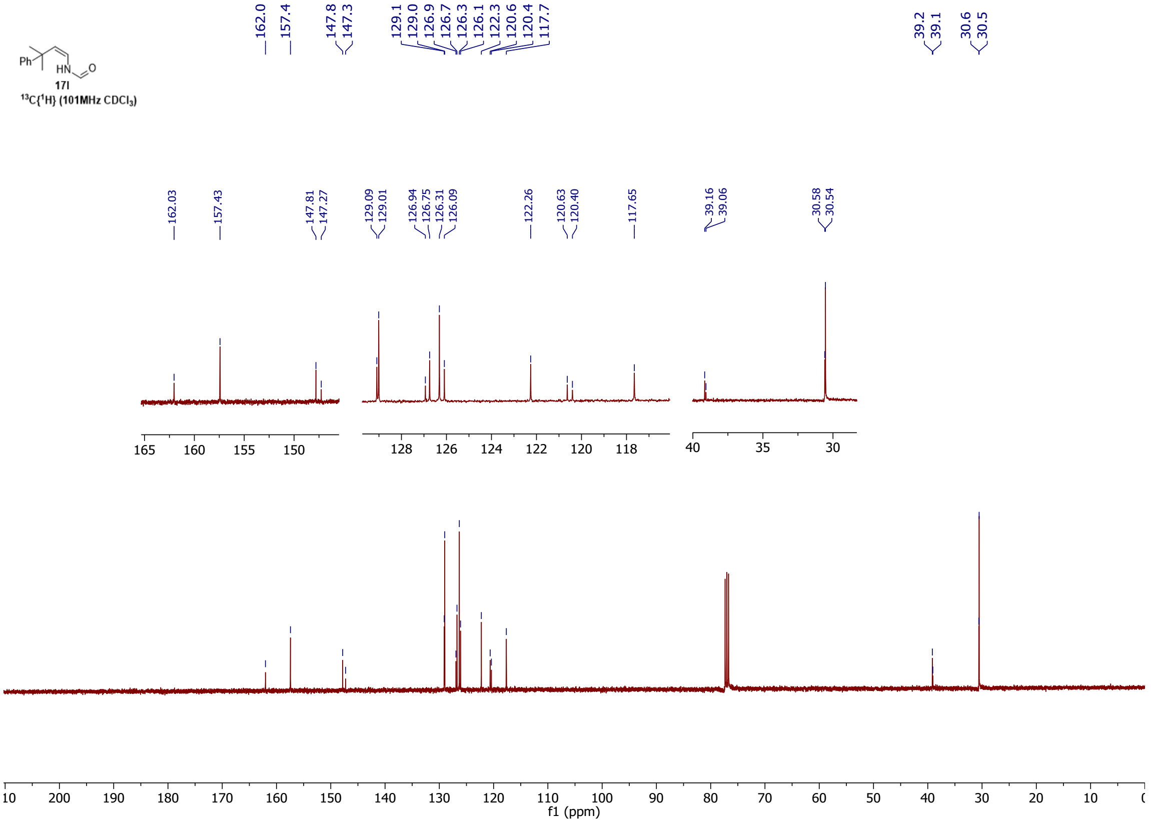

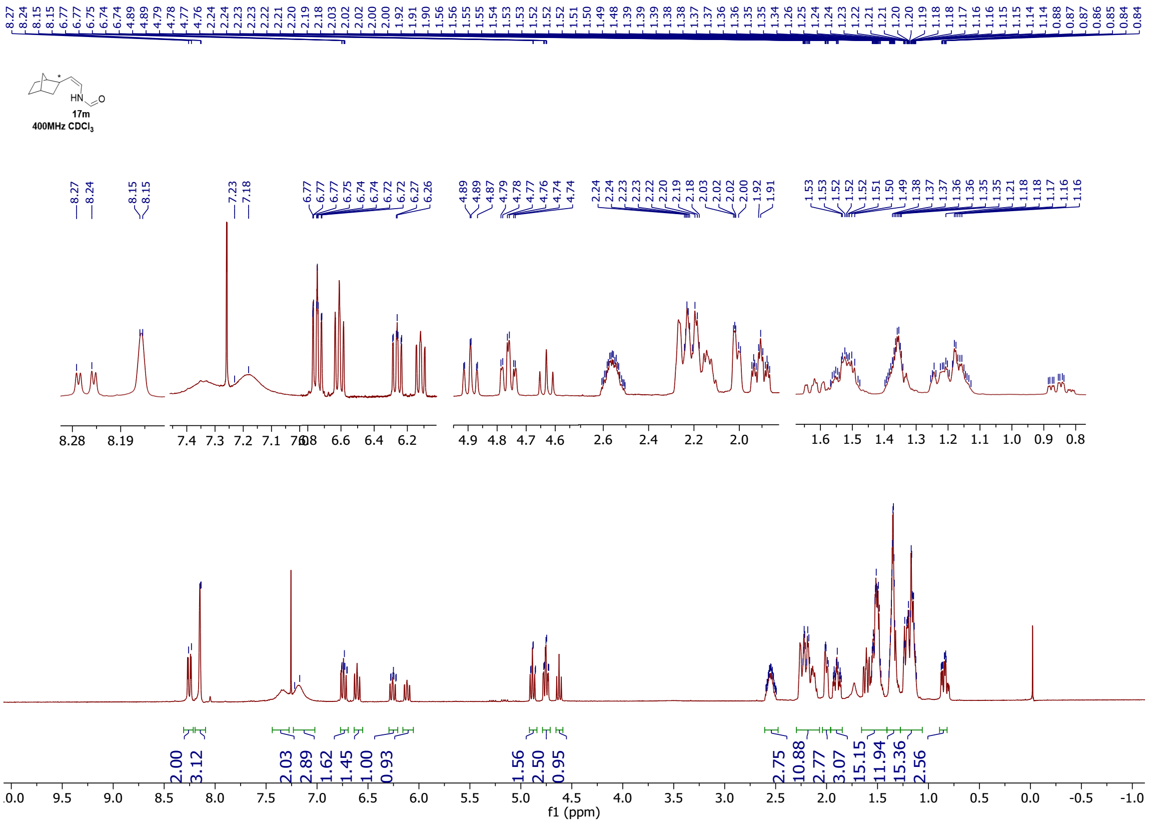

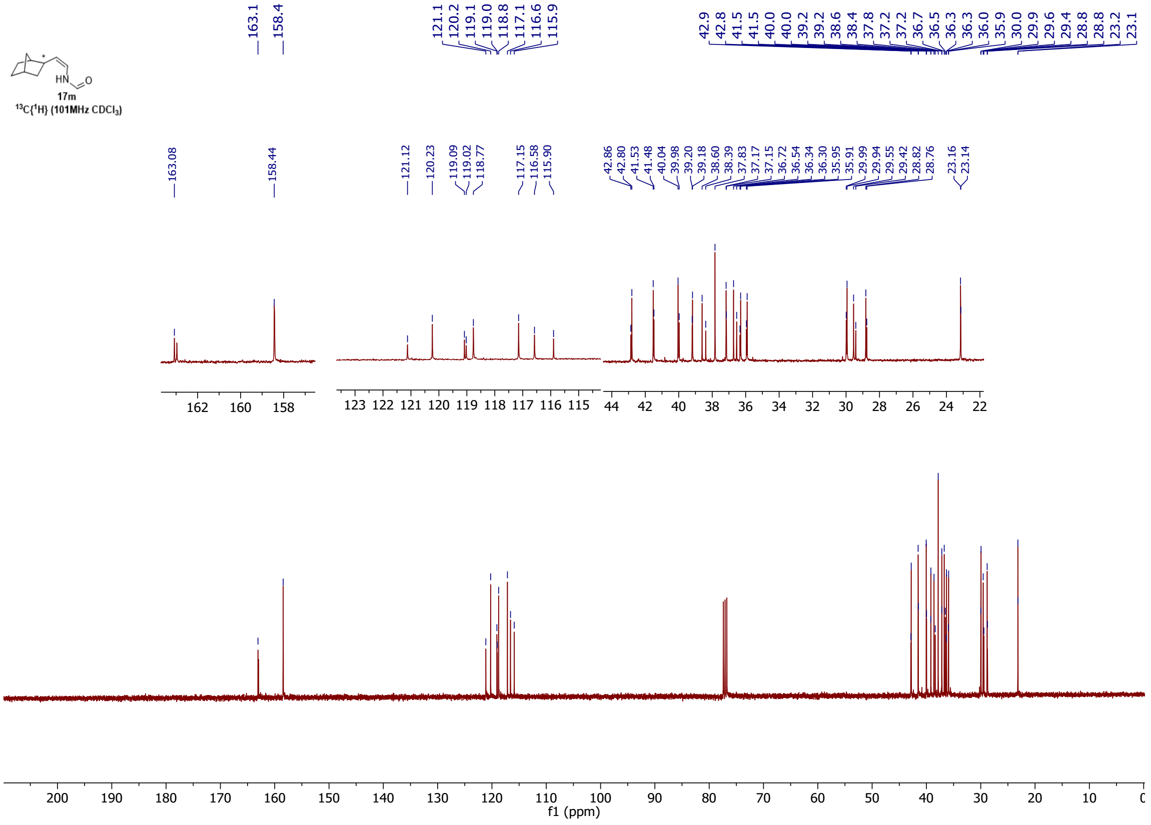

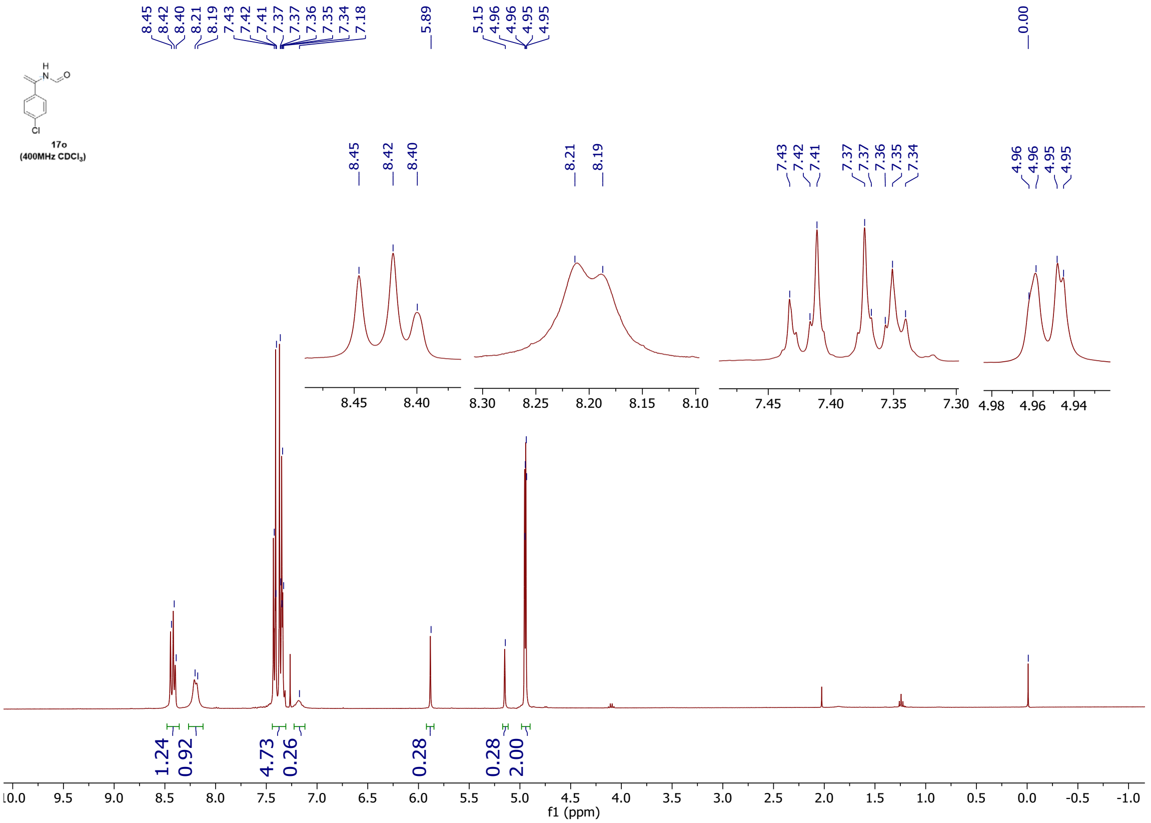
17o
(400MHz CDCl3)
f1 (ppm)

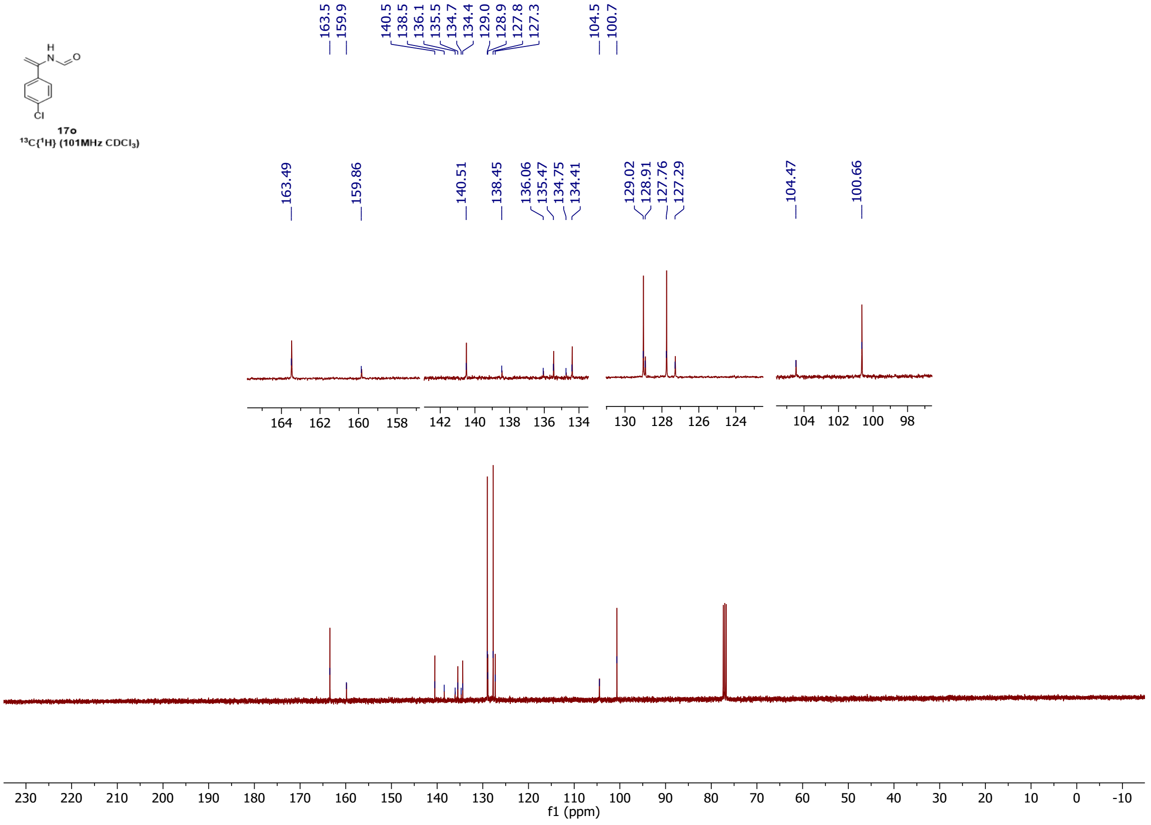
17o
13C{1H} (101MHz CDCl3)
f1 (ppm)

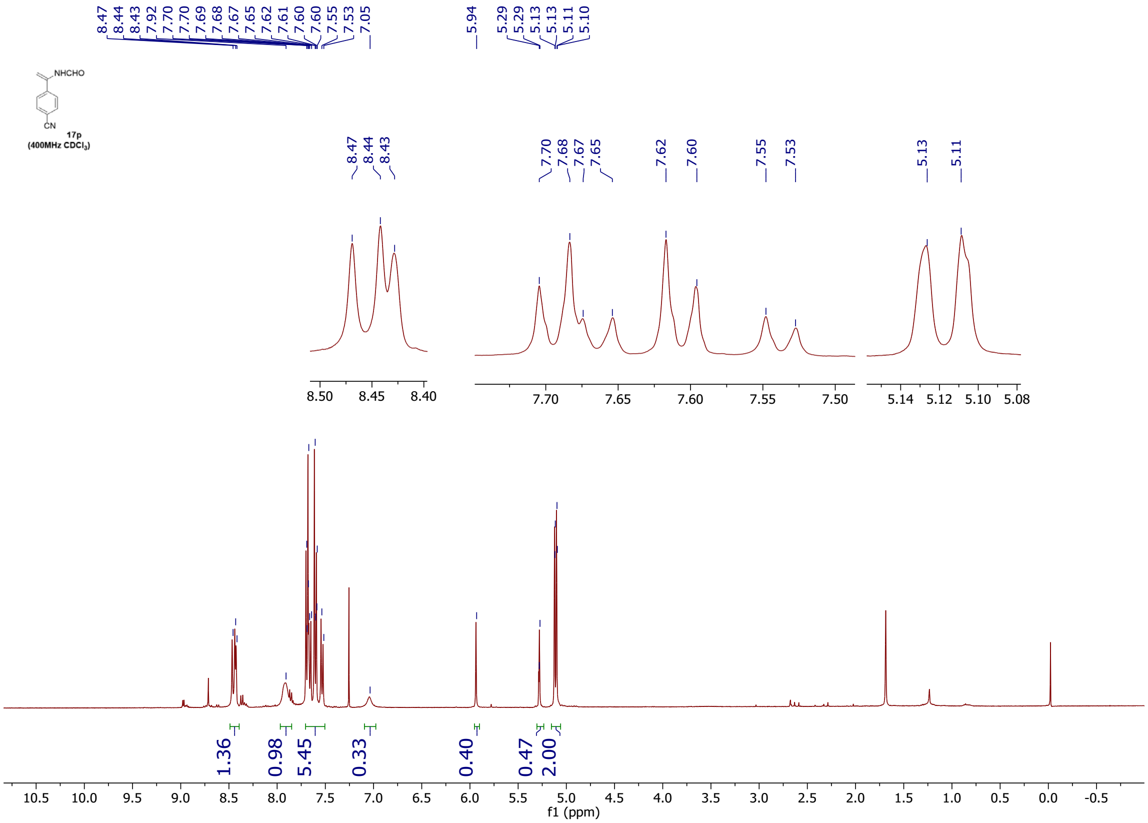

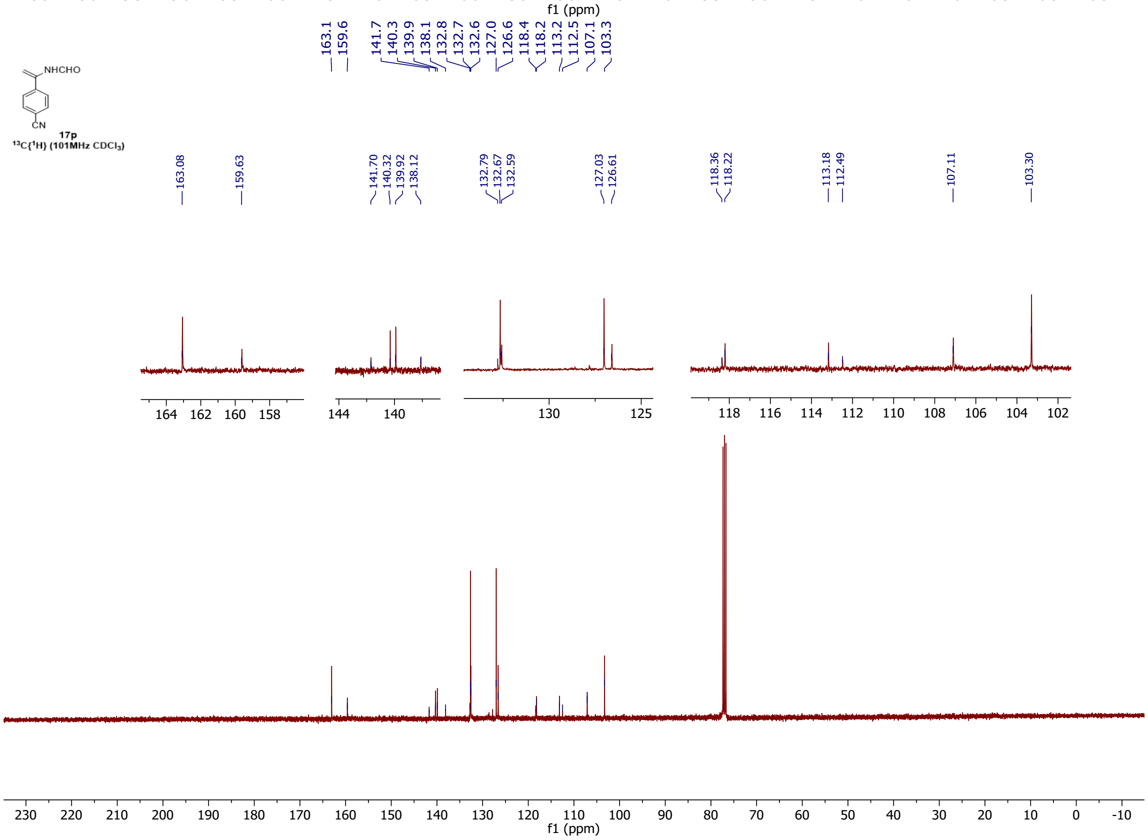

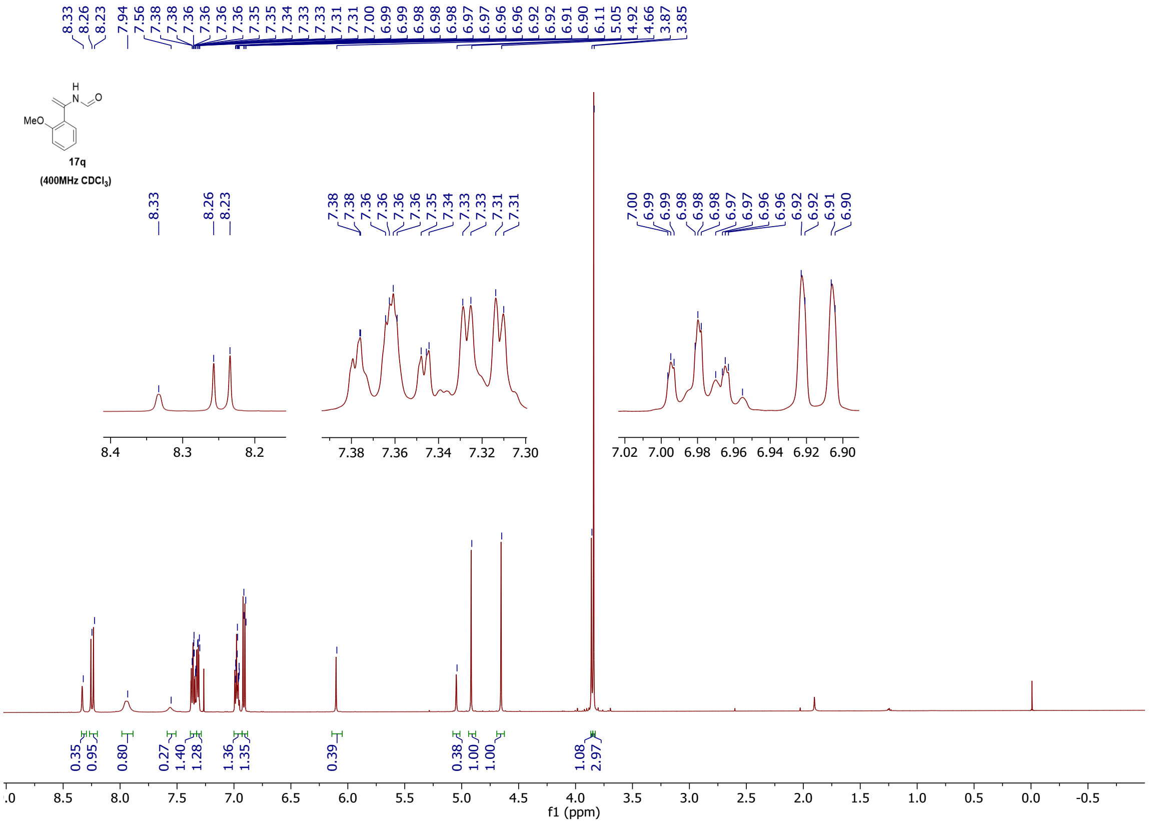
17q
(400MHz CDCl3)
f1 (ppm)

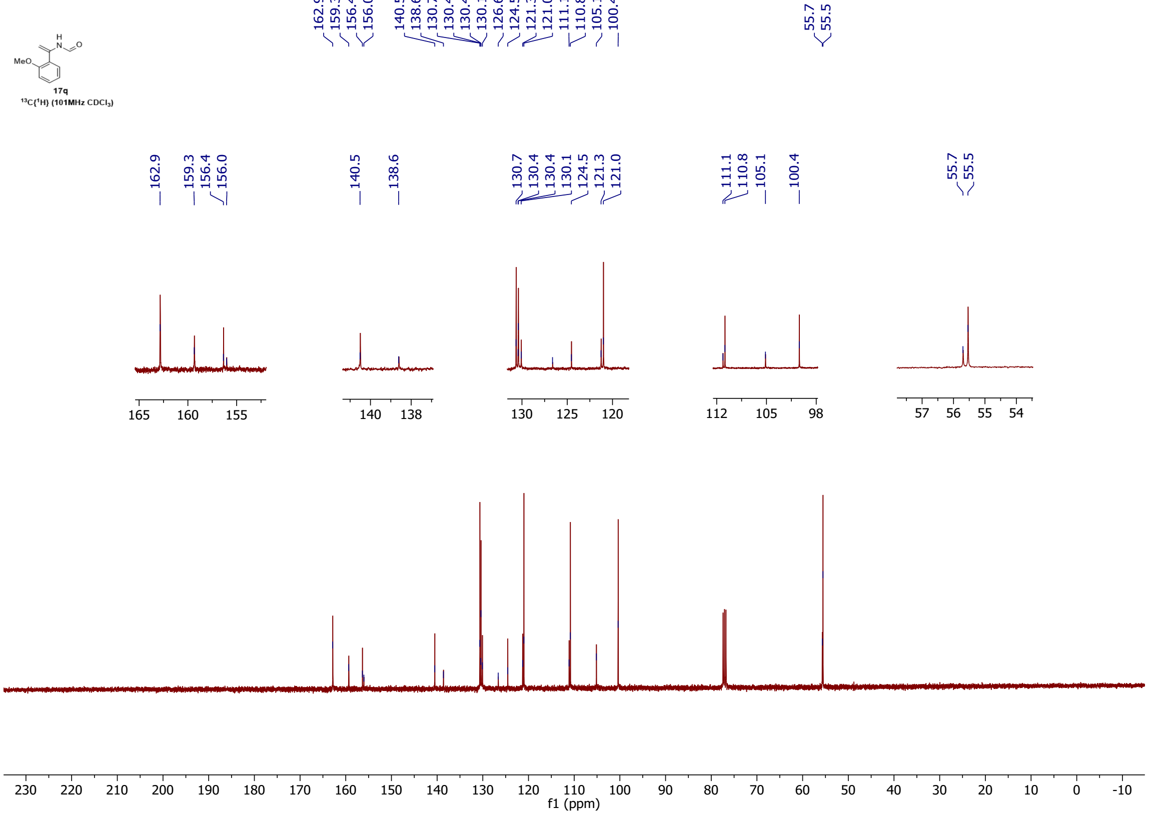
17q
13C{1H} (101MHz CDCl3)
f1 (ppm)

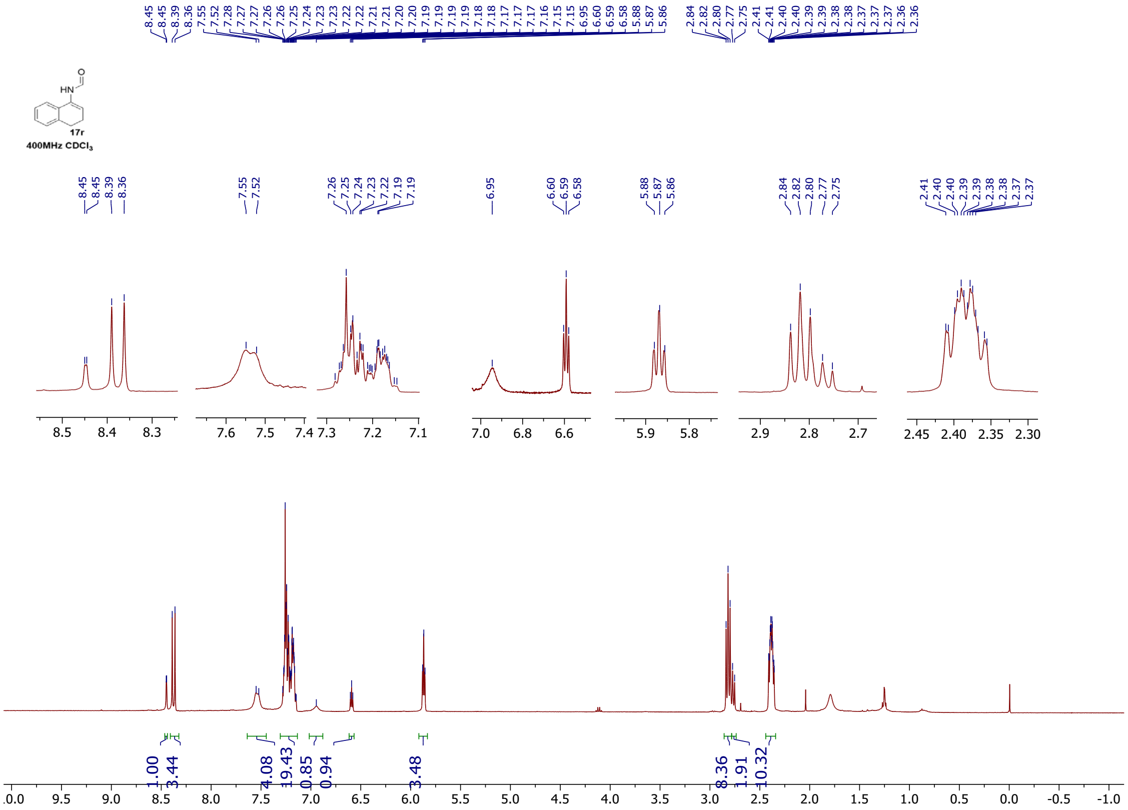
17r
400MHz CDCl3

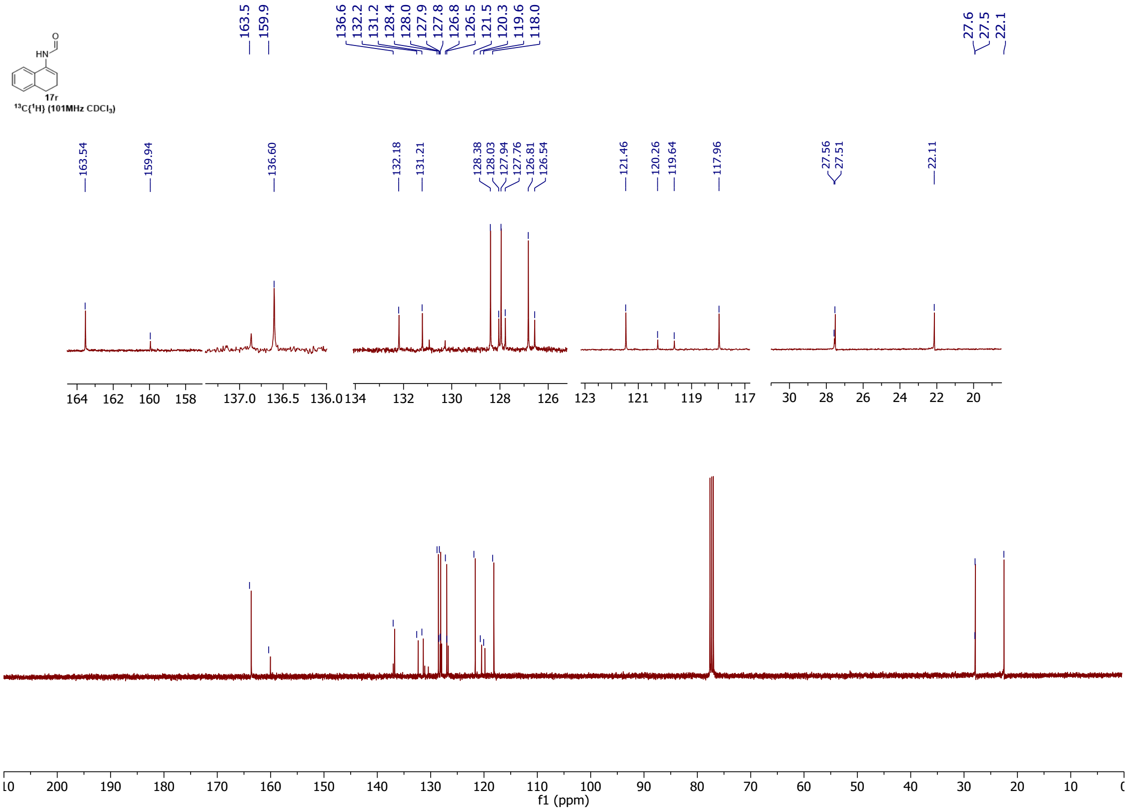
163.5
159.9
136.6
132.2
131.2
128.4
128.0
127.9
127.8
126.8
126.5
121.5
120.3
119.6
118.0
27.6
27.5
22.1
17r
13C{1H} (101MHz CDCl3)
f1 (ppm)

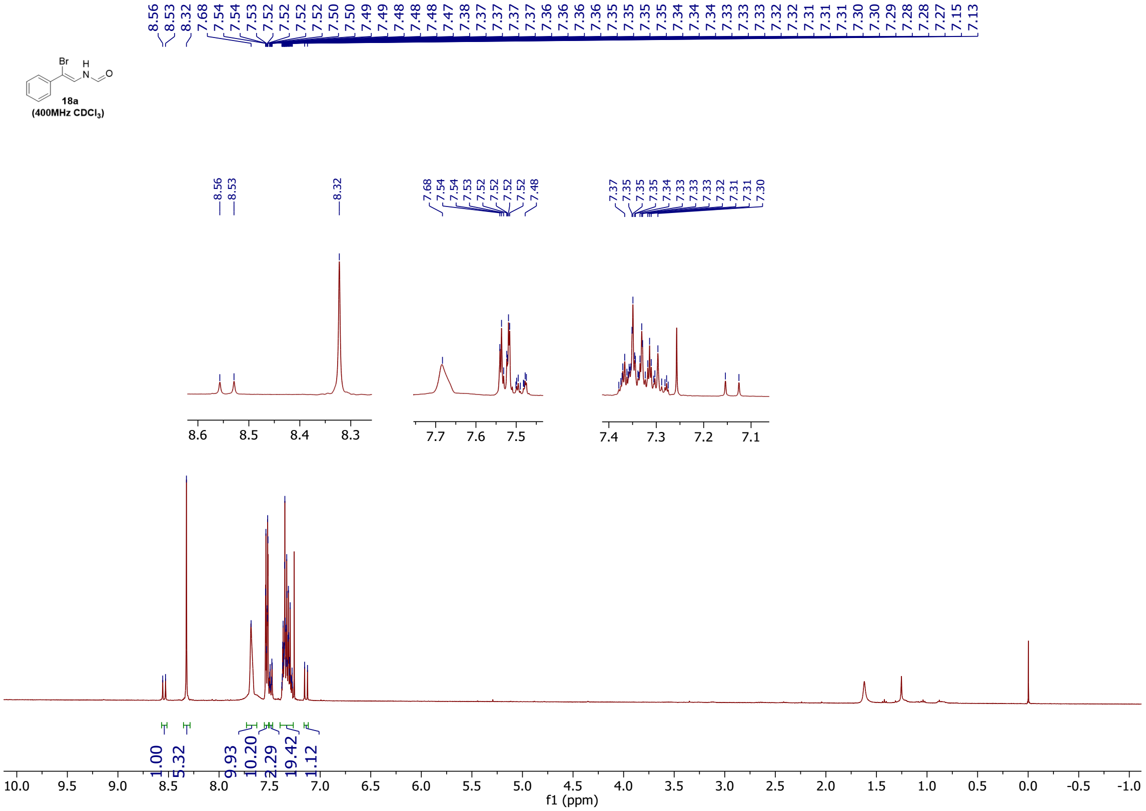
18a
(400MHz CDCl3)
8.56 8.53 8.32 7.68 7.54 7.54 7.53 7.52 7.52 7.52 7.52 7.52 7.50 7.50 7.49 7.49 7.48 7.48 7.48 7.47 7.38 7.37 7.37 7.37 7.37 7.36 7.36 7.36 7.35 7.35 7.35 7.35 7.34 7.34 7.33 7.33 7.33 7.32 7.32 7.31 7.31 7.31 7.30 7.29 7.28 7.28 7.27 7.15 7.13
1.00 5.32 9.93 10.20 2.29 19.42 1.12
f1 (ppm)

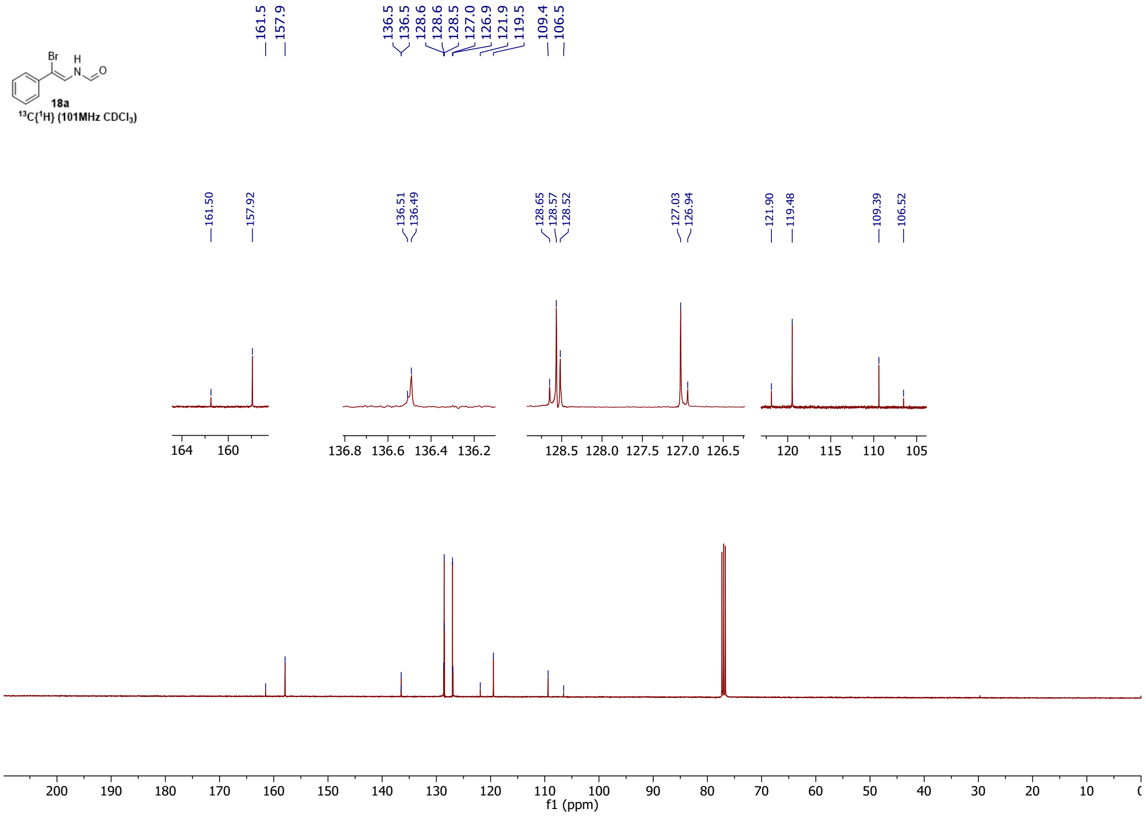
18a
13C{1H} (101MHz CDCl3)
161.5 157.9 136.5 136.5 128.6 128.6 128.5 127.0 126.9 121.9 119.5 109.4 106.5
161.50 157.92 136.51 136.49 128.65 128.57 128.52 127.03 126.94 121.90 119.48 109.39 106.52
f1 (ppm)

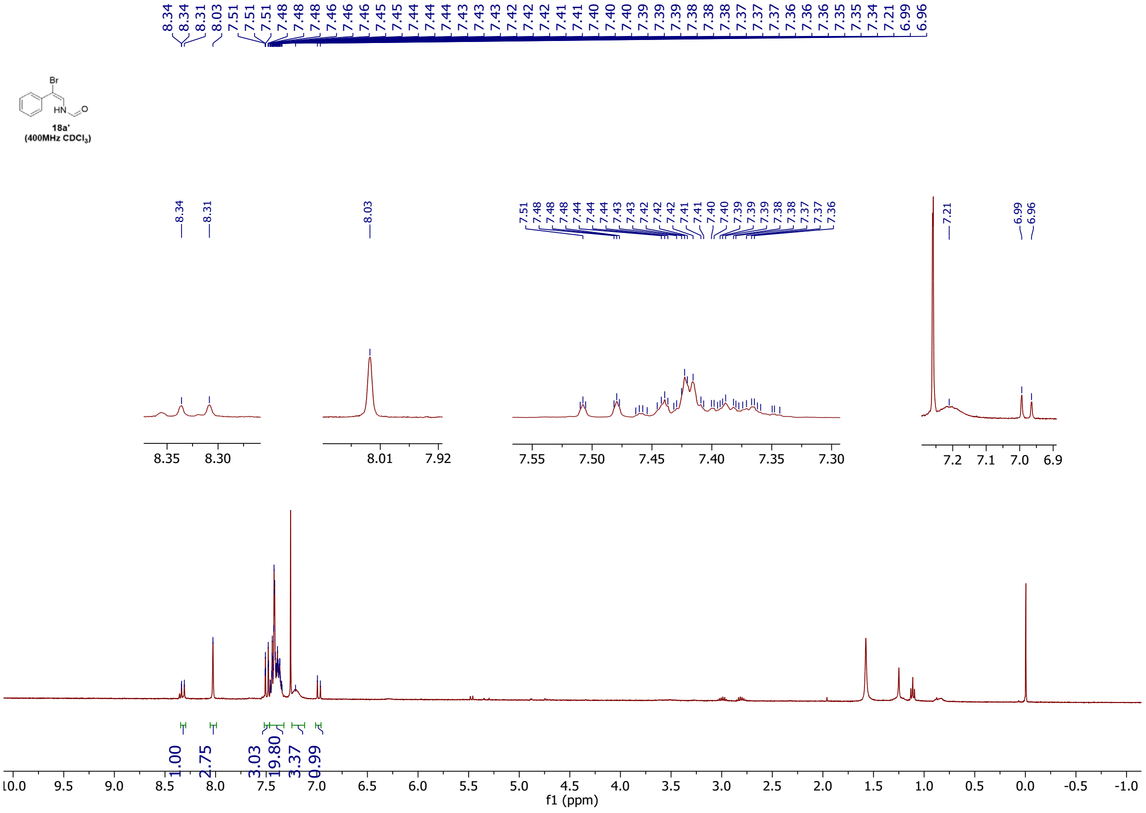
18a'
(400MHz CDCl3)
f1 (ppm)

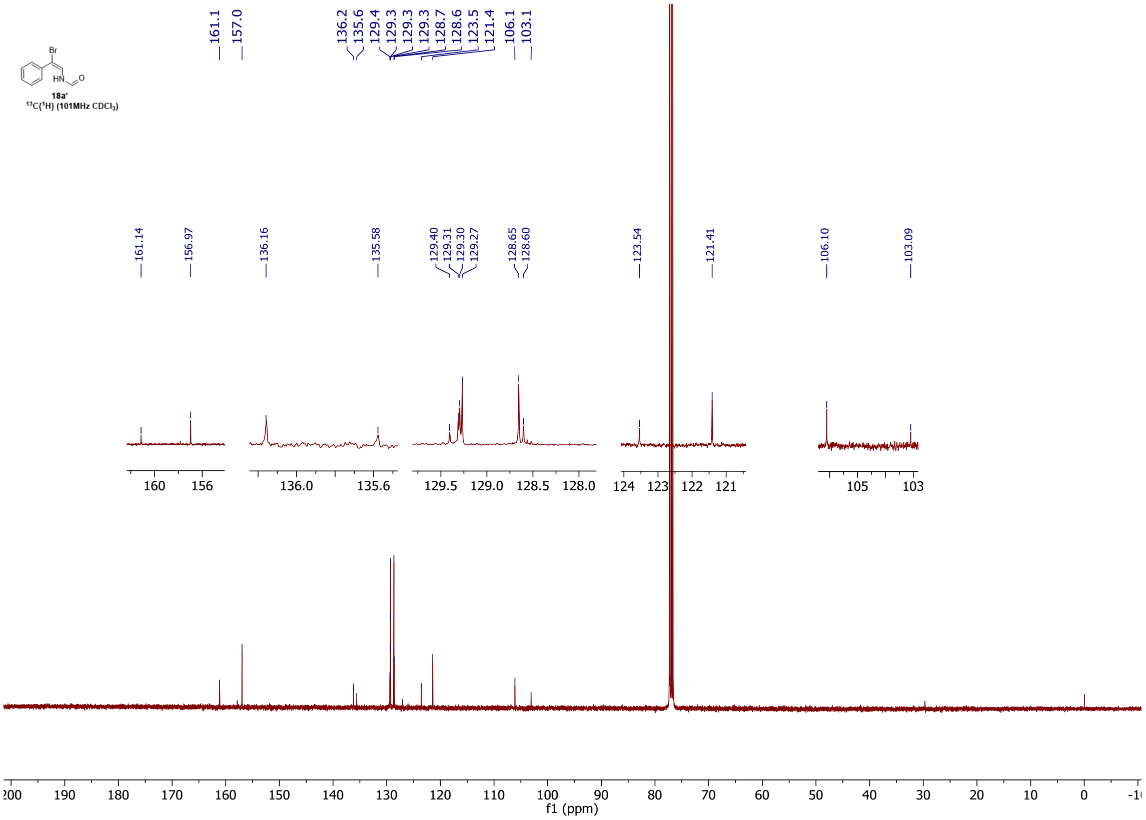
18a'
13C{1H} (101MHz CDCl3)
f1 (ppm)

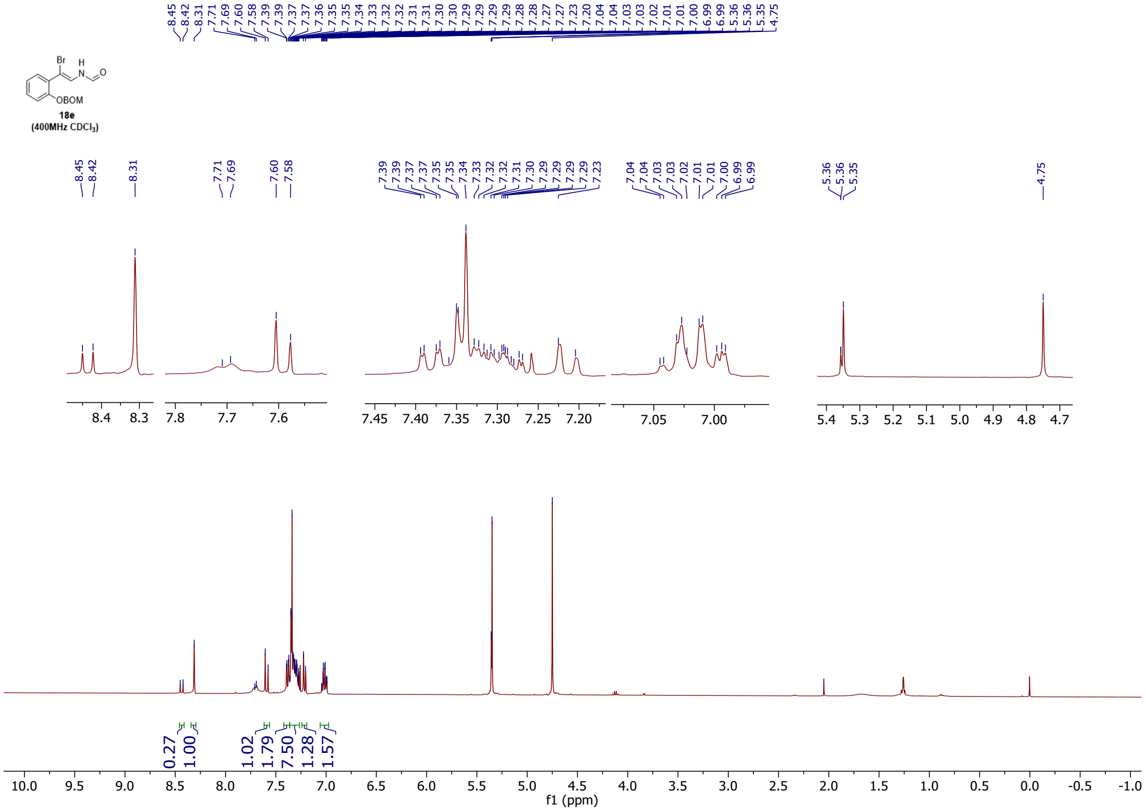

18e
(400MHz $CDCl_3$)

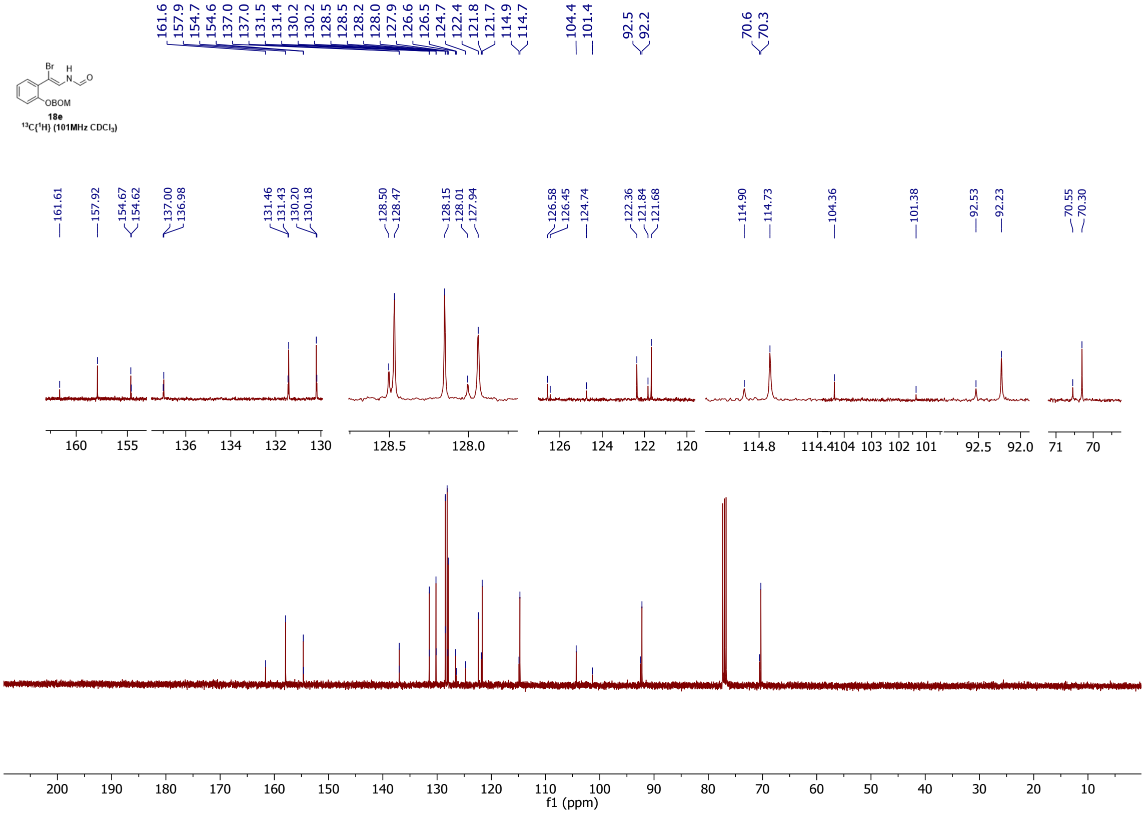

18e
$^{13}C\{^1H\}$ (101MHz $CDCl_3$)

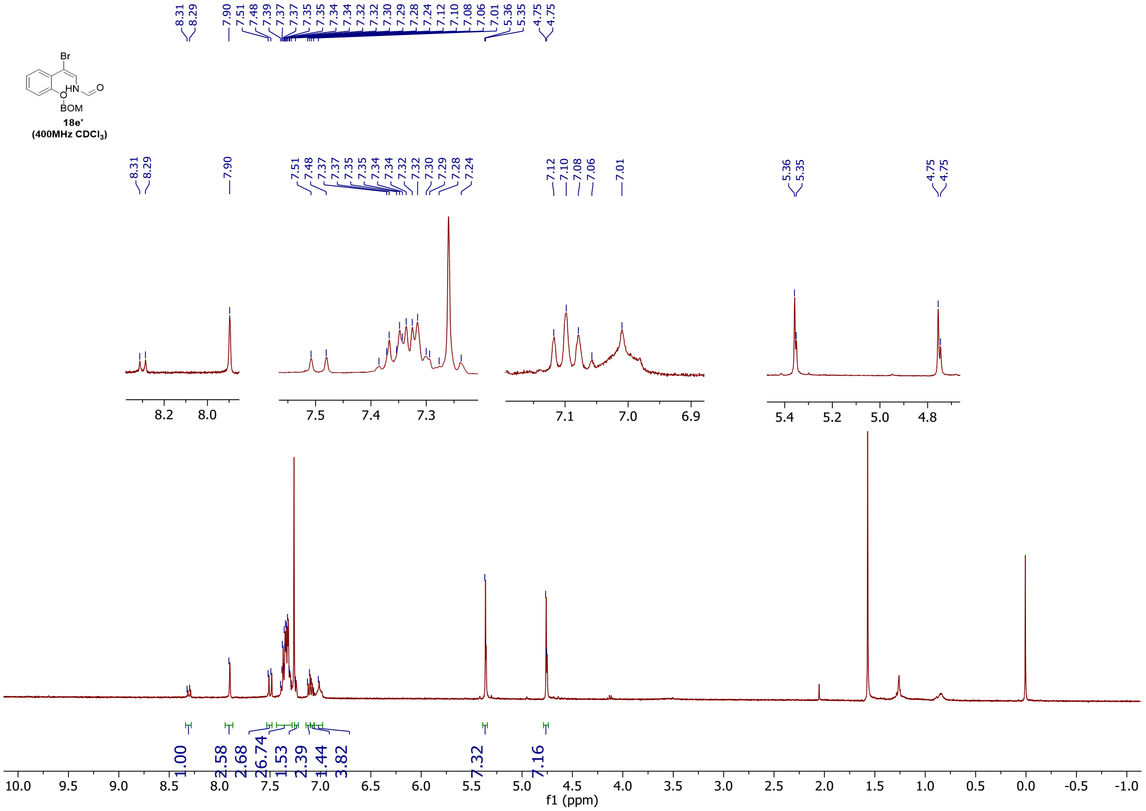
Br
HN
O
BOM
18e'
(400MHz CDCl3)
f1 (ppm)

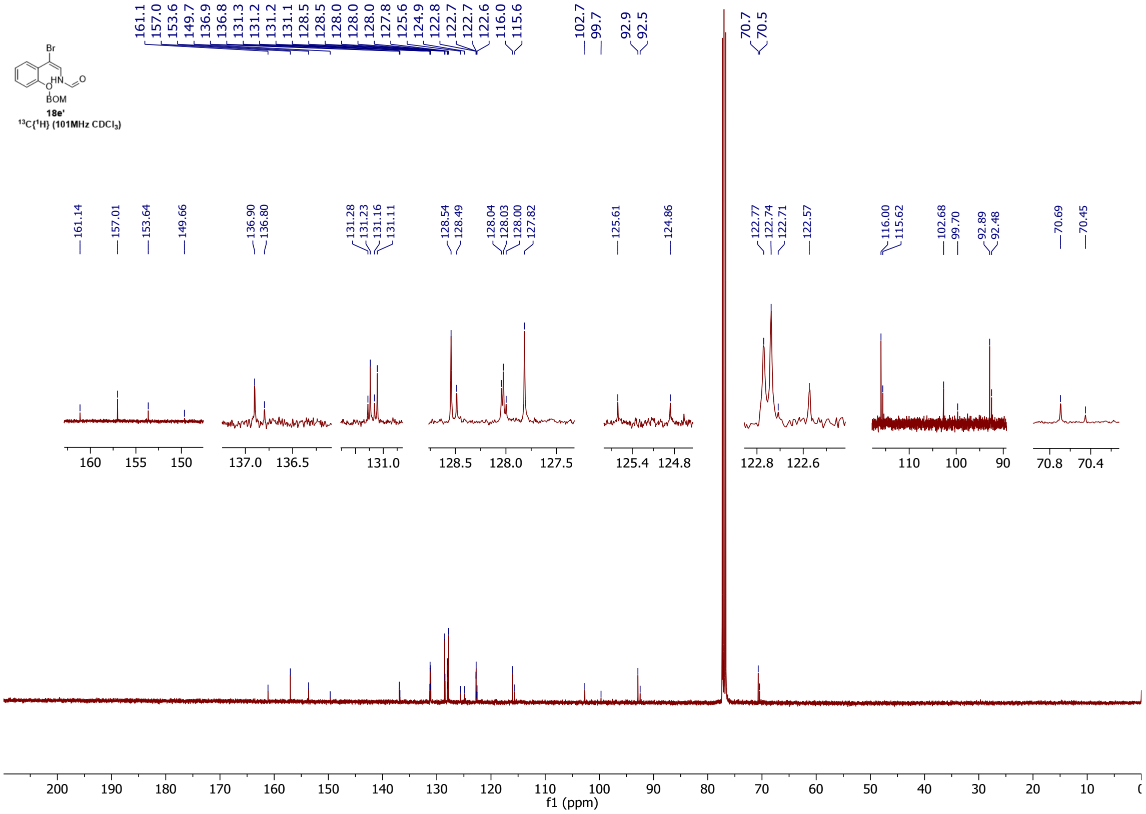
Br
HN
O
BOM
18e'
13C{1H} (101MHz CDCl3)
f1 (ppm)

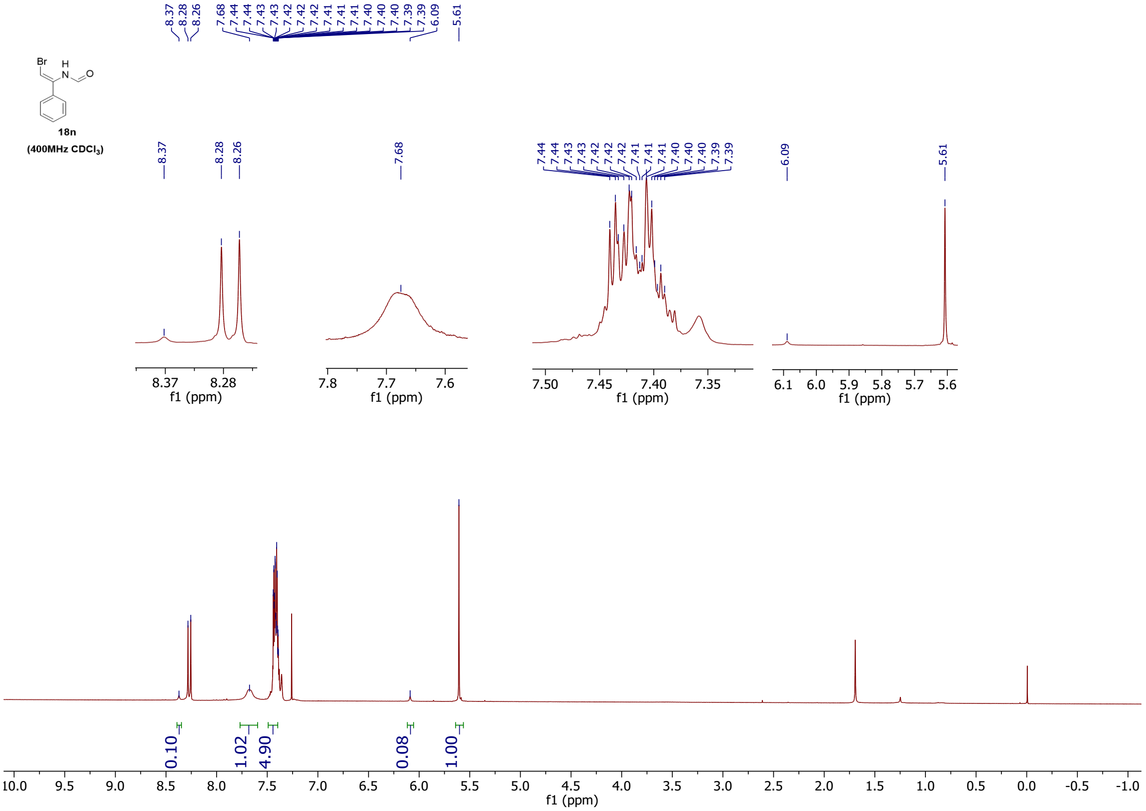

Br
H
N
O
18n
(400MHz CDCl$_3$)
8.37
8.28
8.26
7.68
7.44
7.44
7.43
7.43
7.42
7.42
7.42
7.41
7.41
7.41
7.40
7.40
7.40
7.39
7.39
6.09
5.61
f1 (ppm)
0.10
1.02
4.90
0.08
1.00

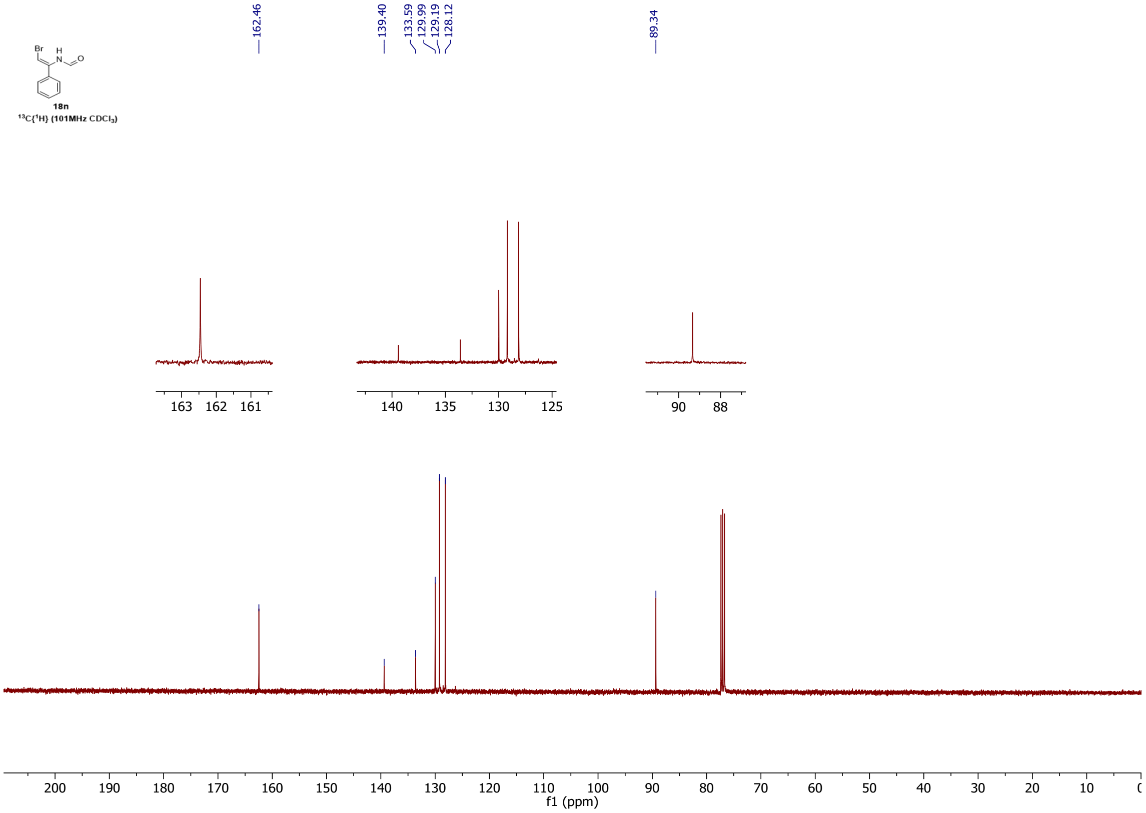

Br
H
N
O
18n
$^{13}C\{^1H\}$ (101MHz CDCl$_3$)
162.46
139.40
133.59
129.99
129.19
128.12
89.34
f1 (ppm)

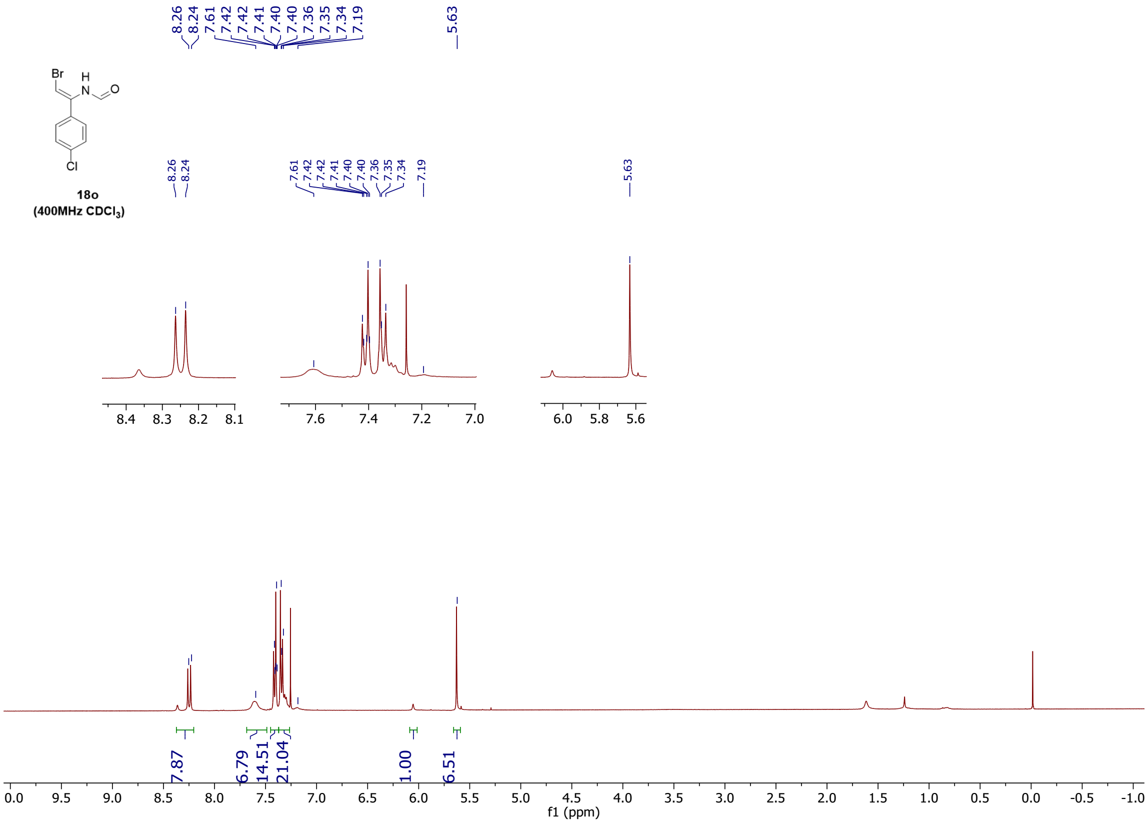
8.26
8.24
7.61
7.42
7.42
7.41
7.40
7.40
7.36
7.35
7.34
7.19
5.63
Br
H
N
O
Cl
18o
(400MHz CDCl3)
7.87
6.79
14.51
21.04
1.00
6.51
f1 (ppm)

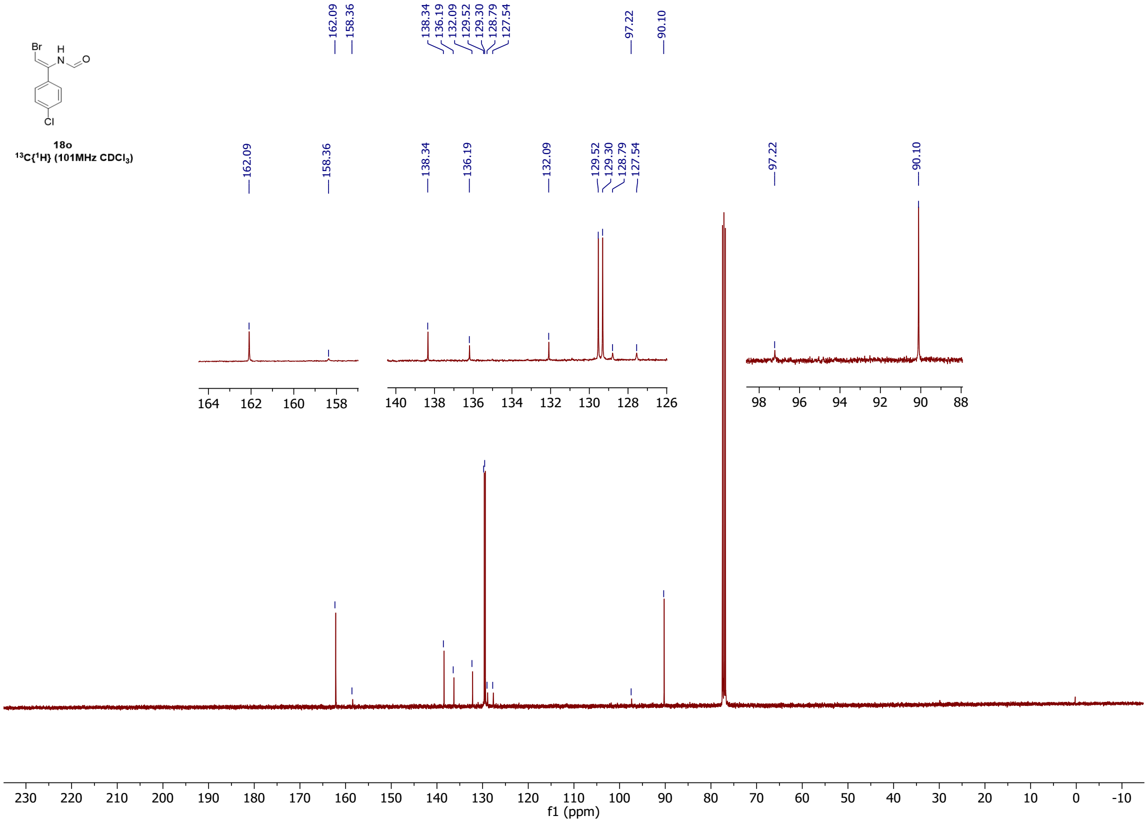
162.09
158.36
138.34
136.19
132.09
129.52
129.30
128.79
127.54
97.22
90.10
Br
H
N
O
Cl
18o
13C{1H} (101MHz CDCl3)
f1 (ppm)

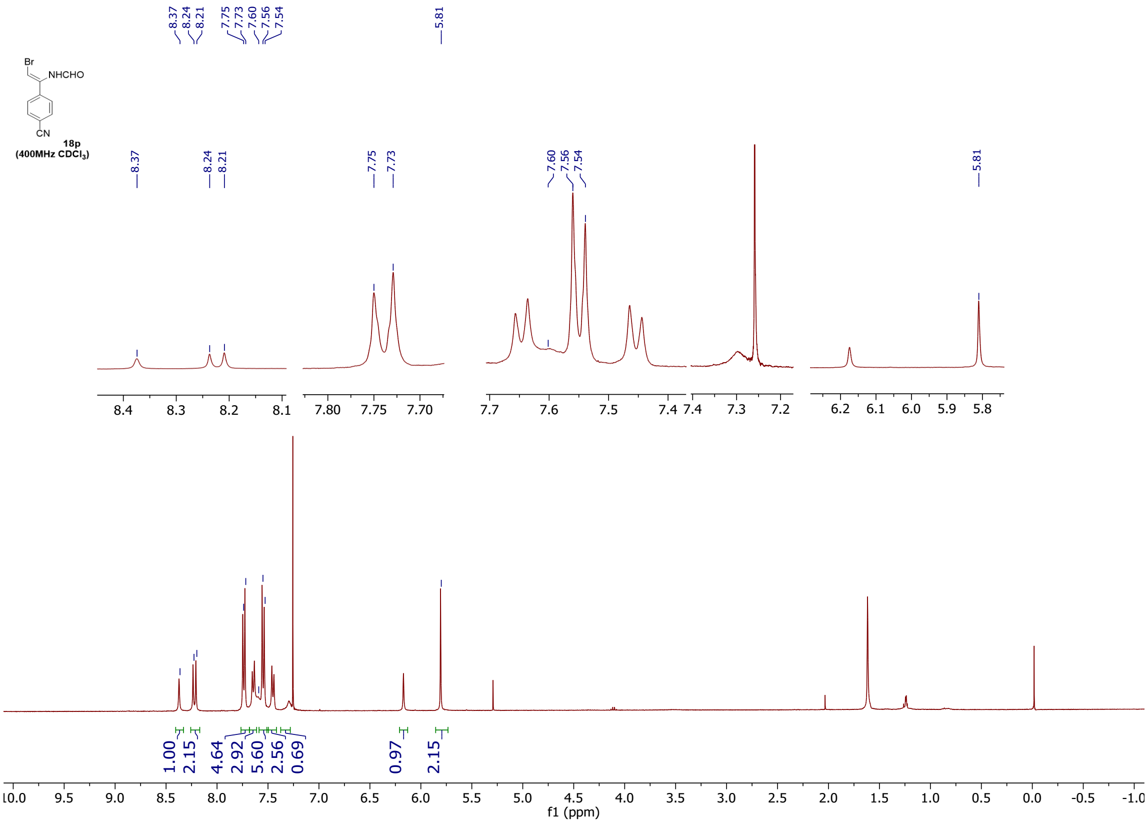
Br
NHCHO
CN
18p
(400MHz CDCl3)
f1 (ppm)

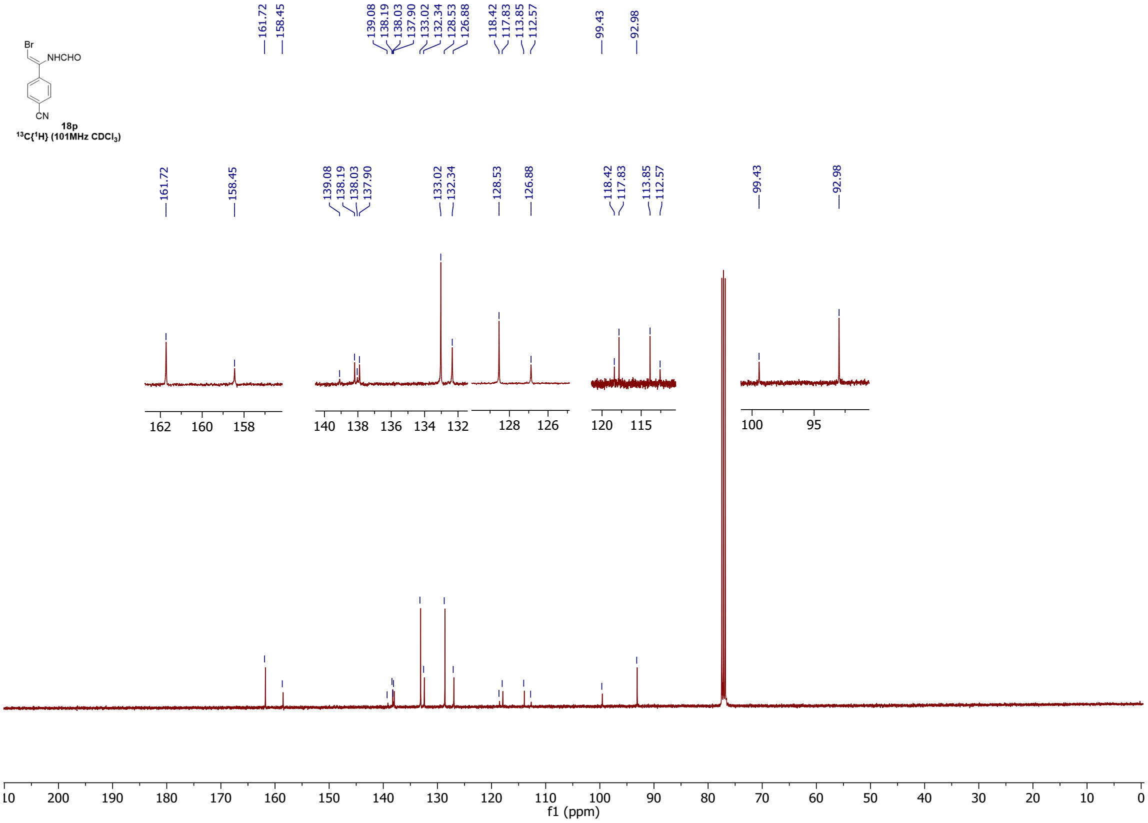
Br
NHCHO
CN
18p
13C{1H} (101MHz CDCl3)
f1 (ppm)

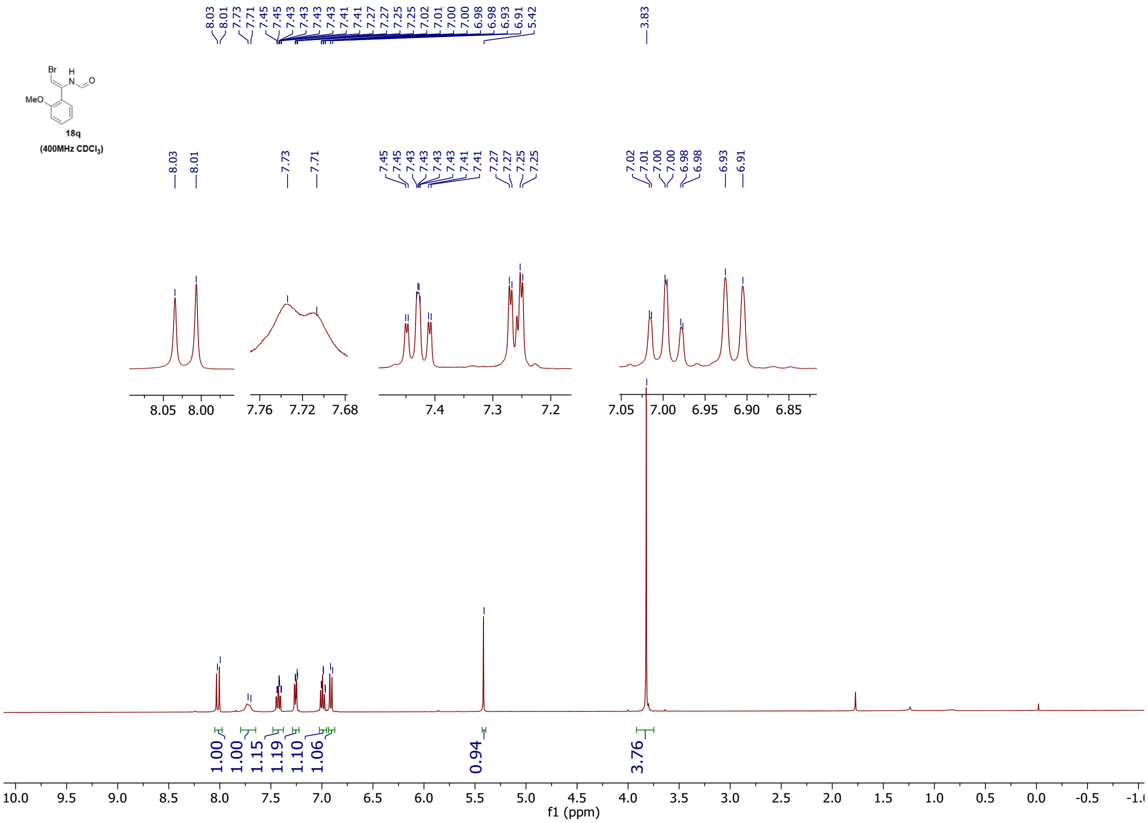
Br
H
N
O
MeO
18q
(400MHz CDCl3)
8.03 8.01 7.73 7.71 7.45 7.43 7.43 7.43 7.43 7.41 7.41 7.27 7.27 7.25 7.25 7.02 7.01 7.00 7.00 6.98 6.98 6.93 6.91 5.42
3.83
1.00 1.00 1.15 1.19 1.10 1.06 0.94 3.76
f1 (ppm)

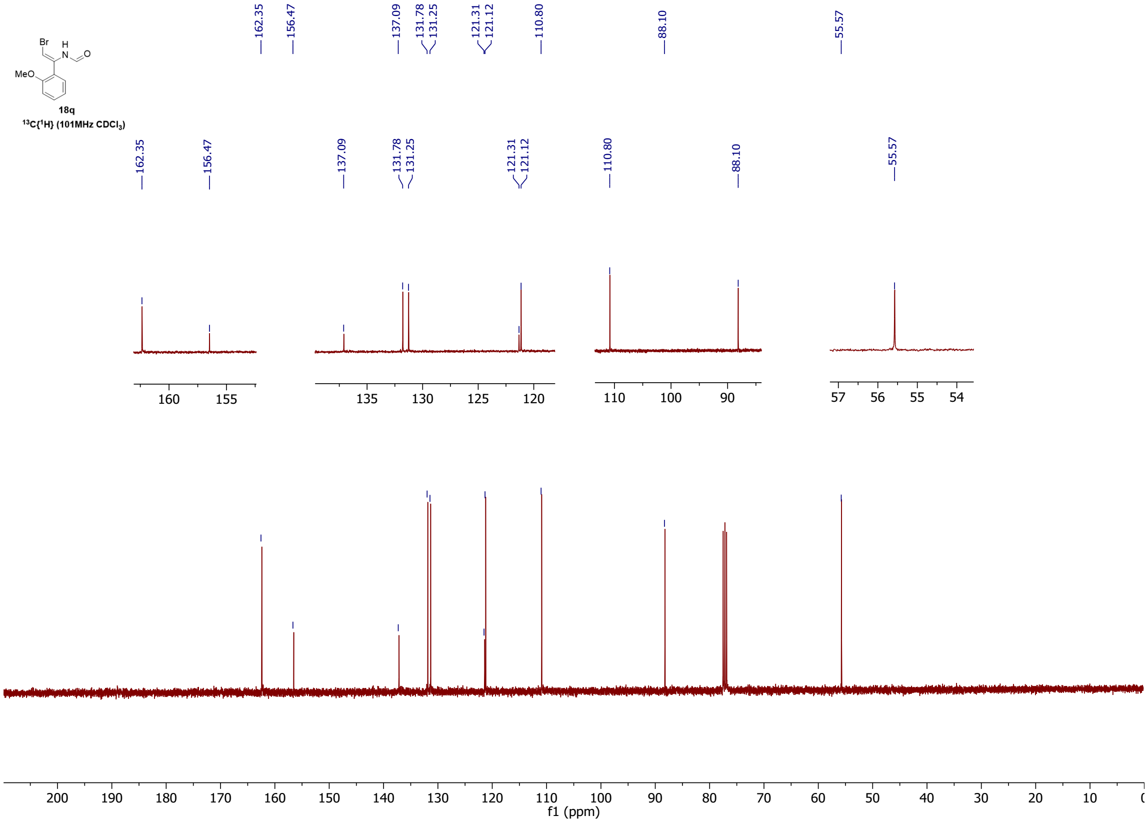
Br
H
N
O
MeO
18q
13C(1H) (101MHz CDCl3)
162.35 156.47 137.09 131.78 131.25 121.31 121.12 110.80 88.10 55.57
f1 (ppm)

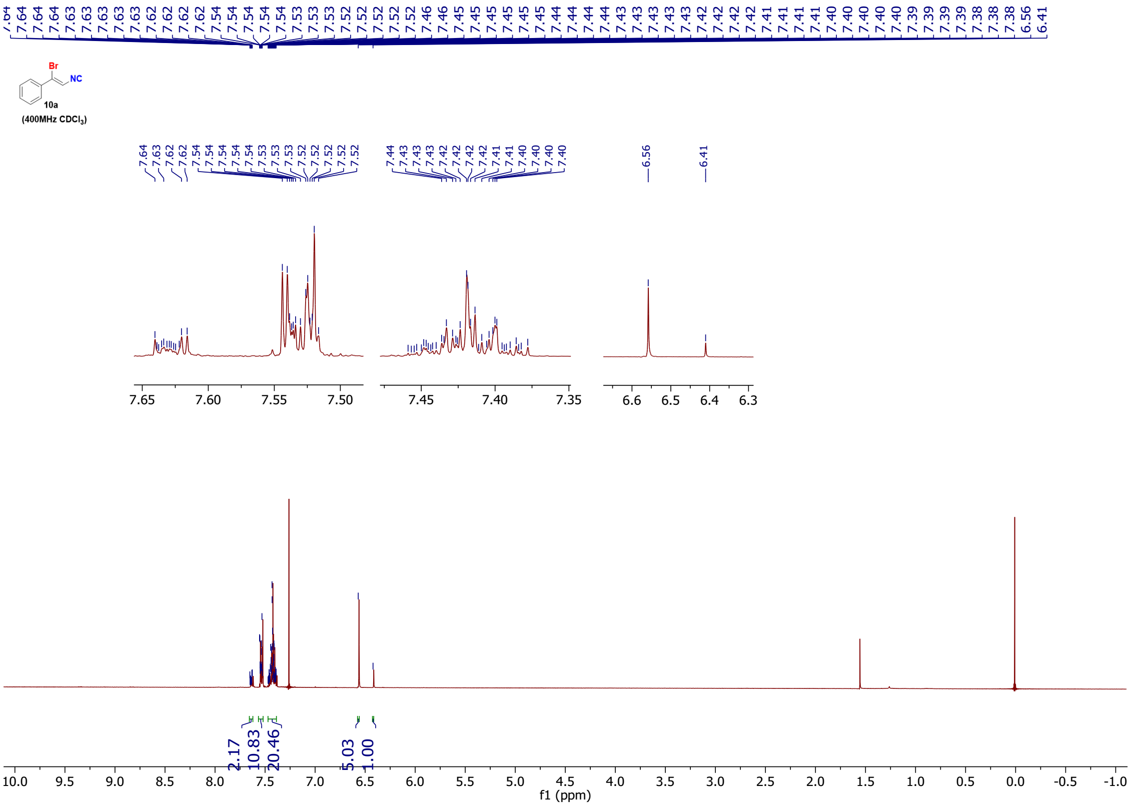
Br
NC
10a
(400MHz CDCl3)
f1 (ppm)

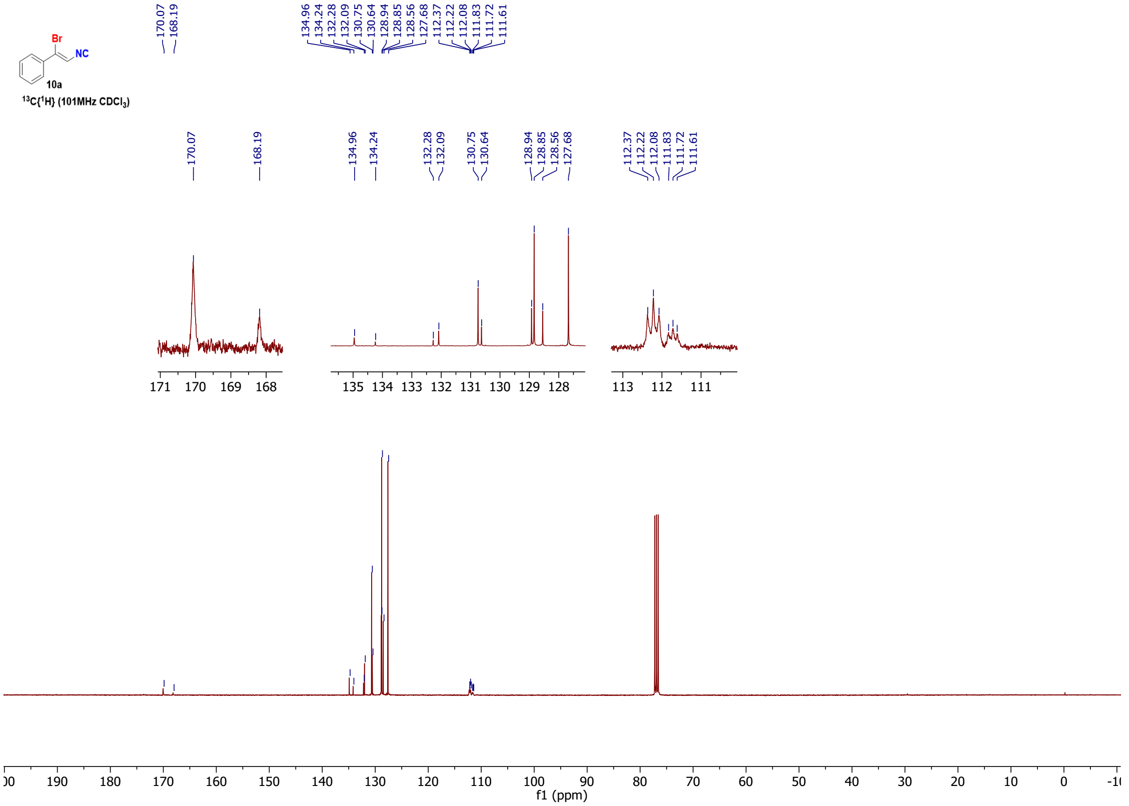
Br
NC
10a
13C{1H} (101MHz CDCl3)
f1 (ppm)

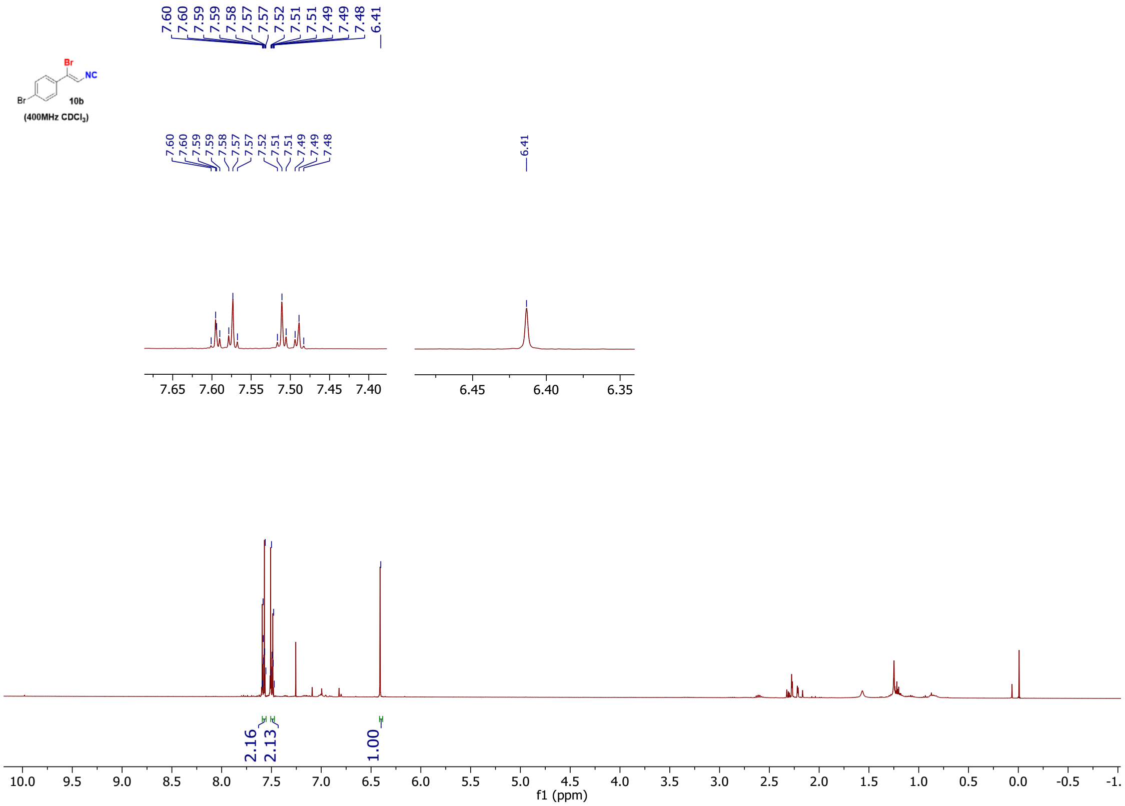
Br
NC
Br
10b
(400MHz CDCl3)
7.60
7.60
7.59
7.59
7.58
7.57
7.57
7.52
7.51
7.51
7.49
7.49
7.48
6.41
2.16
2.13
1.00
f1 (ppm)

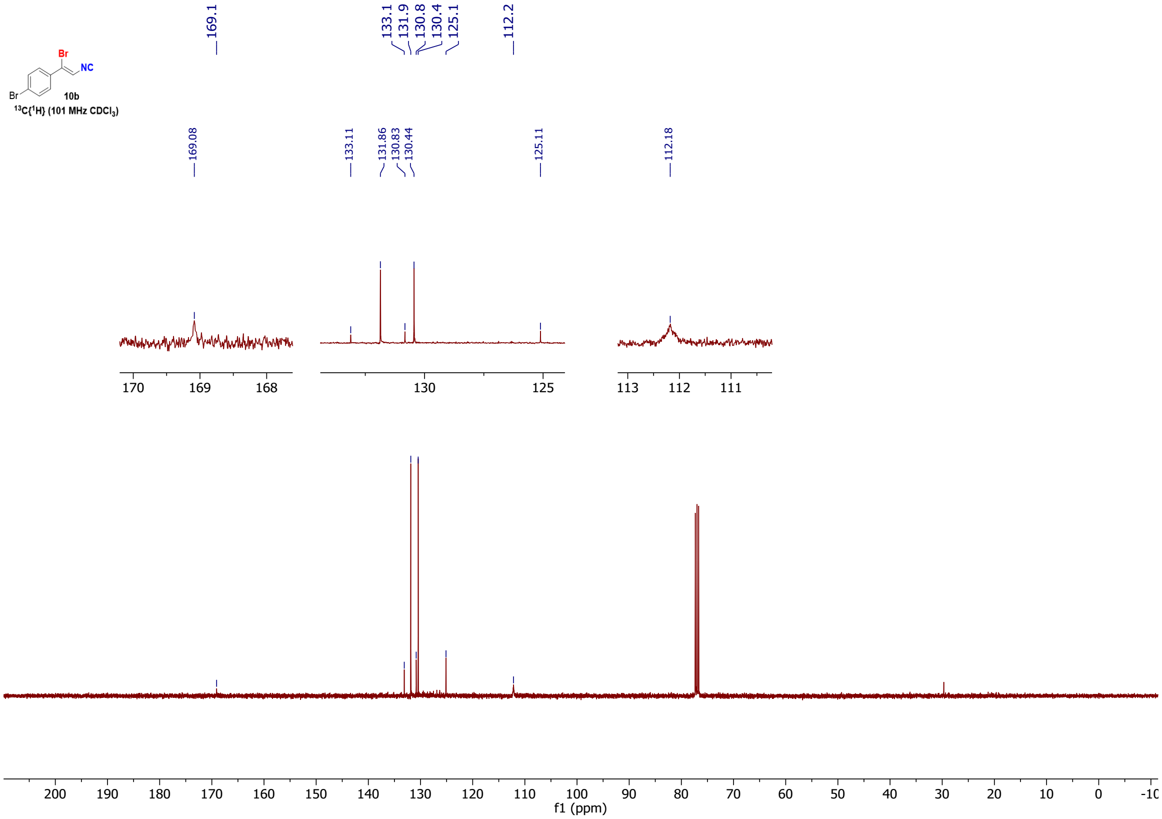
Br
NC
Br
10b
13C{1H} (101 MHz CDCl3)
169.1
133.1
131.9
130.8
130.4
125.1
112.2
169.08
133.11
131.86
130.83
130.44
125.11
112.18
f1 (ppm)

Figure S1. gNOESY Spectrum for **10b**

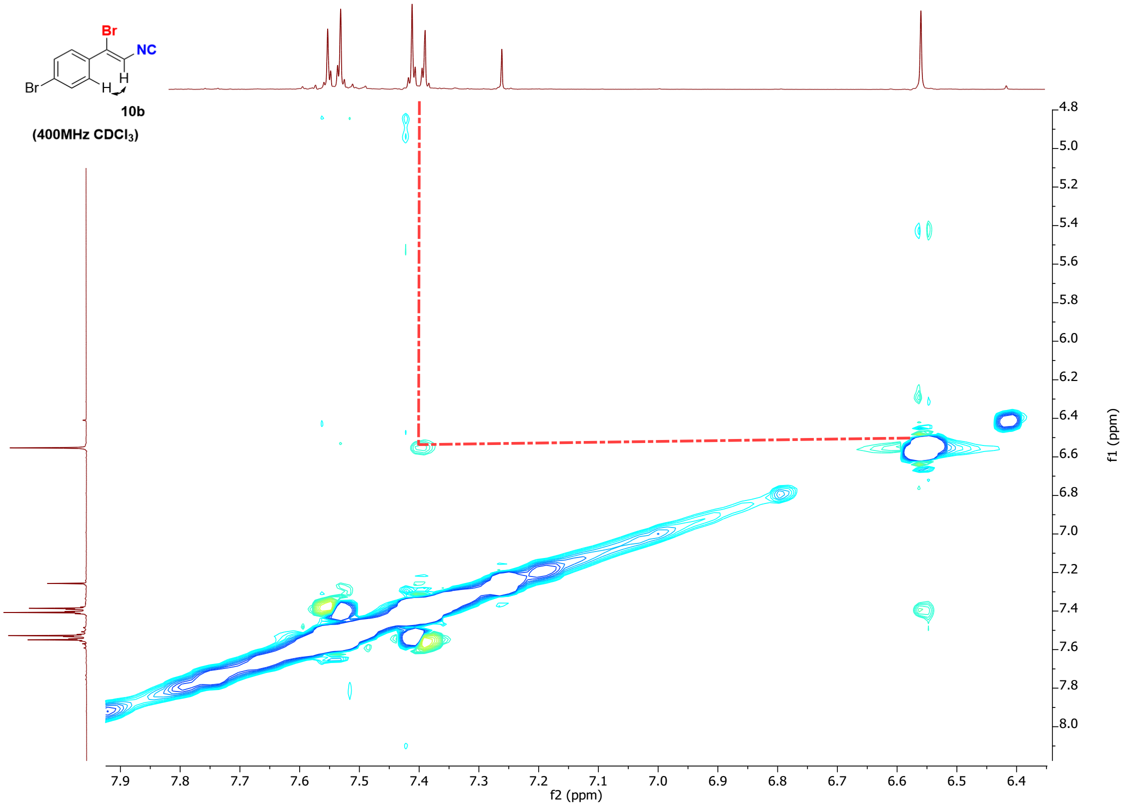

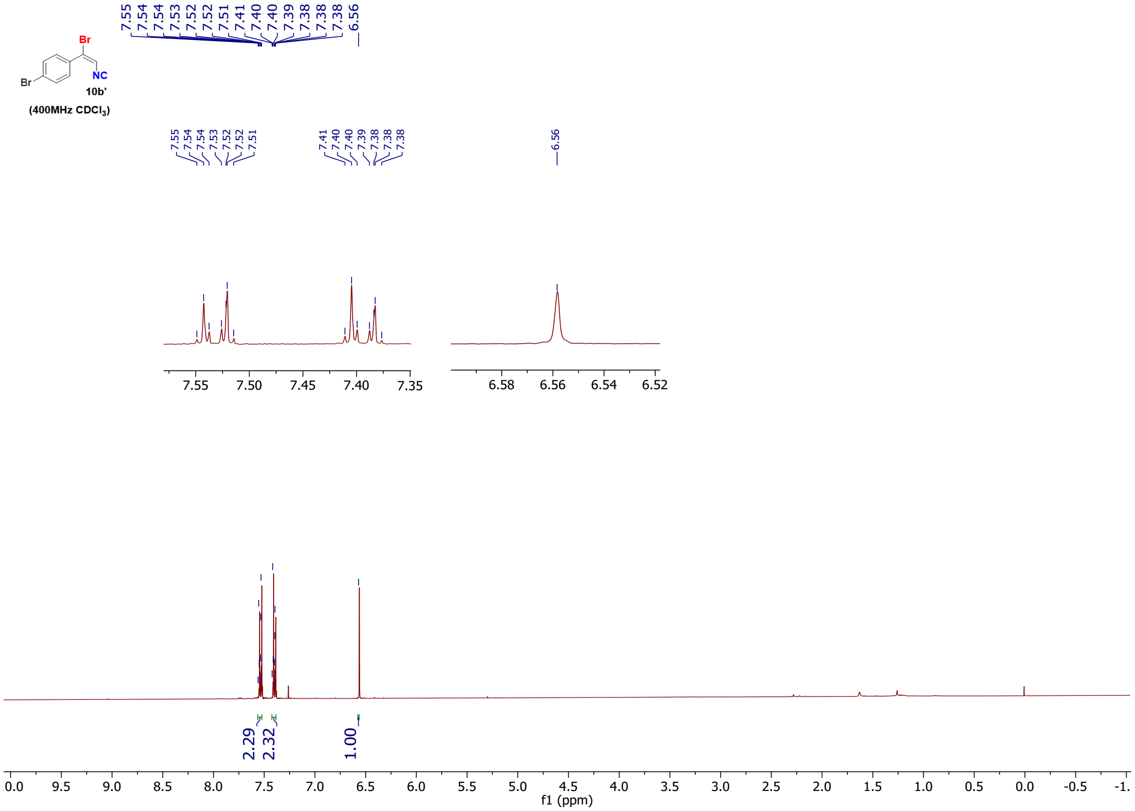
Br
Br
NC
10b'
(400MHz CDCl3)
f1 (ppm)

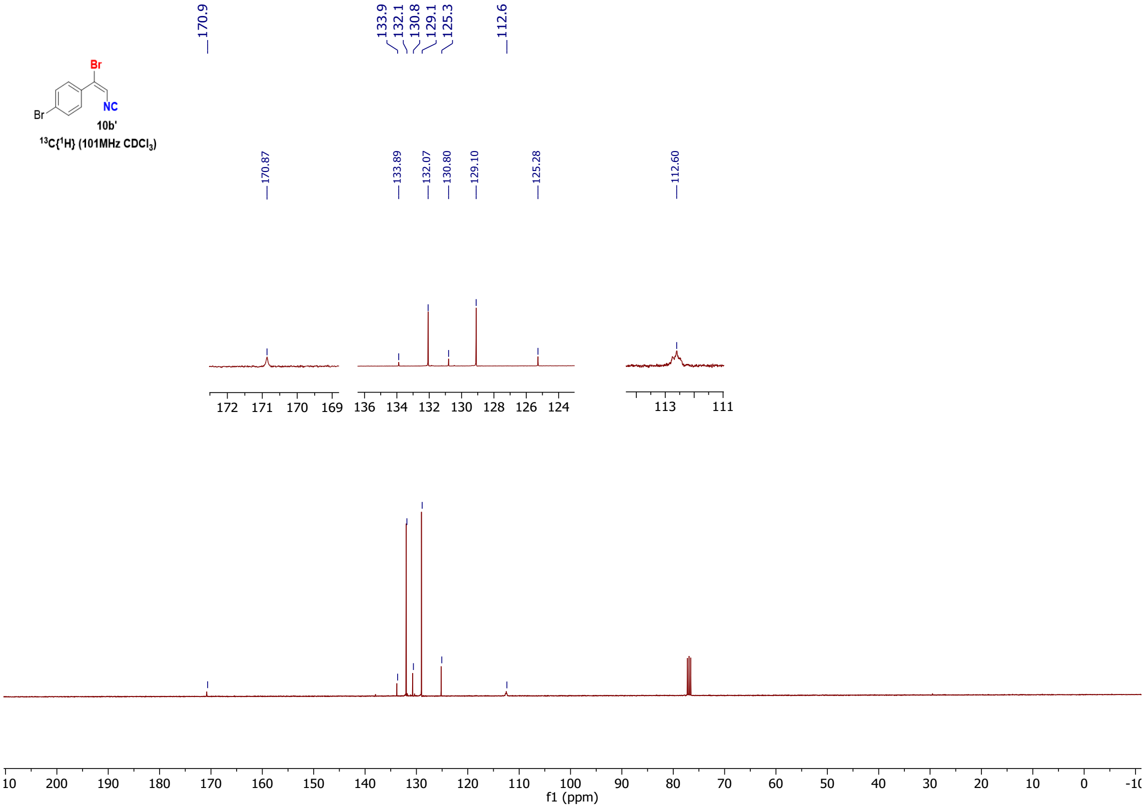
Br
Br
NC
10b'
13C{1H} (101MHz CDCl3)
f1 (ppm)

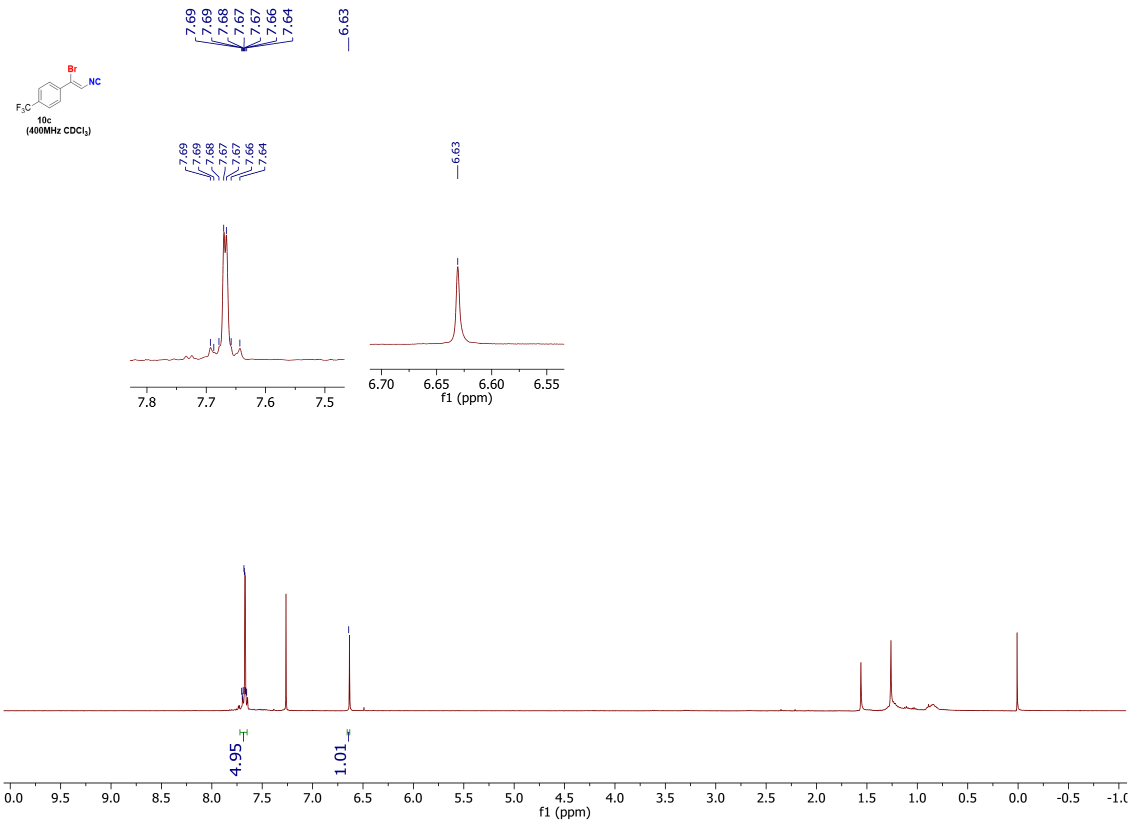

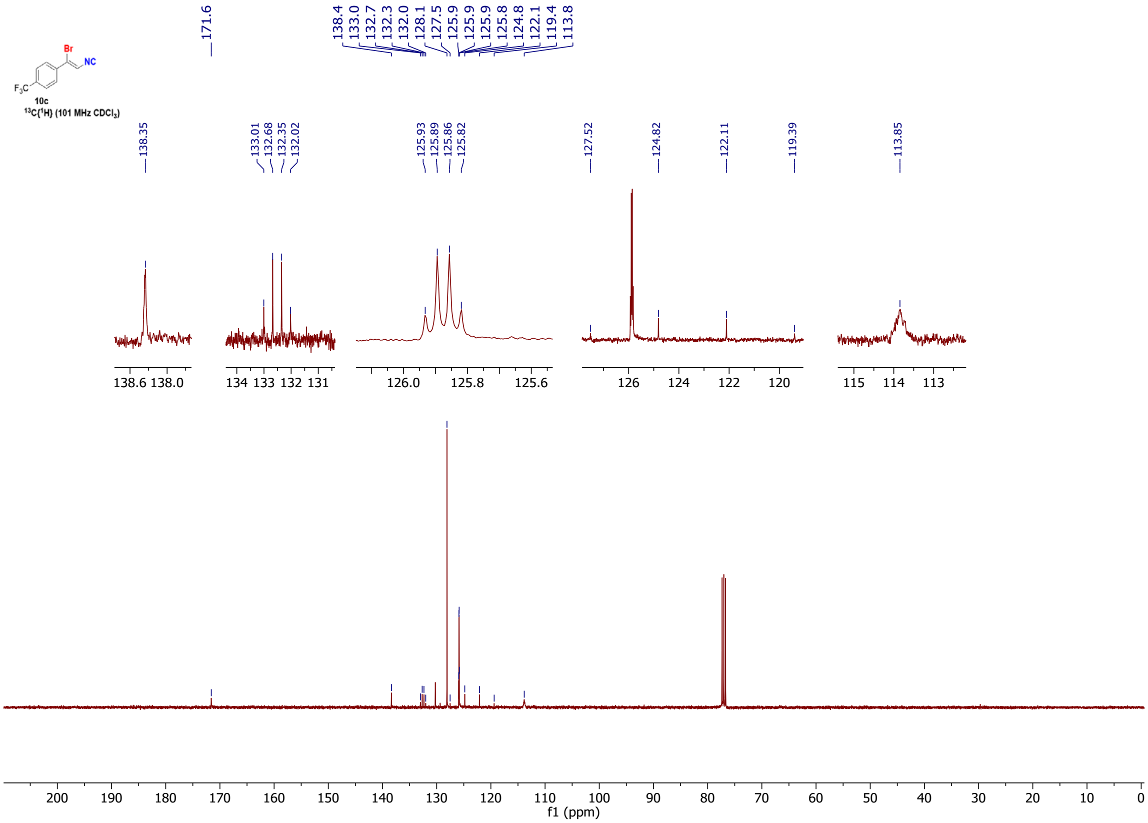

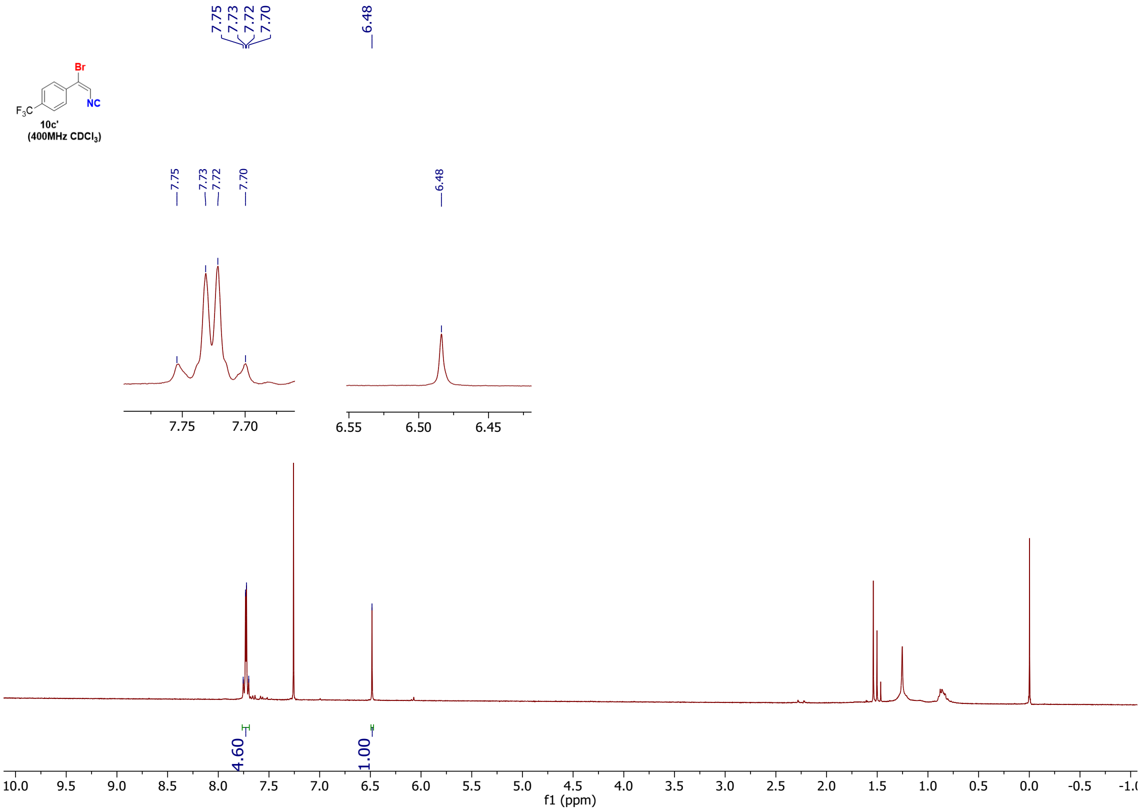

7.75
7.73
7.72
7.70
6.48
10c'
(400MHz CDCl3)
4.60
1.00
f1 (ppm)

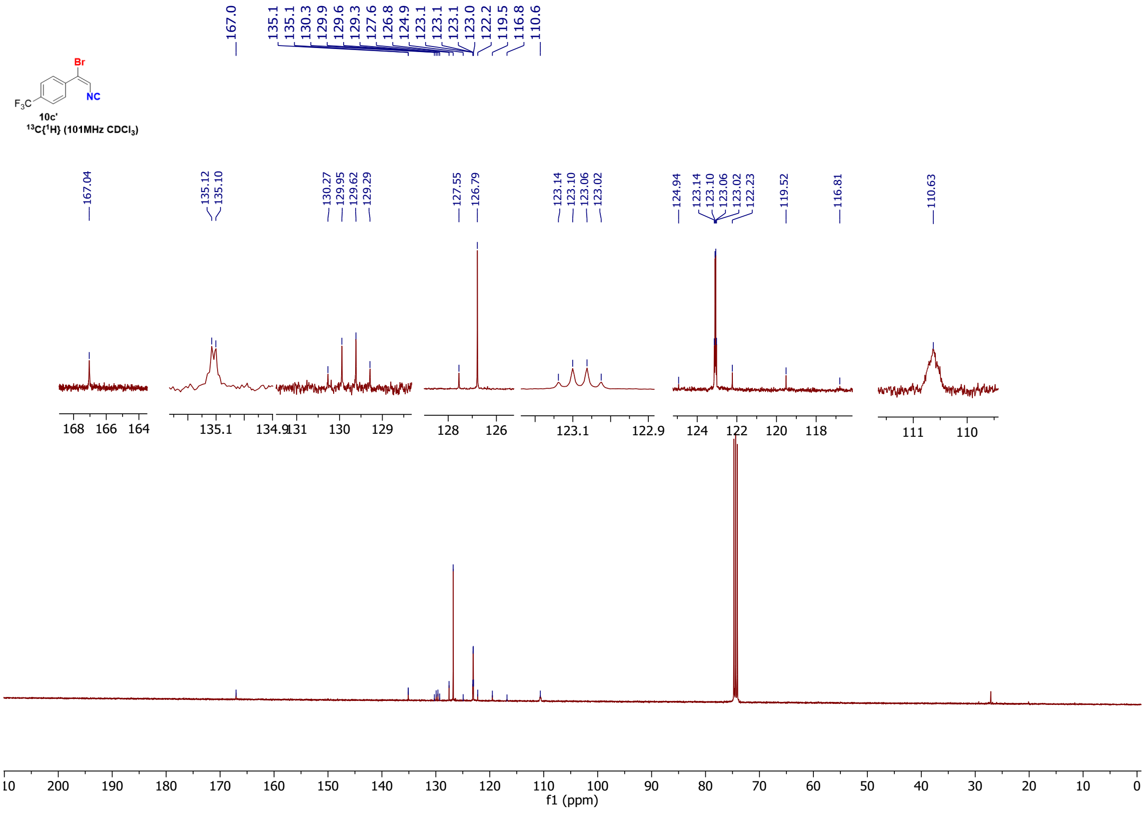

167.0
135.1
135.1
130.3
129.9
129.6
129.3
127.6
126.8
124.9
123.1
123.1
123.1
123.0
122.2
119.5
116.8
110.6
10c'
13C{1H} (101MHz CDCl3)
f1 (ppm)

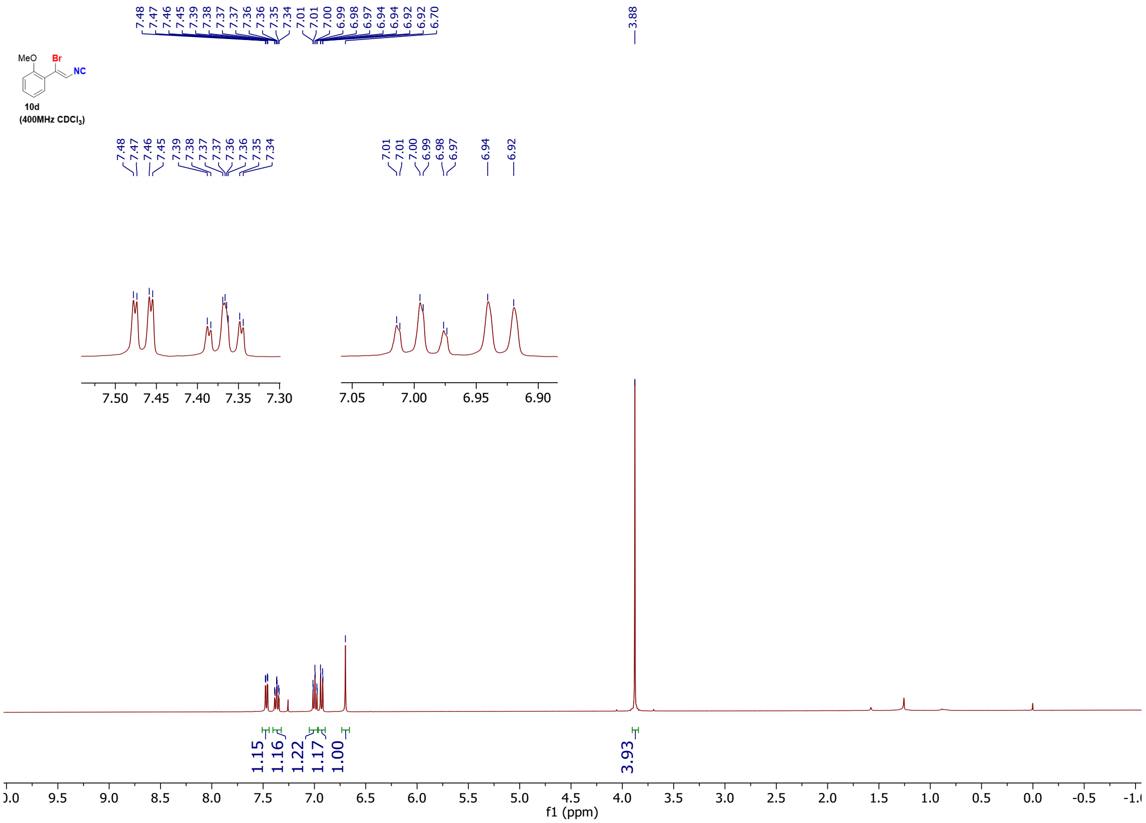
MeO
Br
NC
10d
(400MHz CDCl3)
3.88
1.15
1.16
1.22
1.17
1.00
3.93
f1 (ppm)

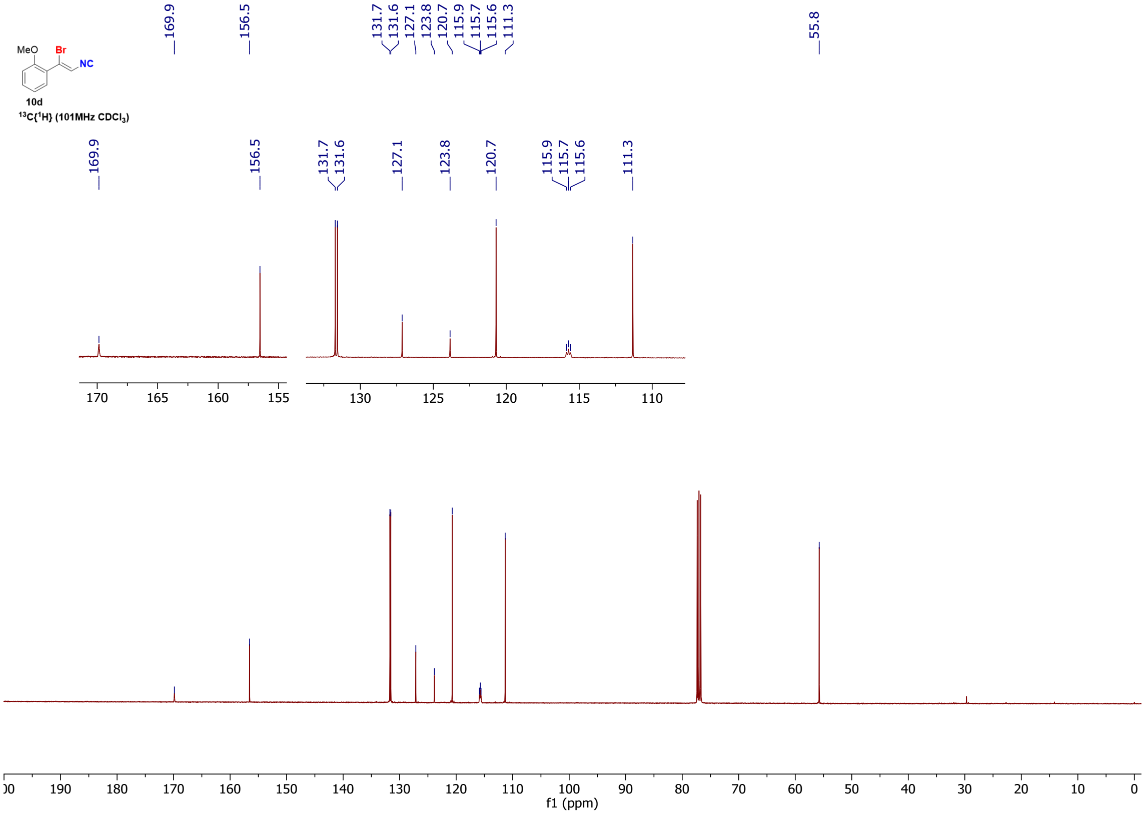
MeO
Br
NC
10d
13C{1H} (101MHz CDCl3)
169.9
156.5
131.7
131.6
127.1
123.8
120.7
115.9
115.7
115.6
111.3
55.8
f1 (ppm)

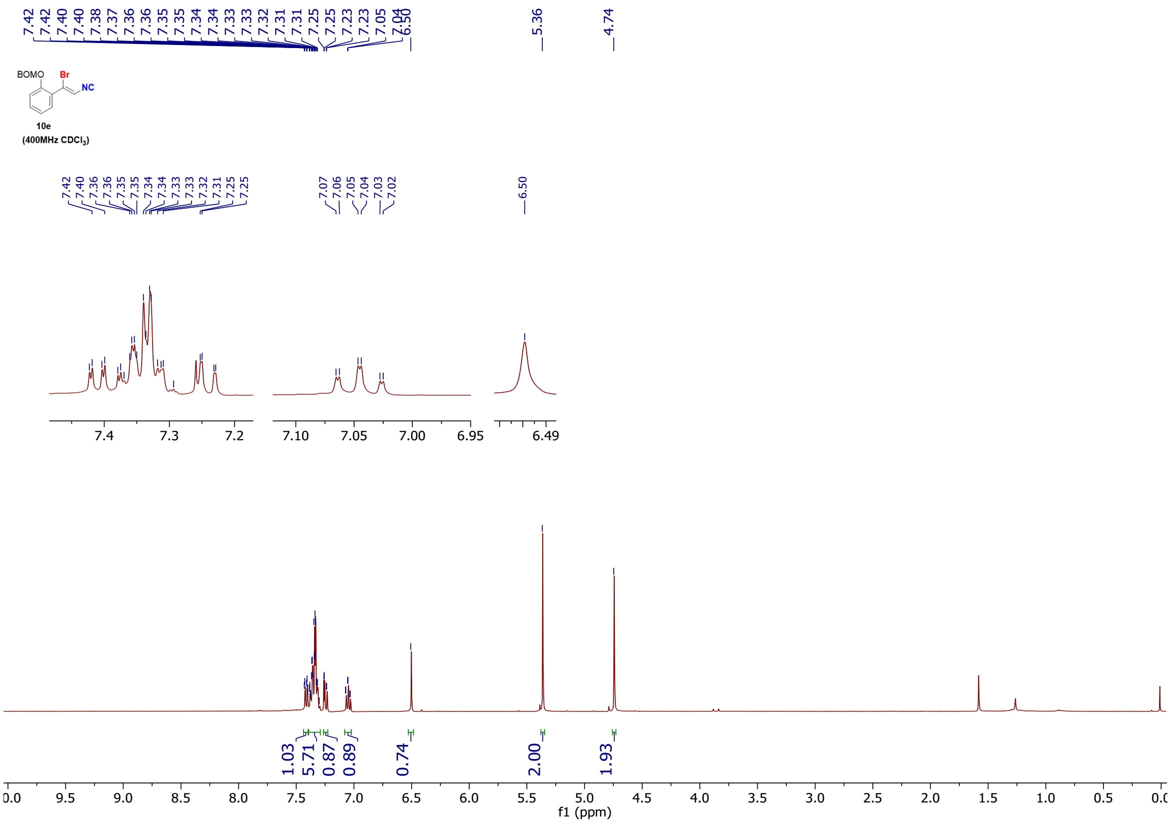
BOMO
Br
NC
10e
(400MHz CDCl3)
1.03
5.71
0.87
0.89
0.74
2.00
1.93
f1 (ppm)

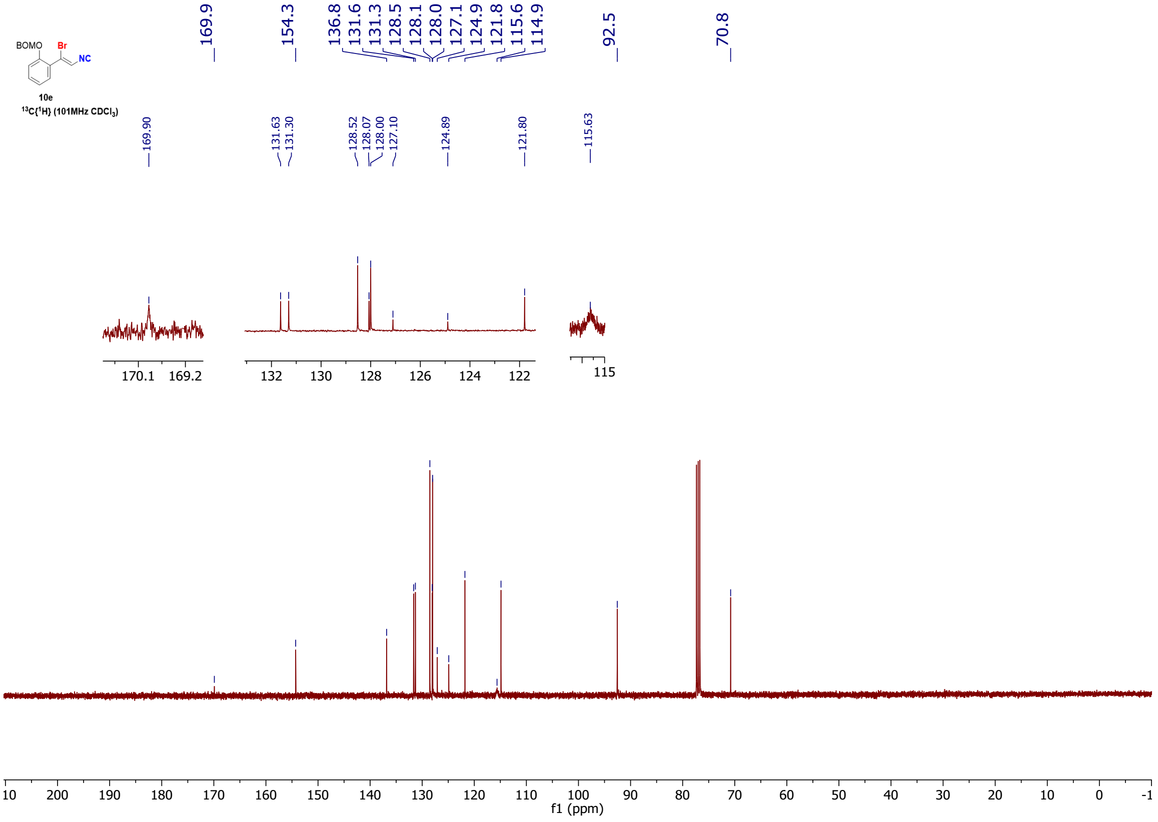
BOMO
Br
NC
10e
13C{1H} (101MHz CDCl3)
169.9
154.3
136.8
131.6
131.3
128.5
128.1
128.0
127.1
124.9
121.8
115.6
114.9
92.5
70.8
f1 (ppm)

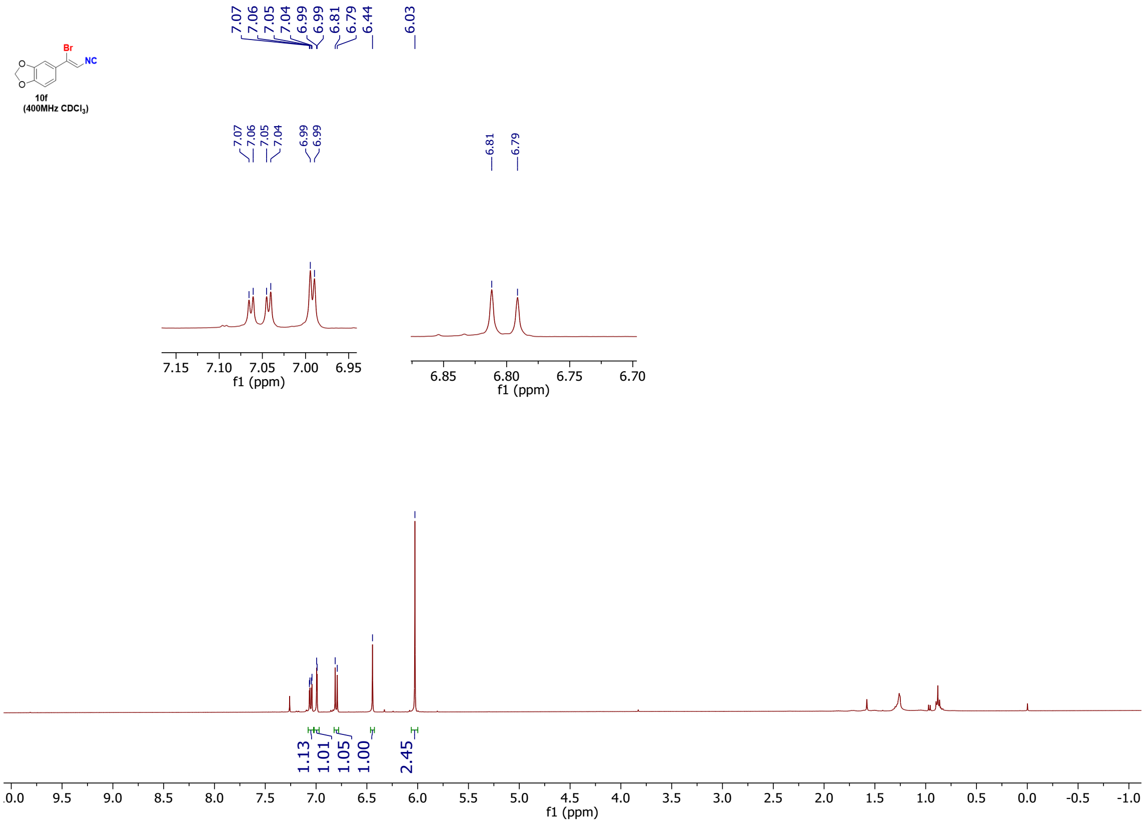

Br
NC
10f
(400MHz CDCl3)
7.07
7.06
7.05
7.04
6.99
6.99
6.81
6.79
6.44
6.03
1.13
1.01
1.05
1.00
2.45
f1 (ppm)

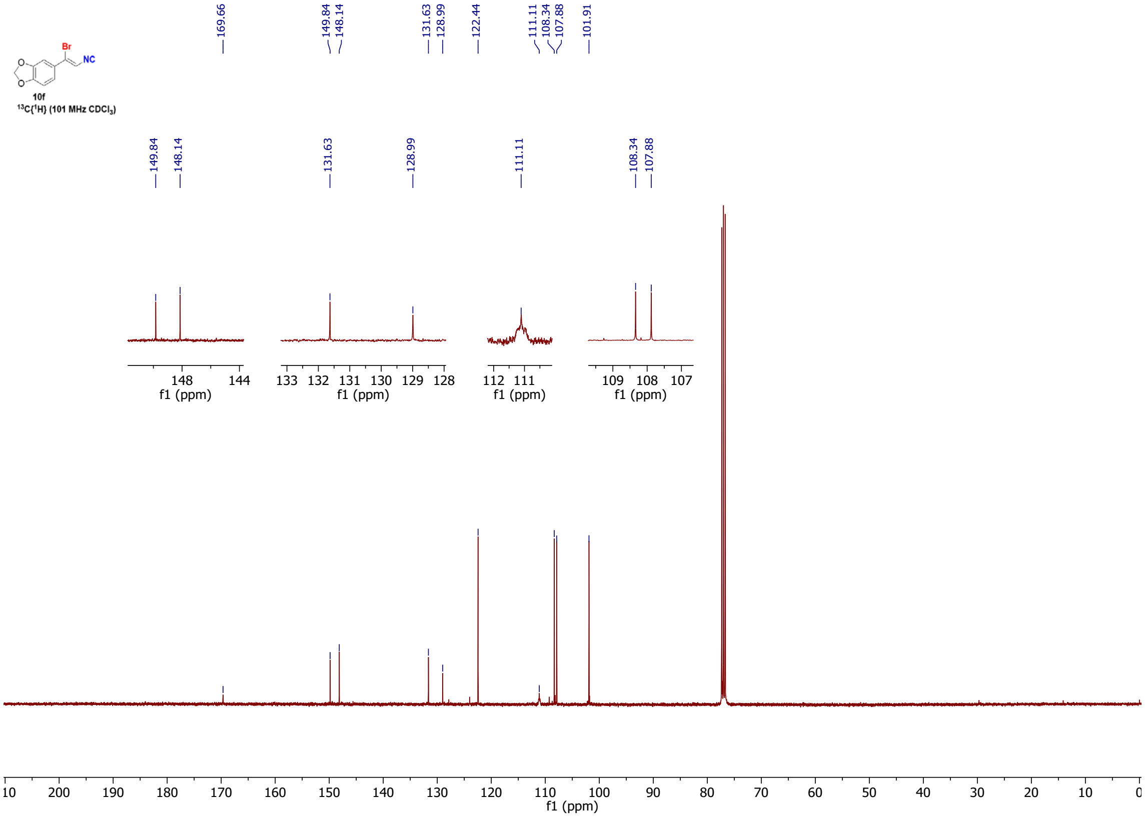

Br
NC
10f
13C{1H} (101 MHz CDCl3)
169.66
149.84
148.14
131.63
128.99
122.44
111.11
108.34
107.88
101.91
f1 (ppm)

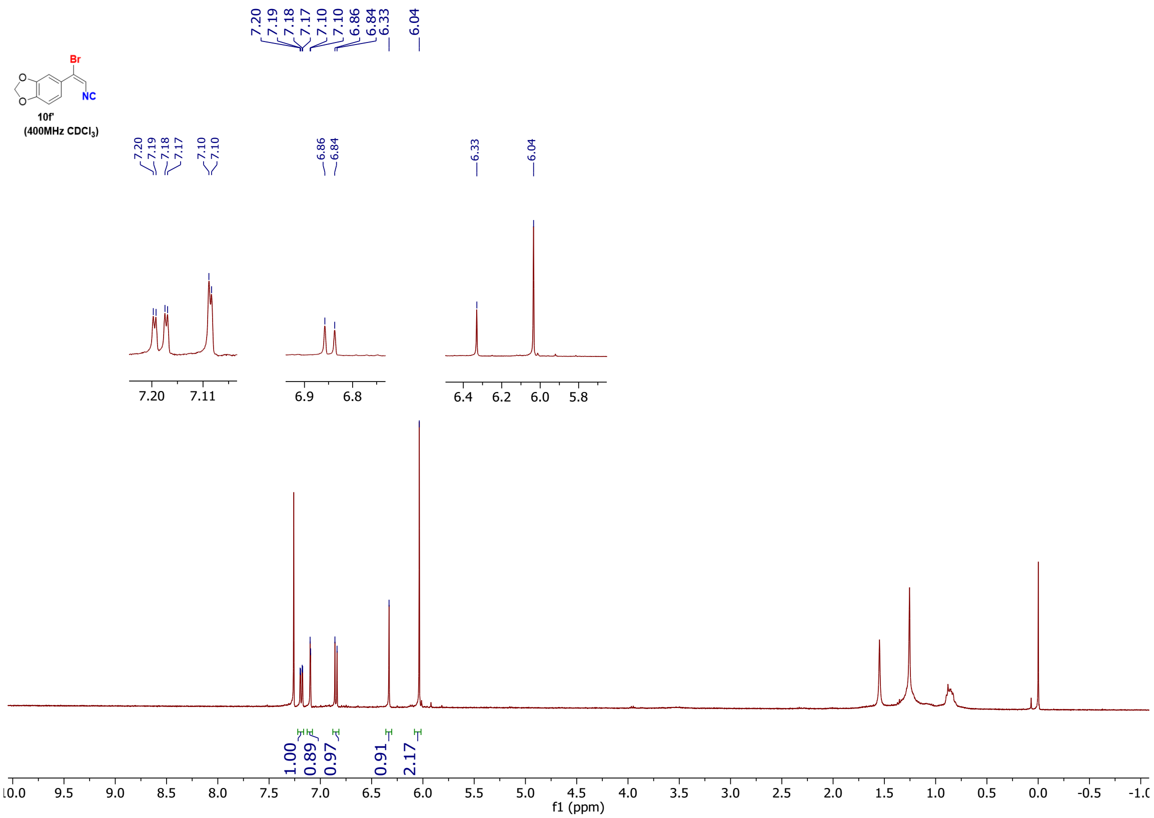

10f'
(400MHz CDCl3)
7.20
7.19
7.18
7.17
7.10
7.10
6.86
6.84
6.33
6.04
1.00
0.89
0.97
0.91
2.17
f1 (ppm)

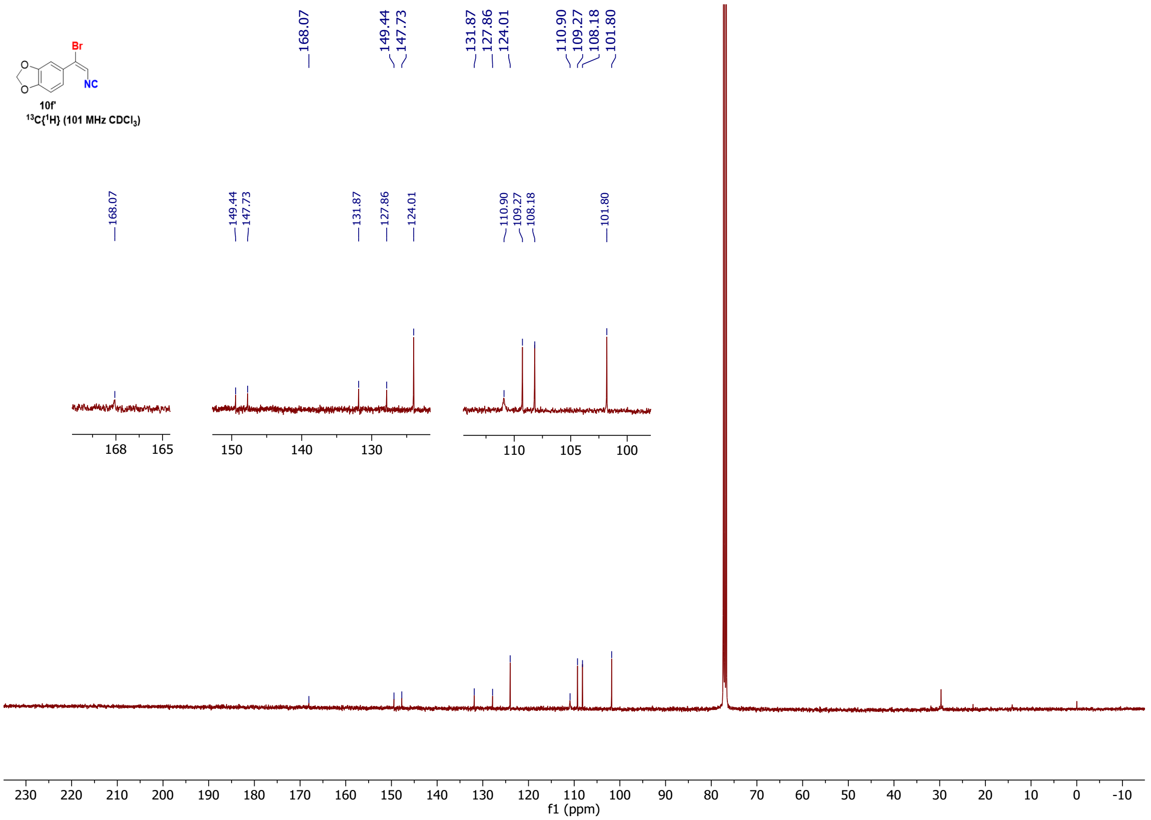

10f'
13C{1H} (101 MHz CDCl3)
168.07
149.44
147.73
131.87
127.86
124.01
110.90
109.27
108.18
101.80
f1 (ppm)

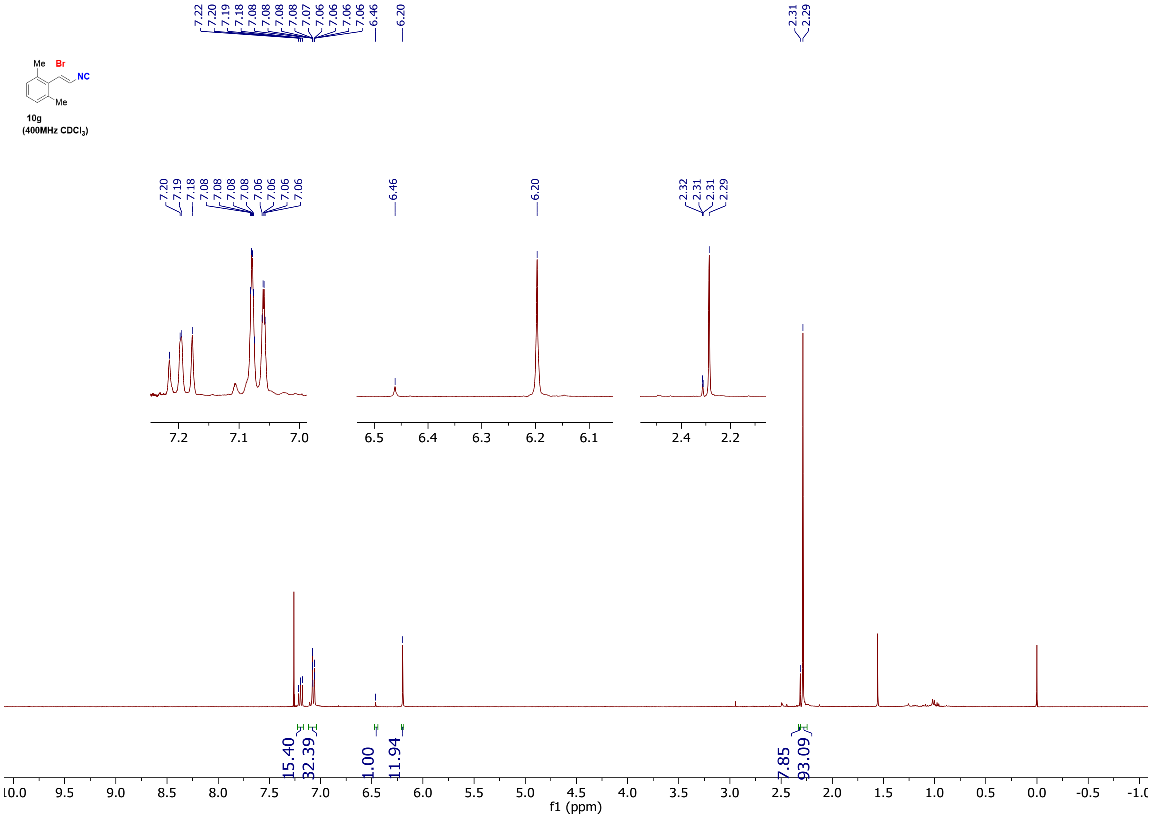
Me
Br
NC
Me
10g
(400MHz CDCl3)
7.22 7.20 7.19 7.18 7.08 7.08 7.08 7.08 7.07 7.06 7.06 7.06 7.06 6.46 6.20
2.31 2.29
15.40
32.39
1.00
11.94
7.85
93.09
f1 (ppm)

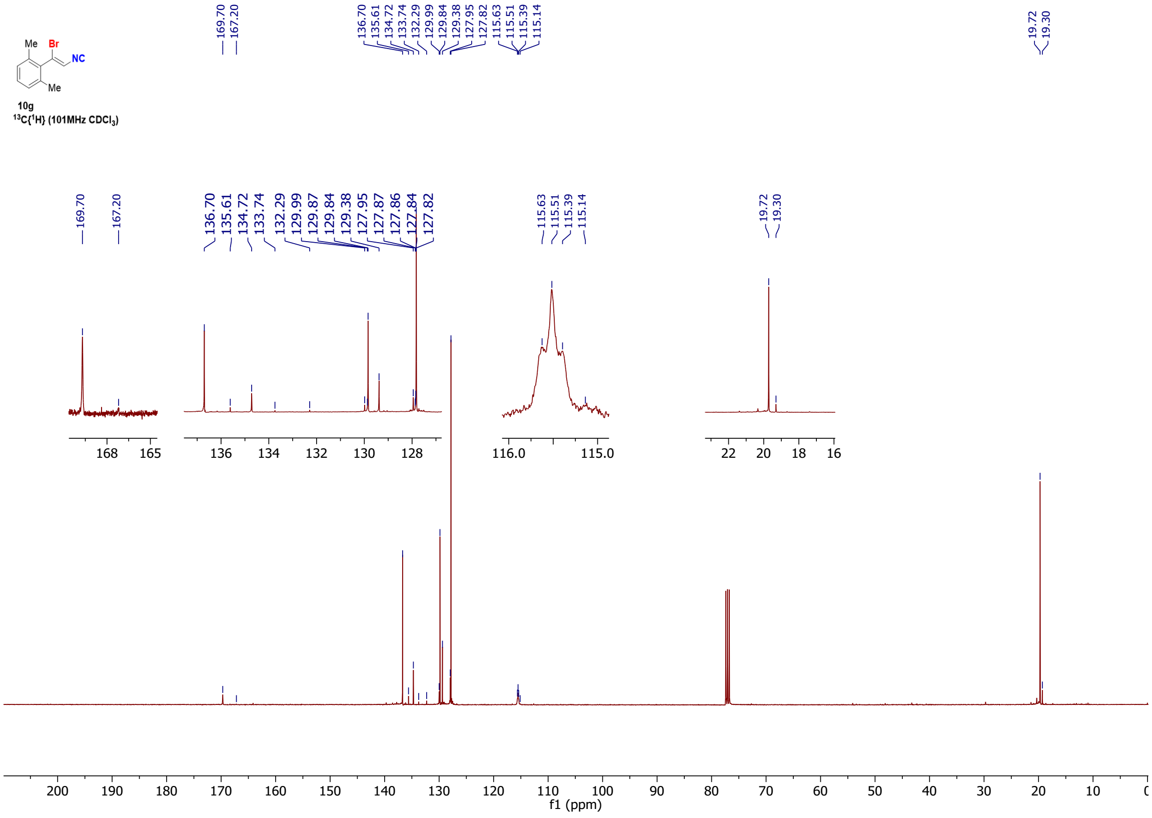
Me
Br
NC
Me
10g
13C{1H} (101MHz CDCl3)
169.70 167.20
136.70 135.61 134.72 133.74 132.29 129.99 129.84 129.38 127.95 127.82 115.63 115.51 115.39 115.14
19.72 19.30
f1 (ppm)

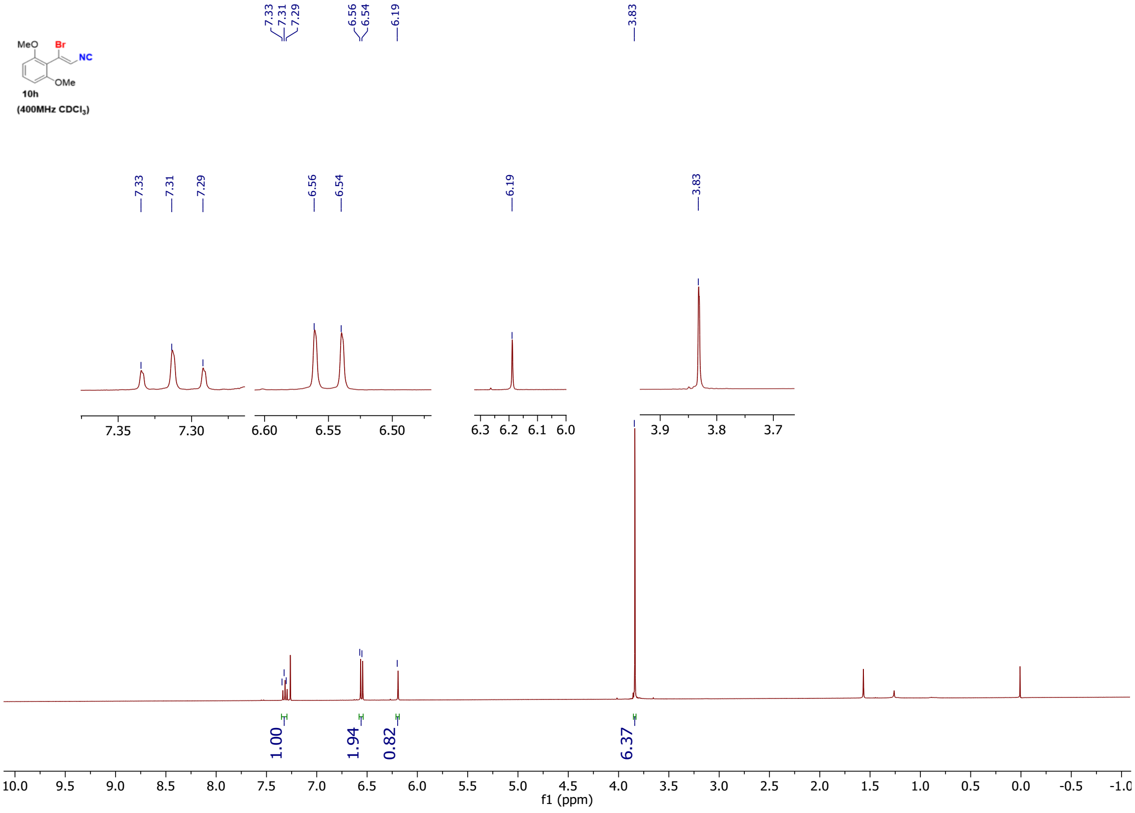
10h
(400MHz CDCl3)
f1 (ppm)

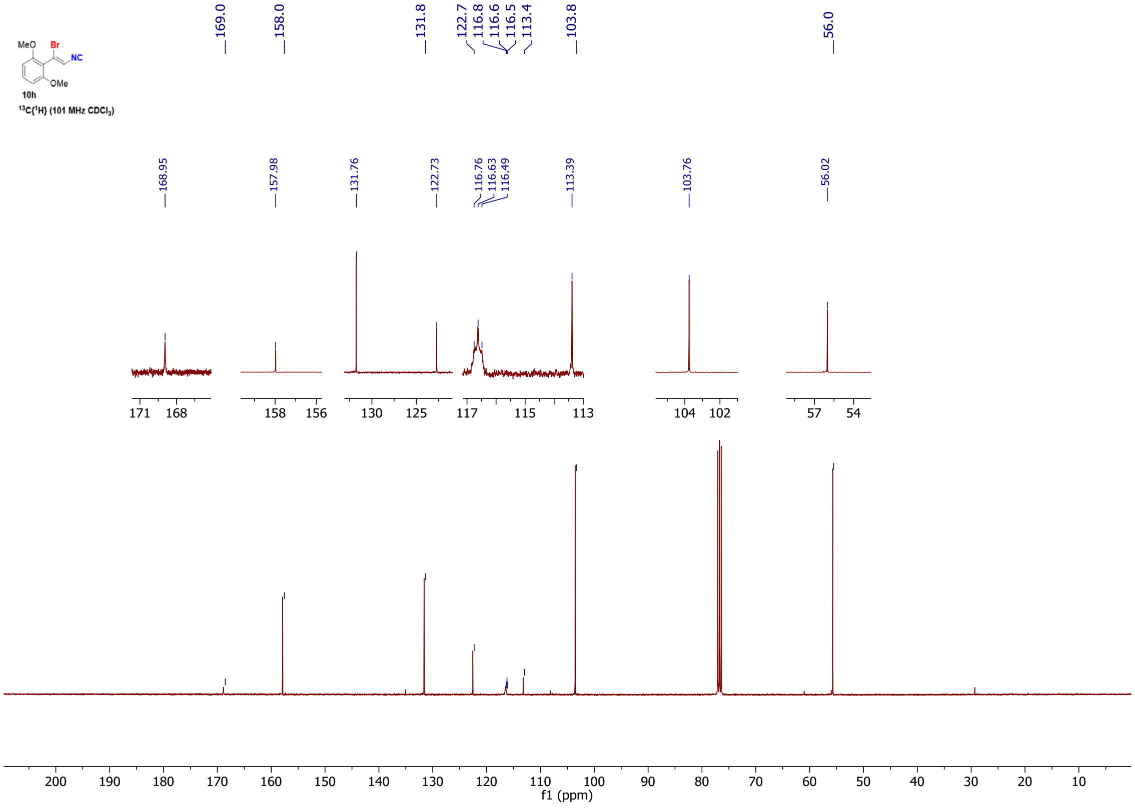
10h
13C{1H} (101 MHz CDCl3)
f1 (ppm)

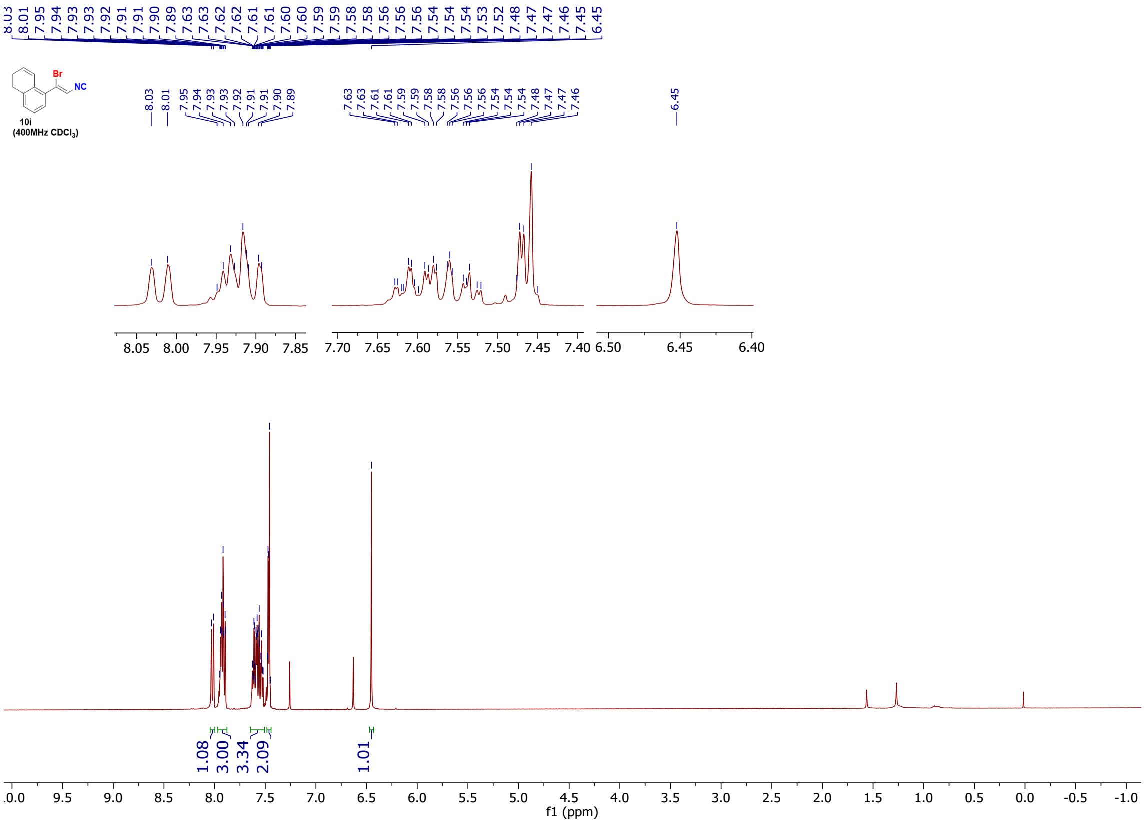
10i
(400MHz CDCl3)
1.08
3.00
3.34
2.09
1.01
f1 (ppm)

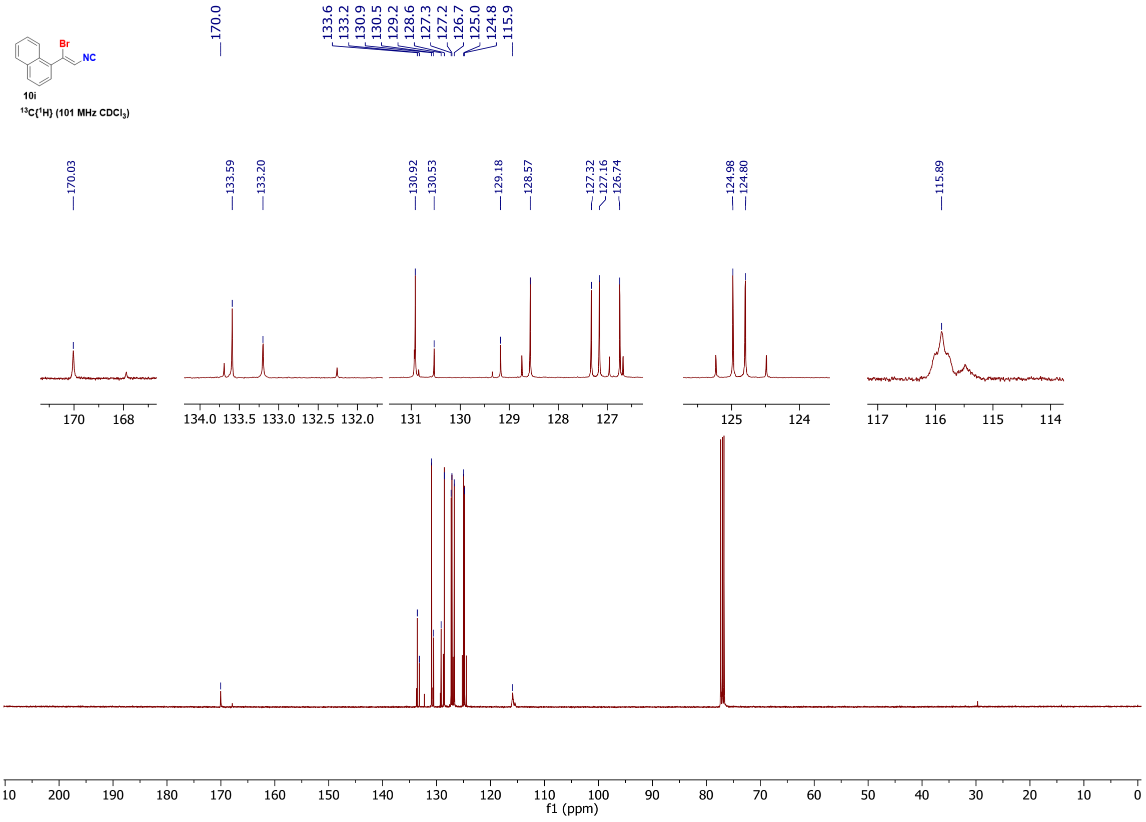
10i
13C{1H} (101 MHz CDCl3)
170.0
133.6
133.2
130.9
130.5
129.2
128.6
127.3
127.2
126.7
125.0
124.8
115.9
f1 (ppm)

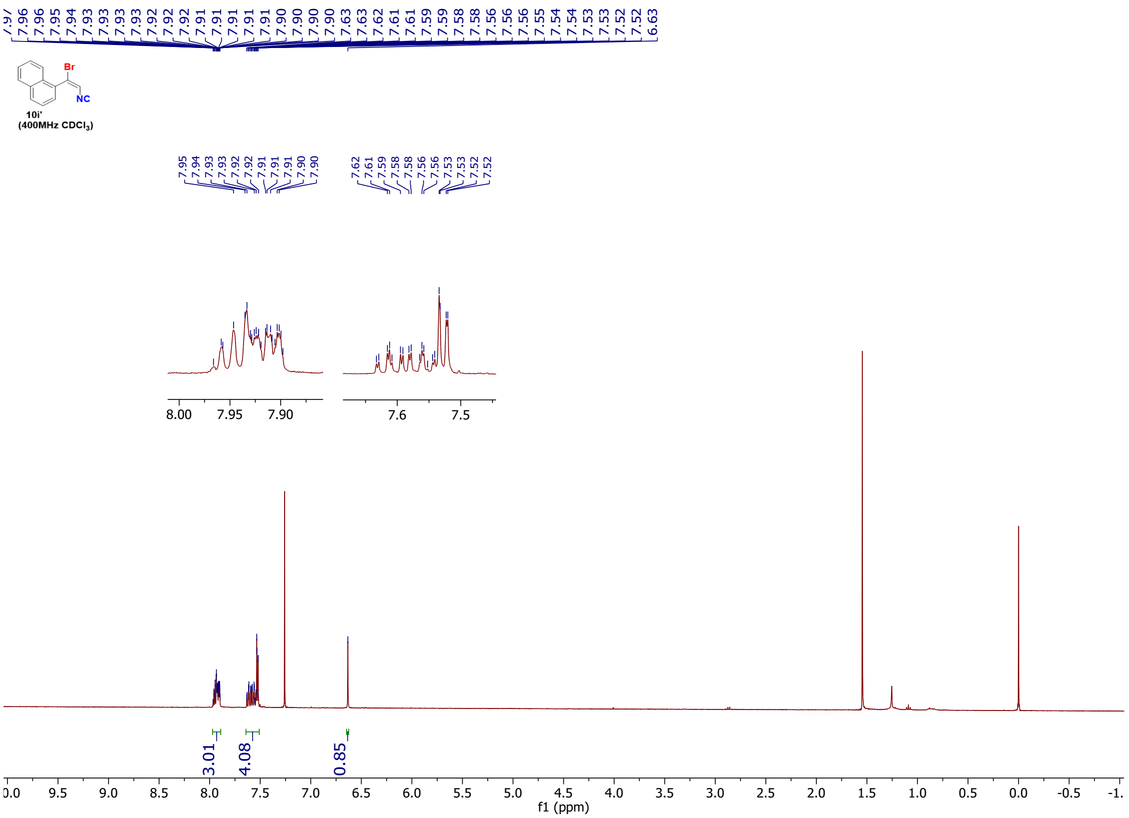

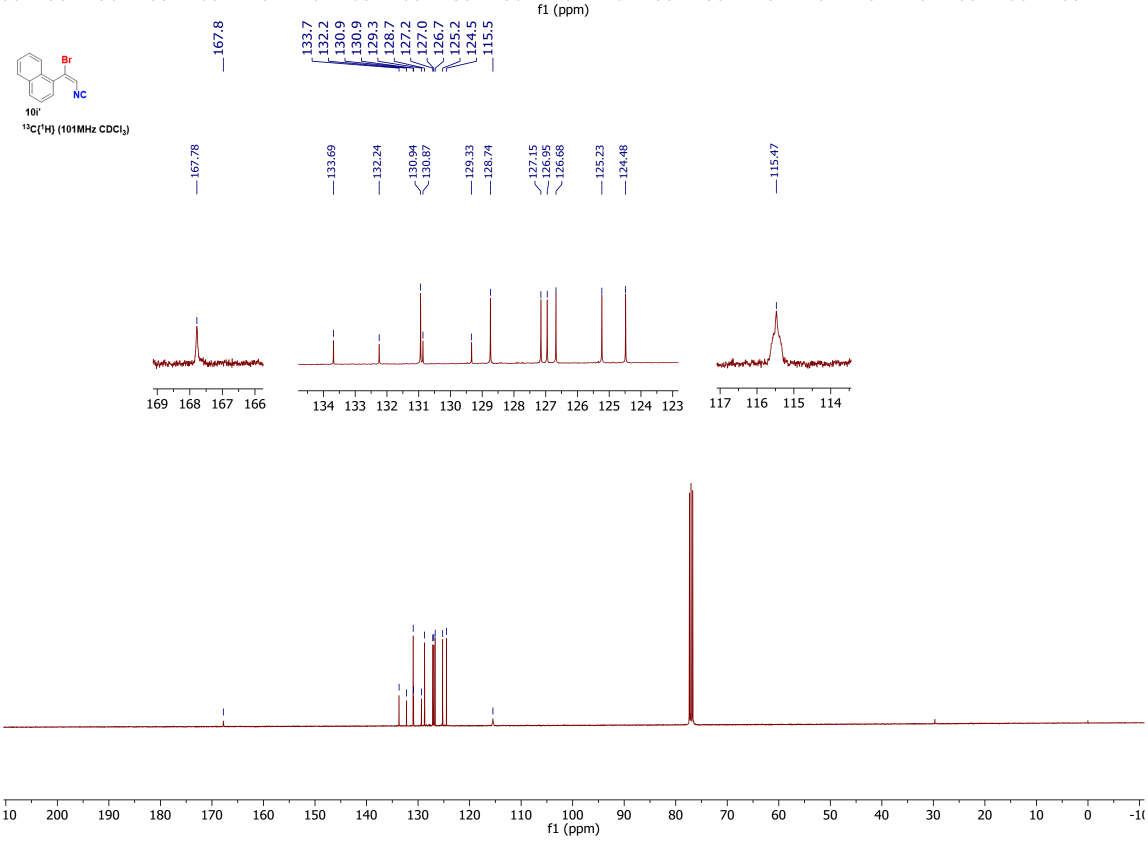

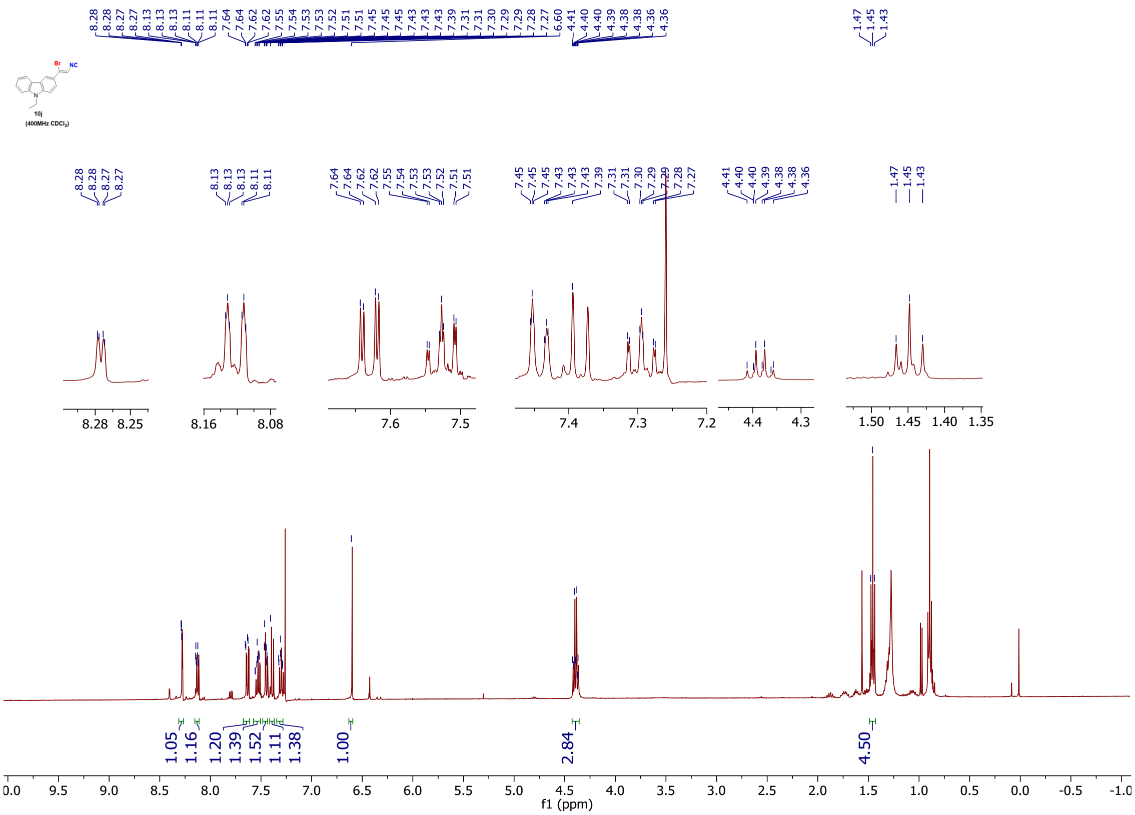

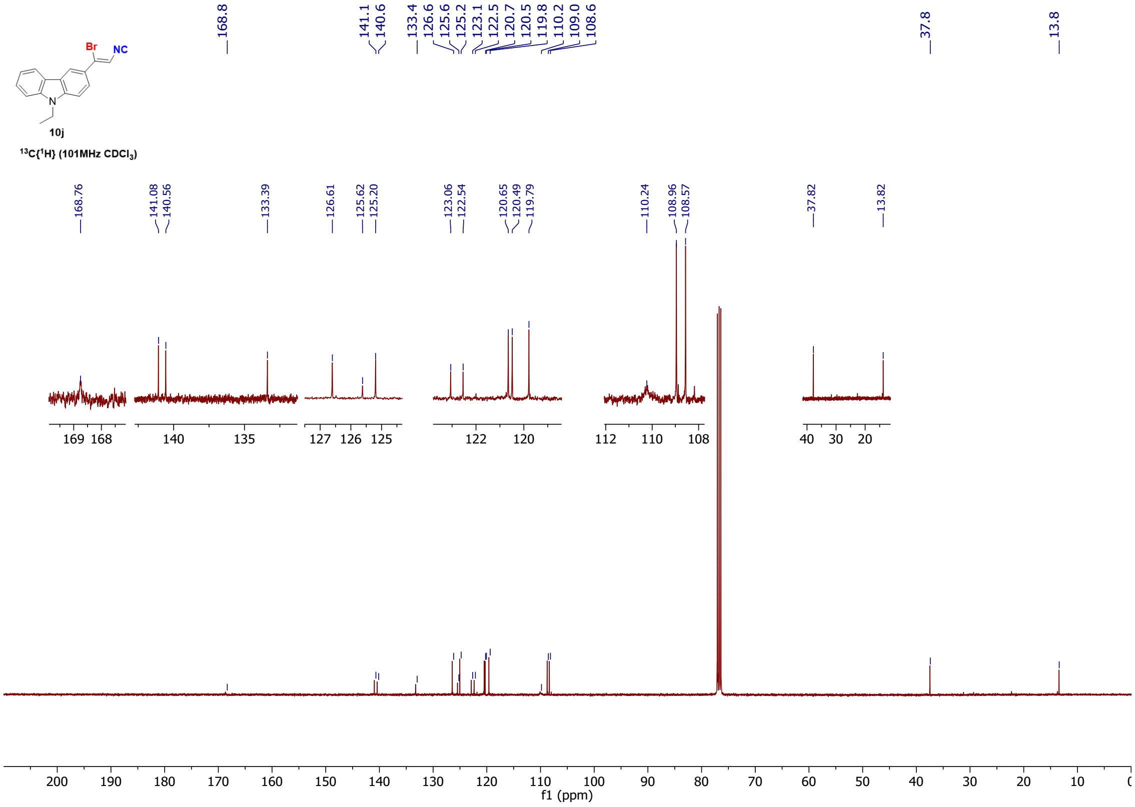

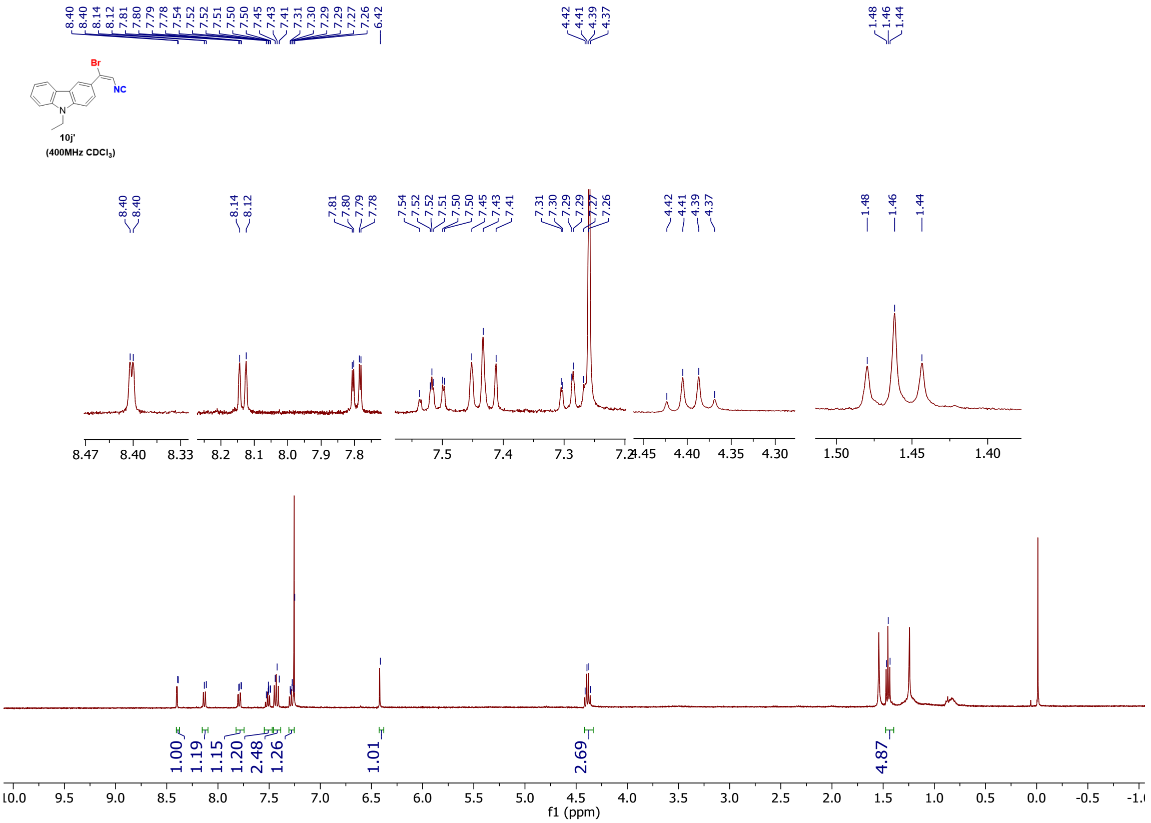
10j'
(400MHz CDCl3)
f1 (ppm)

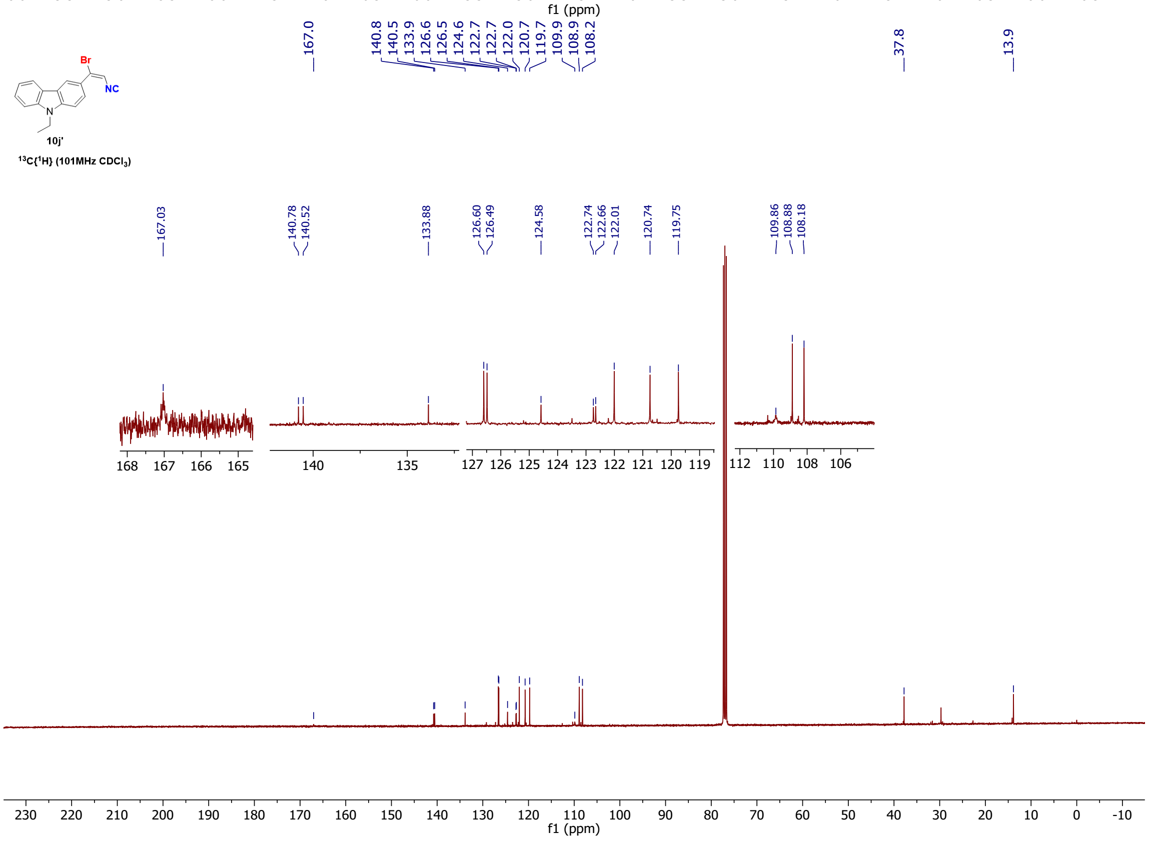
10j'
13C{1H} (101MHz CDCl3)
f1 (ppm)

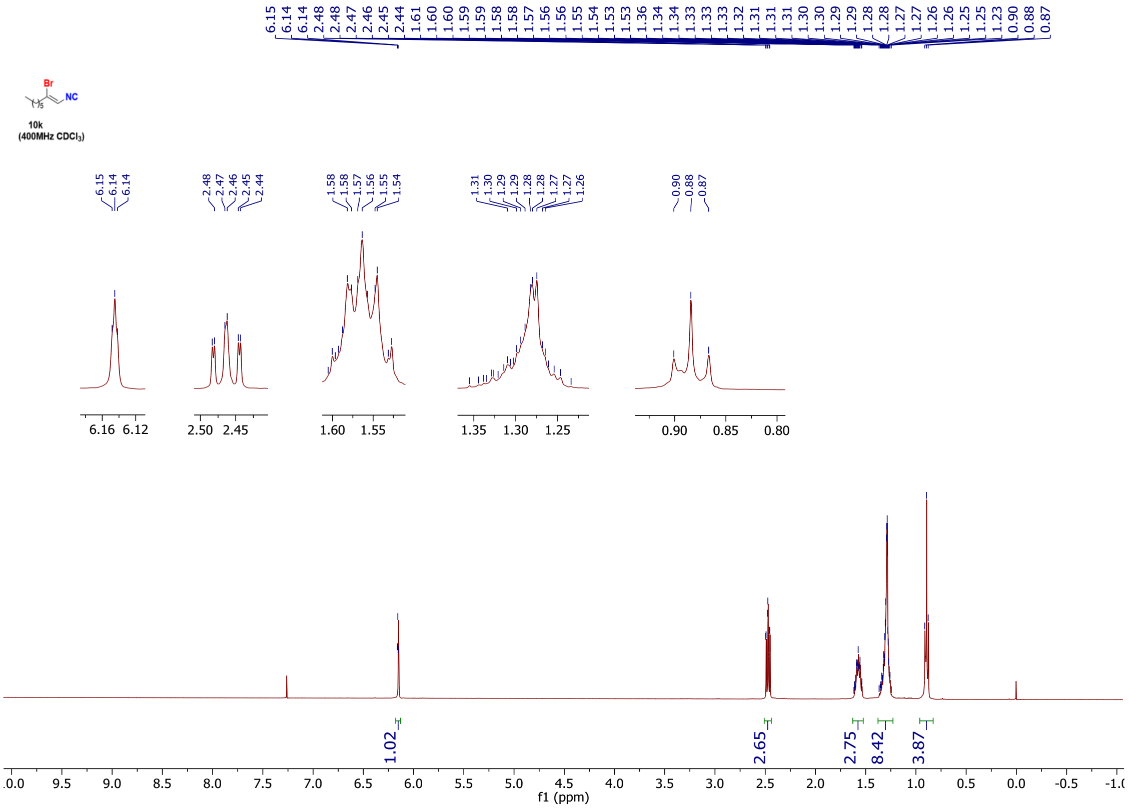
10k
(400MHz CDCl3)
f1 (ppm)

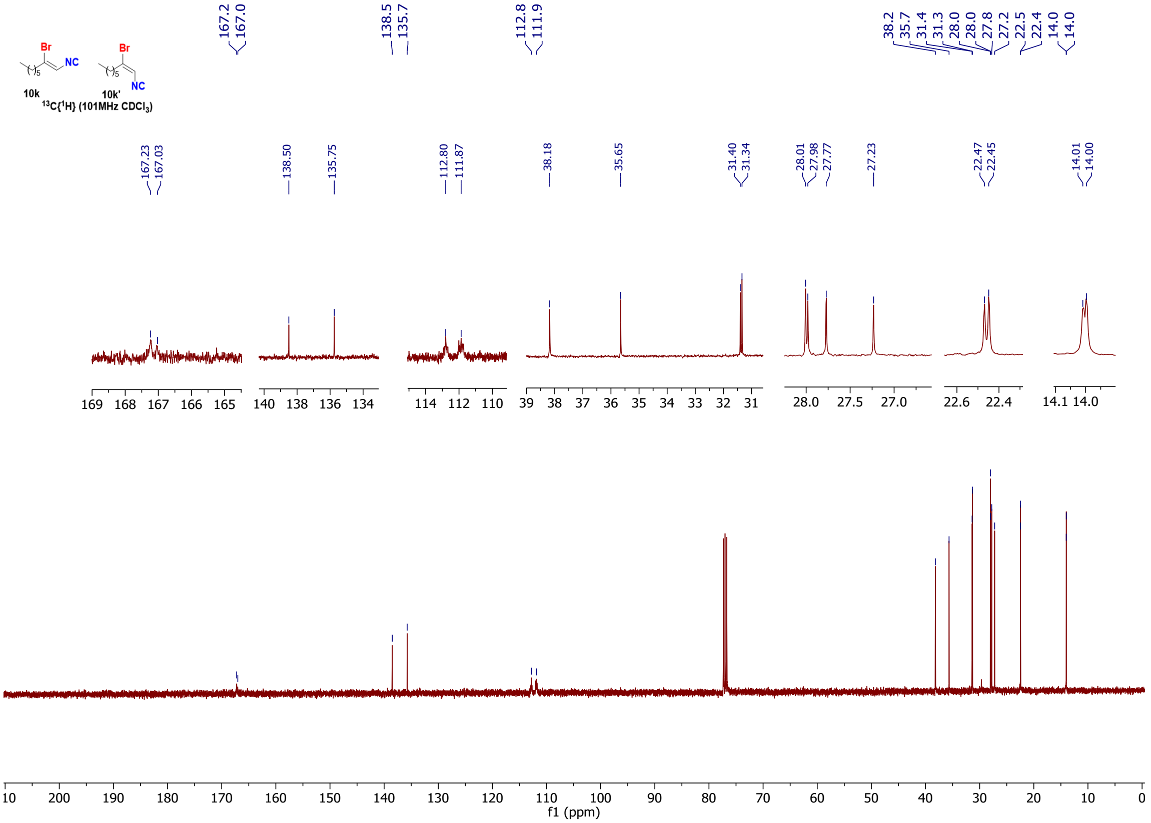
10k
10k'
13C{1H} (101MHz CDCl3)
f1 (ppm)

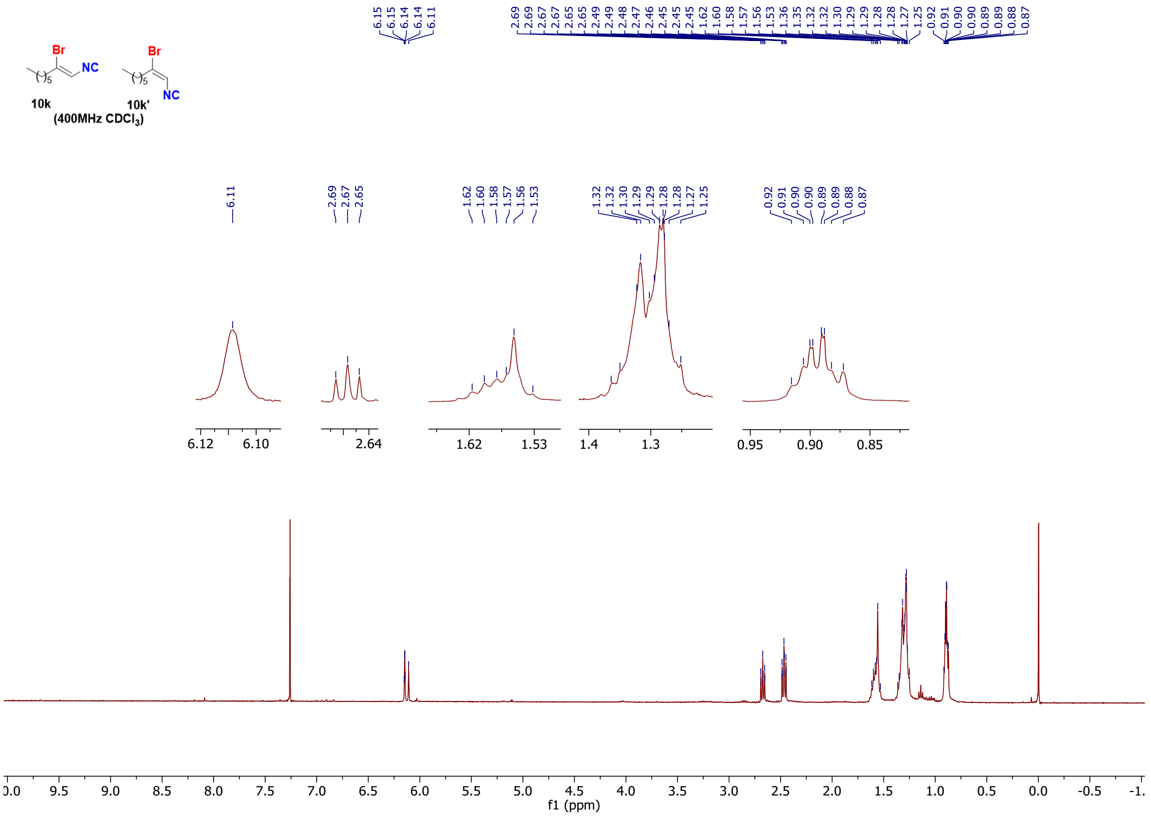

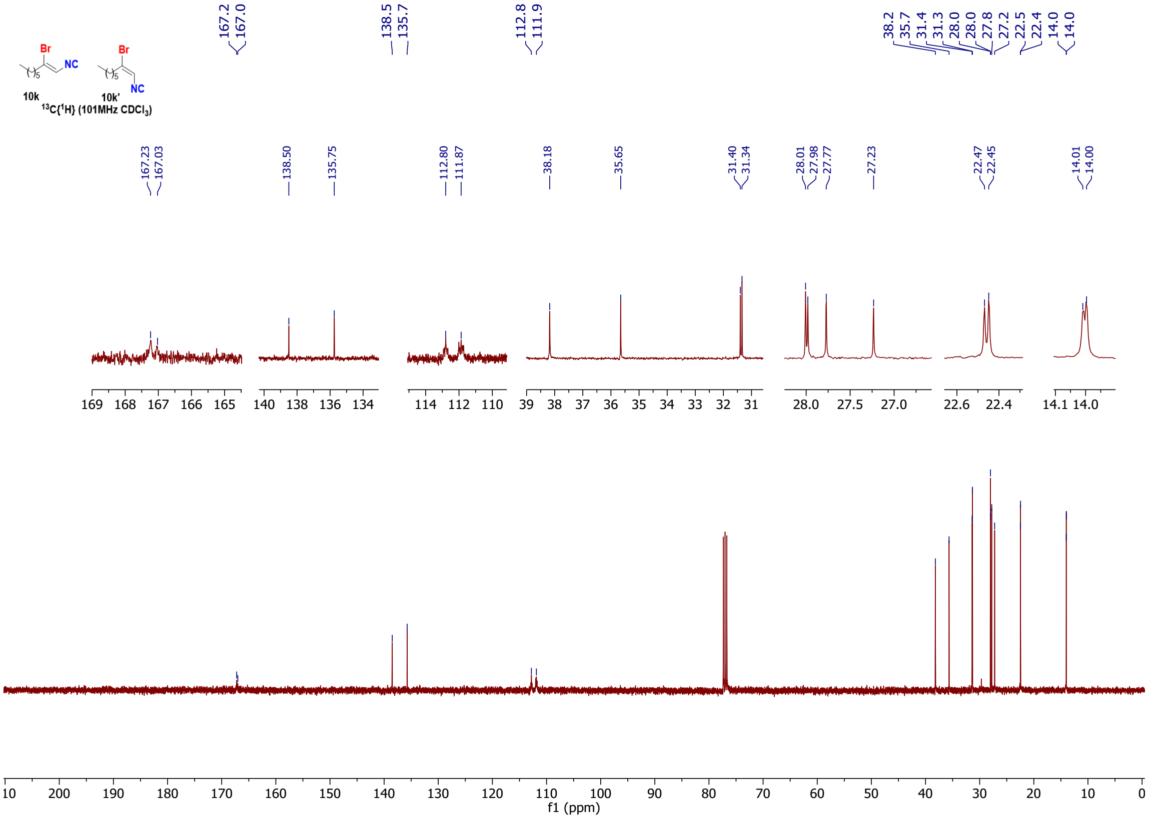

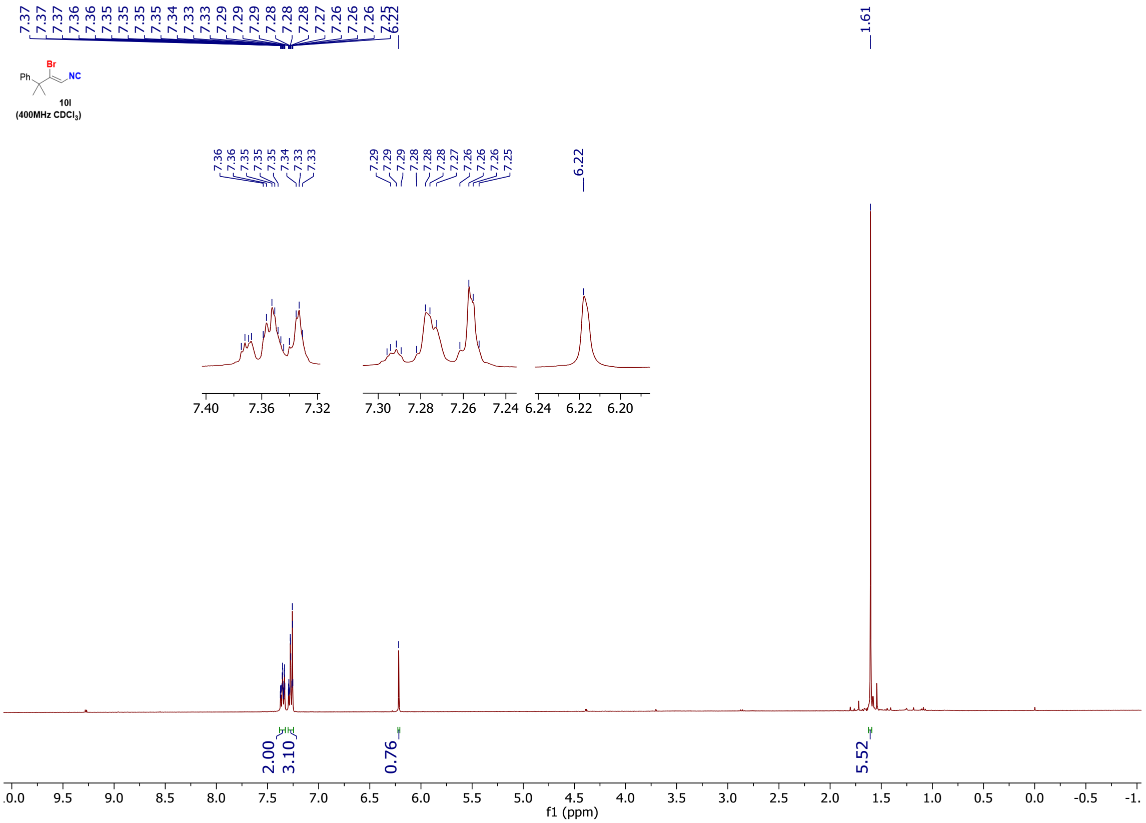
Br
Ph
NC
10l
(400MHz CDCl3)
f1 (ppm)

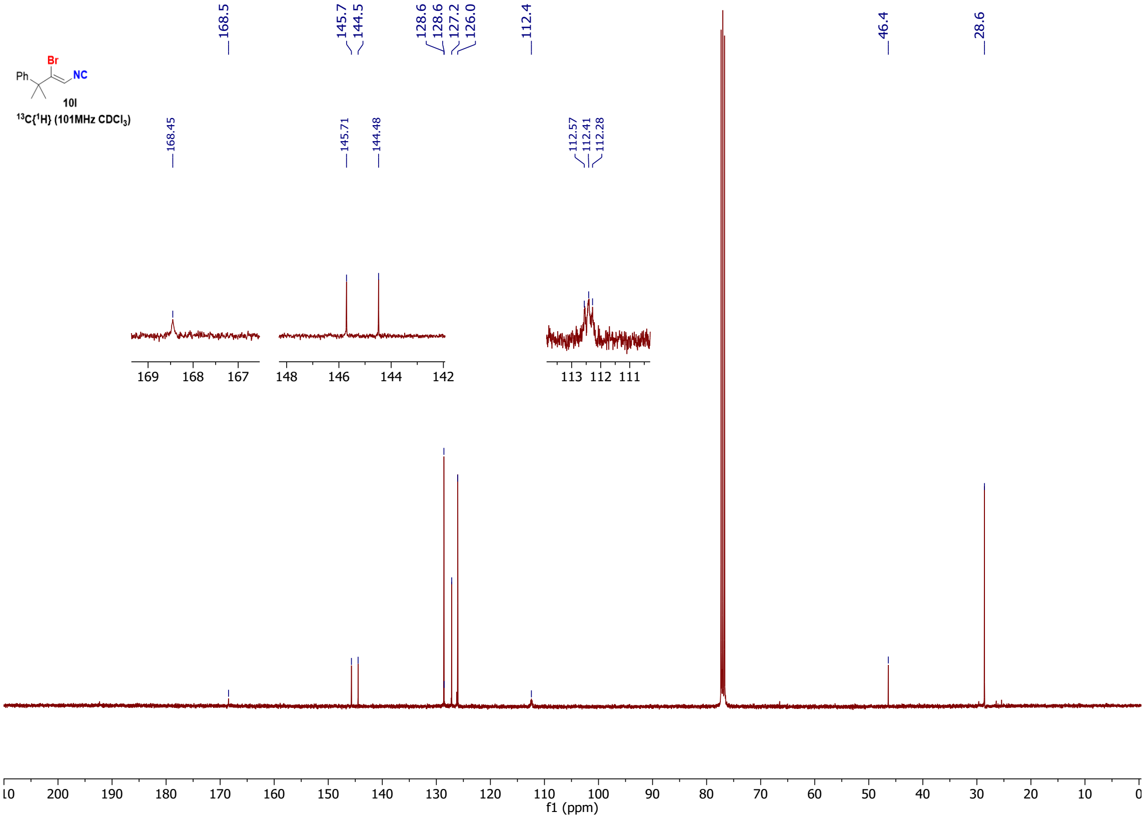
Br
Ph
NC
10l
13C{1H} (101MHz CDCl3)
f1 (ppm)

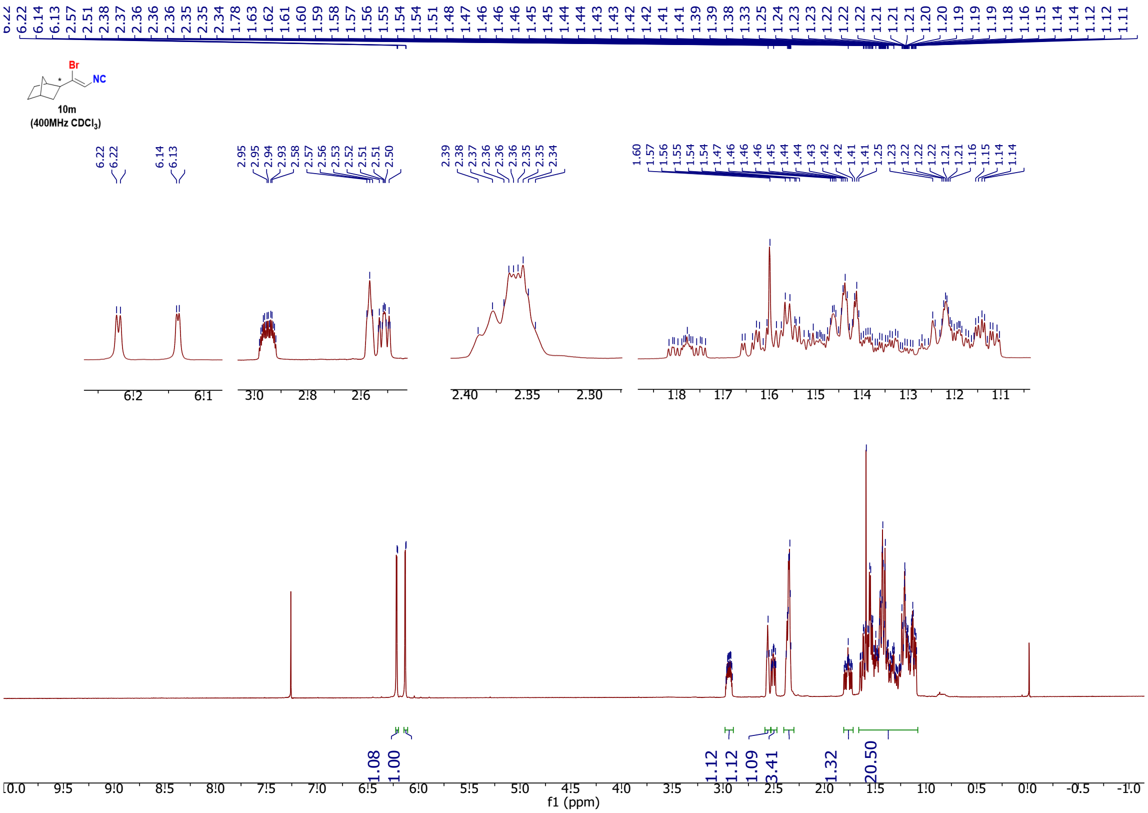
Br
NC
10m
(400MHz CDCl$_3$)
f1 (ppm)

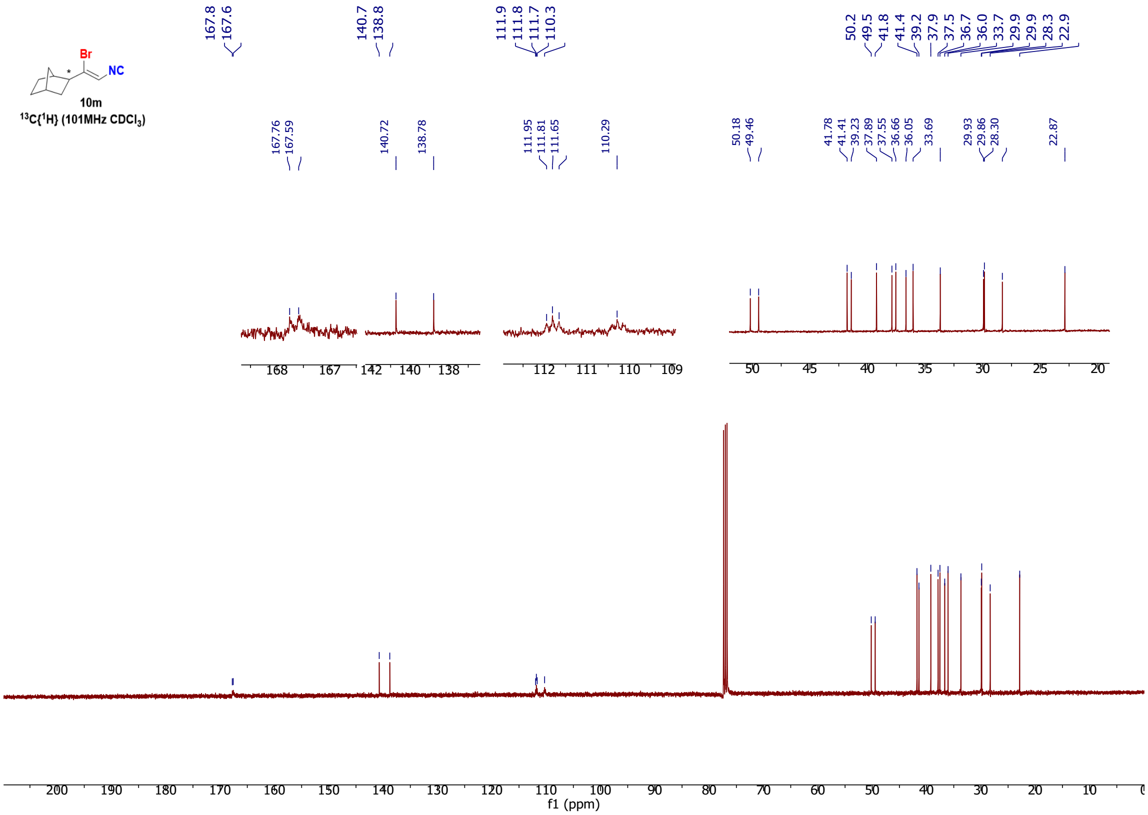
Br
NC
10m
$^{13}C\{^1H\}$ (101MHz CDCl$_3$)
f1 (ppm)

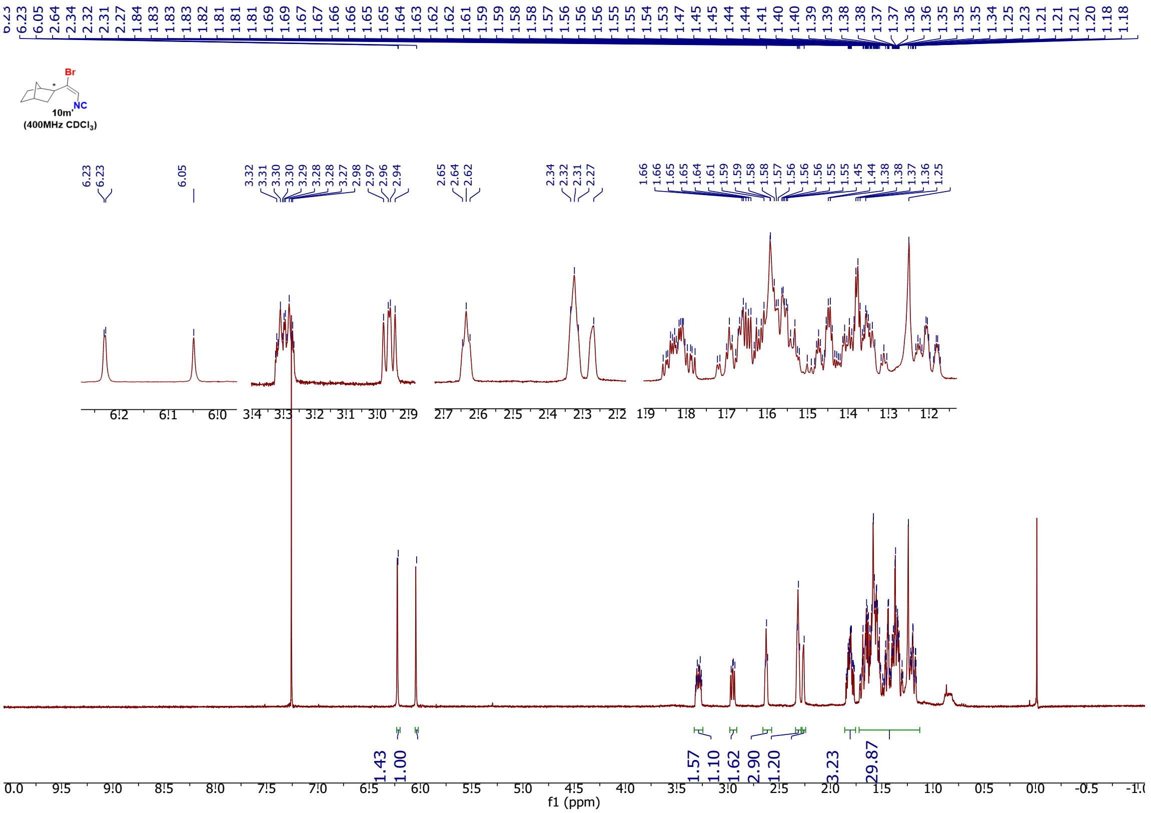

Br
NC
10m'
(400MHz CDCl3)
1.43
1.00
1.57
1.10
1.62
2.90
1.20
3.23
29.87
f1 (ppm)

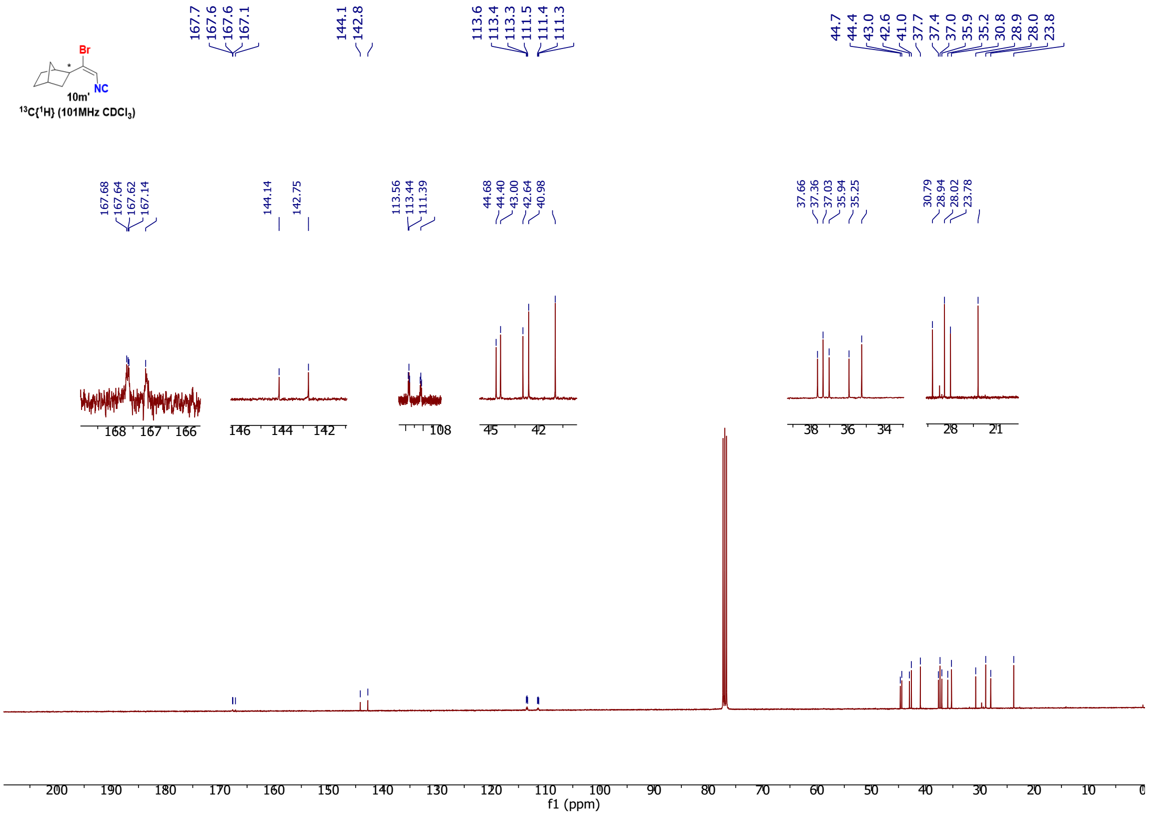

Br
NC
10m'
13C{1H} (101MHz CDCl3)
f1 (ppm)

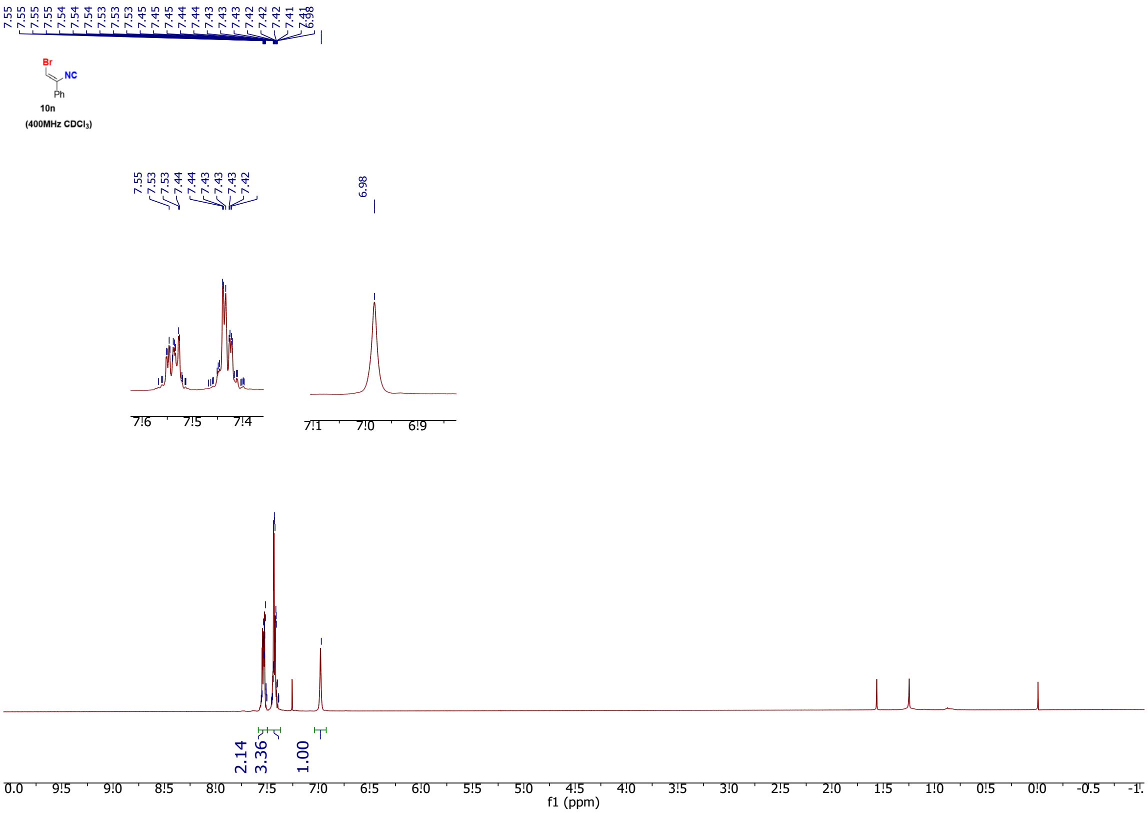
10n
(400MHz CDCl3)
2.14
3.36
1.00
f1 (ppm)

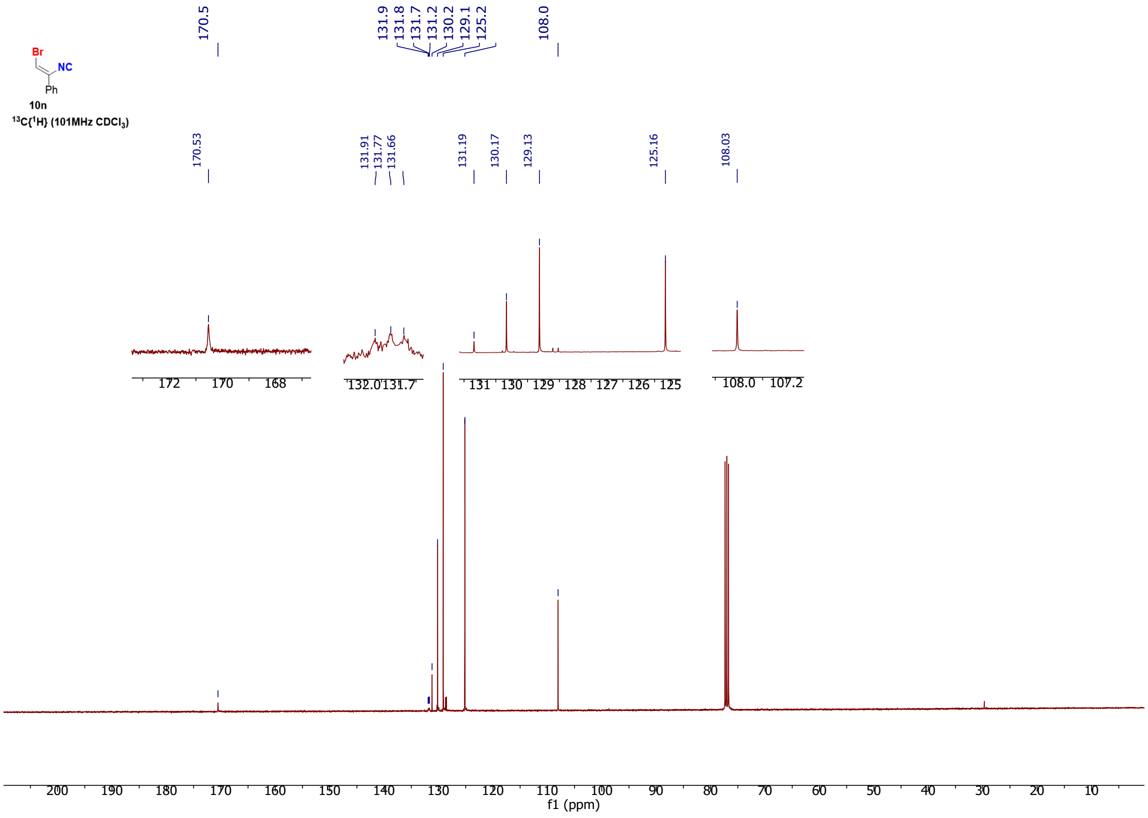
10n
13C{1H} (101MHz CDCl3)
f1 (ppm)

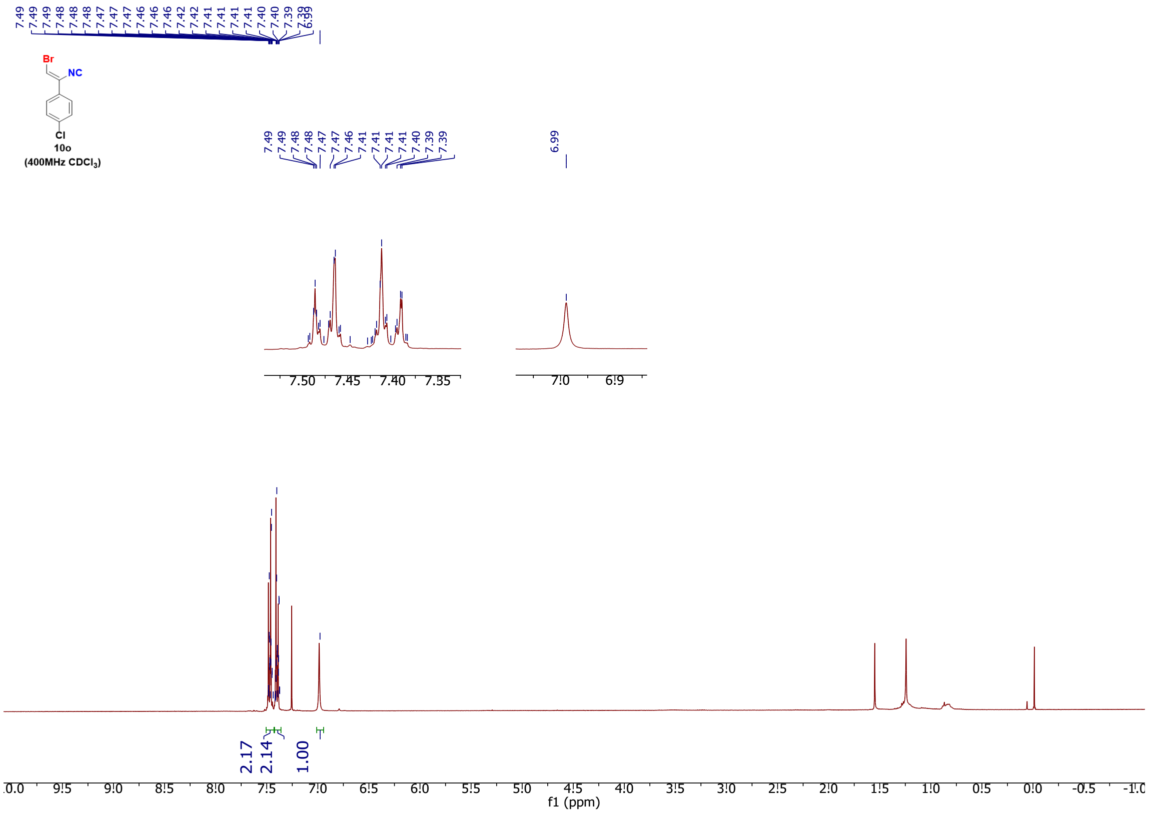
7.49
7.49
7.48
7.48
7.48
7.47
7.47
7.46
7.46
7.42
7.41
7.41
7.41
7.41
7.40
7.40
7.39
7.39
6.99
Br
NC
Cl
10o
(400MHz CDCl$_3$)
2.17
2.14
1.00
f1 (ppm)

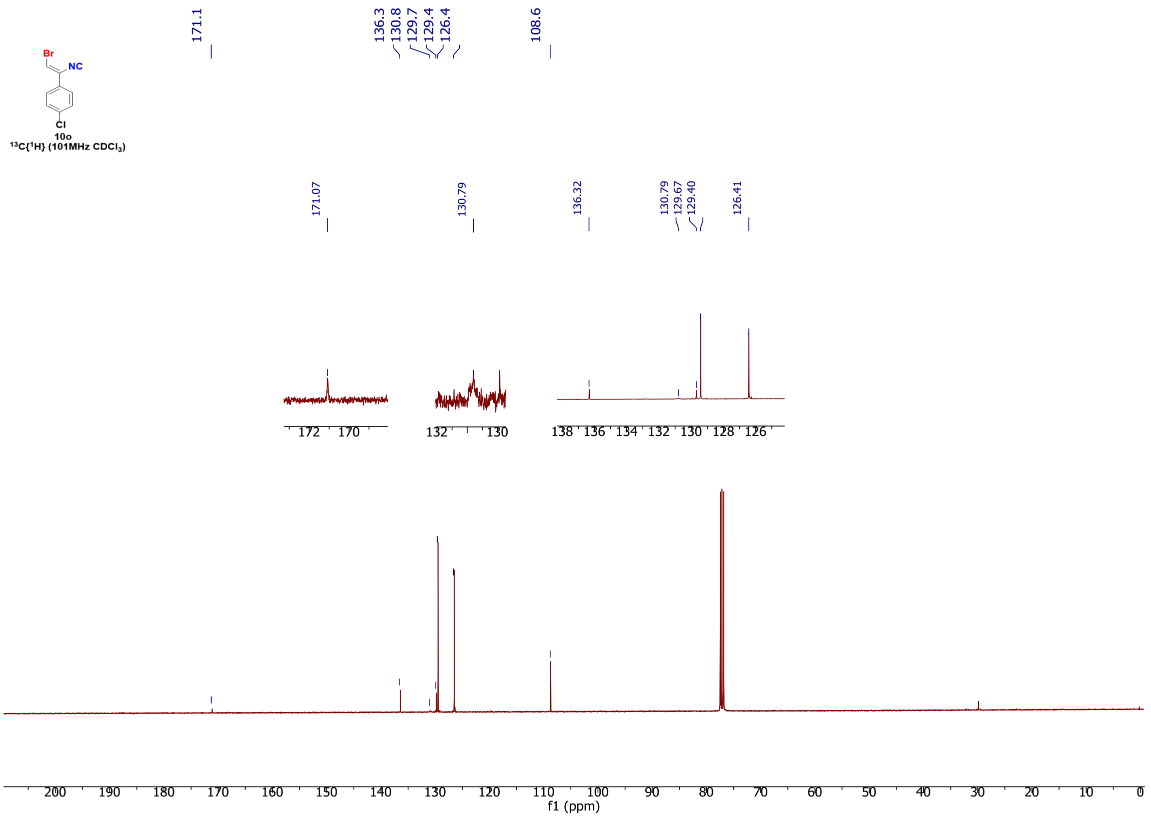
171.1
136.3
130.8
129.7
129.4
126.4
108.6
Br
NC
Cl
10o
$^{13}C\{^1H\}$ (101MHz CDCl$_3$)
171.07
130.79
136.32
130.79
129.67
129.40
126.41
f1 (ppm)

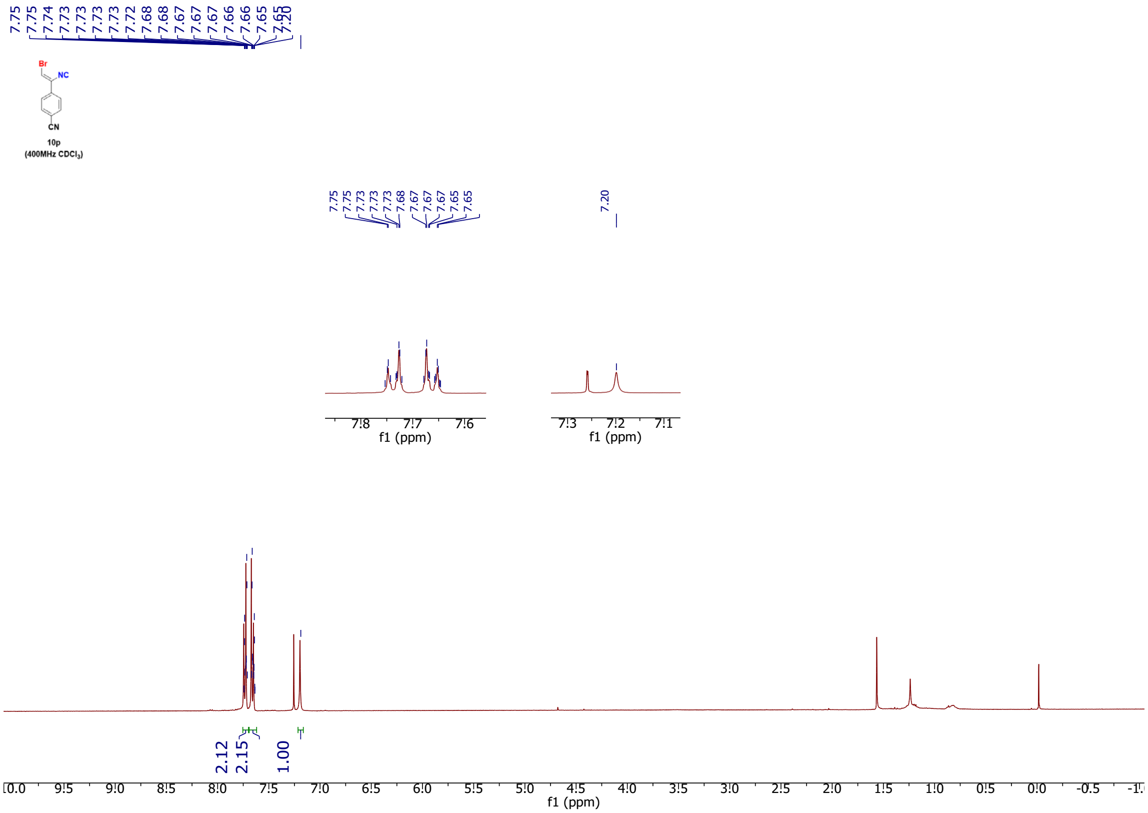
7.75
7.75
7.74
7.73
7.73
7.73
7.73
7.72
7.68
7.68
7.67
7.67
7.67
7.66
7.66
7.65
7.65
7.20
Br
NC
CN
10p
(400MHz CDCl3)
7.20
f1 (ppm)
2.12
2.15
1.00

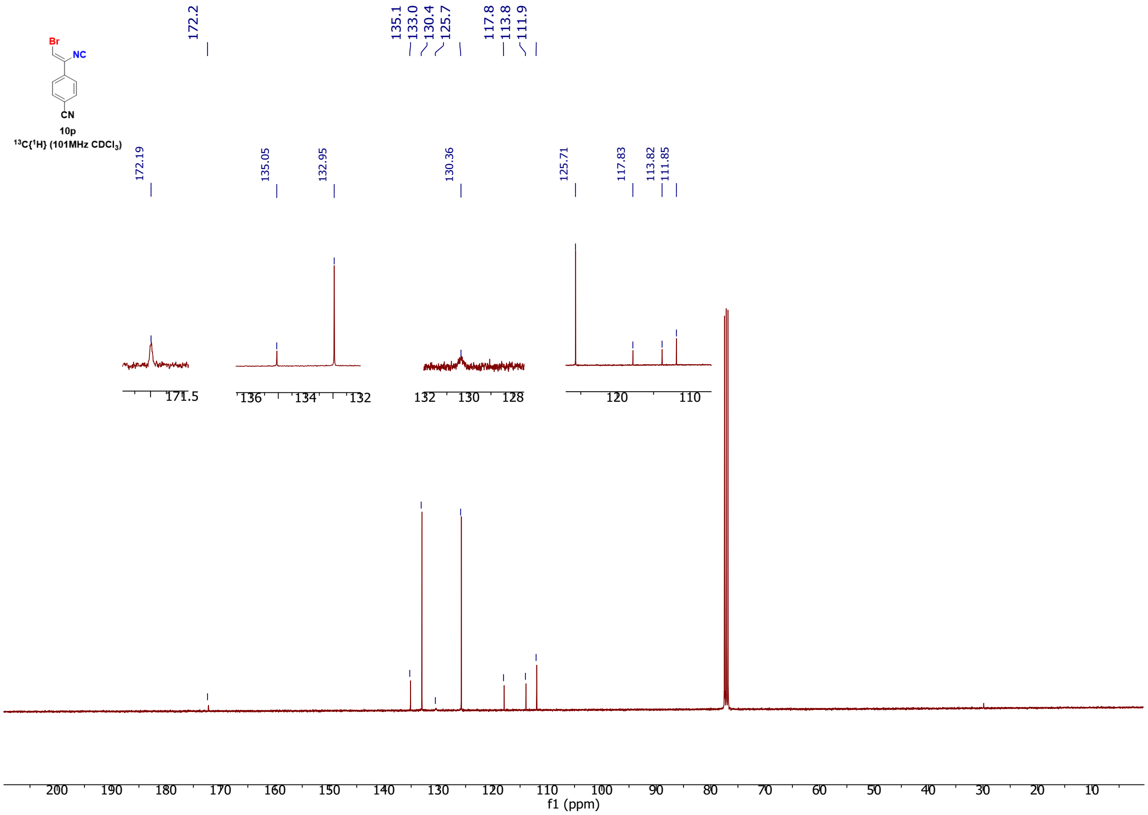
172.2
135.1
133.0
130.4
125.7
117.8
113.8
111.9
Br
NC
CN
10p
13C{1H} (101MHz CDCl3)
172.19
135.05
132.95
130.36
125.71
117.83
113.82
111.85
f1 (ppm)

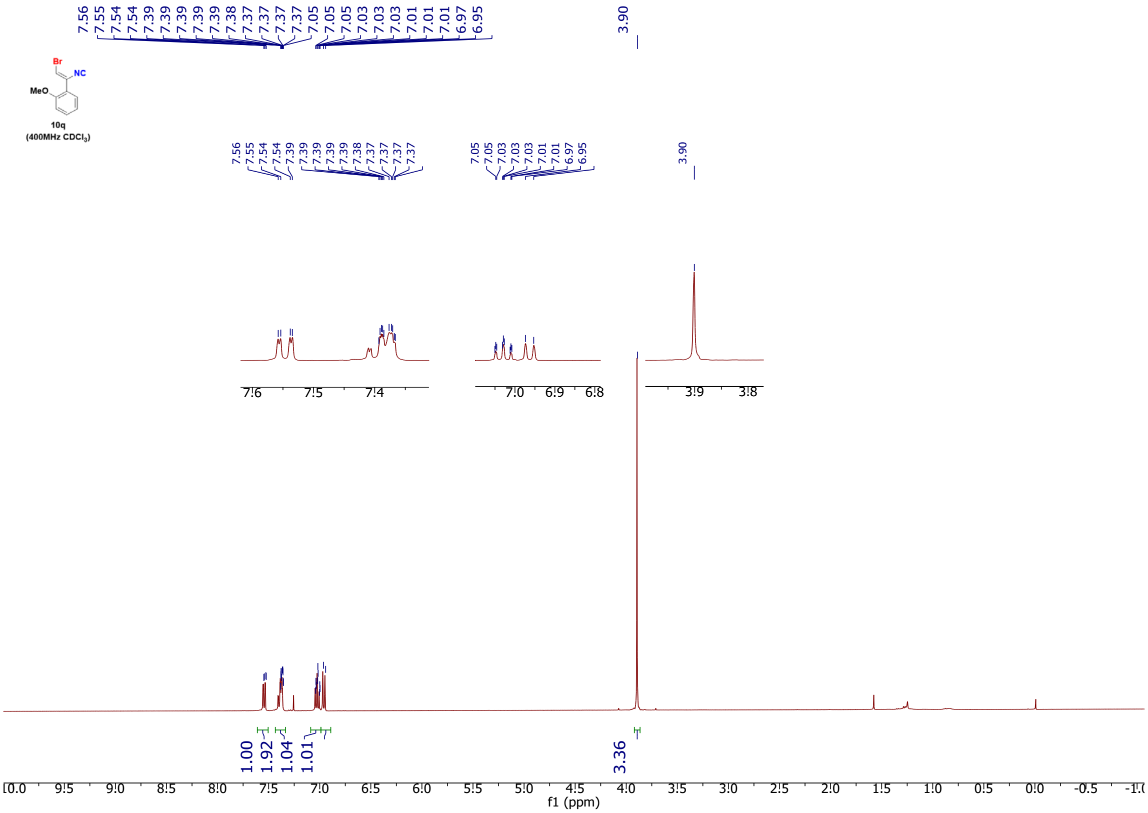
Br
NC
MeO
10q
(400MHz CDCl3)
7.56 7.55 7.54 7.54 7.39 7.39 7.39 7.39 7.39 7.38 7.37 7.37 7.37 7.37 7.05 7.05 7.05 7.03 7.03 7.03 7.01 7.01 6.97 6.95
3.90
1.00 1.92 1.04 1.01
3.36
f1 (ppm)

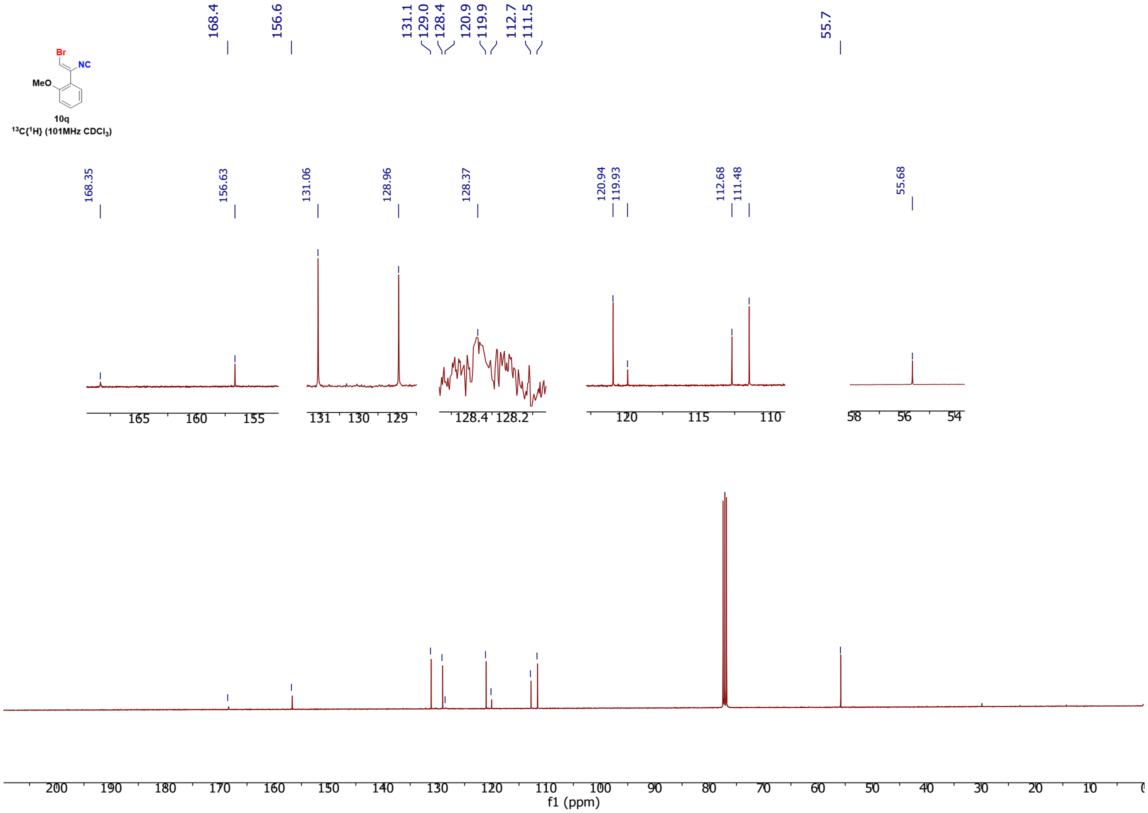
Br
NC
MeO
10q
13C{1H} (101MHz CDCl3)
168.4 156.6 131.1 129.0 128.4 120.9 119.9 112.7 111.5 55.7
168.35 156.63 131.06 128.96 128.37 120.94 119.93 112.68 111.48 55.68
f1 (ppm)

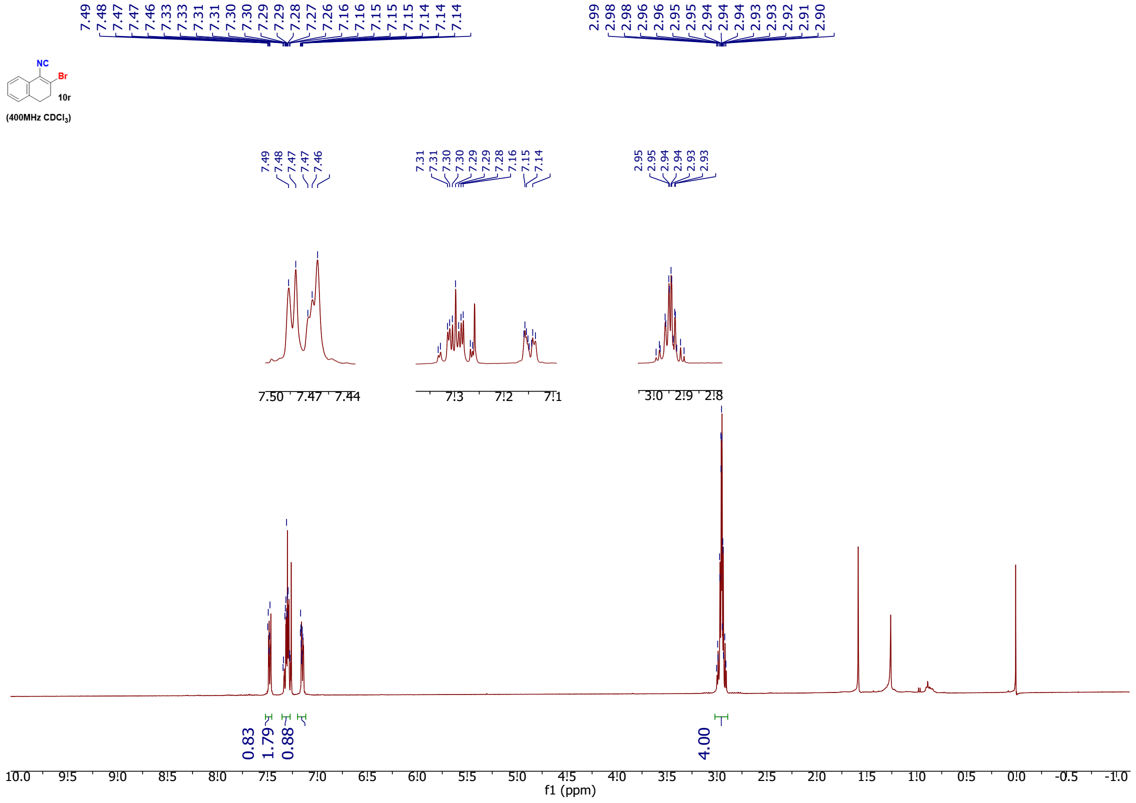

7.49
7.48
7.47
7.46
7.33
7.33
7.31
7.31
7.30
7.29
7.29
7.28
7.27
7.26
7.16
7.16
7.15
7.15
7.14
7.14
7.14
2.99
2.98
2.98
2.96
2.95
2.95
2.94
2.94
2.94
2.93
2.93
2.92
2.91
2.90
NC
Br
10r
(400MHz CDCl3)
0.83
1.79
0.88
4.00
f1 (ppm)

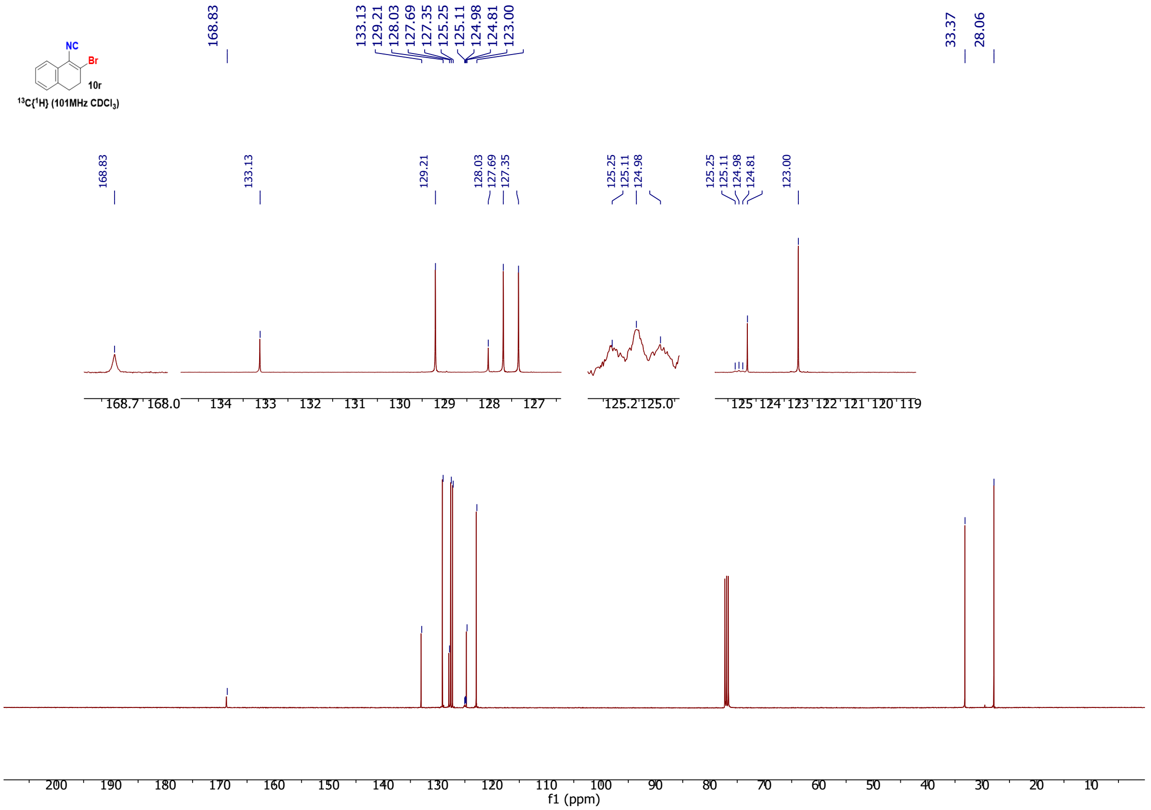

168.83
133.13
129.21
128.03
127.69
127.35
125.25
125.11
124.98
124.81
123.00
33.37
28.06
NC
Br
10r
13C{1H} (101MHz CDCl3)
f1 (ppm)

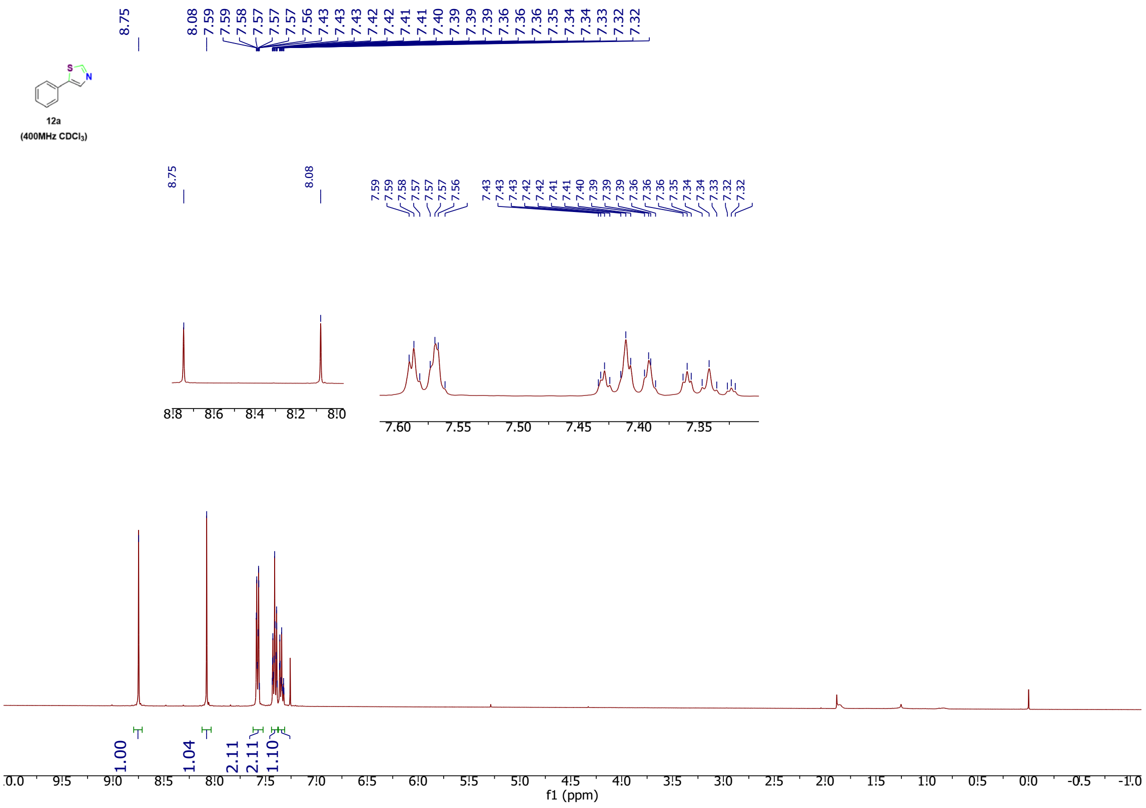
8.75
8.08 7.59 7.59 7.58 7.57 7.57 7.57 7.57 7.56 7.43 7.43 7.43 7.42 7.42 7.41 7.41 7.40 7.39 7.39 7.39 7.36 7.36 7.36 7.35 7.34 7.34 7.33 7.32 7.32
S
N
12a
(400MHz CDCl3)
1.00
1.04
2.11
2.11
1.10
f1 (ppm)

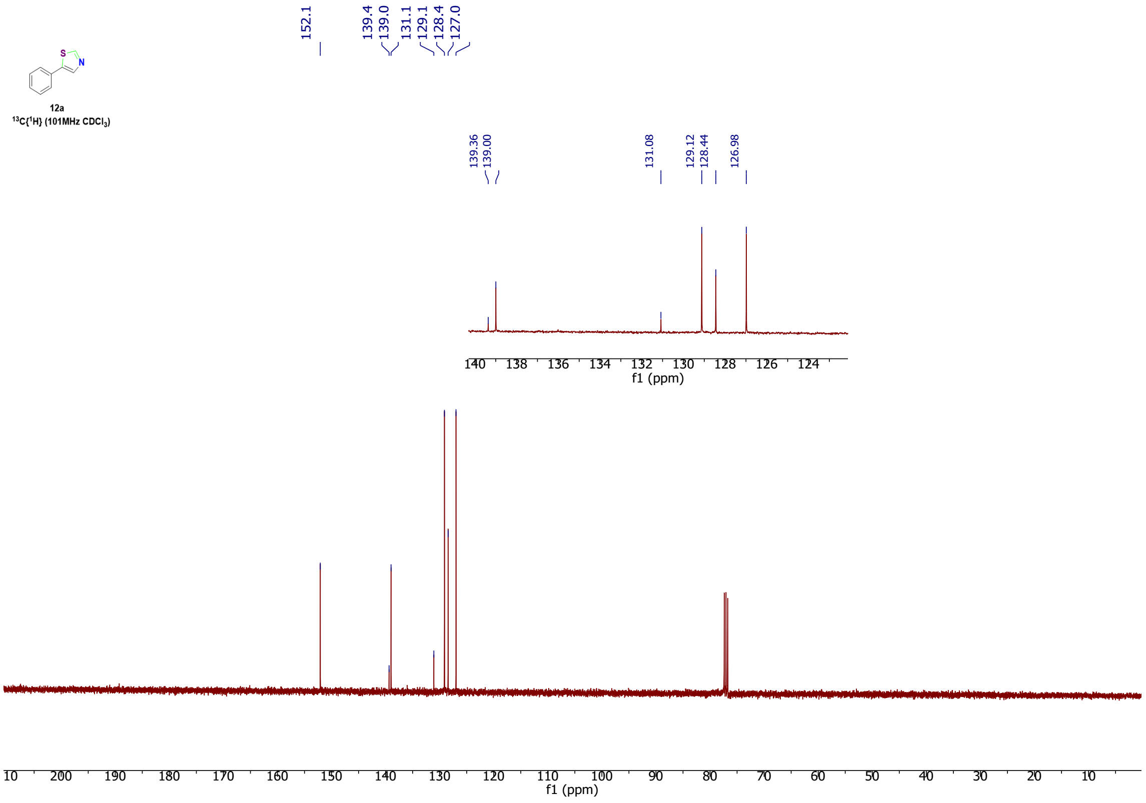
152.1
139.4
139.0
131.1
129.1
128.4
127.0
S
N
12a
13C{1H} (101MHz CDCl3)
139.36
139.00
131.08
129.12
128.44
126.98
f1 (ppm)

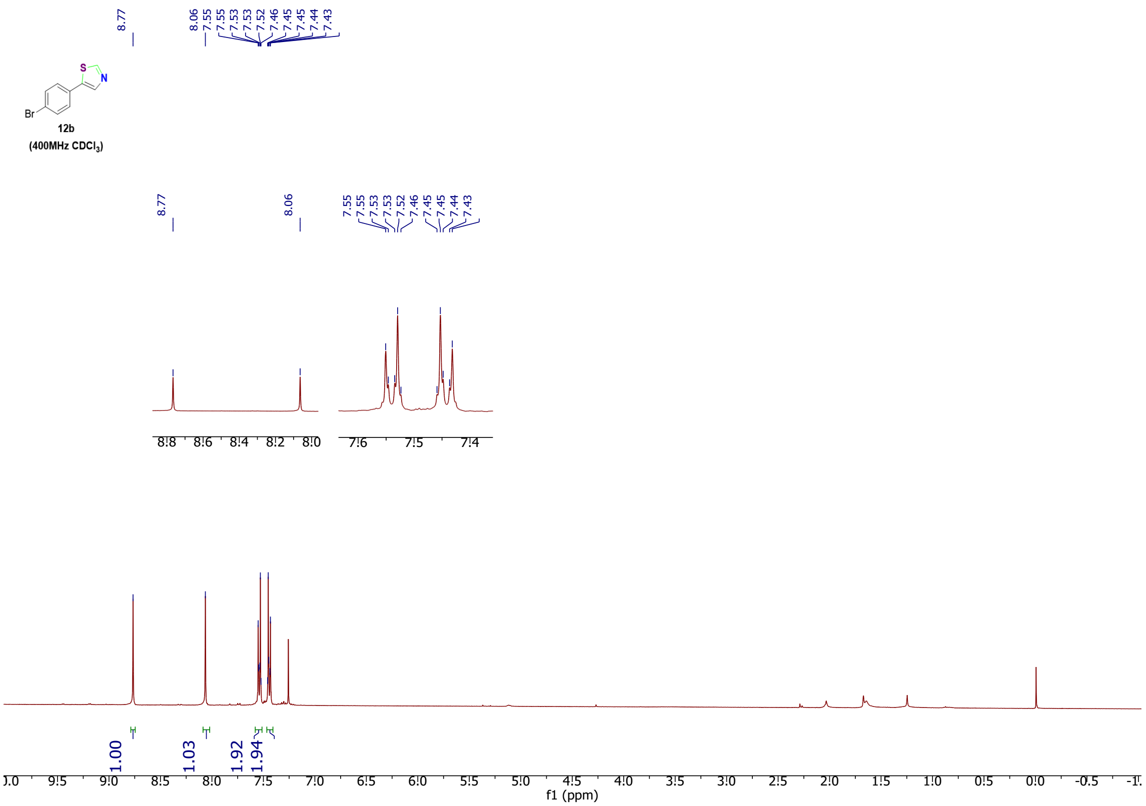
12b
(400MHz CDCl$_3$)
8.77
8.06
7.55
7.55
7.53
7.53
7.52
7.46
7.45
7.45
7.44
7.43
1.00
1.03
1.92
1.94
f1 (ppm)

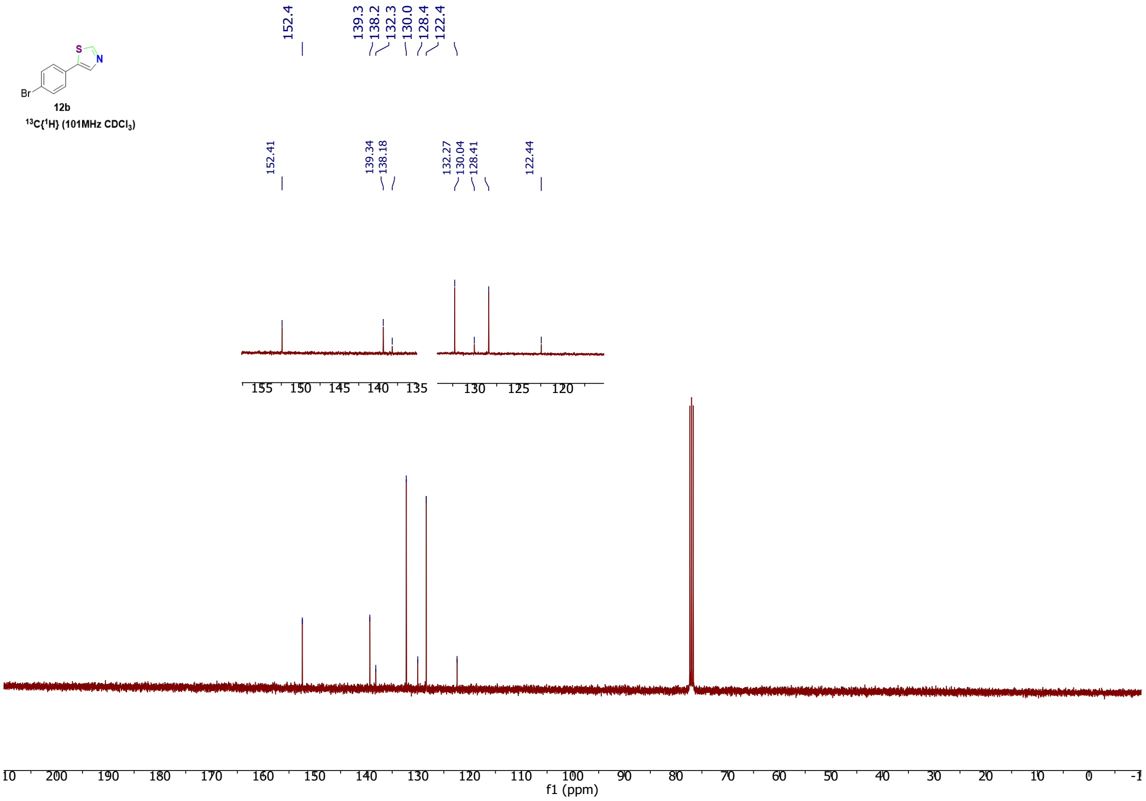
12b
$^{13}C\{^1H\}$ (101MHz CDCl$_3$)
152.4
139.3
138.2
132.3
130.0
128.4
122.4
152.41
139.34
138.18
132.27
130.04
128.41
122.44
f1 (ppm)

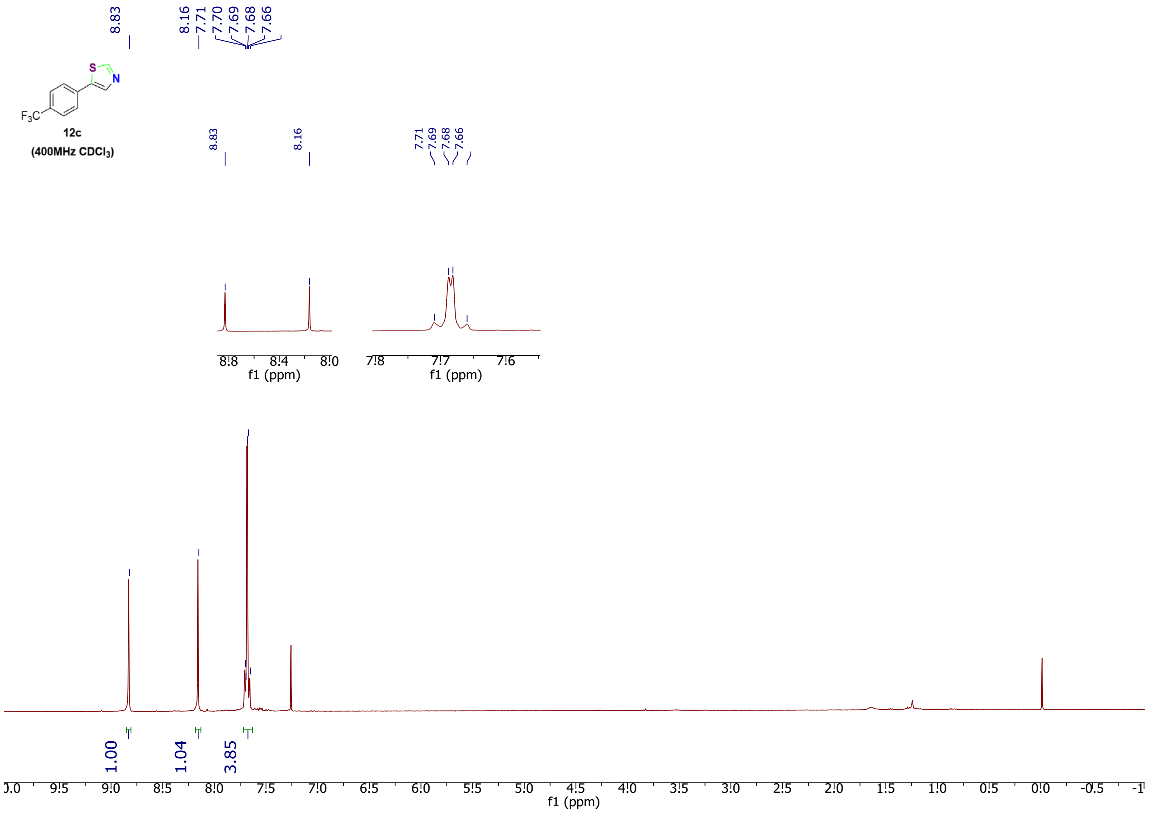
8.83
8.16
7.71
7.70
7.69
7.68
7.66
12c
(400MHz CDCl3)
8.83
8.16
7.71
7.69
7.68
7.66
8.8
8.4
8.0
f1 (ppm)
7.8
7.7
7.6
f1 (ppm)
1.00
1.04
3.85
9.5
9.0
8.5
8.0
7.5
7.0
6.5
6.0
5.5
5.0
4.5
4.0
3.5
3.0
2.5
2.0
1.5
1.0
0.5
0.0
-0.5
f1 (ppm)

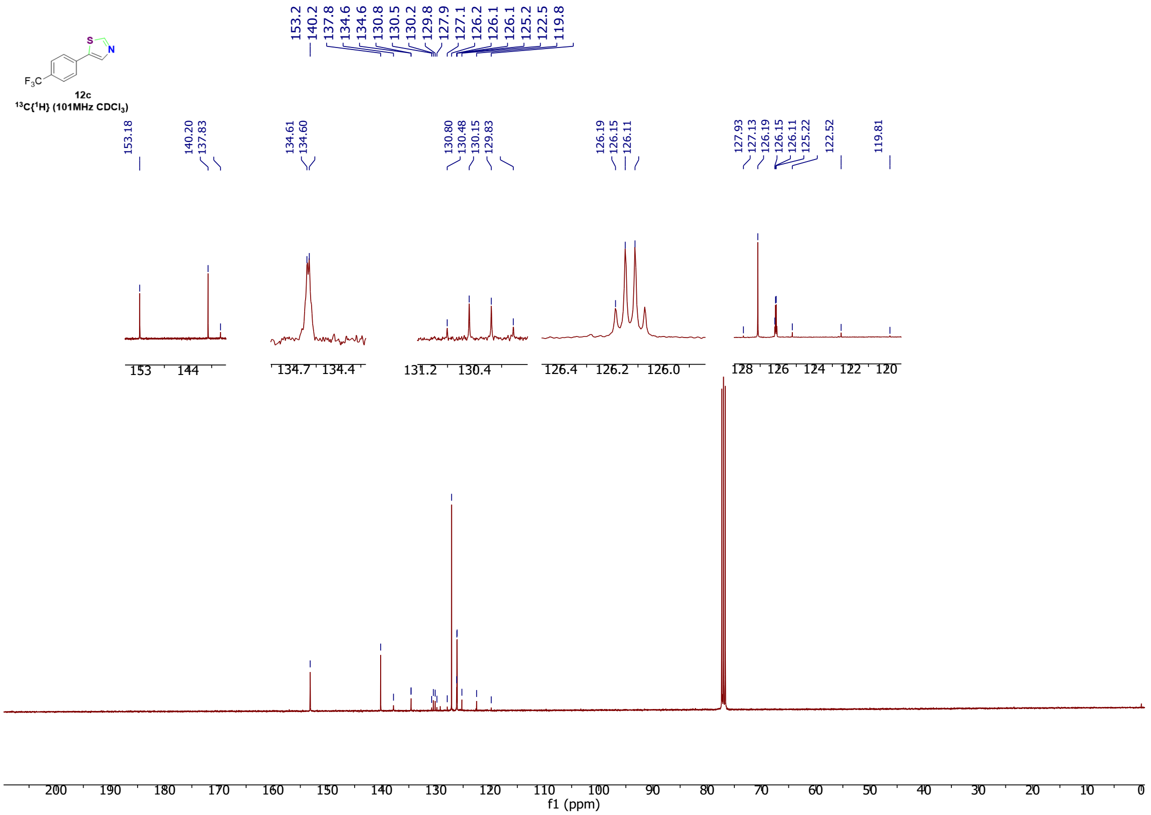
153.2
140.2
137.8
134.6
134.6
130.8
130.5
130.2
129.8
127.9
127.1
126.2
126.1
126.1
125.2
122.5
119.8
12c
13C{1H} (101MHz CDCl3)
153.18
140.20
137.83
134.61
134.60
130.80
130.48
130.15
129.83
126.19
126.15
126.11
127.93
127.13
126.19
126.15
126.11
125.22
122.52
119.81
153
144
134.7
134.4
131.2
130.4
126.4
126.2
126.0
128
126
124
122
120
200
190
180
170
160
150
140
130
120
110
100
90
80
70
60
50
40
30
20
10
0
f1 (ppm)

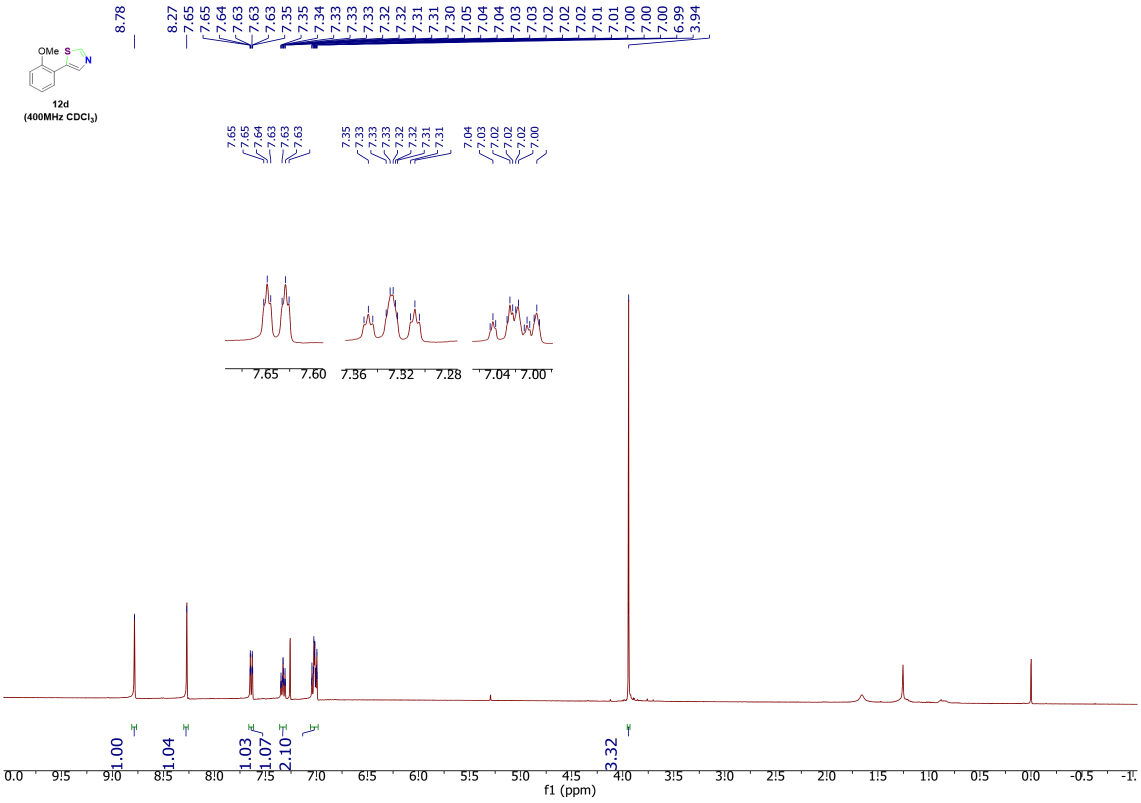

OMe
S
N
12d
(400MHz CDCl3)
8.78
8.27
7.65
7.64
7.63
7.35
7.34
7.33
7.32
7.31
7.30
7.05
7.04
7.03
7.02
7.01
7.00
6.99
3.94
1.00
1.04
1.03
1.07
2.10
3.32
f1 (ppm)

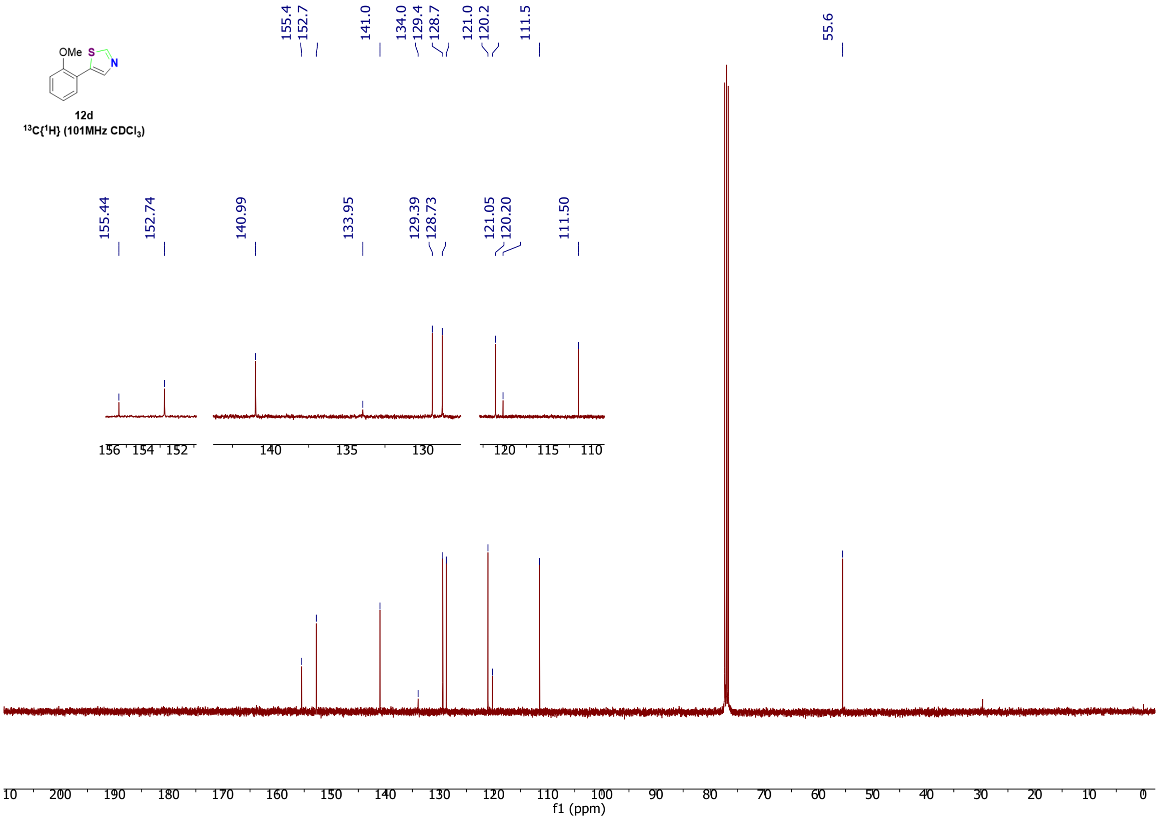

OMe
S
N
12d
13C{1H} (101MHz CDCl3)
155.4
152.7
141.0
134.0
129.4
128.7
121.0
120.2
111.5
55.6
155.44
152.74
140.99
133.95
129.39
128.73
121.05
120.20
111.50
f1 (ppm)

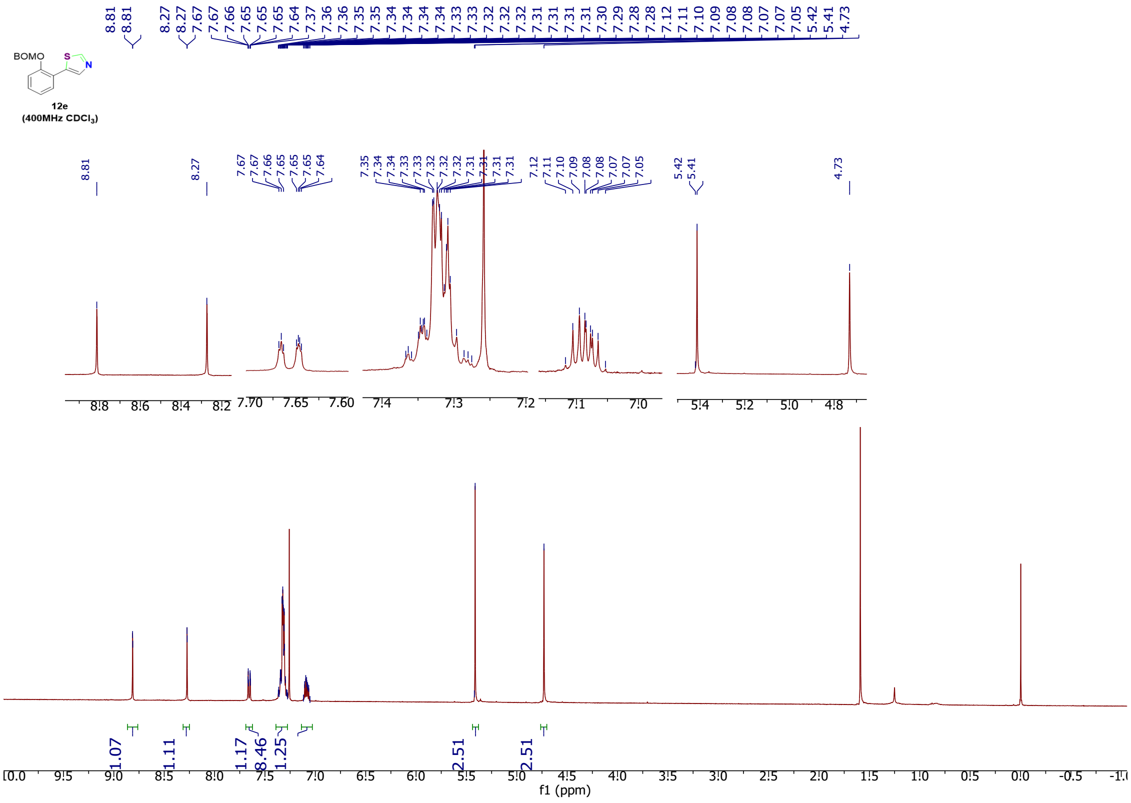

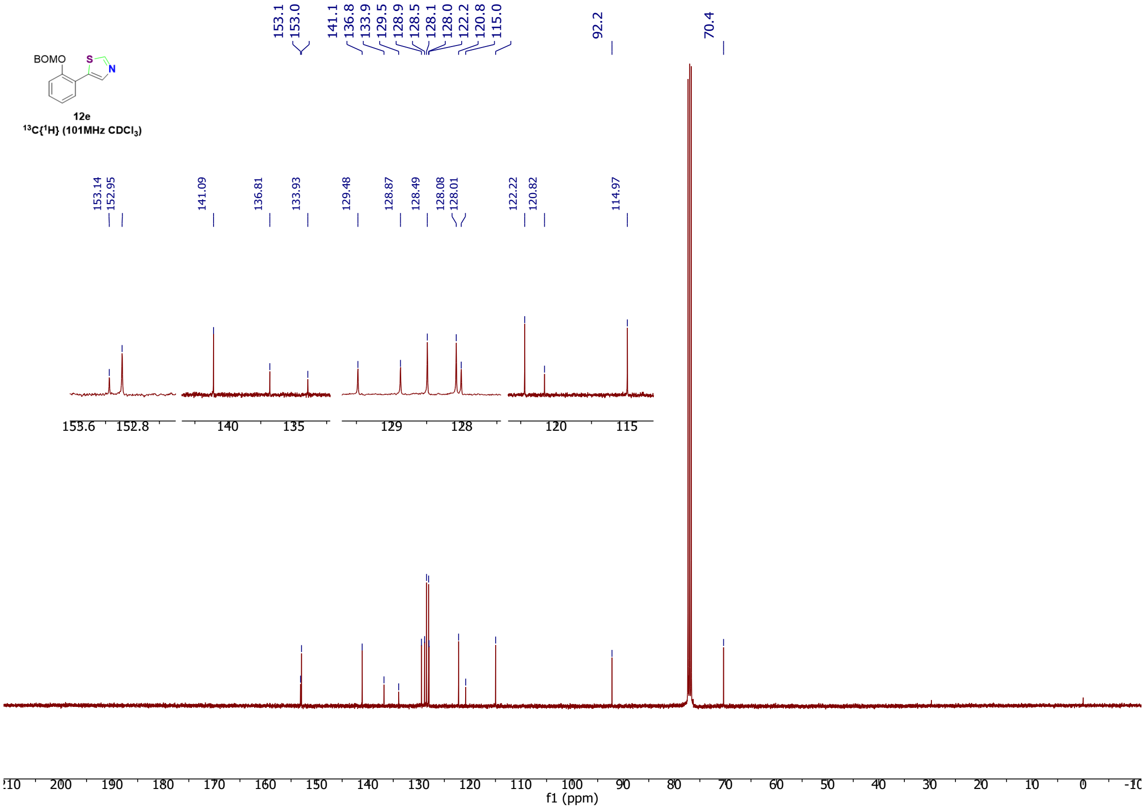

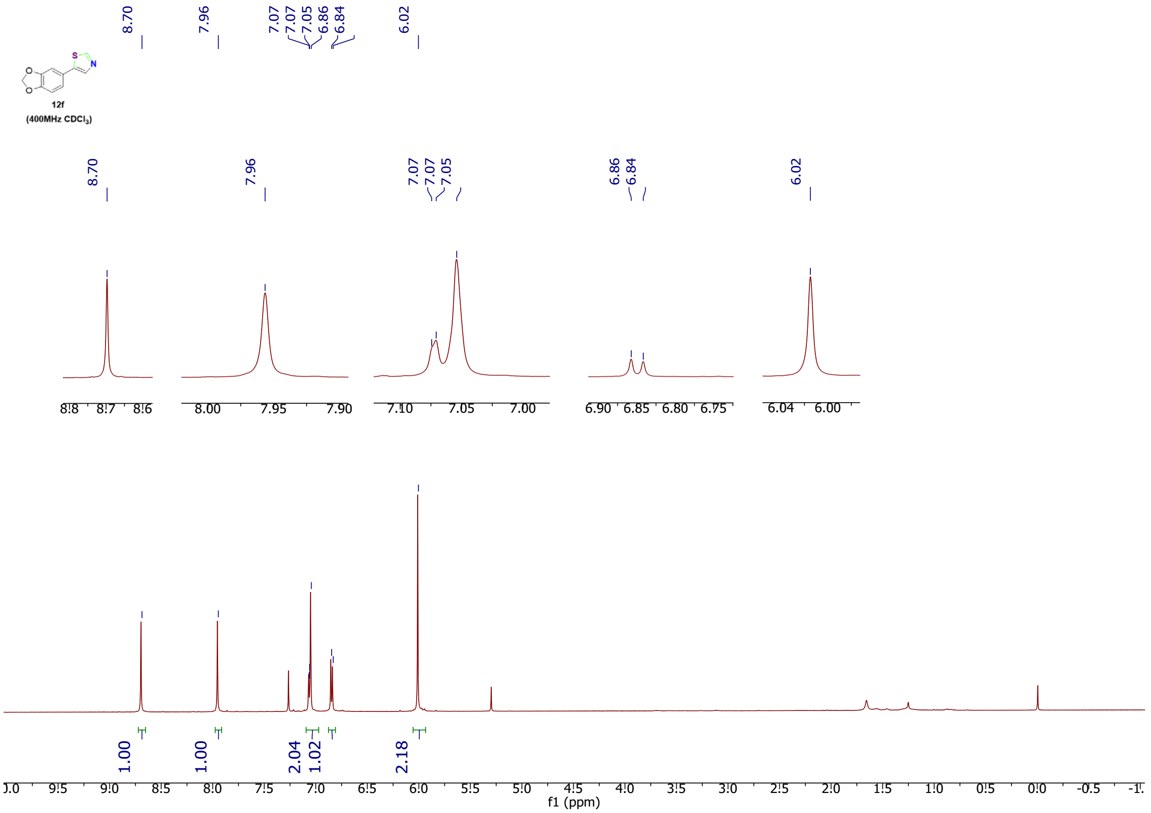
8.70
7.96
7.07
7.07
7.05
6.86
6.84
6.02
12f
(400MHz CDCl3)
1.00
1.00
2.04
1.02
2.18
f1 (ppm)

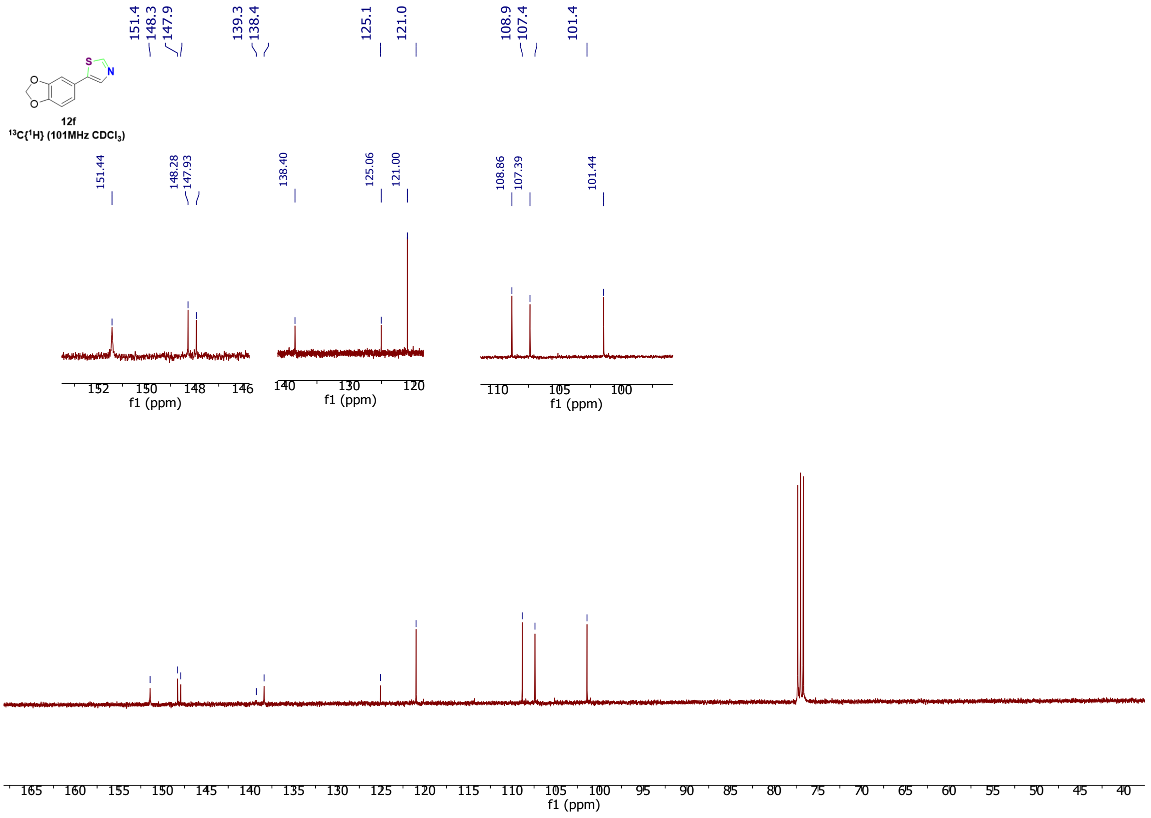
151.4
148.3
147.9
139.3
138.4
125.1
121.0
108.9
107.4
101.4
12f
13C{1H} (101MHz CDCl3)
151.44
148.28
147.93
138.40
125.06
121.00
108.86
107.39
101.44
f1 (ppm)

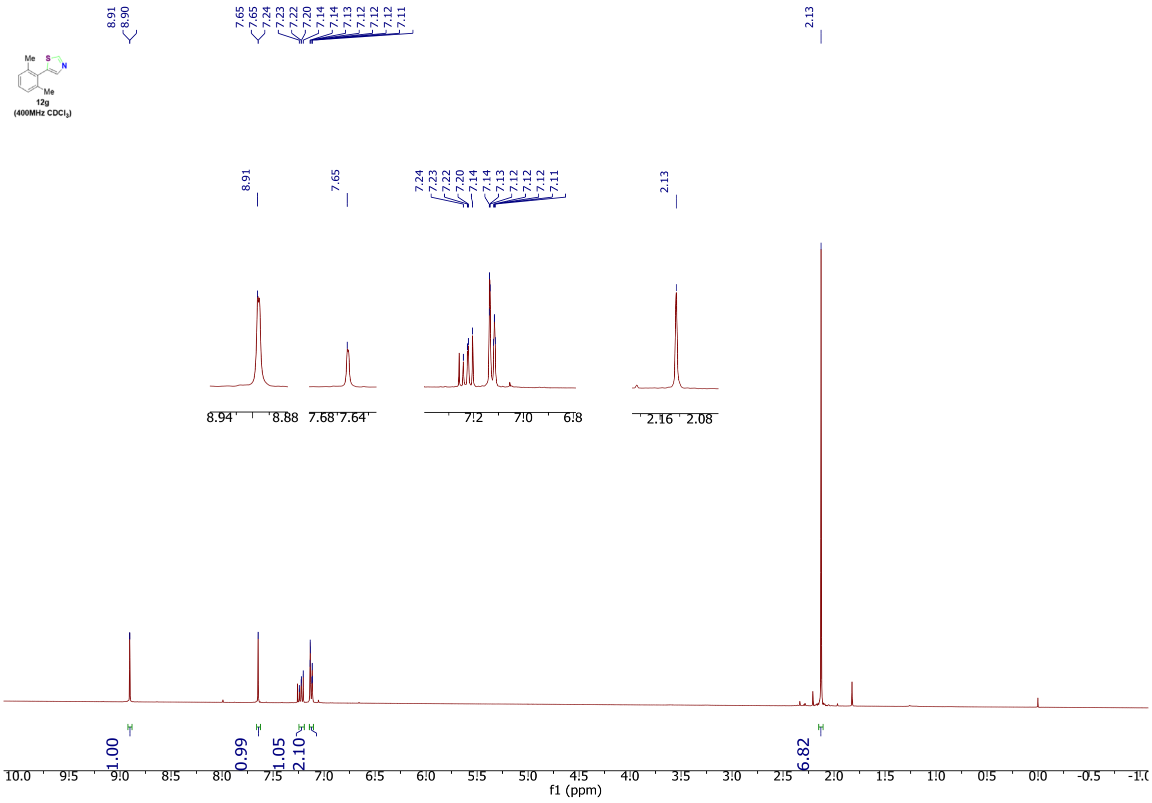
12g
(400MHz CDCl3)
8.91
8.90
7.65
7.65
7.24
7.23
7.22
7.20
7.14
7.14
7.13
7.12
7.12
7.12
7.11
2.13
1.00
0.99
1.05
2.10
6.82
f1 (ppm)

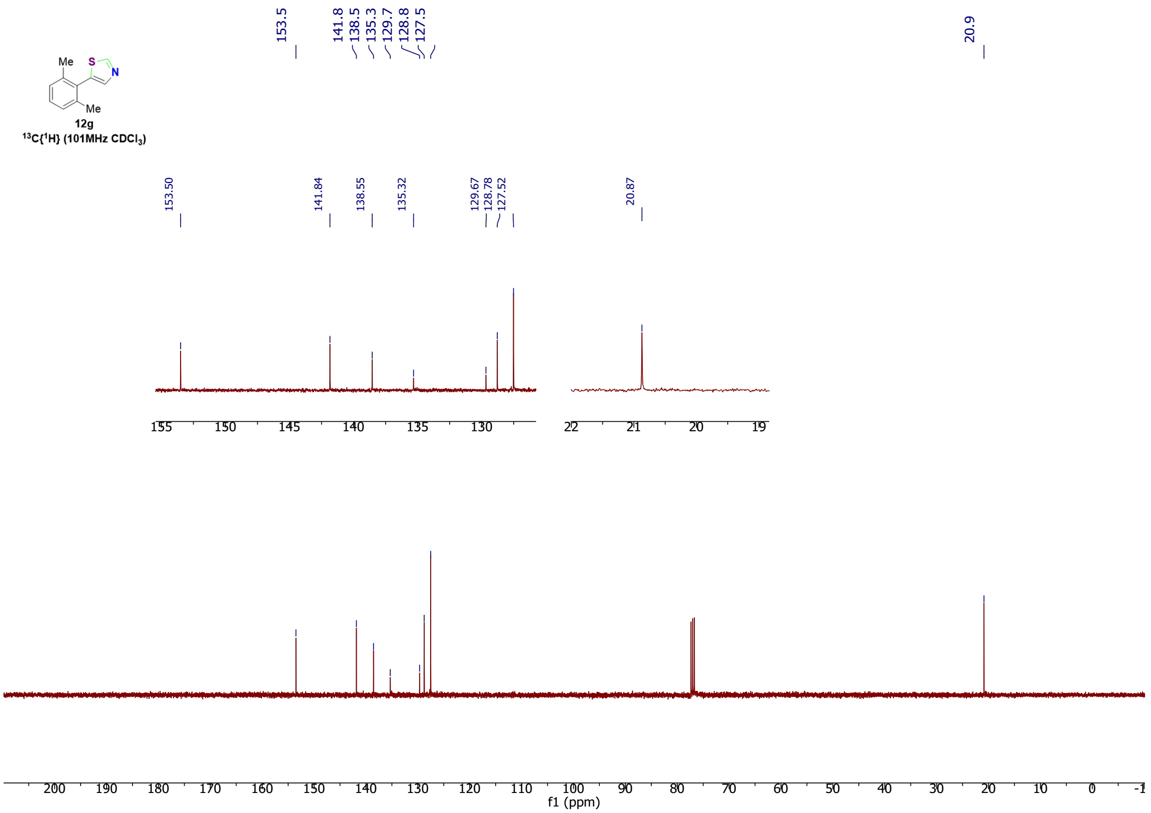
12g
13C{1H} (101MHz CDCl3)
153.5
141.8
138.5
135.3
129.7
128.8
127.5
20.9
153.50
141.84
138.55
135.32
129.67
128.78
127.52
20.87
f1 (ppm)

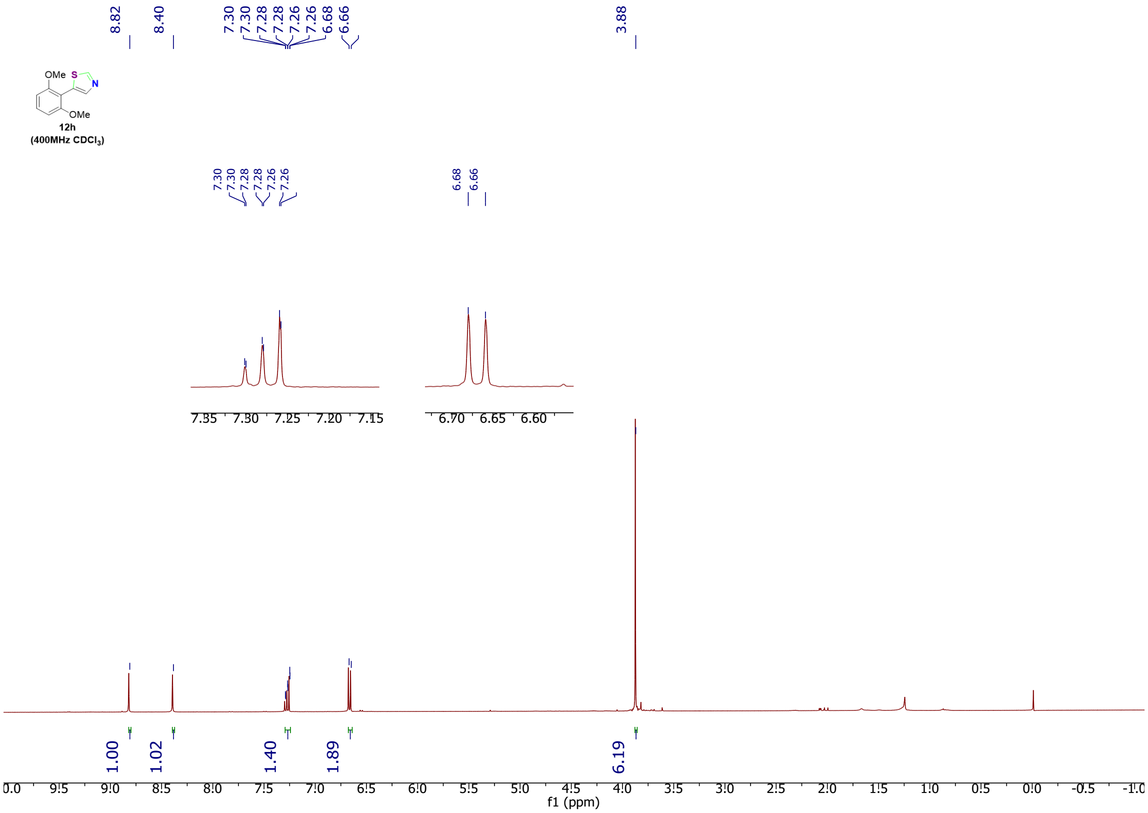
OMe
S
N
OMe
12h
(400MHz CDCl3)
8.82
8.40
7.30
7.30
7.28
7.28
7.26
7.26
6.68
6.66
3.88
1.00
1.02
1.40
1.89
6.19
f1 (ppm)

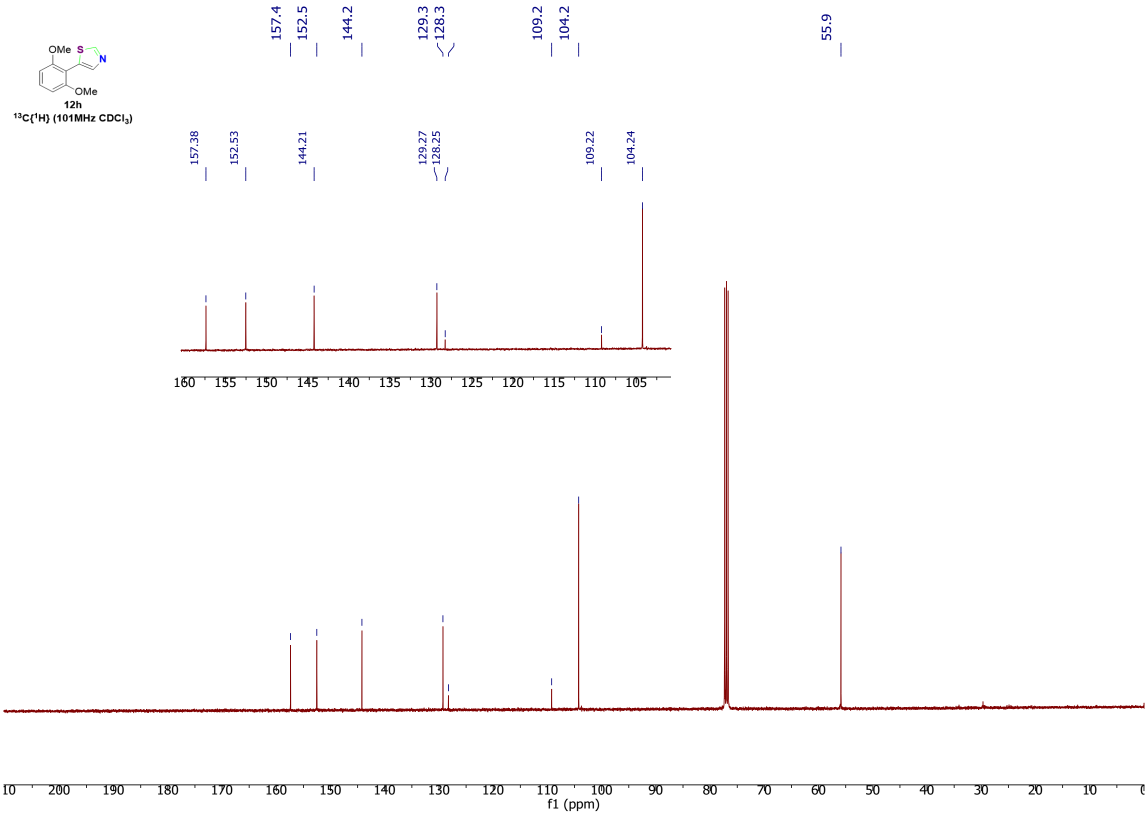
OMe
S
N
OMe
12h
13C{1H} (101MHz CDCl3)
157.4
152.5
144.2
129.3
128.3
109.2
104.2
55.9
157.38
152.53
144.21
129.27
128.25
109.22
104.24
f1 (ppm)

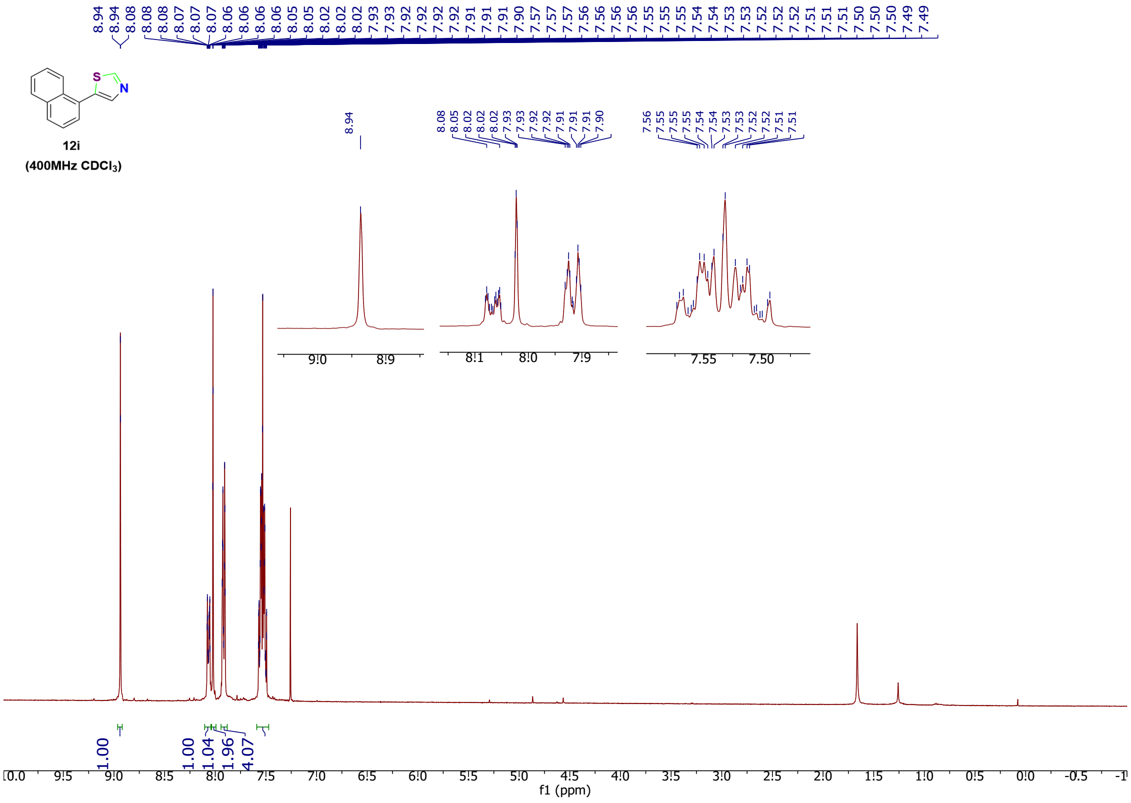
S
N
12i
(400MHz CDCl3)
1.00
1.00
1.04
1.96
4.07
f1 (ppm)

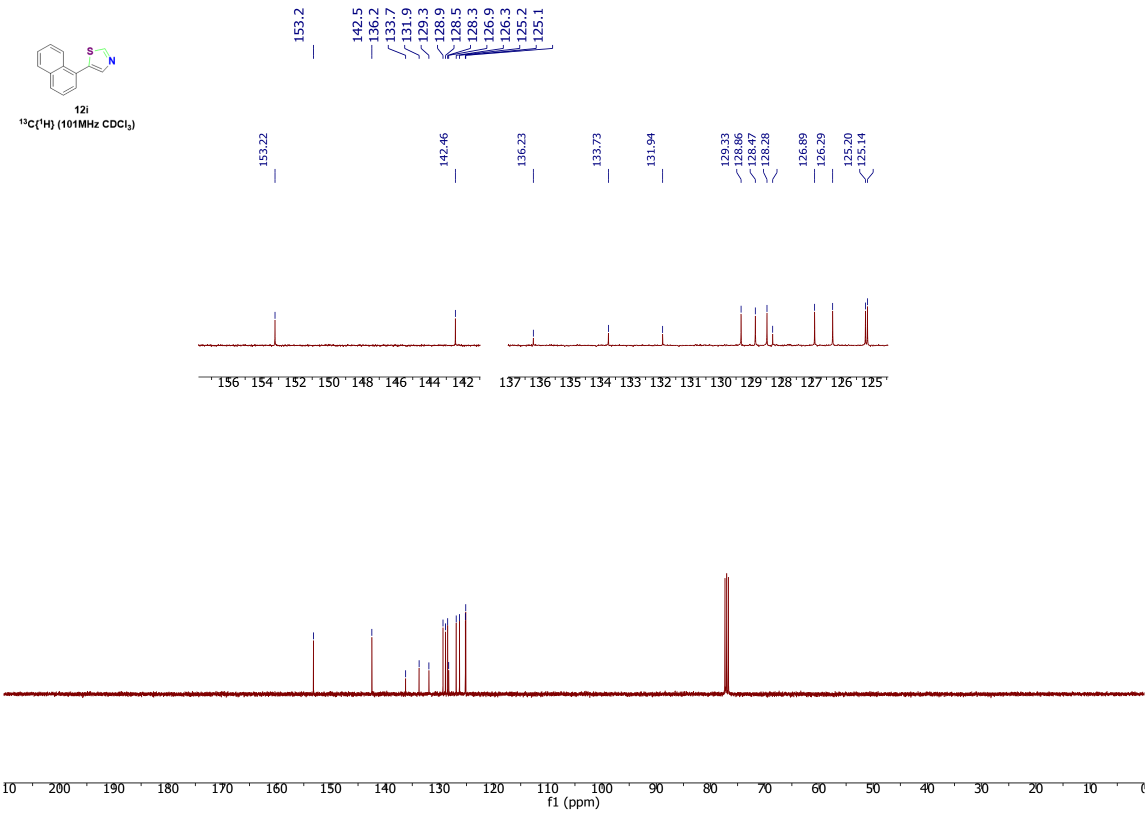
S
N
12i
13C{1H} (101MHz CDCl3)
153.2
142.5
136.2
133.7
131.9
129.3
128.9
128.5
128.3
126.9
126.3
125.2
125.1
153.22
142.46
136.23
133.73
131.94
129.33
128.86
128.47
128.28
126.89
126.29
125.20
125.14
f1 (ppm)

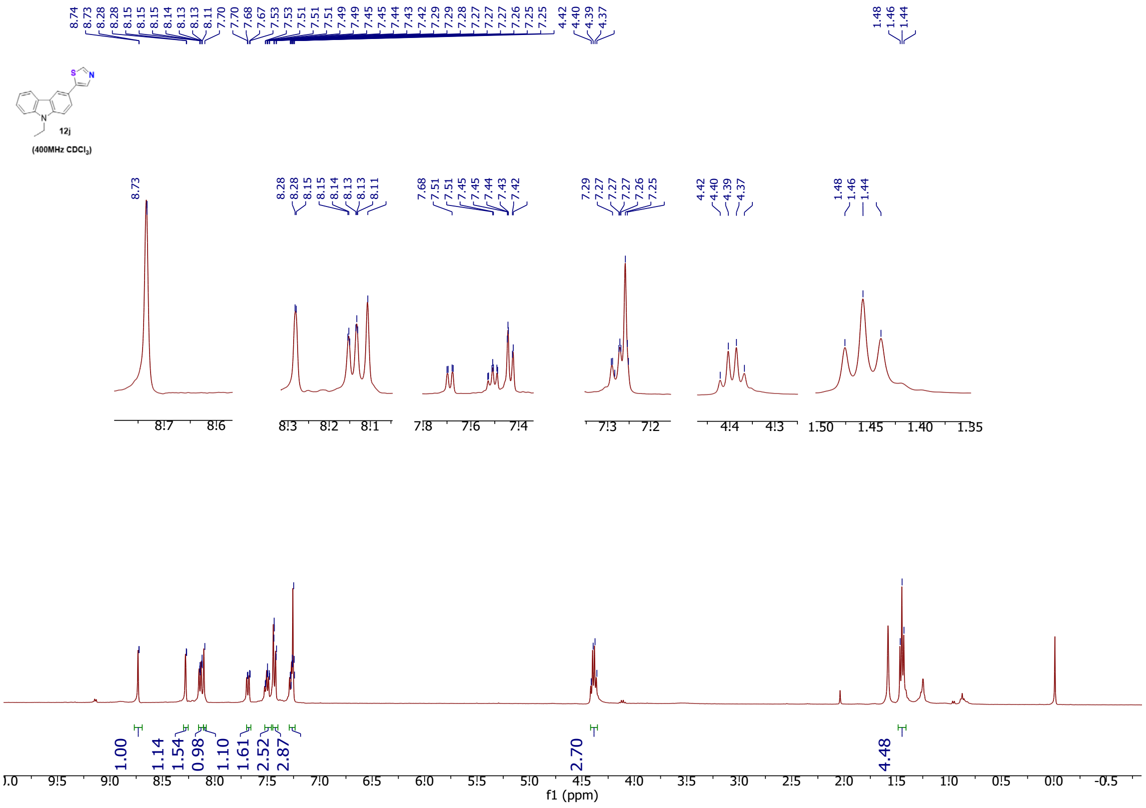

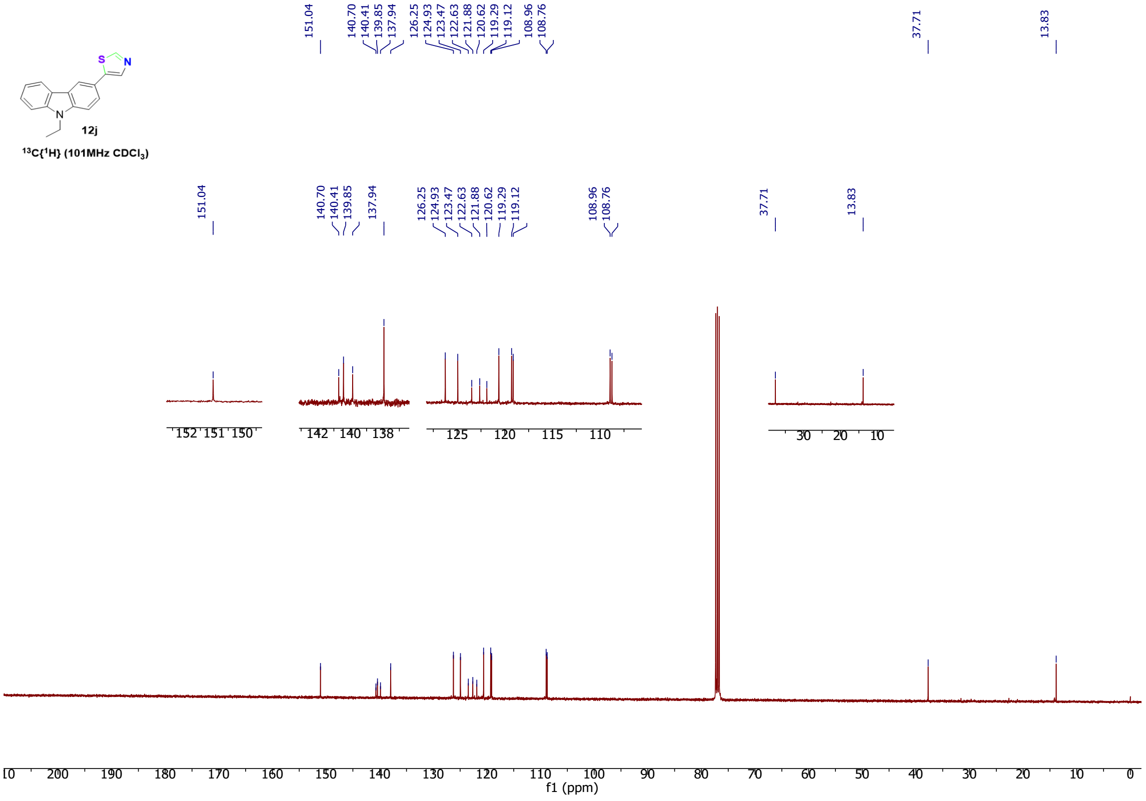

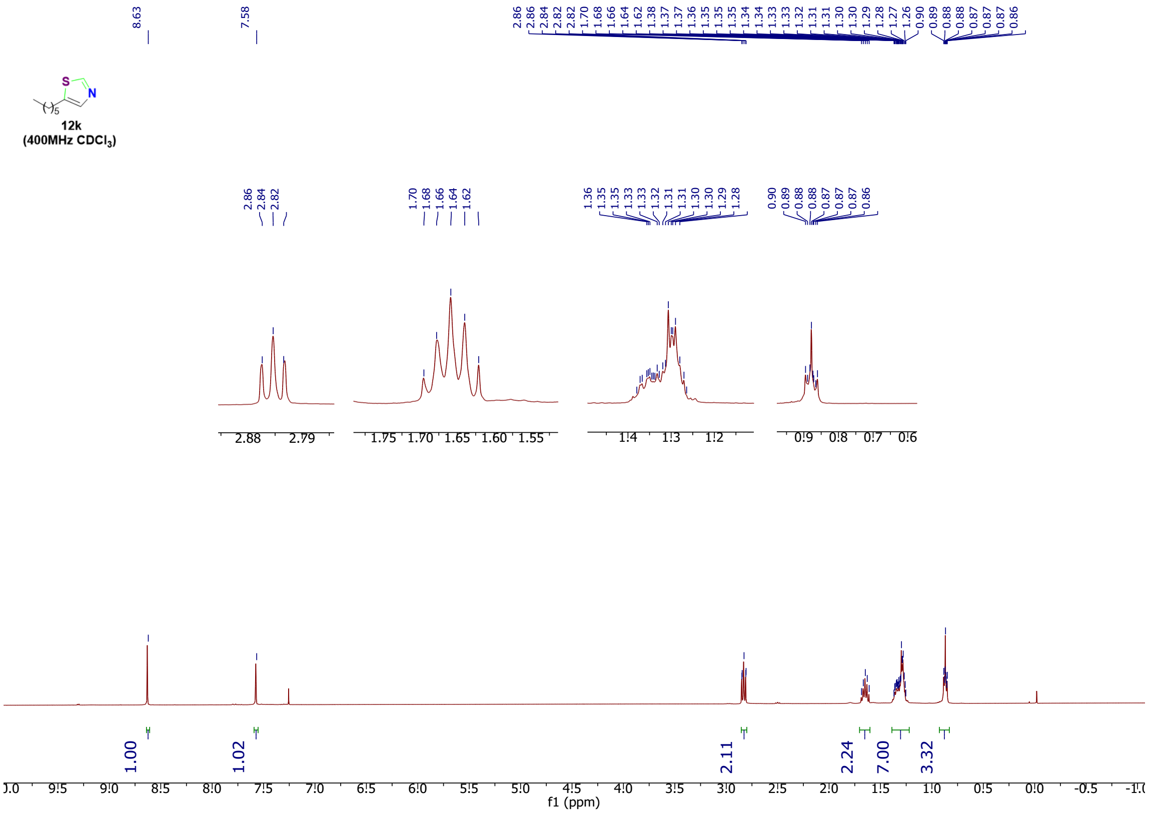
12k
(400MHz CDCl$_3$)

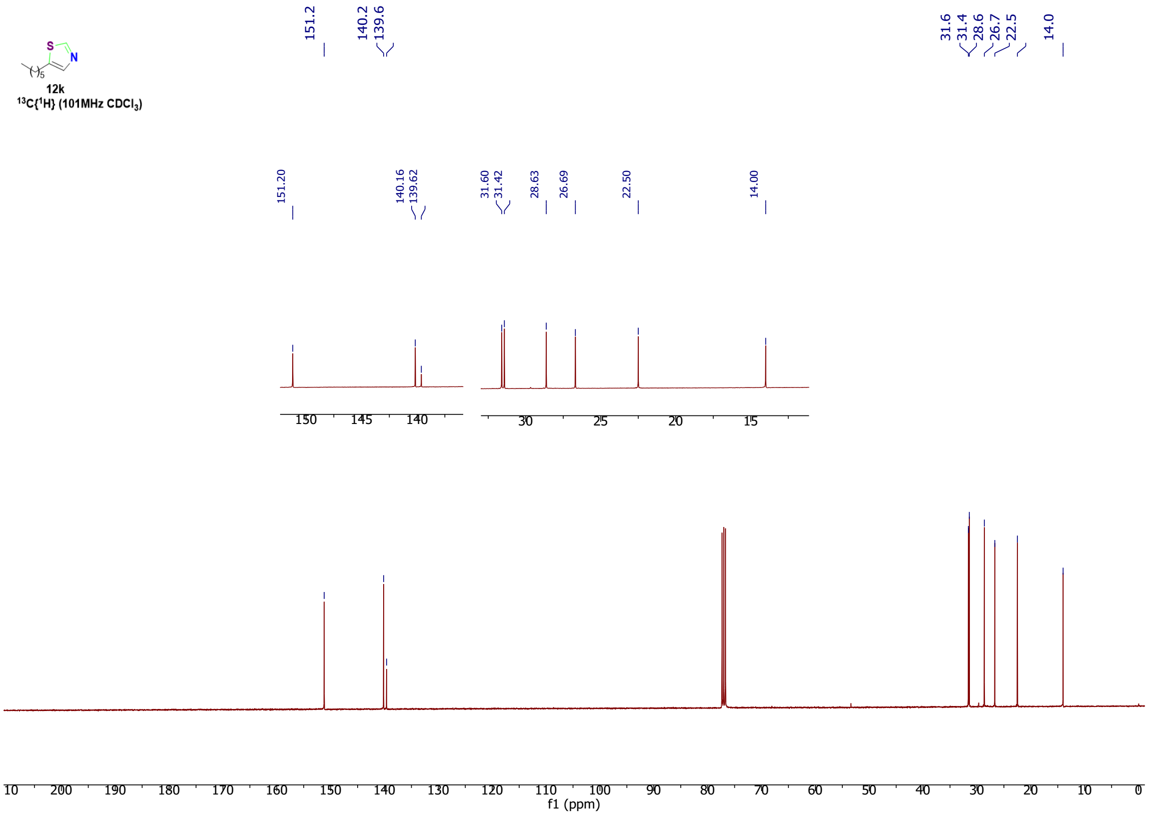
12k
$^{13}C\{^1H\}$ (101MHz CDCl$_3$)

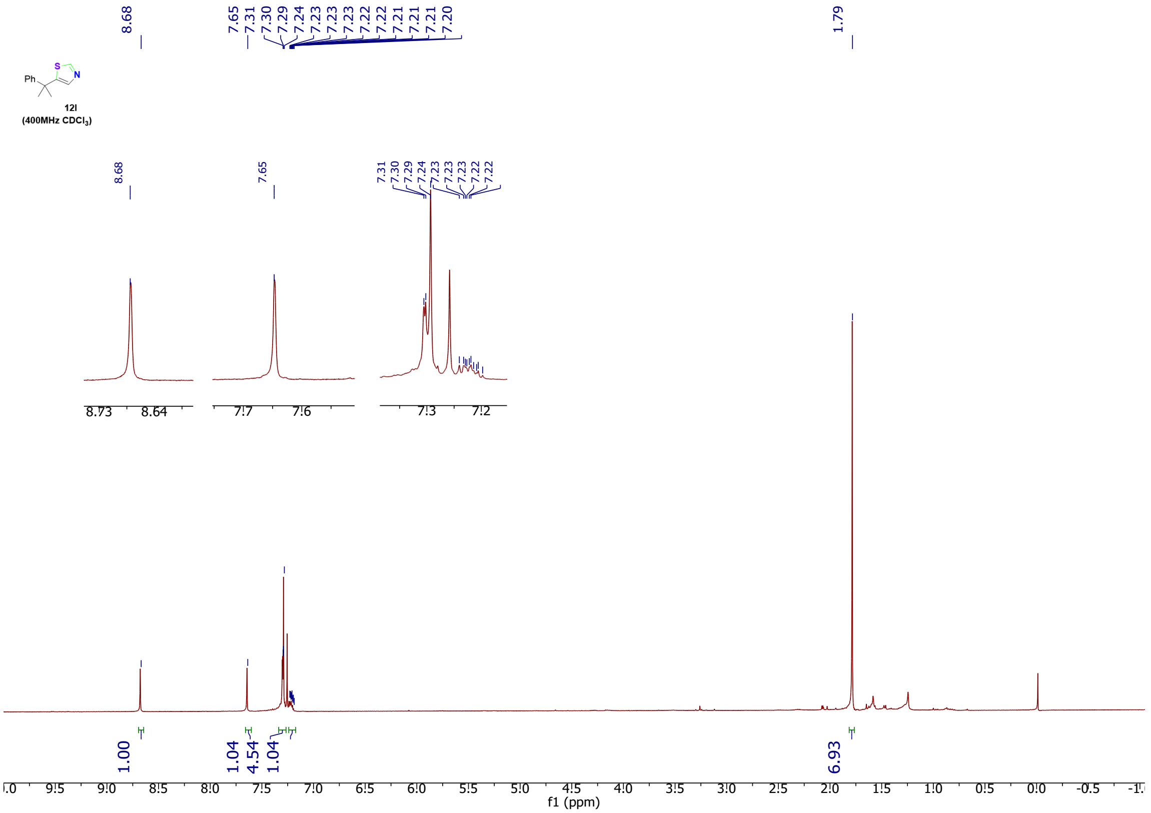

8.68
7.65
7.31
7.30
7.29
7.24
7.23
7.23
7.23
7.22
7.22
7.21
7.21
7.20
1.79
Ph
S
N
12l
(400MHz CDCl3)
1.00
1.04
4.54
1.04
6.93
f1 (ppm)

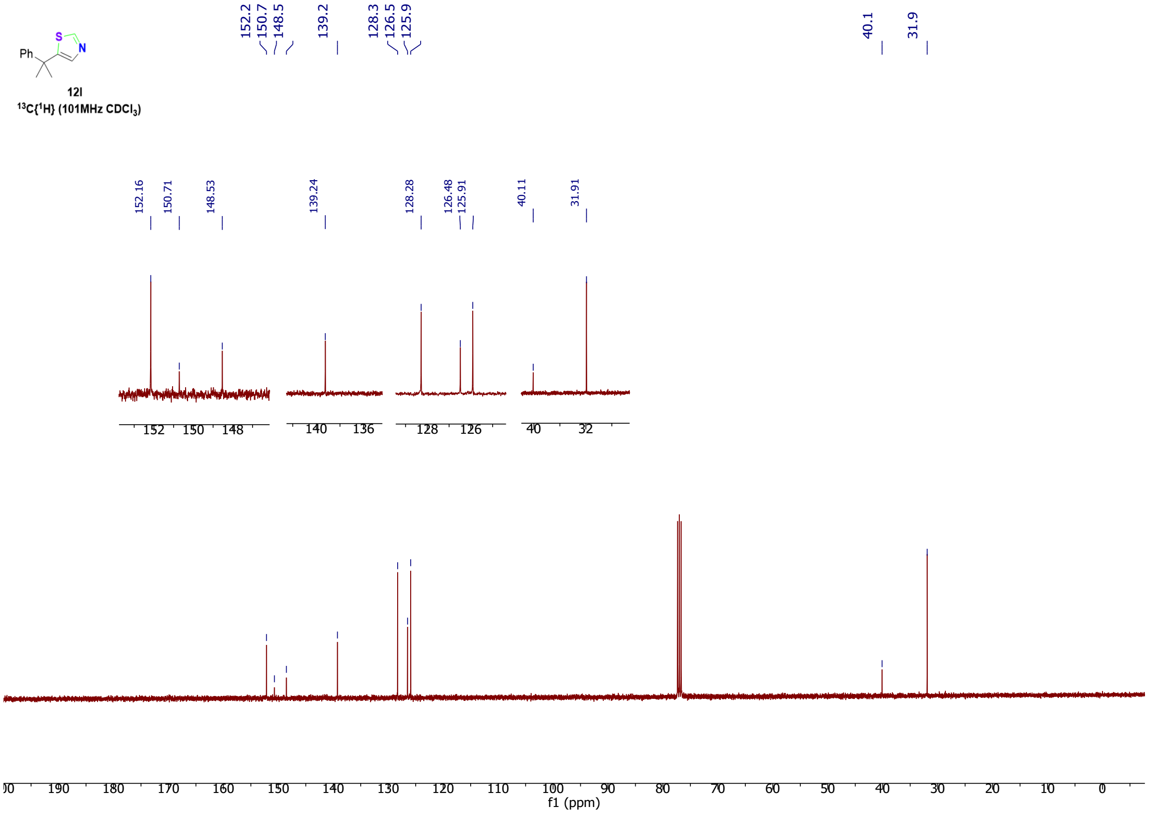

152.2
150.7
148.5
139.2
128.3
126.5
125.9
40.1
31.9
Ph
S
N
12l
13C{1H} (101MHz CDCl3)
152.16
150.71
148.53
139.24
128.28
126.48
125.91
40.11
31.91
f1 (ppm)

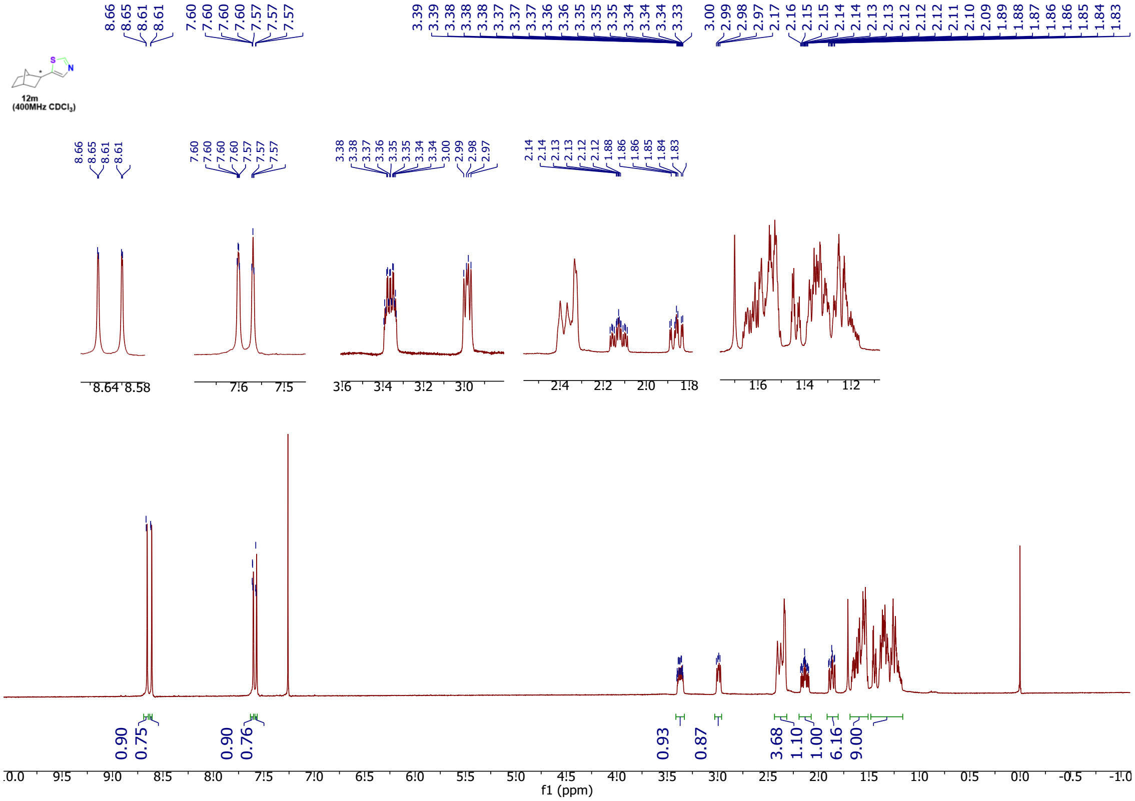
12m
(400MHz CDCl3)
0.90
0.75
0.90
0.76
0.93
0.87
3.68
1.10
1.00
6.16
9.00
f1 (ppm)

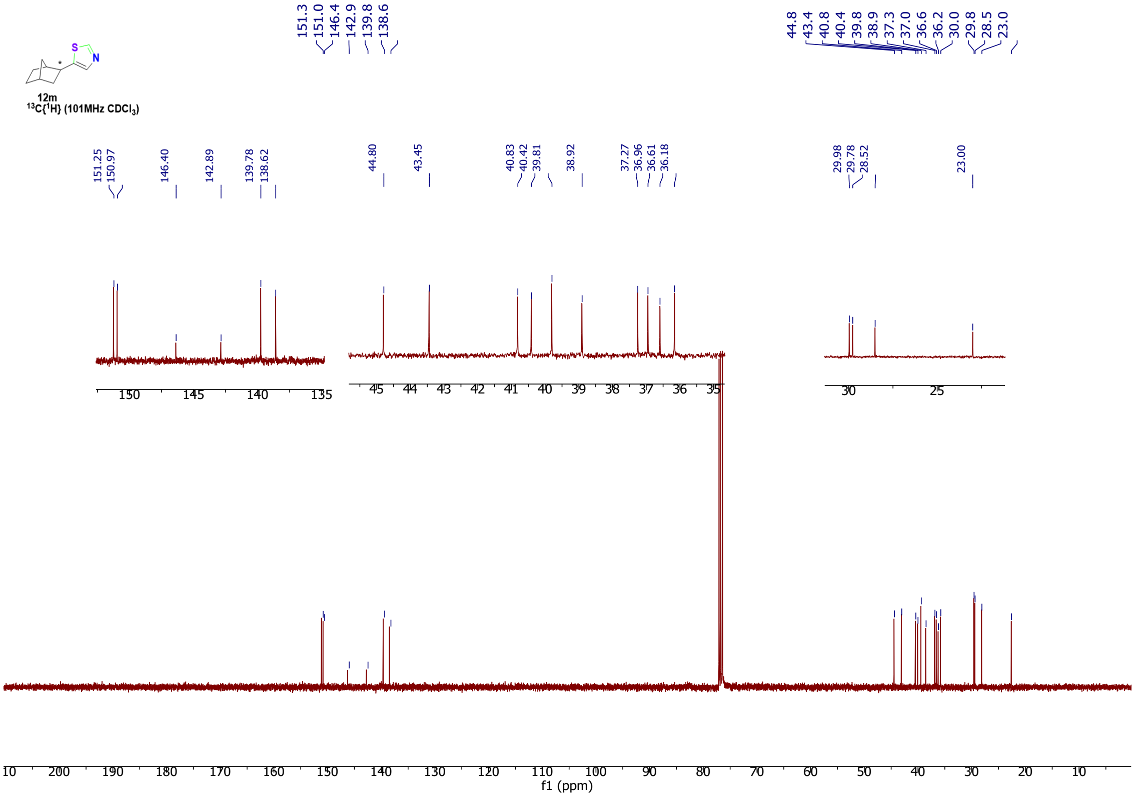
12m
13C{1H} (101MHz CDCl3)
151.25
150.97
146.40
142.89
139.78
138.62
44.80
43.45
40.83
40.42
39.81
38.92
37.27
36.96
36.61
36.18
29.98
29.78
28.52
23.00
f1 (ppm)

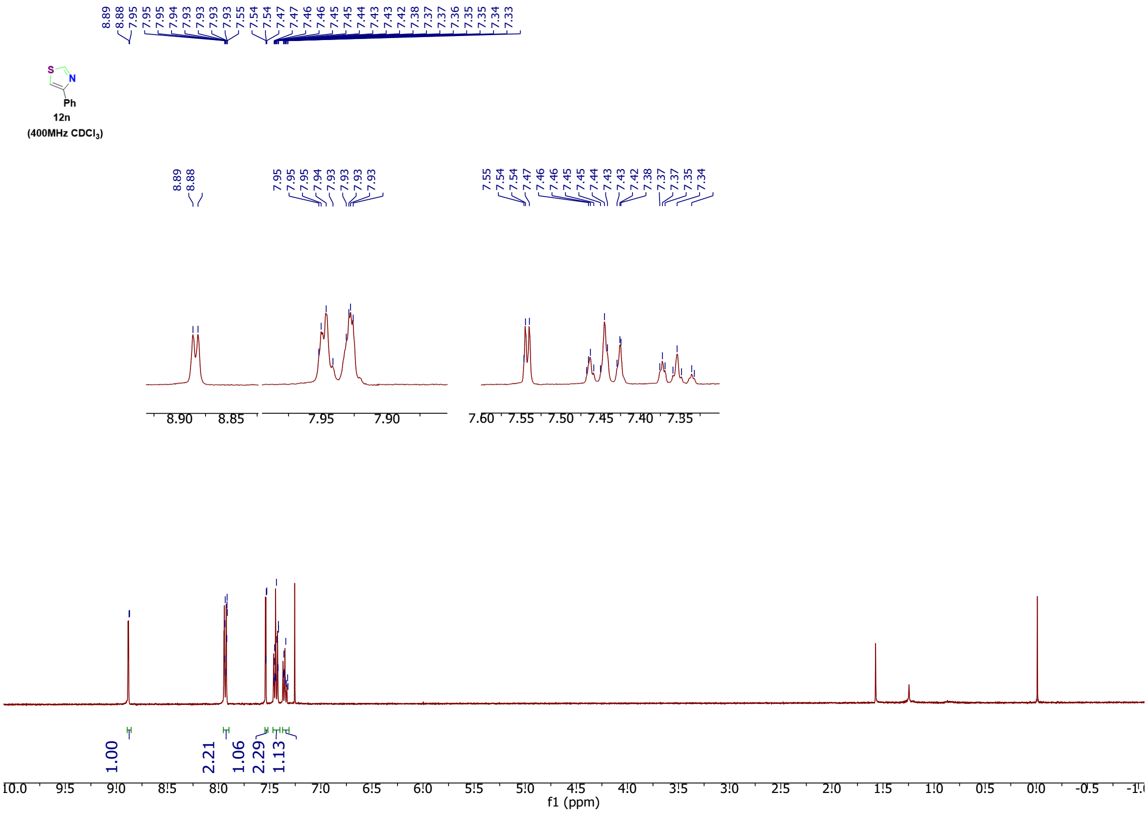

S
N
Ph
12n
(400MHz CDCl3)
1.00
2.21
1.06
2.29
1.13
f1 (ppm)

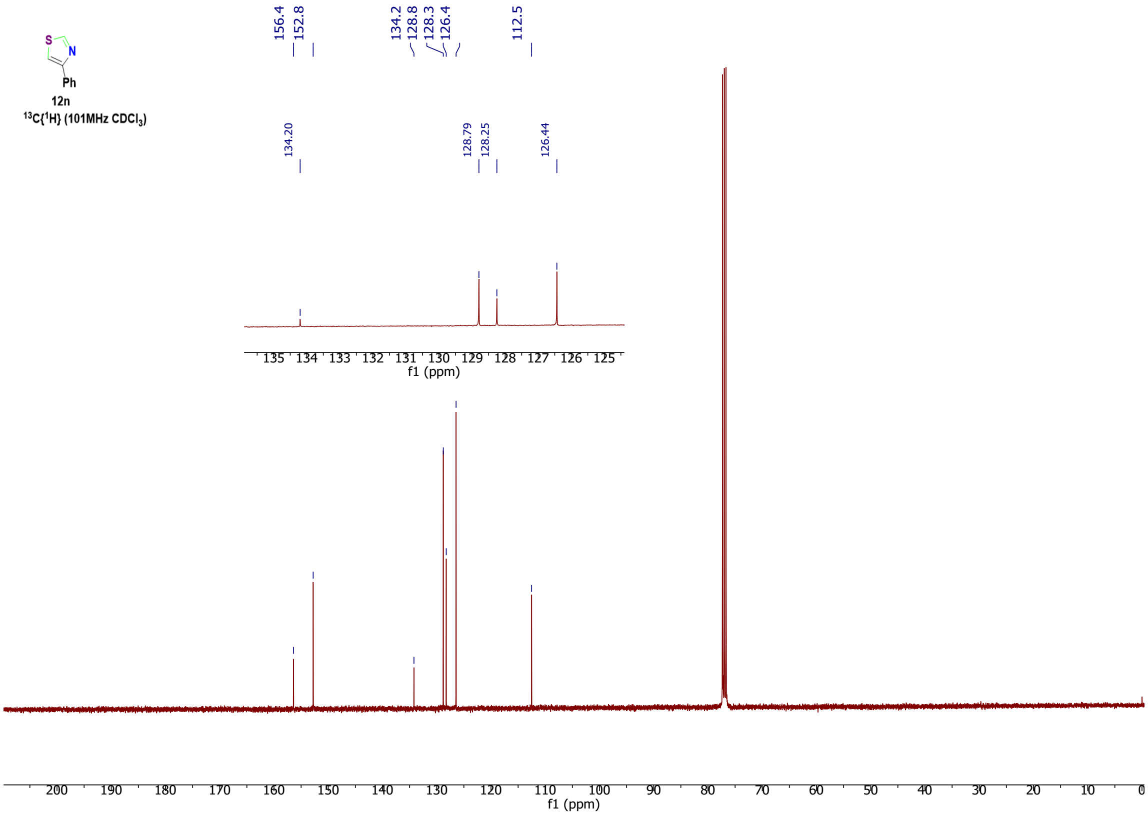

156.4
152.8
134.2
128.8
128.3
126.4
112.5
S
N
Ph
12n
13C{1H} (101MHz CDCl3)
134.20
128.79
128.25
126.44
f1 (ppm)

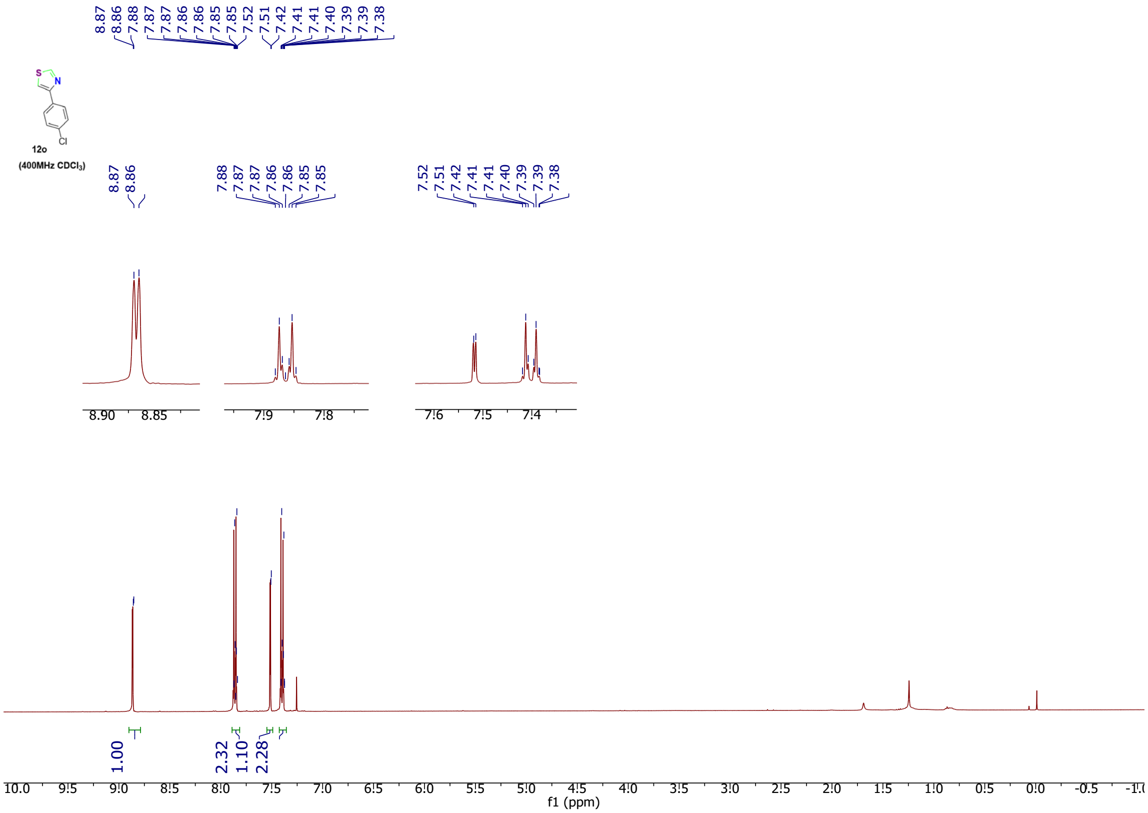
12o
(400MHz CDCl3)
8.87
8.86
7.88
7.87
7.87
7.86
7.86
7.85
7.85
7.52
7.51
7.42
7.41
7.41
7.40
7.39
7.39
7.38
1.00
2.32
1.10
2.28
f1 (ppm)

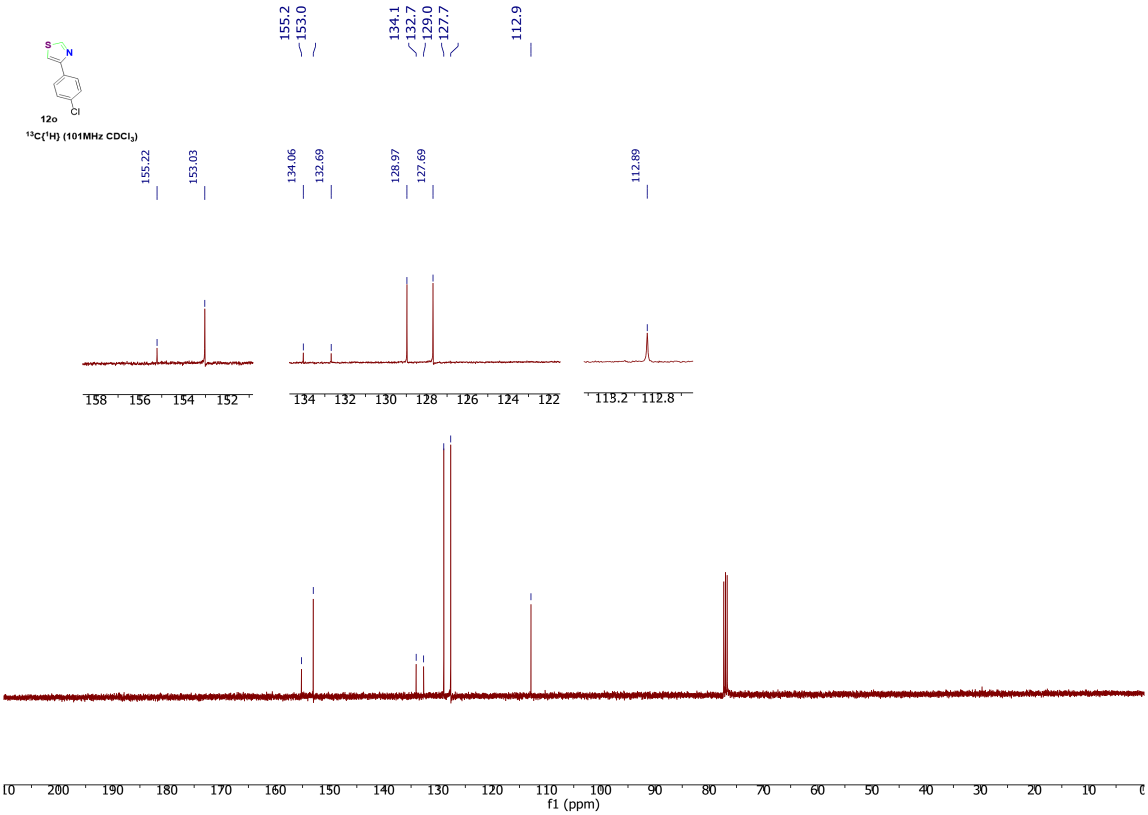
12o
13C{1H} (101MHz CDCl3)
155.2
153.0
134.1
132.7
129.0
127.7
112.9
155.22
153.03
134.06
132.69
128.97
127.69
112.89
f1 (ppm)

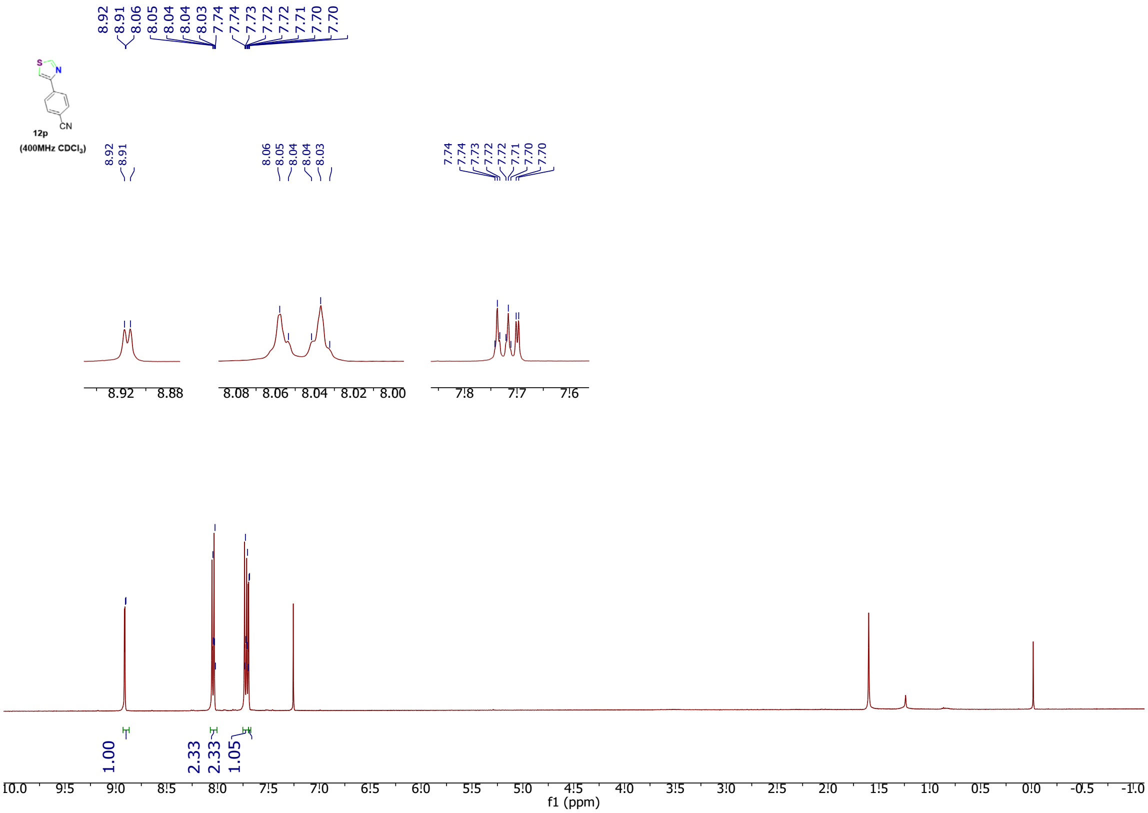
8.92 8.91 8.06 8.05 8.04 8.04 8.03 7.74 7.74 7.73 7.72 7.72 7.71 7.70 7.70
12p
(400MHz CDCl3)
1.00
2.33
2.33
1.05
f1 (ppm)

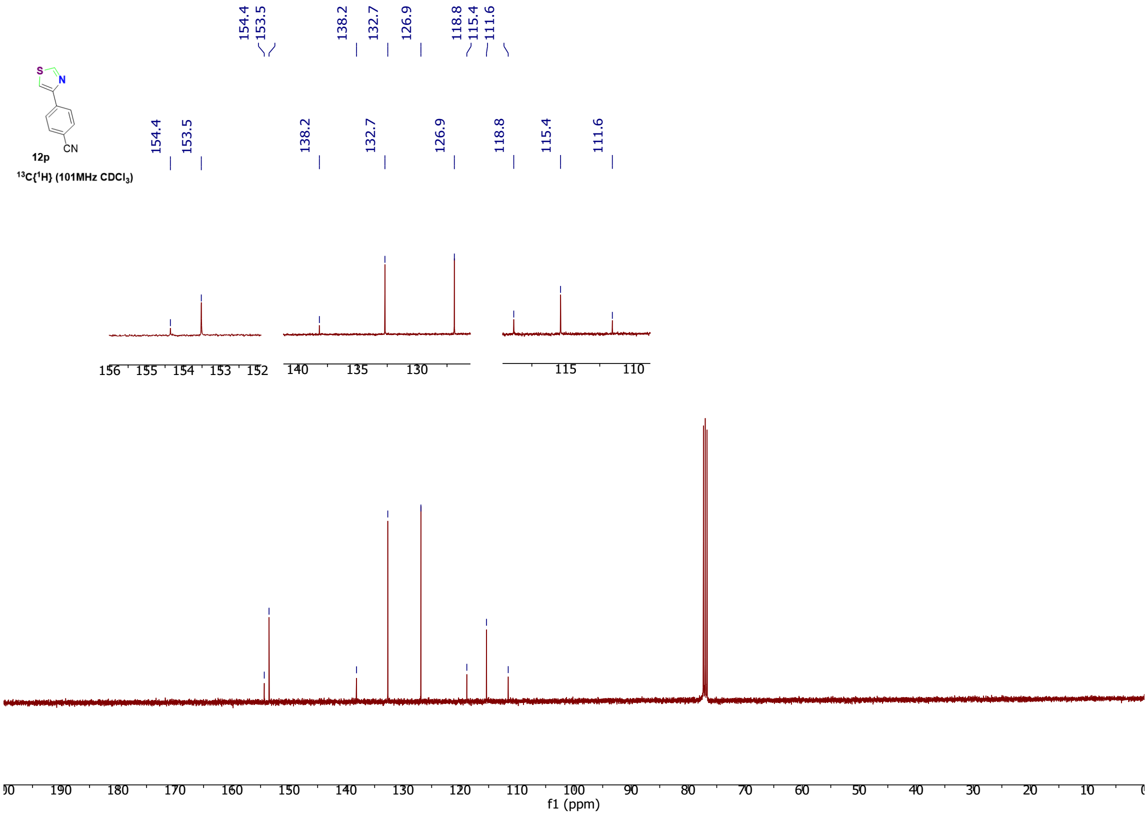
154.4 153.5 138.2 132.7 126.9 118.8 115.4 111.6
12p
13C{1H} (101MHz CDCl3)
f1 (ppm)

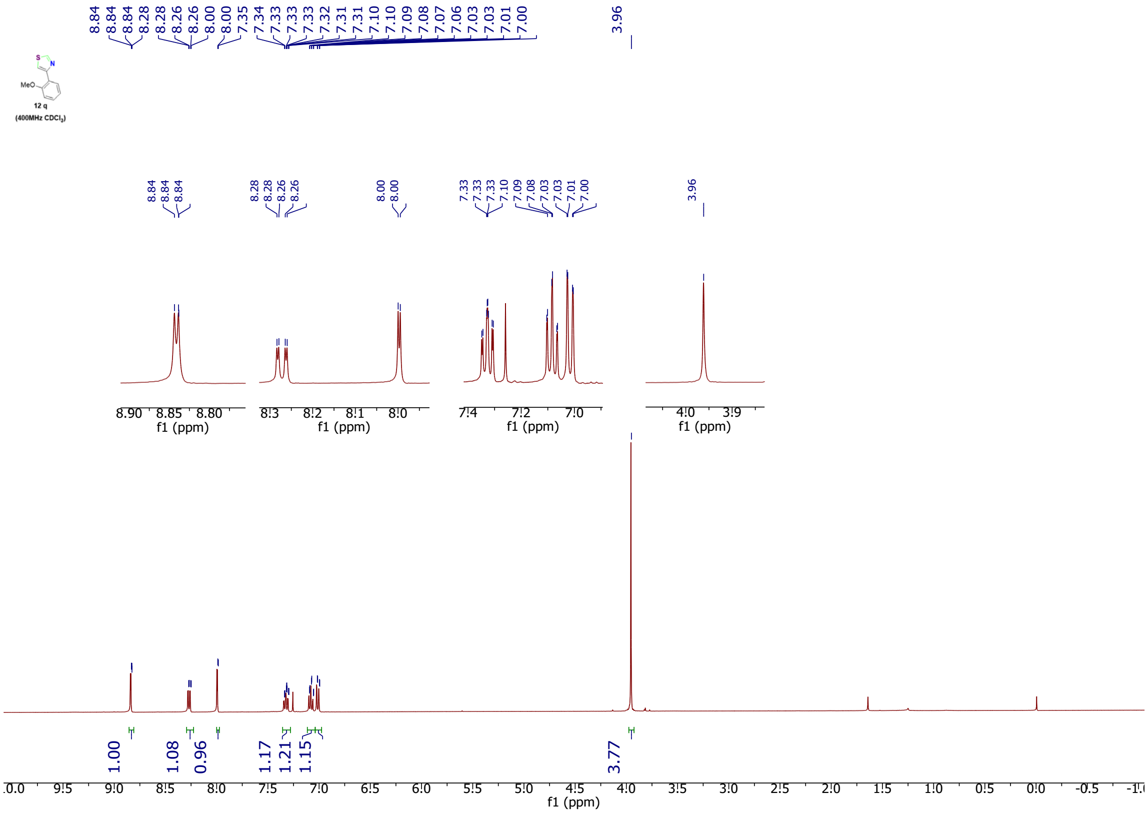

12 q
(400MHz CDCl3)
f1 (ppm)

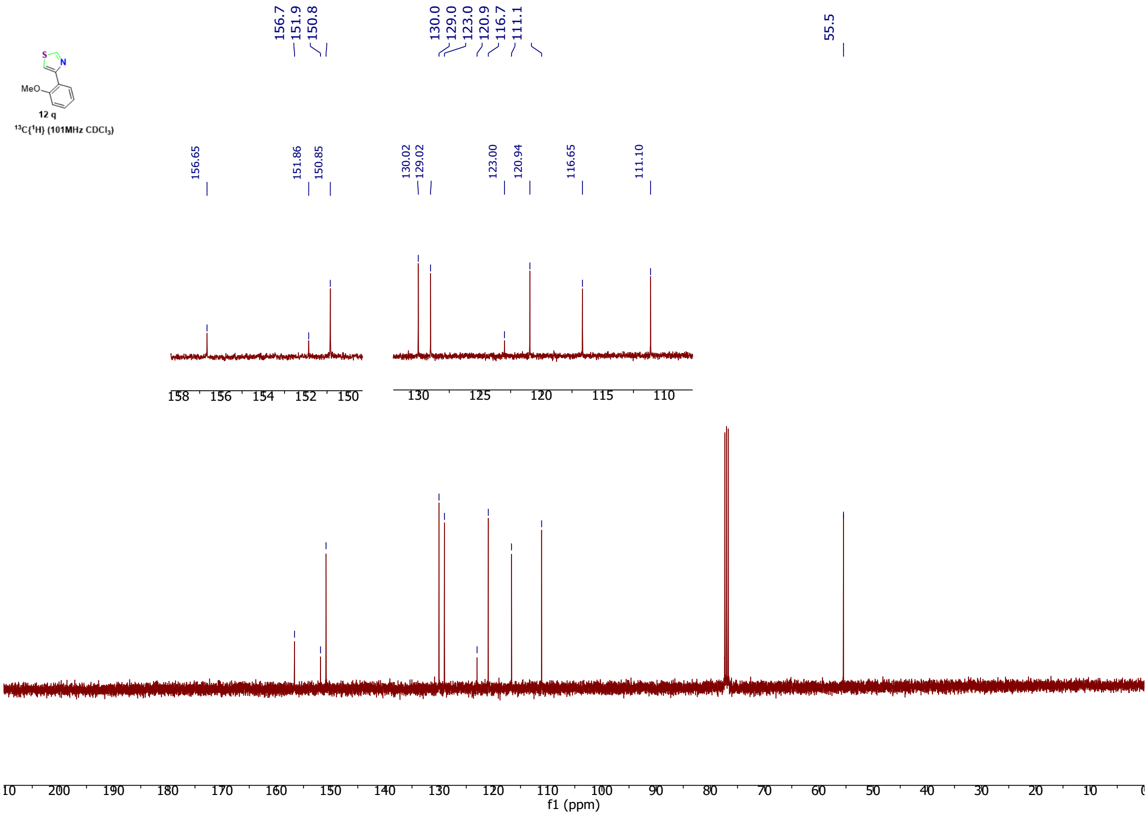

12 q
13C{1H} (101MHz CDCl3)
f1 (ppm)

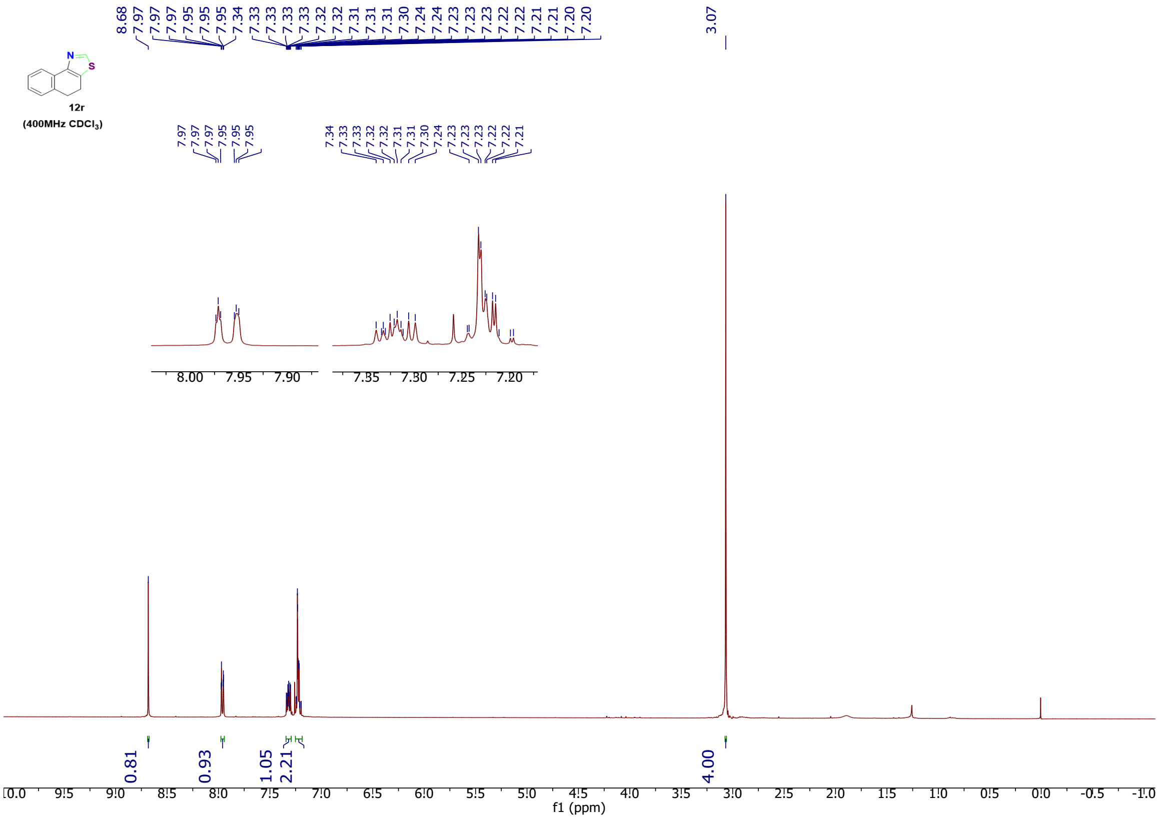
12r
(400MHz CDCl3)
f1 (ppm)

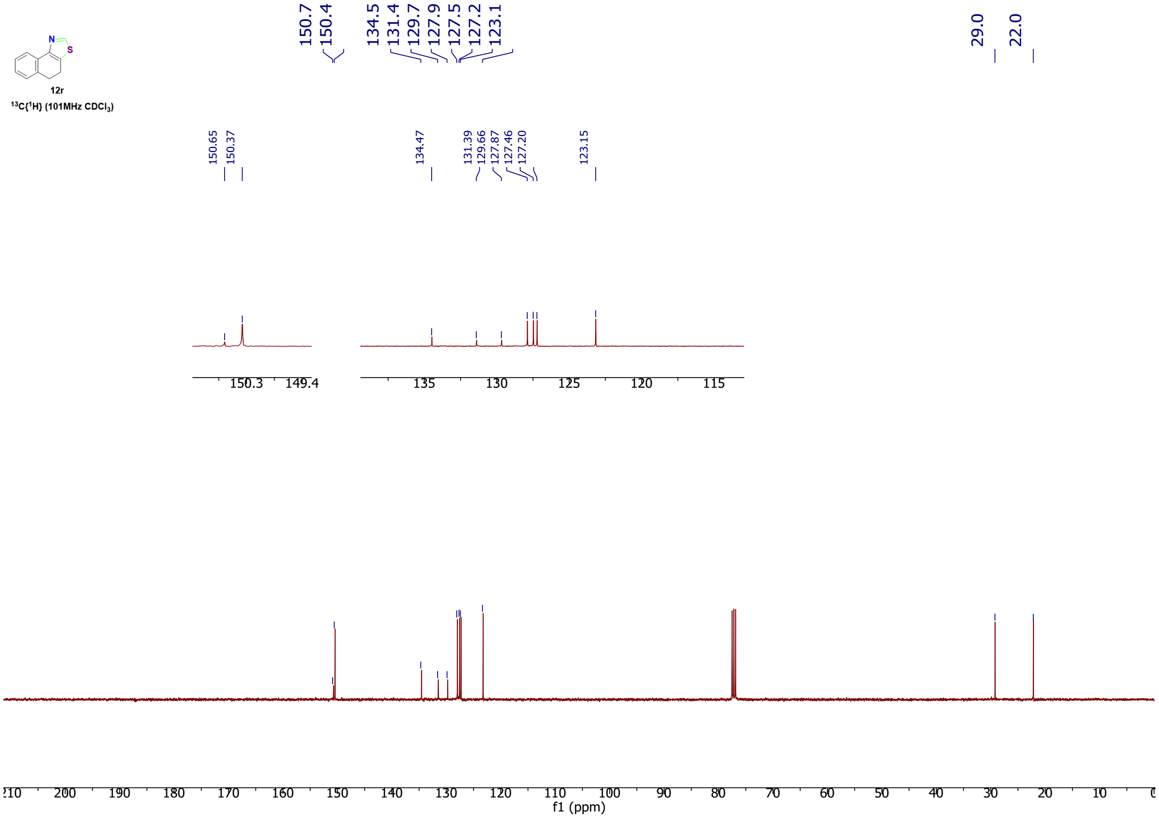
12r
13C{1H} (101MHz CDCl3)
f1 (ppm)

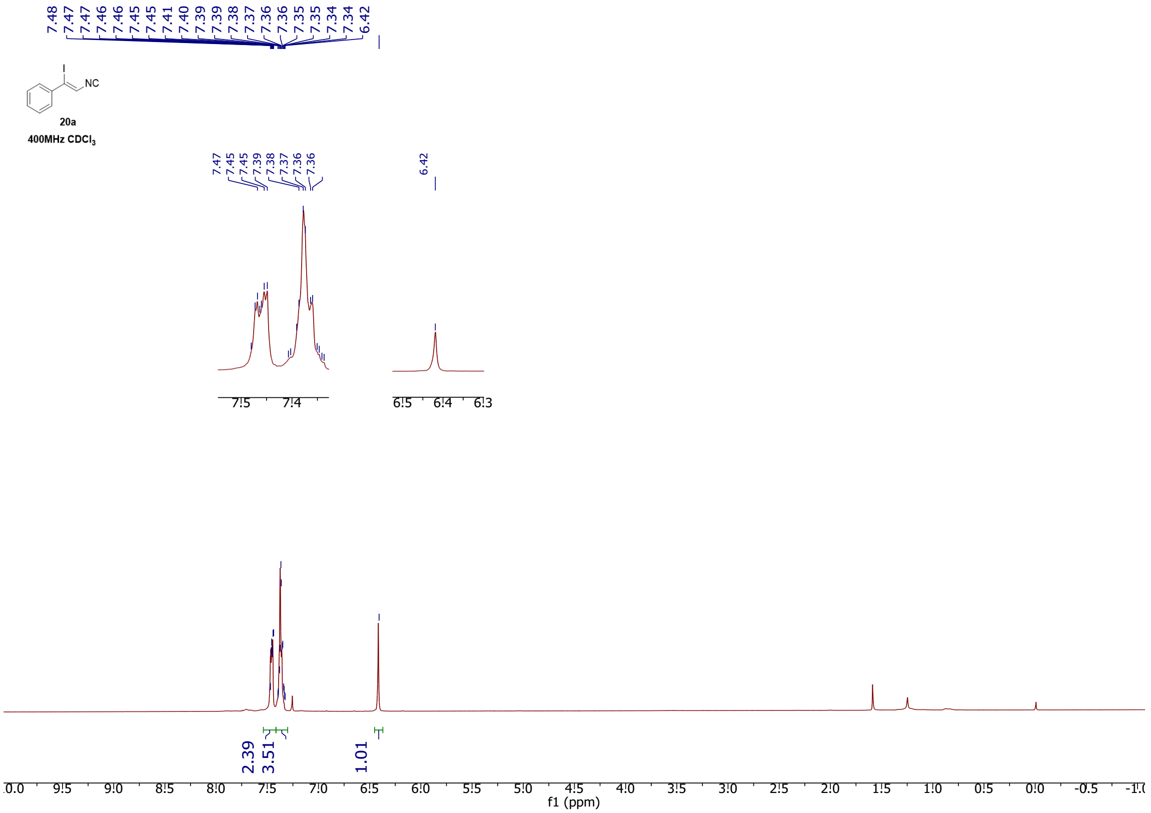

NC
I
20a
400MHz CDCl3
7.48 7.47 7.47 7.46 7.46 7.45 7.45 7.41 7.40 7.39 7.39 7.38 7.37 7.36 7.36 7.35 7.35 7.34 7.34 6.42
2.39
3.51
1.01
f1 (ppm)

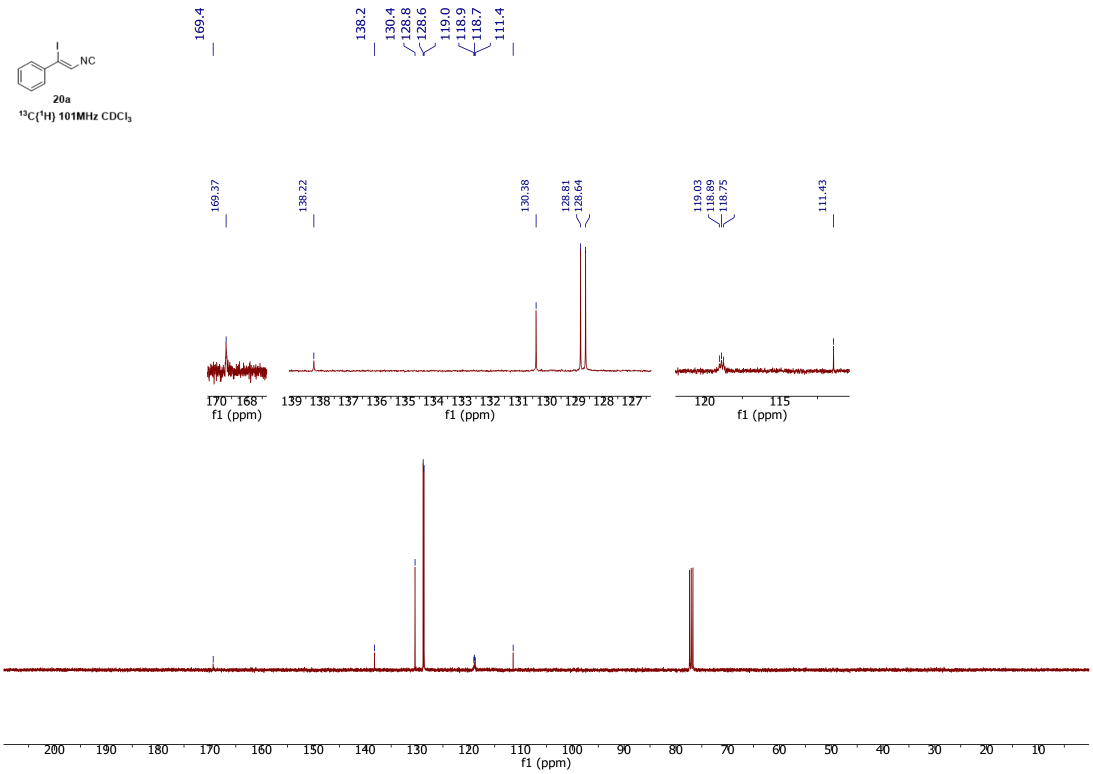

NC
I
20a
13C{1H} 101MHz CDCl3
169.4 138.2 130.4 128.8 128.6 119.0 118.9 118.7 111.4
169.37 138.22 130.38 128.81 128.64 119.03 118.89 118.75 111.43
f1 (ppm)

Figure S2. gNOESY for **20a**

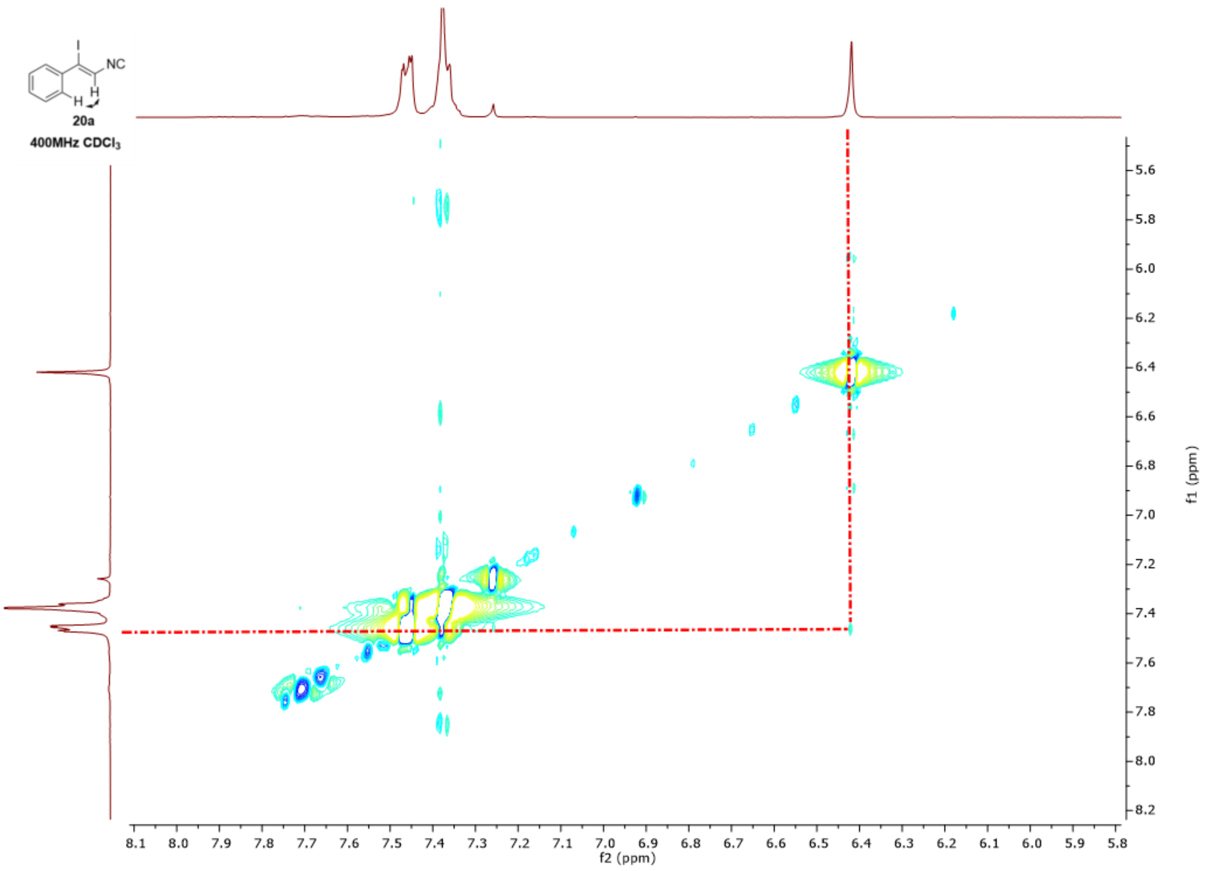

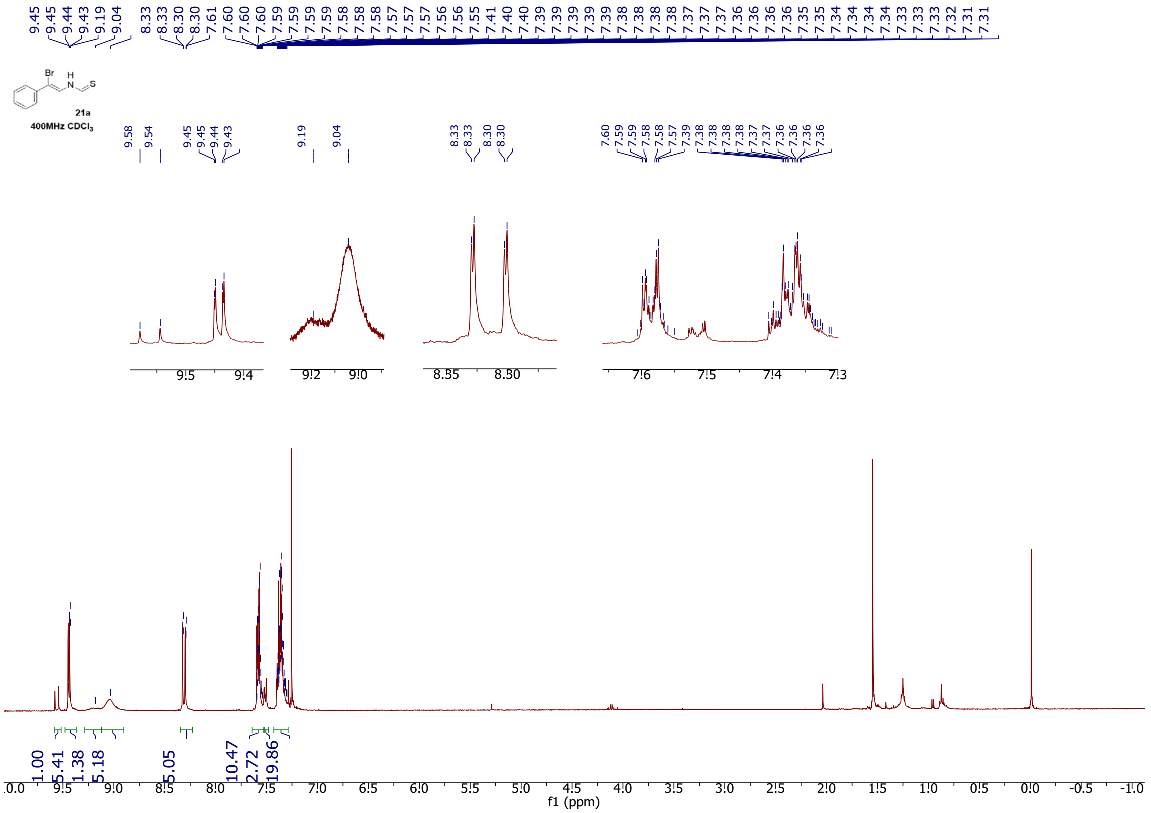

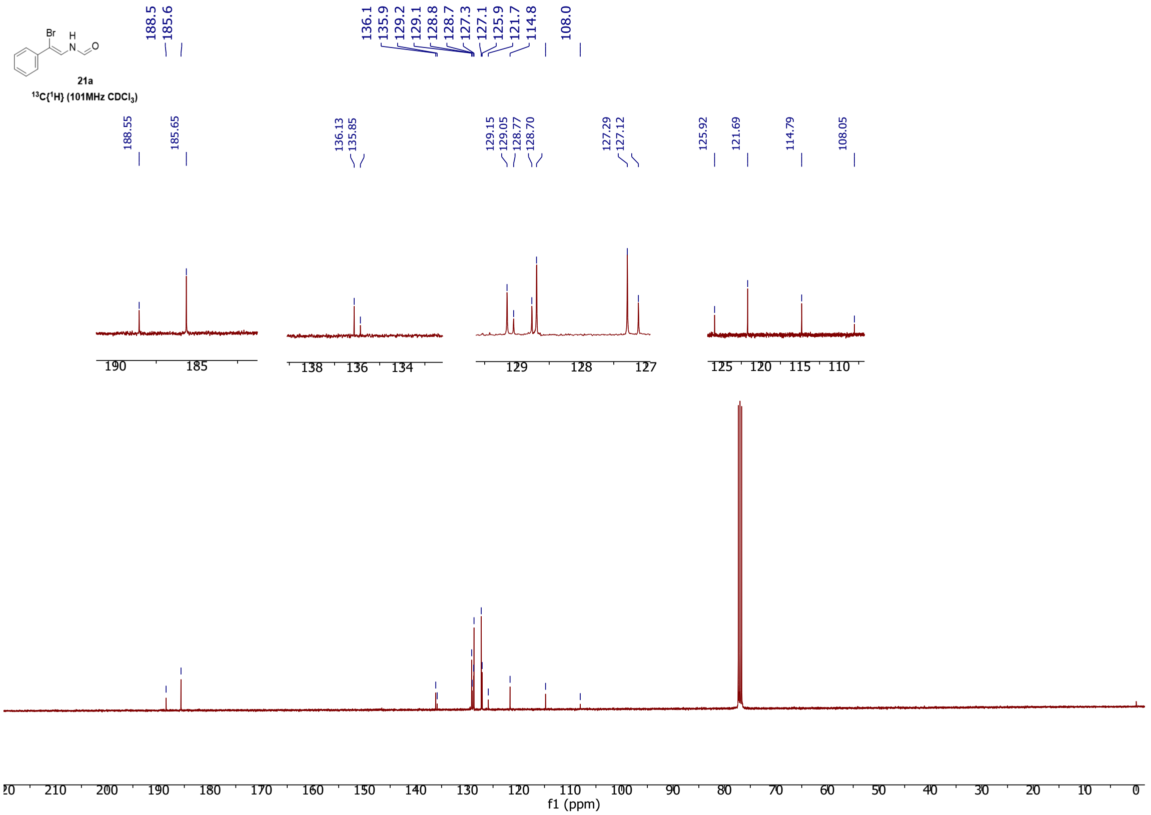

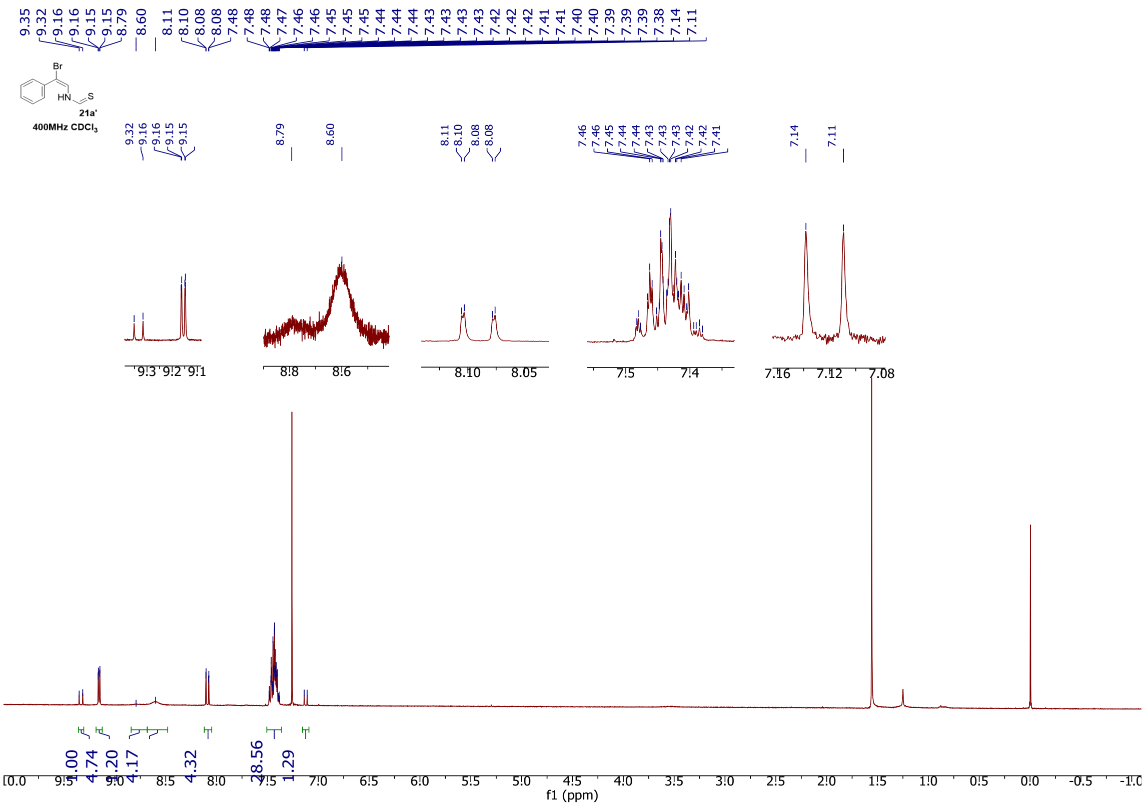
Br
HN
S
21a'
400MHz CDCl3
f1 (ppm)

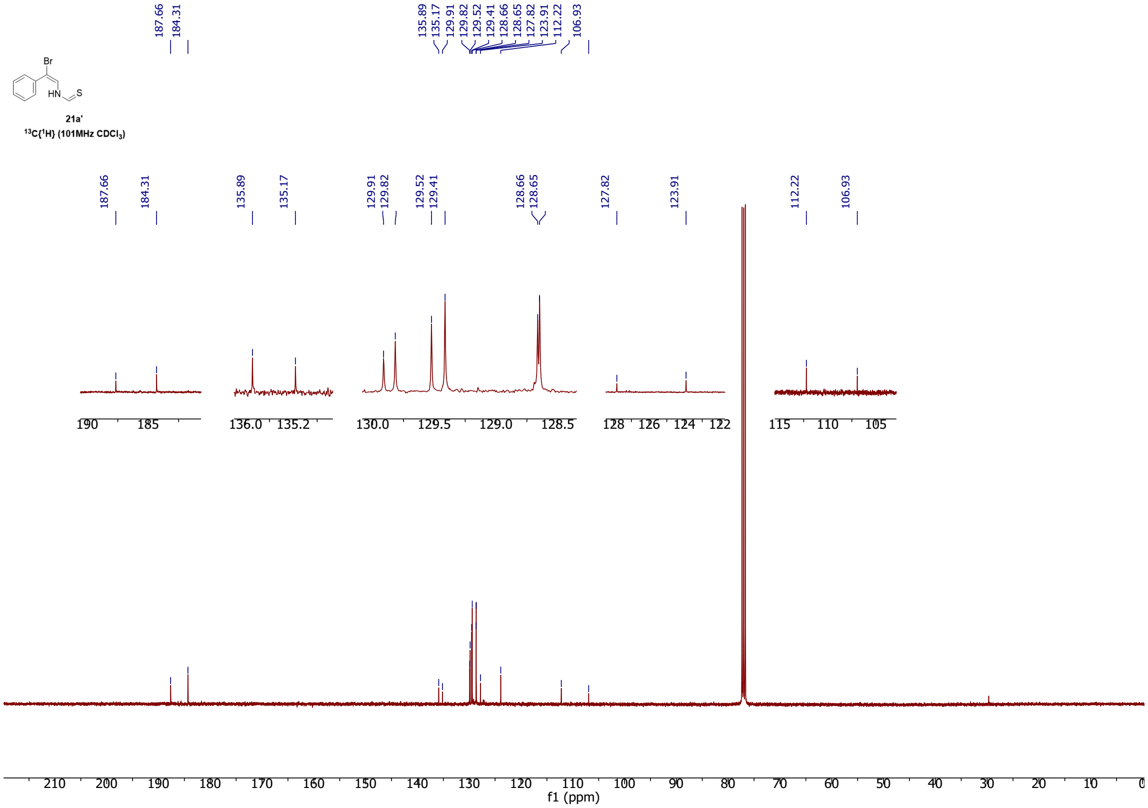
Br
HN
S
21a'
13C{1H} (101MHz CDCl3)
f1 (ppm)

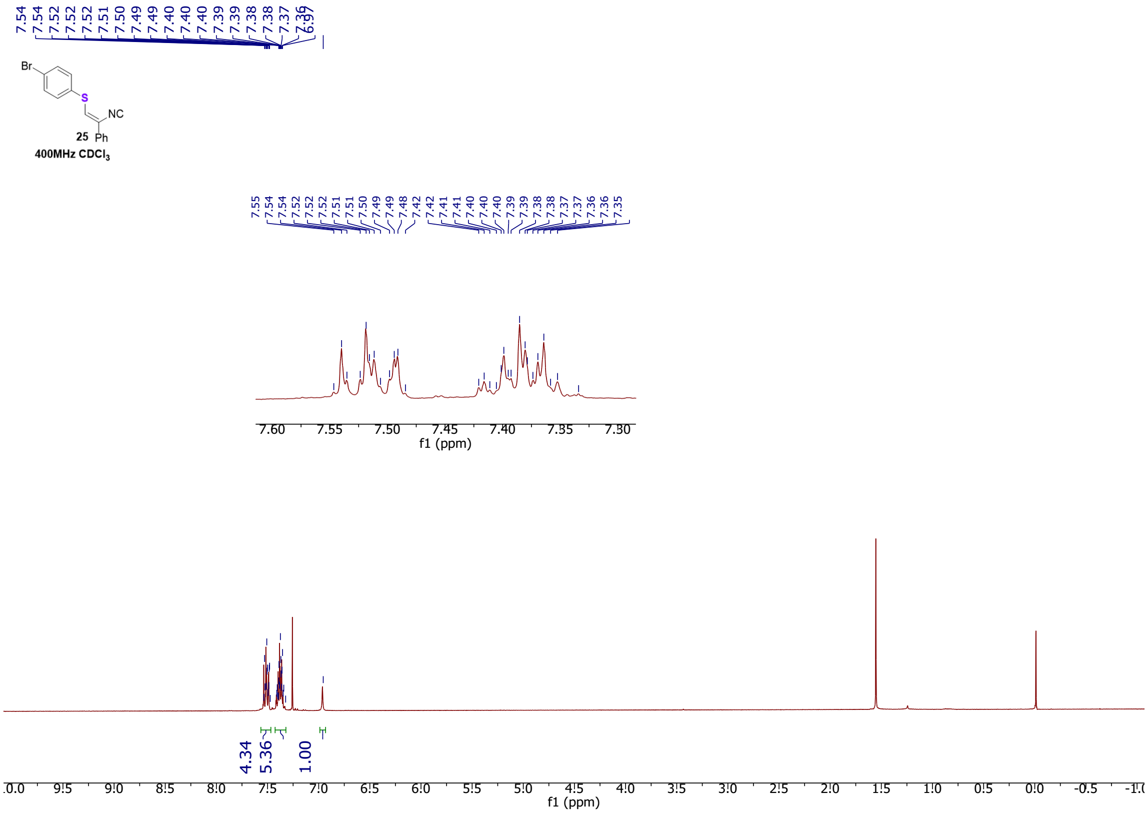
Br
S
NC
25 Ph
400MHz CDCl3
4.34
5.36
1.00
f1 (ppm)

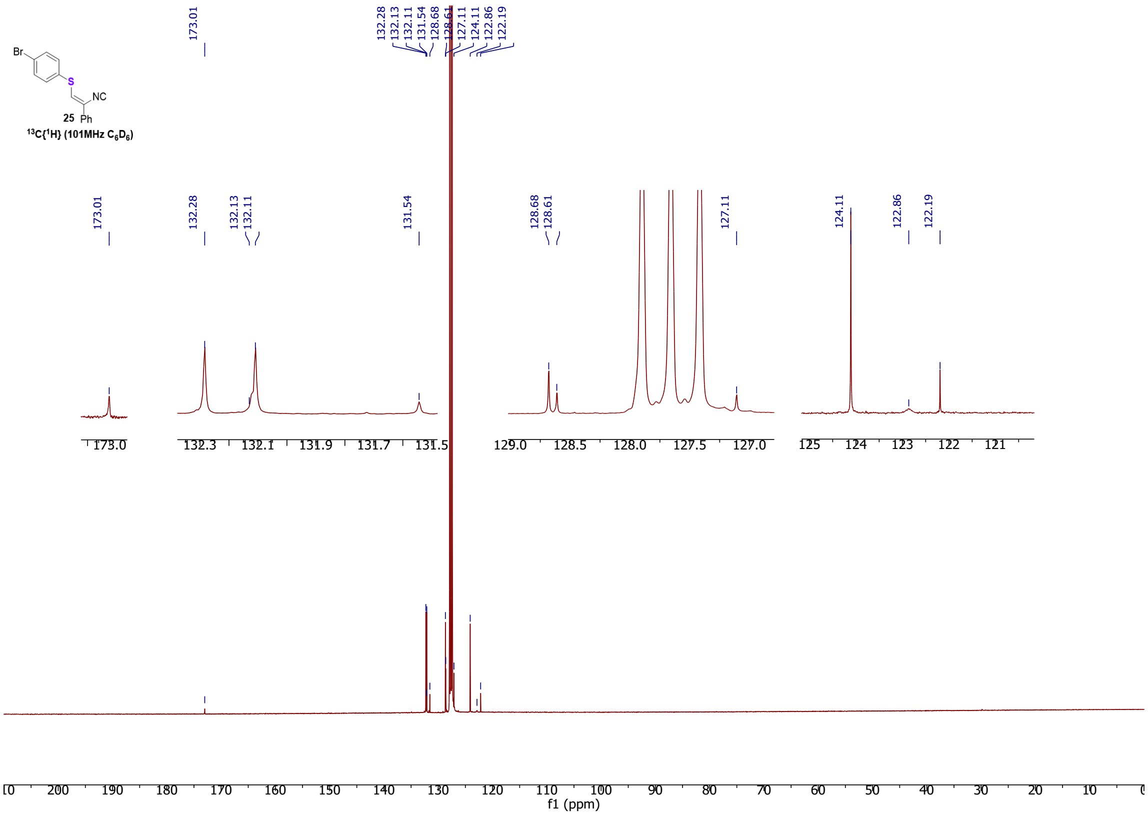
Br
S
NC
25 Ph
13C{1H} (101MHz C6D6)
173.01
132.28
132.13
132.11
131.54
128.68
128.61
127.11
124.11
122.86
122.19
f1 (ppm)

Figure S3. gNOESY for **25**

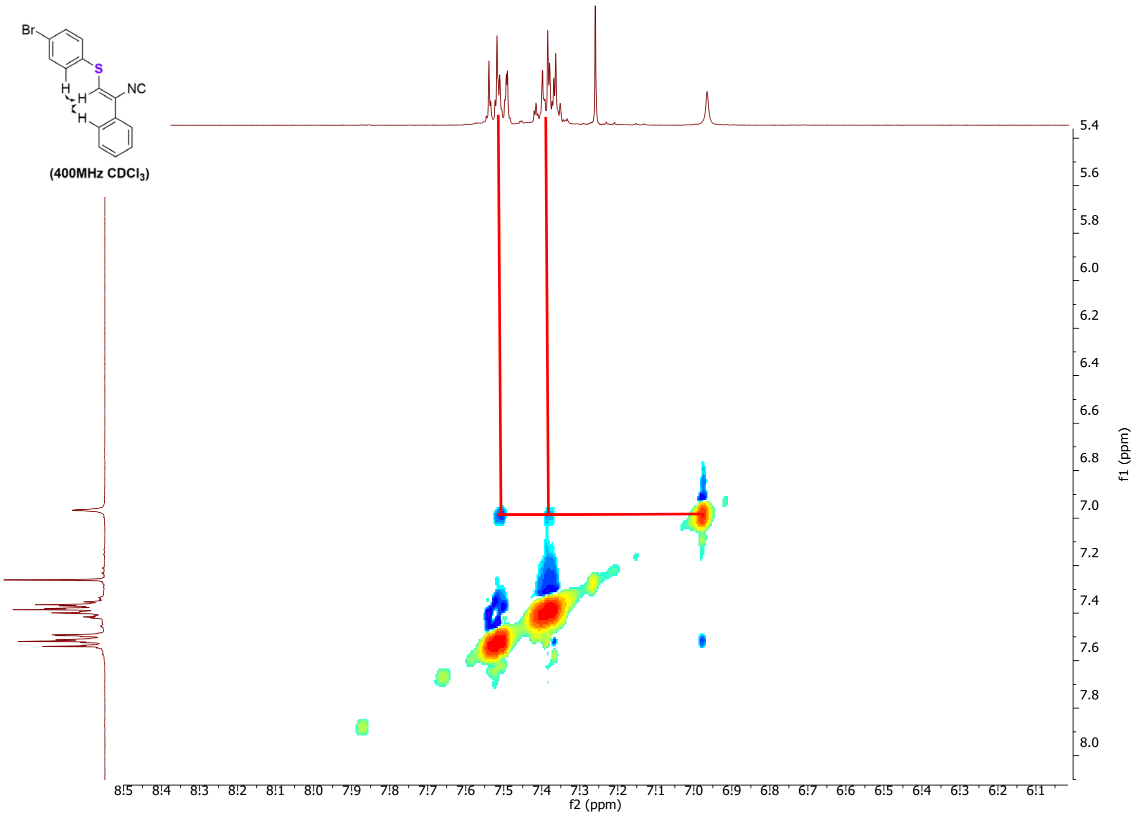

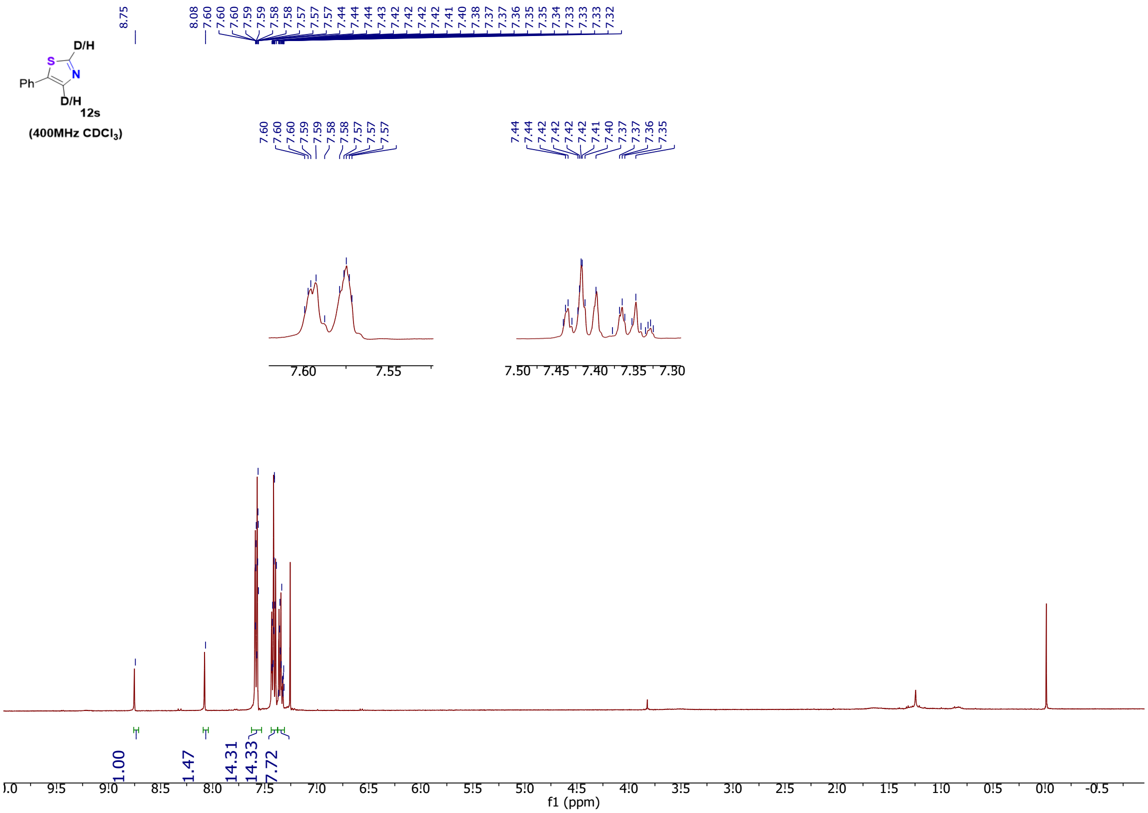

D/H
S
N
Ph
D/H
12s
(400MHz CDCl3)
8.75
8.08
f1 (ppm)
1.00
1.47
14.31
14.33
7.72

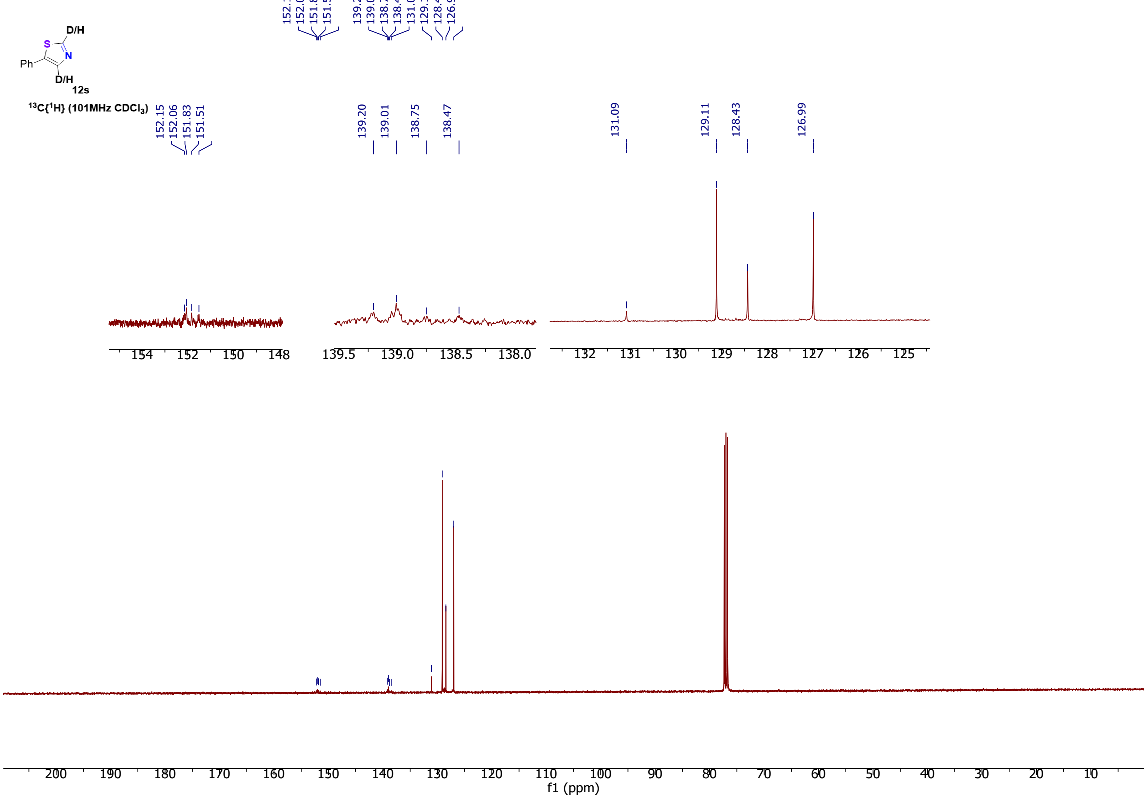

D/H
S
N
Ph
D/H
12s
13C{1H} (101MHz CDCl3)
152.15
152.06
151.83
151.51
139.20
139.01
138.75
138.47
131.09
129.11
128.43
126.99
f1 (ppm)

References

[1] Chao, A.; Alwedi, E.; Fleming, F. F. Isocyanide Purification: C-2 Silica Cleans Up a Dirty Little Secret *Synthesis* **2019,** *51*, 2122-2127.

[2] Fillion, E.; Trepanier, E. V.; Heikkinen, J. J.; Remorova, A. A.; Carson, J. R.; Goll, M. J.; Seed, A. Palladium Catalyzed Intramolecular Reactions of(E)-2,2-Disubstituted 1-Alkenyldimethylalanes with Aryl Triflates *Organometallics* **2009,** *28,* 3518–3531.

[3] Bull, J. A.; Mousseau, J. J.; Charette, A. B. Convenient One-Pot Synthesis of (E)-*β*-Aryl Vinyl Halides from Benzyl Bromides and Dihalomethanes *Org. Lett.* **2008,** *10*, 5485-5488.

[4] White, D. J.; Blakemore, R. P.; Green, J. N.; Bryan Hauser, E., Holoboski, A. M.; Keown, E, L.; Nylund Kolz, S. C.; Phillips, W. B. Total Synthesis of Rhizoxin D, a Potent Antimitotic Agent from the Fungus Rhizopus chinensis *J. Org. Chem.* **2002**, 67, 7750-7760.

[5] Previously isolated but not fully characterized: Liu, Q.; Wang, Z.; Peng, X.; Wong, N.C. Ligand-Free Iron-Catalyzed Carbon(sp2)−Carbon(sp2) Cross-Couplingof Alkenyllithium with Vinyl Halides *J. Org. Chem.* **2018**, 83, 6325–6333.

[6] Beshai, M.; Dhudshia, B.; Mills, R.; Thadani, N, A. Terminal alkynes from aldehydes via dehydrohalogenation of (Z)-1-iodo-1-alkenes with TBAF *Tetrahedron Lett.* **2008,** *49*, 6794–6796.

[7] Dudnik, A. S.; Schwier, T.; Gevorgyan, V. Gold-Catalyzed Double Migration-Benzannulation Cascade toward Naphthalenes. *Org. Lett*. **2008**, *10*, 1465–1468.

[8] Bauer, A.; Di Mauro, G.; Li, J.; Maulide, N. An α-Cyclopropanation of Carbonyl Derivatives by Oxidative Umpolung. *Angew. Chem. Int. Ed.* **2020**, *59*, 18208–18212.

[9] Tian, H.; Holyoke, C. W., Jr.; Fleming, F. F. Stereoselective Synthesis of (E)- and (Z)-Isocyanoalkenes. *Org. Lett*. **2022**, *24*, 8657-8661.

[10] Li, Y.; Fleming, F. F., Direct Conversion of Nitriles into Alkene "Isonitriles". *Angew Chem Int Ed Engl* **2016,** *55*, 14770-14773.

[11] Barton, D. H. R.; Bowles, T.; Husinec, S.; Forbes, J. E.; Llobera, A.; Porter, A. E. A.; Zard, S. Z. Reductive Formylation of Oximes; an Approach to the Synthesis of Vinyl Isonitriles. *Tetrahedron Lett.* **1988**, *29*, 3343-3346.

[12] Farkas, R.; Molnar, A. E.; Acs, P.; Takacs, A.; Kollar. L.; High-yielding synthesis of 1-carboxamido-3,4-dihydronaphthalenes via palladium-catalyzed aminocarbonylation *Tetrahedron* **2013**, *69*, 500-504

[13] (a) Nenajdenko, V. G.; Gulevich, A. V.; Sokolova, N. V.; Mironov, A. V.; Balenkova, E. S. Chiral Isocyanoazides: Efficient Bifunctional Reagents for Bioconjugation *Eur. J. Org. Chem.* **2010**, *1445*–1449. (b) McClure, K. F.; Abramov, Y. A.; Laird, E. R.; Barberia, J. T.; Cai, W.; Carty, T. J.; Cortina, S. R.; Danley, D. E.; Dipesa, A. J.; Donahue, K. M.; Dombroski, M. A.; Elliott, N. C.; Gabel, C. A.; Han, S.; Hynes, T. R.; LeMotte, P. K.; Mansour, M. N.; Marr, E. S.; Letavic, M. A.; Pandit, J.; Ripin, D. B.; Sweeney, F. J.; Tan, D.; Tao, Y. Theoretical and Experimental Design of Atypical Kinase Inhibitors: Application to p38 MAP Kinase. *J. Med. Chem.* **2005**, *48*, 5728–5737. (c) Kotha, S.; Sreenivasachary, N. Synthesis of constrained α-amino acid derivatives via ring-closing metathesis *Bioorg. Med. Chem. Lett.* **1998**, *8*, 257–260.

[14] Sinclair, S. G.; Kukor. J. A.; Imperial, G. K; and Schipper. J. D.; Transition-Metal-Free ipso-Arylative Condensation *Macromolecules* **2020**, *53*, 5169–5176

[15] Tani, S.; Uehara, N. T.; Yamaguchi, J.; Itami, K.; Programmed synthesis of aryl thiazoles through sequential C-H coupling *Chem. Sci.*, **2014**, 5, 123-135
